# Supplementary material for: In-silico genome wide analysis of Mitogen activated protein kinase kinase kinase gene family in C. sinensis
Source: PLoS One. 2021 Nov 4;16(11):e0258657. doi: 10.1371/journal.pone.0258657 (PMC8568164; doi:10.1371/journal.pone.0258657)
Supplement: S2 File — (PDF) [file pone.0258657.s002.pdf]

In-silico genome wide analysis of Mitogen Activated Protein Kinase Kinase Kinase gene family in *C. sinensis*

Abhirup Paul[1†], Anurag P. Srivastava[2†], Shreya Subrahmanya[3], Guoxin Shen[4†*], Neelam Mishra[3*]

[1]Department of Biochemistry

REVA University

Bangalore, Karnataka,

India

[2]Department of life Sciences

Garden City University

Bangalore, Karnataka,

India

[3]Department of Botany

St. Joseph's College autonomous

Bangalore, Karnataka,

India

[4]Sericultural Research Institute,

Zhejiang Academy of Agricultural Sciences

Hangzhou 310021, China

[†]These authors contributed equally to this work.

[*]Corresponding authors:

Guoxin Shen, Ph.D., Professor, Tel: +86-571-86404298; Fax: +86-571-86404298

Email address: guoxin.shen@ttu.edu

Neelam Mishra, Ph.D., Assistant professor

Email address: neelamiitkgp@gmail.com; neelammishra@sjc.ac.in

Orcid id:

1. Abhirup Paul: 0000-0003-2143-7511
2. Neelam Mishra: 0000-0001-6191-5392

TEA028357.1 Length: 628
TEA028357.1 Number of predicted TMHs: 0
TEA028357.1 Exp number of AAs in TMHs: 0.02198
TEA028357.1 Exp number, first 60 AAs: 0.00018
TEA028357.1 Total prob of N-in: 0.00262
TEA028357.1 TMHMM2.0 outside 1 628

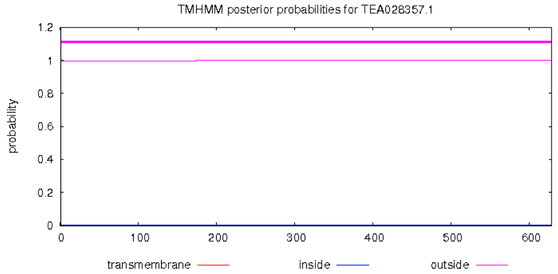

TEA025870.1 Length: 776
TEA025870.1 Number of predicted TMHs: 0
TEA025870.1 Exp number of AAs in TMHs: 0.19882
TEA025870.1 Exp number, first 60 AAs: 0.00141
TEA025870.1 Total prob of N-in: 0.00426
TEA025870.1 TMHMM2.0 outside 1 776

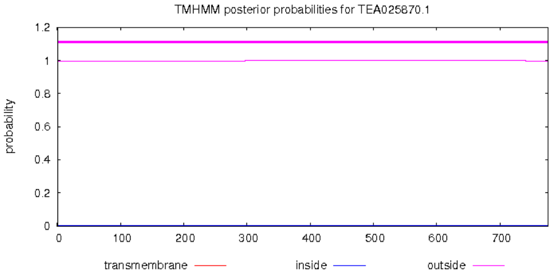

TEA016319.1 Length: 627
TEA016319.1 Number of predicted TMHs: 0
TEA016319.1 Exp number of AAs in TMHs: 0.11408
TEA016319.1 Exp number, first 60 AAs: 3e-05
TEA016319.1 Total prob of N-in: 0.00653
TEA016319.1 TMHMM2.0 outside 1 627

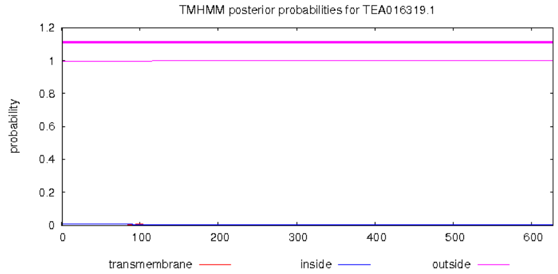

TEA029598.1 Length: 423
TEA029598.1 Number of predicted TMHs: 0
TEA029598.1 Exp number of AAs in TMHs: 0.02545
TEA029598.1 Exp number, first 60 AAs: 0.00568
TEA029598.1 Total prob of N-in: 0.01343
TEA029598.1 TMHMM2.0 outside 1 423

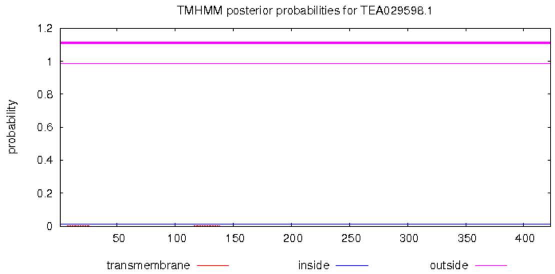

TEA008165.1 Length: 1032
TEA008165.1 Number of predicted TMHs: 0
TEA008165.1 Exp number of AAs in TMHs: 6.76661
TEA008165.1 Exp number, first 60 AAs: 3.17779
TEA008165.1 Total prob of N-in: 0.04338
TEA008165.1 TMHMM2.0 outside 1 1032

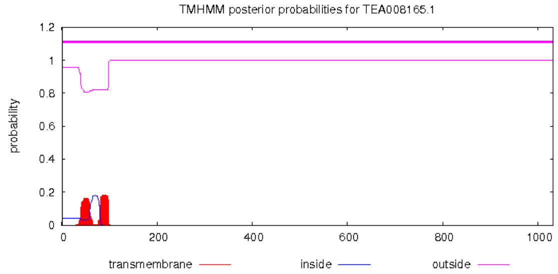

TEA027265.1 Length: 939
TEA027265.1 Number of predicted TMHs: 0
TEA027265.1 Exp number of AAs in TMHs: 0
TEA027265.1 Exp number, first 60 AAs: 0
TEA027265.1 Total prob of N-in: 0.00001
TEA027265.1 TMHMM2.0 outside 1 939

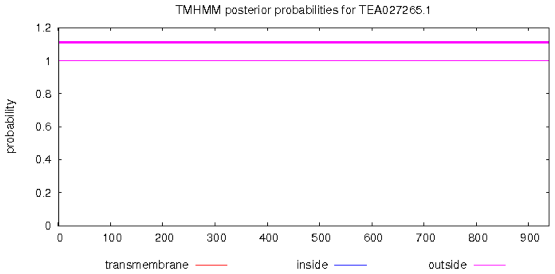

TEA006319.1 Length: 683
TEA006319.1 Number of predicted TMHs: 0
TEA006319.1 Exp number of AAs in TMHs: 0.00042
TEA006319.1 Exp number, first 60 AAs: 0
TEA006319.1 Total prob of N-in: 0.00017
TEA006319.1 TMHMM2.0 outside 1 683

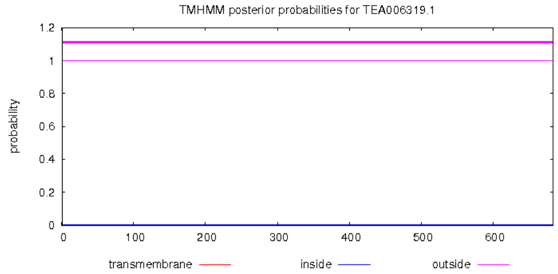

TEA006473.1 Length: 710
TEA006473.1 Number of predicted TMHs: 0
TEA006473.1 Exp number of AAs in TMHs: 0.00515
TEA006473.1 Exp number, first 60 AAs: 0
TEA006473.1 Total prob of N-in: 0.00031
TEA006473.1 TMHMM2.0 outside 1 710

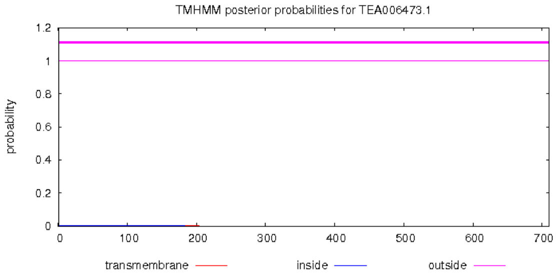

‡ **TEA014429.1** Length: 1191

```
# TEA014429.1 Number of predicted TMHs:  0
# TEA014429.1 Exp number of AAs in TMHs: 0.02164
# TEA014429.1 Exp number, first 60 AAs:  0.00074
# TEA014429.1 Total prob of N-in:        0.00023
TEA014429.1    TMHMM2.0        outside      1  1191
```

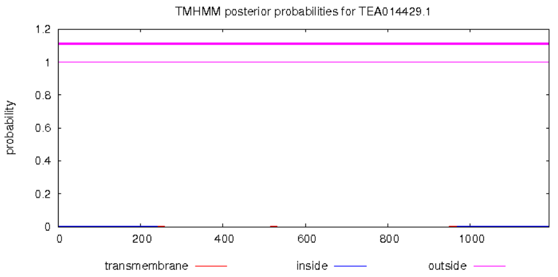

‡ **TEA031711.1** Length: 562

```
# TEA031711.1 Number of predicted TMHs:  0
# TEA031711.1 Exp number of AAs in TMHs: 0.0069899
# TEA031711.1 Exp number, first 60 AAs:  0.00016
# TEA031711.1 Total prob of N-in:        0.00833
TEA031711.1    TMHMM2.0        outside      1   562
```

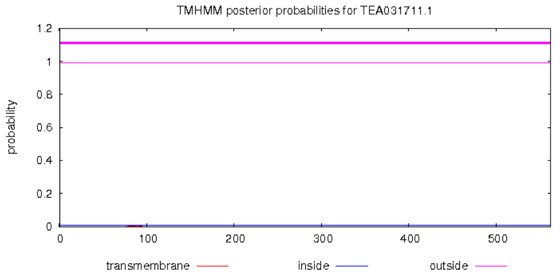

† **TEA001470.1** Length: 789

```
# TEA001470.1 Number of predicted TMHs:  0
# TEA001470.1 Exp number of AAs in TMHs: 3.06197
# TEA001470.1 Exp number, first 60 AAs:  0.21965
# TEA001470.1 Total prob of N-in:        0.01032
TEA001470.1    TMHMM2.0        outside      1   789
```

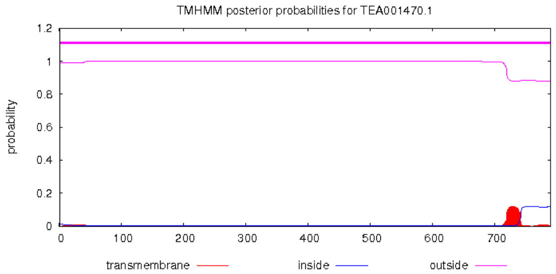

† **TEA017119.1** Length: 506

```
# TEA017119.1 Number of predicted TMHs:  0
# TEA017119.1 Exp number of AAs in TMHs: 0.04644
# TEA017119.1 Exp number, first 60 AAs:  0.00226
# TEA017119.1 Total prob of N-in:        0.01372
TEA017119.1    TMHMM2.0        outside      1   506
```

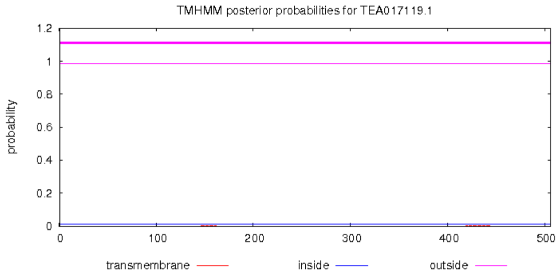

† **TEA005306.1** Length: 1097

```
# TEA005306.1 Number of predicted TMHs:  0
# TEA005306.1 Exp number of AAs in TMHs: 0.03434
# TEA005306.1 Exp number, first 60 AAs:  0
# TEA005306.1 Total prob of N-in:        0.00020
TEA005306.1    TMHMM2.0        outside      1  1097
```

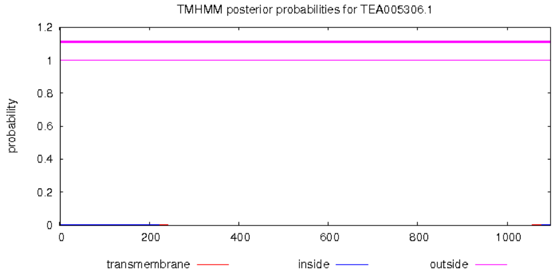

† **TEA009902.1** Length: 450

```
# TEA009902.1 Number of predicted TMHs:  0
# TEA009902.1 Exp number of AAs in TMHs: 0.71758
# TEA009902.1 Exp number, first 60 AAs:  0.10658
# TEA009902.1 Total prob of N-in:        0.00748
TEA009902.1    TMHMM2.0        outside      1   450
```

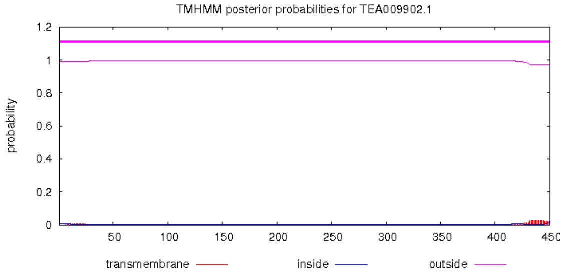

† **TEA005122.1** Length: 334

```
# TEA005122.1 Number of predicted TMHs:  0
# TEA005122.1 Exp number of AAs in TMHs: 0.01241
# TEA005122.1 Exp number, first 60 AAs:  0.00929
# TEA005122.1 Total prob of N-in:        0.04268
TEA005122.1    TMHMM2.0        outside      1   334
```

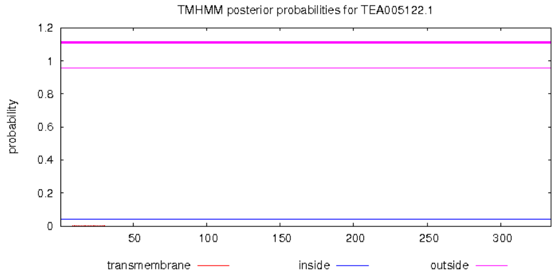

† **TEA028214.1** Length: 344

```
# TEA028214.1 Number of predicted TMHs:  0
# TEA028214.1 Exp number of AAs in TMHs: 0.00766
# TEA028214.1 Exp number, first 60 AAs:  0.00225
# TEA028214.1 Total prob of N-in:        0.02030
TEA028214.1    TMHMM2.0        outside      1   344
```

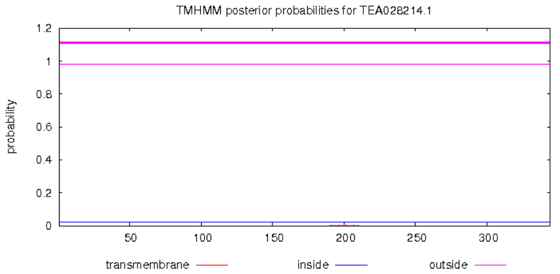

```
# TEA031689.1 Length: 311
# TEA031689.1 Number of predicted TMHs:  0
# TEA031689.1 Exp number of AAs in TMHs: 0.28379
# TEA031689.1 Exp number, first 60 AAs:  0.25957
# TEA031689.1 Total prob of N-in:        0.04973
TEA031689.1     TMHMM2.0        outside      1   311
```

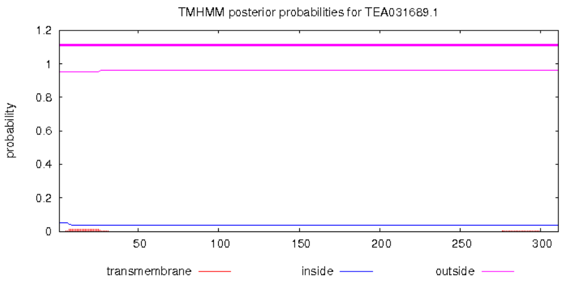

S1 Fig. Transmembrane helices of MAPKKKs of MEKK subfamily in *C. sinensis*. TMHMM Server, v.2.0 (http://www.cbs.dtu.dk/services/TMHMM/), was used to predict the presence of transmembrane helices. The plot shows the posterior probabilities of inside/outside/TM helix. The plot is obtained by calculating the total probability that a residue sits in helix, inside, or outside summed over all possible paths through the model. The red peaks represent the possible transmembrane helices present. If the red peak crosses the transmembrane line (represented in pink colour), then its calculated as 1 transmembrane helix.

```
# TEA001765.1 Length: 842
# TEA001765.1 Number of predicted TMHs:  0
# TEA001765.1 Exp number of AAs in TMHs: 1.26294
# TEA001765.1 Exp number, first 60 AAs:  0
# TEA001765.1 Total prob of N-in:        0.00418
TEA001765.1     TMHMM2.0        outside      1   842
```

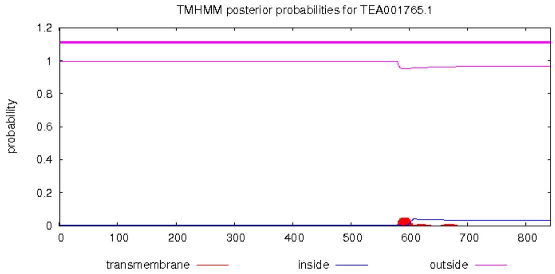


```
# TEA002020.1 Length: 896
# TEA002020.1 Number of predicted TMHs:  0
# TEA002020.1 Exp number of AAs in TMHs: 0.01584
# TEA002020.1 Exp number, first 60 AAs:  0
# TEA002020.1 Total prob of N-in:        0.00015
TEA002020.1     TMHMM2.0        outside      1   896
```

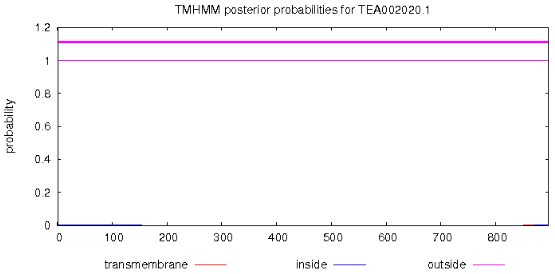


```
# TEA000256.1 Length: 1086
# TEA000256.1 Number of predicted TMHs:  0
# TEA000256.1 Exp number of AAs in TMHs: 0.00826
# TEA000256.1 Exp number, first 60 AAs:  0
# TEA000256.1 Total prob of N-in:        0.00004
TEA000256.1     TMHMM2.0        outside      1  1086
```

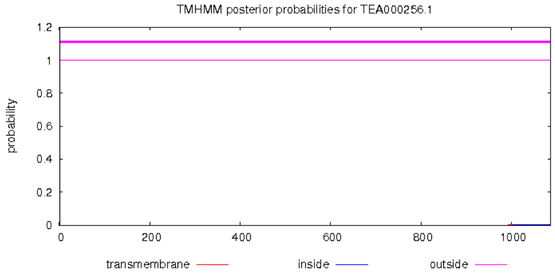


```
# TEA029086.1 Length: 919
# TEA029086.1 Number of predicted TMHs:  0
# TEA029086.1 Exp number of AAs in TMHs: 0.27701
# TEA029086.1 Exp number, first 60 AAs:  0
# TEA029086.1 Total prob of N-in:        0.00050
TEA029086.1     TMHMM2.0        outside      1   919
```

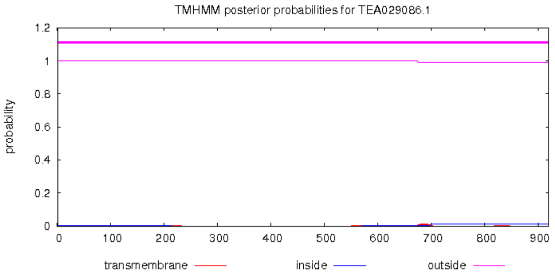


```
# TEA022129.1 Length: 940
# TEA022129.1 Number of predicted TMHs:  0
# TEA022129.1 Exp number of AAs in TMHs: 37.32592
# TEA022129.1 Exp number, first 60 AAs:  0
# TEA022129.1 Total prob of N-in:        0.17108
TEA022129.1     TMHMM2.0        outside      1   940
```

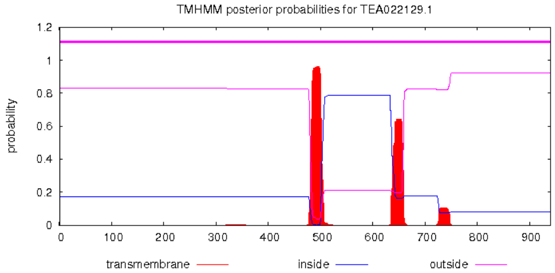


```
# TEA019143.1 Length: 724
# TEA019143.1 Number of predicted TMHs:  0
# TEA019143.1 Exp number of AAs in TMHs: 0.01226
# TEA019143.1 Exp number, first 60 AAs:  0
# TEA019143.1 Total prob of N-in:        0.00005
TEA019143.1     TMHMM2.0        outside      1   724
```

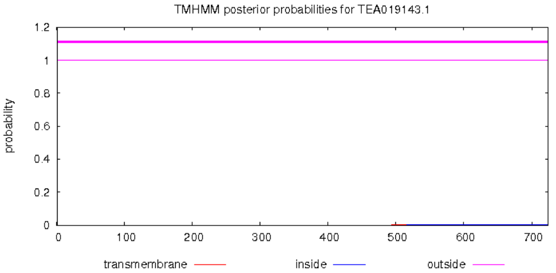


```
# TEA028452.1 Length: 846
# TEA028452.1 Number of predicted TMHs:  0
# TEA028452.1 Exp number of AAs in TMHs: 0.05099
# TEA028452.1 Exp number, first 60 AAs:  0
# TEA028452.1 Total prob of N-in:        0.00001
TEA028452.1     TMHMM2.0        outside      1   846
```

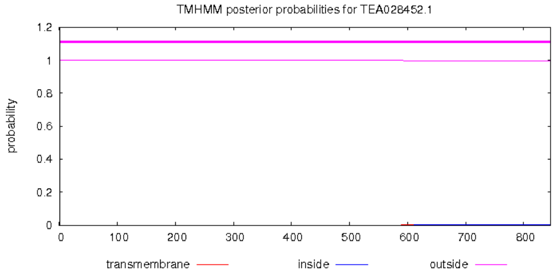


```
# TEA016969.1 Length: 1107
# TEA016969.1 Number of predicted TMHs:  0
# TEA016969.1 Exp number of AAs in TMHs: 6.33735
# TEA016969.1 Exp number, first 60 AAs:  0
# TEA016969.1 Total prob of N-in:        0.27806
TEA016969.1     TMHMM2.0        outside      1  1107
```

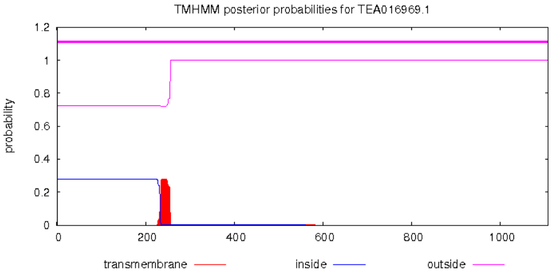

```
# TEA013270.1 Length: 755
# TEA013270.1 Number of predicted TMHs:  0
# TEA013270.1 Exp number of AAs in TMHs: 0.10844
# TEA013270.1 Exp number, first 60 AAs:  0
# TEA013270.1 Total prob of N-in:        0.00501
TEA013270.1     TMHMM2.0        outside      1   755
```

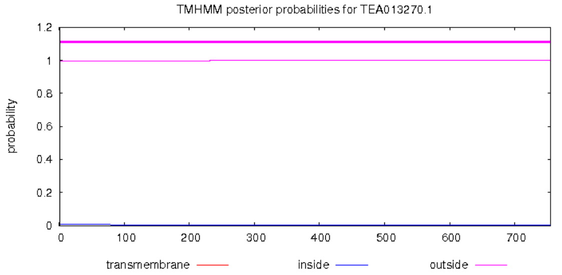


```
# TEA026716.1 Length: 368
# TEA026716.1 Number of predicted TMHs:  0
# TEA026716.1 Exp number of AAs in TMHs: 0.00933
# TEA026716.1 Exp number, first 60 AAs:  0.00015
# TEA026716.1 Total prob of N-in:        0.02057
TEA026716.1     TMHMM2.0        outside      1   368
```

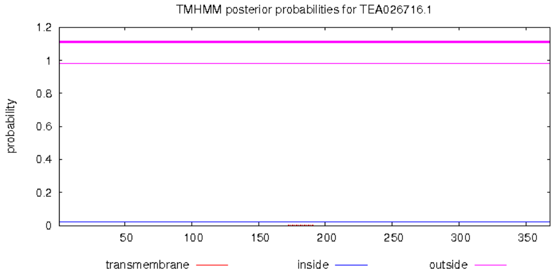


```
# TEA028758.1 Length: 1213
# TEA028758.1 Number of predicted TMHs:  0
# TEA028758.1 Exp number of AAs in TMHs: 0.0185
# TEA028758.1 Exp number, first 60 AAs:  0.00018
# TEA028758.1 Total prob of N-in:        0.00001
TEA028758.1     TMHMM2.0        outside      1  1213
```

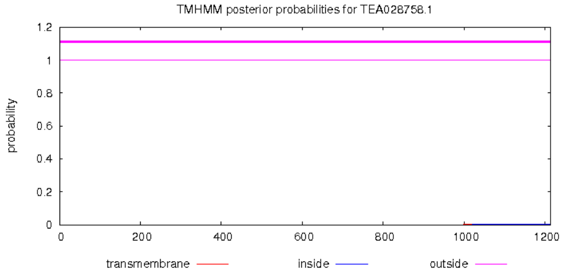


```
# TEA010804.1 Length: 305
# TEA010804.1 Number of predicted TMHs:  0
# TEA010804.1 Exp number of AAs in TMHs: 0.00132
# TEA010804.1 Exp number, first 60 AAs:  0
# TEA010804.1 Total prob of N-in:        0.01241
TEA010804.1     TMHMM2.0        outside      1   305
```

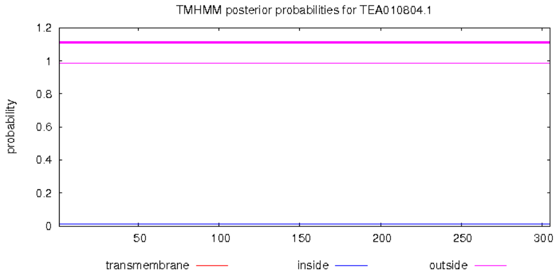


```
# TEA009451.1 Length: 1333
# TEA009451.1 Number of predicted TMHs:  0
# TEA009451.1 Exp number of AAs in TMHs: 0.02716
# TEA009451.1 Exp number, first 60 AAs:  0.02022
# TEA009451.1 Total prob of N-in:        0.00132
TEA009451.1     TMHMM2.0        outside      1  1333
```

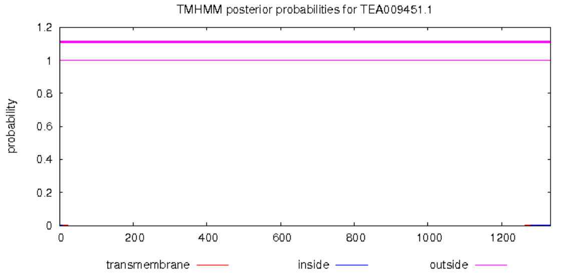


```
# TEA021421.1 Length: 1331
# TEA021421.1 Number of predicted TMHs:  0
# TEA021421.1 Exp number of AAs in TMHs: 0.01119
# TEA021421.1 Exp number, first 60 AAs:  0
# TEA021421.1 Total prob of N-in:        0.00001
TEA021421.1     TMHMM2.0        outside      1  1331
```

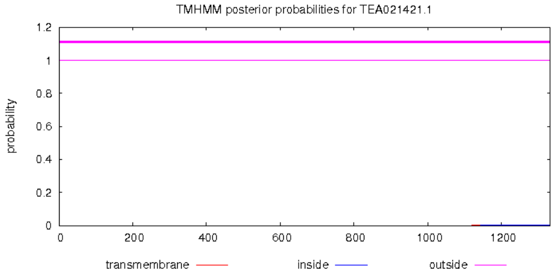


```
# TEA017670.1 Length: 561
# TEA017670.1 Number of predicted TMHs:  0
# TEA017670.1 Exp number of AAs in TMHs: 0.03517
# TEA017670.1 Exp number, first 60 AAs:  0
# TEA017670.1 Total prob of N-in:        0.00270
TEA017670.1     TMHMM2.0        outside      1   561
```

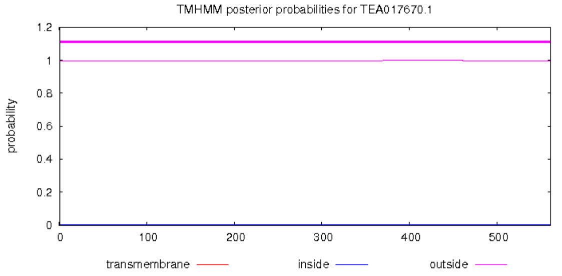


```
# TEA019184.1 Length: 601
# TEA019184.1 Number of predicted TMHs:  0
# TEA019184.1 Exp number of AAs in TMHs: 0.01866
# TEA019184.1 Exp number, first 60 AAs:  0
# TEA019184.1 Total prob of N-in:        0.00147
TEA019184.1     TMHMM2.0        outside      1   601
```

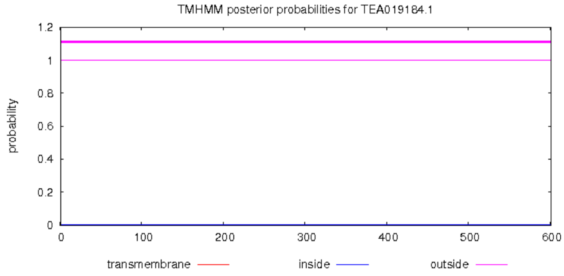

```
# TEA000933.1 Length: 407
# TEA000933.1 Number of predicted TMHs:  0
# TEA000933.1 Exp number of AAs in TMHs: 0.46154
# TEA000933.1 Exp number, first 60 AAs:  0
# TEA000933.1 Total prob of N-in:        0.00794
TEA000933.1     TMHMM2.0        outside      1   407
```

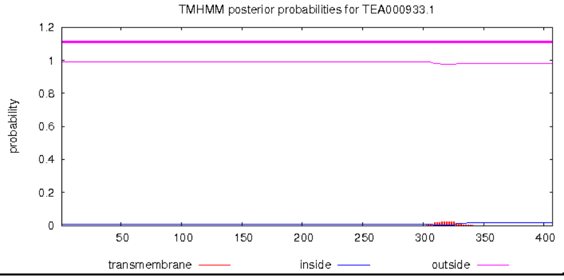


```
# TEA031230.1 Length: 489
# TEA031230.1 Number of predicted TMHs:  0
# TEA031230.1 Exp number of AAs in TMHs: 0.13166
# TEA031230.1 Exp number, first 60 AAs:  0.00013
# TEA031230.1 Total prob of N-in:        0.00470
TEA031230.1     TMHMM2.0        outside      1   489
```

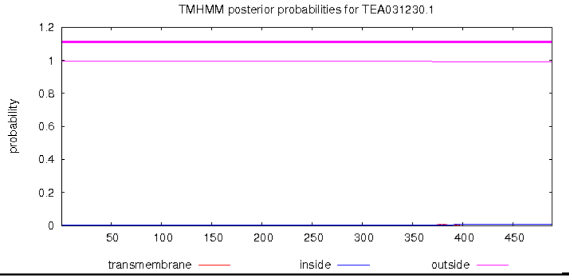


```
# TEA022171.1 Length: 404
# TEA022171.1 Number of predicted TMHs:  0
# TEA022171.1 Exp number of AAs in TMHs: 0.01418
# TEA022171.1 Exp number, first 60 AAs:  0
# TEA022171.1 Total prob of N-in:        0.01930
TEA022171.1     TMHMM2.0        outside      1   404
```

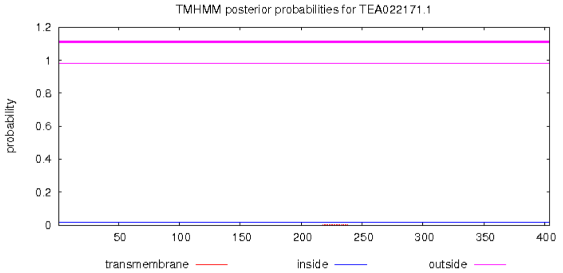


```
# TEA011280.1 Length: 368
# TEA011280.1 Number of predicted TMHs:  0
# TEA011280.1 Exp number of AAs in TMHs: 0.0076
# TEA011280.1 Exp number, first 60 AAs:  0
# TEA011280.1 Total prob of N-in:        0.00291
TEA011280.1     TMHMM2.0        outside      1   368
```

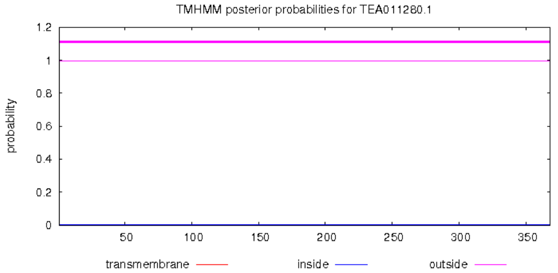


```
# TEA031223.1 Length: 434
# TEA031223.1 Number of predicted TMHs:  0
# TEA031223.1 Exp number of AAs in TMHs: 0.27035
# TEA031223.1 Exp number, first 60 AAs:  0
# TEA031223.1 Total prob of N-in:        0.01318
TEA031223.1     TMHMM2.0        outside      1   434
```

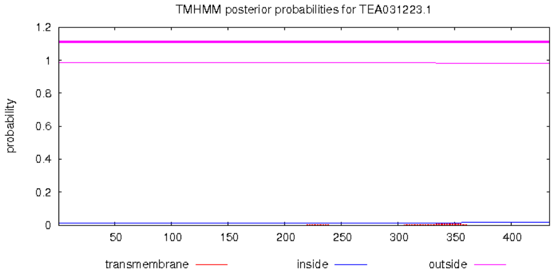


```
# TEA007232.1 Length: 368
# TEA007232.1 Number of predicted TMHs:  0
# TEA007232.1 Exp number of AAs in TMHs: 0.04776
# TEA007232.1 Exp number, first 60 AAs:  0
# TEA007232.1 Total prob of N-in:        0.01435
TEA007232.1     TMHMM2.0        outside      1   368
```

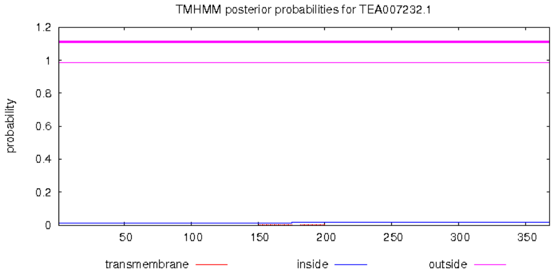


```
# TEA016553.1 Length: 432
# TEA016553.1 Number of predicted TMHs:  0
# TEA016553.1 Exp number of AAs in TMHs: 0.01056
# TEA016553.1 Exp number, first 60 AAs:  0
# TEA016553.1 Total prob of N-in:        0.00632
TEA016553.1     TMHMM2.0        outside      1   432
```

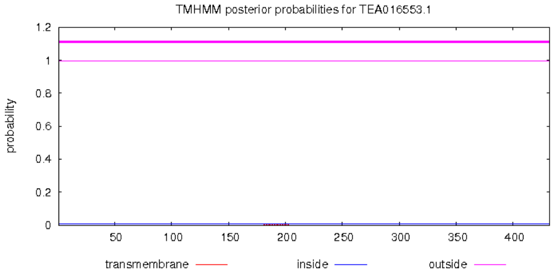


```
# TEA033032.1 Length: 415
# TEA033032.1 Number of predicted TMHs:  0
# TEA033032.1 Exp number of AAs in TMHs: 0.16877
# TEA033032.1 Exp number, first 60 AAs:  8e-05
# TEA033032.1 Total prob of N-in:        0.00767
TEA033032.1     TMHMM2.0        outside      1   415
```

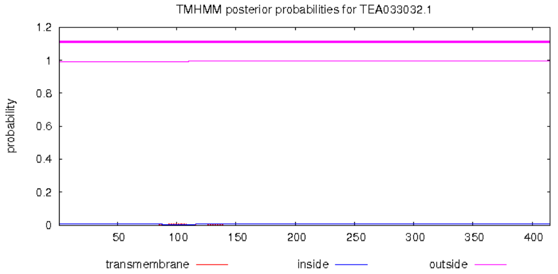

```
# TEA001764.1 Length: 351
# TEA001764.1 Number of predicted TMHs:  0
# TEA001764.1 Exp number of AAs in TMHs: 0.22163
# TEA001764.1 Exp number, first 60 AAs:  0.00043
# TEA001764.1 Total prob of N-in:        0.01088
TEA001764.1     TMHMM2.0        outside      1   351
```

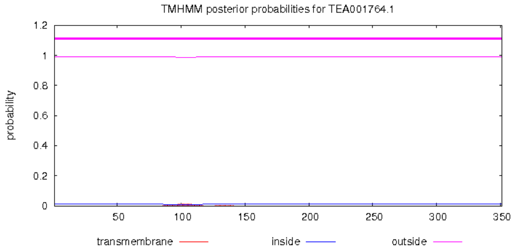


```
# TEA026000.1 Length: 1296
# TEA026000.1 Number of predicted TMHs:  0
# TEA026000.1 Exp number of AAs in TMHs: 0.3705
# TEA026000.1 Exp number, first 60 AAs:  0
# TEA026000.1 Total prob of N-in:        0.00000
TEA026000.1     TMHMM2.0        outside      1  1296
```

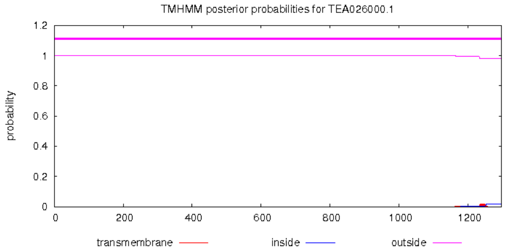


```
# TEA033556.1 Length: 541
# TEA033556.1 Number of predicted TMHs:  0
# TEA033556.1 Exp number of AAs in TMHs: 4.33922
# TEA033556.1 Exp number, first 60 AAs:  0.00021
# TEA033556.1 Total prob of N-in:        0.06460
TEA033556.1     TMHMM2.0        outside      1   541
```

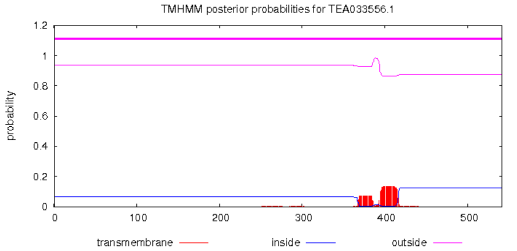


```
# TEA013875.1 Length: 341
# TEA013875.1 Number of predicted TMHs:  0
# TEA013875.1 Exp number of AAs in TMHs: 1.96914
# TEA013875.1 Exp number, first 60 AAs:  0.00196
# TEA013875.1 Total prob of N-in:        0.13897
TEA013875.1     TMHMM2.0        outside      1   341
```

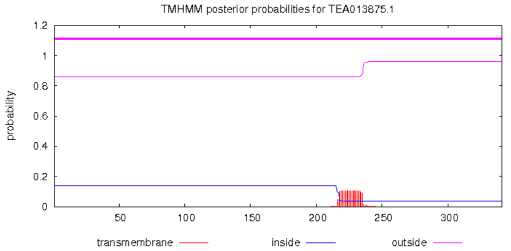


```
# TEA002722.1 Length: 1436
# TEA002722.1 Number of predicted TMHs:  0
# TEA002722.1 Exp number of AAs in TMHs: 0.01114
# TEA002722.1 Exp number, first 60 AAs:  6e-05
# TEA002722.1 Total prob of N-in:        0.00001
TEA002722.1     TMHMM2.0        outside      1  1436
```

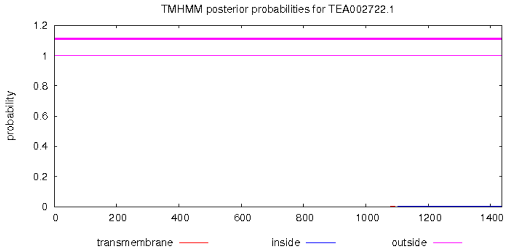


```
# TEA030052.1 Length: 1357
# TEA030052.1 Number of predicted TMHs:  0
# TEA030052.1 Exp number of AAs in TMHs: 0.02427
# TEA030052.1 Exp number, first 60 AAs:  0.00401
# TEA030052.1 Total prob of N-in:        0.00064
TEA030052.1     TMHMM2.0        outside      1  1357
```

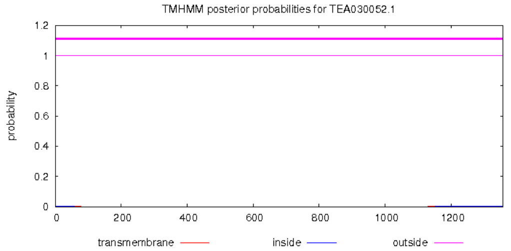


```
# TEA008343.1 Length: 334
# TEA008343.1 Number of predicted TMHs:  0
# TEA008343.1 Exp number of AAs in TMHs: 0.097979999
# TEA008343.1 Exp number, first 60 AAs:  0
# TEA008343.1 Total prob of N-in:        0.02590
TEA008343.1     TMHMM2.0        outside      1   334
```

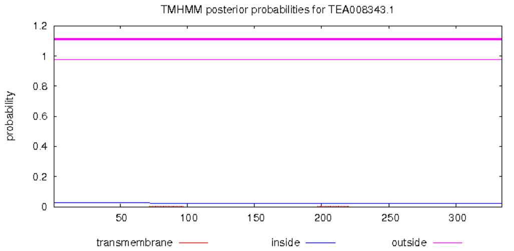

S2 Fig. Transmembrane helices of MAPKKKs of Raf subfamily in *C. sinensis*. TMHMM Server, v.2.0 (http://www.cbs.dtu.dk/services/TMHMM/), was used to predict the presence of transmembrane helices. The plot shows the posterior probabilities of inside/outside/TM helix. The plot is obtained by calculating the total probability that a residue sits in helix, inside, or outside summed over all possible paths through the model. The red peaks represent the possible transmembrane helices present. If the red peak crosses the transmembrane line (represented in pink colour), then its calculated as 1 transmembrane helix.

```
# TEA010125.1 Length: 675
# TEA010125.1 Number of predicted TMHs:  0
# TEA010125.1 Exp number of AAs in TMHs: 0.00052
# TEA010125.1 Exp number, first 60 AAs:  0
# TEA010125.1 Total prob of N-in:        0.00014
TEA010125.1     TMHMM2.0        outside      1   675
```

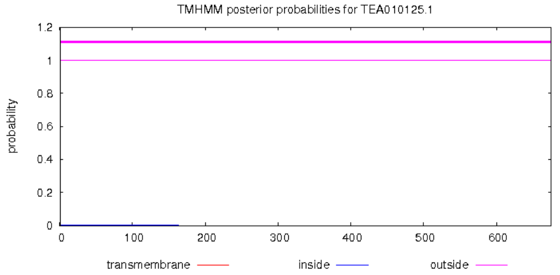


```
# TEA022762.1 Length: 732
# TEA022762.1 Number of predicted TMHs:  0
# TEA022762.1 Exp number of AAs in TMHs: 0.01389
# TEA022762.1 Exp number, first 60 AAs:  0.01054
# TEA022762.1 Total prob of N-in:        0.00094
TEA022762.1     TMHMM2.0        outside      1   732
```

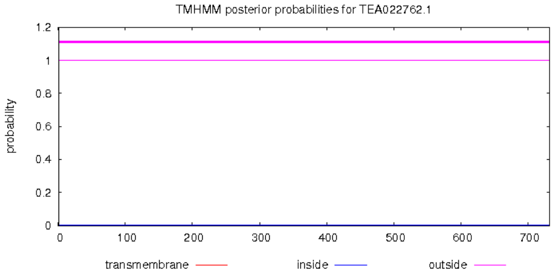


```
# TEA024720.1 Length: 655
# TEA024720.1 Number of predicted TMHs:  0
# TEA024720.1 Exp number of AAs in TMHs: 0.20568
# TEA024720.1 Exp number, first 60 AAs:  0.00353
# TEA024720.1 Total prob of N-in:        0.01086
TEA024720.1     TMHMM2.0        outside      1   655
```

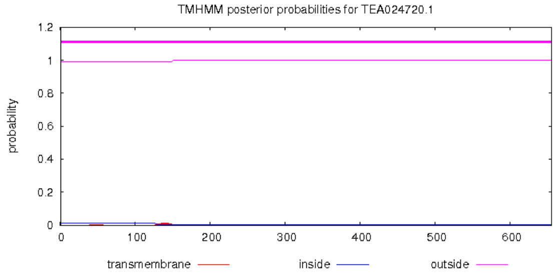


```
# TEA002087.1 Length: 719
# TEA002087.1 Number of predicted TMHs:  0
# TEA002087.1 Exp number of AAs in TMHs: 0.00067
# TEA002087.1 Exp number, first 60 AAs:  0
# TEA002087.1 Total prob of N-in:        0.00011
TEA002087.1     TMHMM2.0        outside      1   719
```

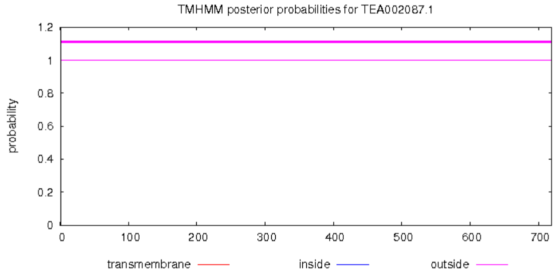


```
# TEA013346.1 Length: 831
# TEA013346.1 Number of predicted TMHs:  0
# TEA013346.1 Exp number of AAs in TMHs: 0.00018
# TEA013346.1 Exp number, first 60 AAs:  0
# TEA013346.1 Total prob of N-in:        0.00002
TEA013346.1     TMHMM2.0        outside      1   831
```

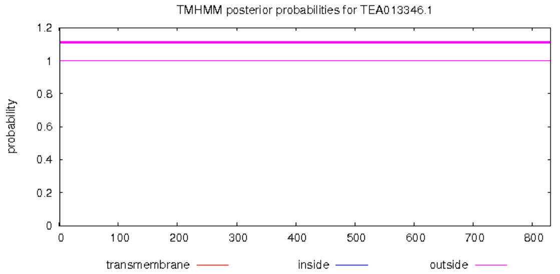


```
# TEA013344.1 Length: 481
# TEA013344.1 Number of predicted TMHs:  0
# TEA013344.1 Exp number of AAs in TMHs: 0.00164
# TEA013344.1 Exp number, first 60 AAs:  0
# TEA013344.1 Total prob of N-in:        0.00069
TEA013344.1     TMHMM2.0        outside      1   481
```

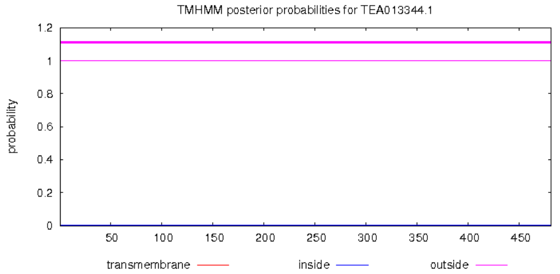


```
# TEA031068.1 Length: 762
# TEA031068.1 Number of predicted TMHs:  0
# TEA031068.1 Exp number of AAs in TMHs: 3.06785
# TEA031068.1 Exp number, first 60 AAs:  1.30494
# TEA031068.1 Total prob of N-in:        0.06089
TEA031068.1     TMHMM2.0        outside      1   762
```

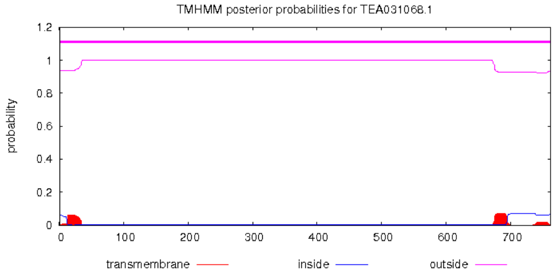


```
# TEA020698.1 Length: 664
# TEA020698.1 Number of predicted TMHs:  0
# TEA020698.1 Exp number of AAs in TMHs: 1.53983
# TEA020698.1 Exp number, first 60 AAs:  0.00118
# TEA020698.1 Total prob of N-in:        0.00060
TEA020698.1     TMHMM2.0        outside      1   664
```

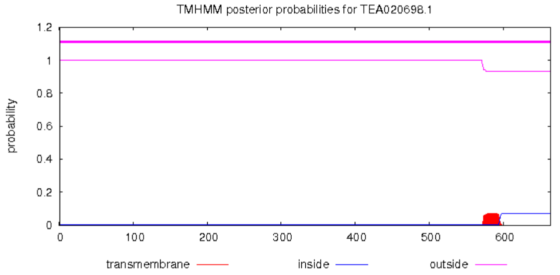

```
# TEA027328.1 Length: 748
# TEA027328.1 Number of predicted TMHs:  1
# TEA027328.1 Exp number of AAs in TMHs: 30.33445
# TEA027328.1 Exp number, first 60 AAs:  29.76268
# TEA027328.1 Total prob of N-in:        0.47196
# TEA027328.1 POSSIBLE N-term signal sequence
TEA027328.1     TMHMM2.0        inside       1    39
TEA027328.1     TMHMM2.0        TMhelix     40    58
TEA027328.1     TMHMM2.0        outside     59   748
```

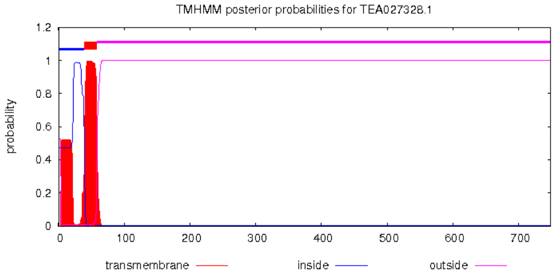


```
# TEA020112.1 Length: 300
# TEA020112.1 Number of predicted TMHs:  0
# TEA020112.1 Exp number of AAs in TMHs: 0.00508
# TEA020112.1 Exp number, first 60 AAs:  0
# TEA020112.1 Total prob of N-in:        0.01462
TEA020112.1     TMHMM2.0        outside      1   300
```

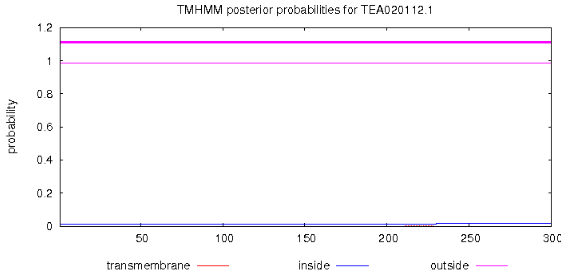


```
# TEA033250.1 Length: 622
# TEA033250.1 Number of predicted TMHs:  0
# TEA033250.1 Exp number of AAs in TMHs: 0.00119
# TEA033250.1 Exp number, first 60 AAs:  0
# TEA033250.1 Total prob of N-in:        0.00024
TEA033250.1     TMHMM2.0        outside      1   622
```

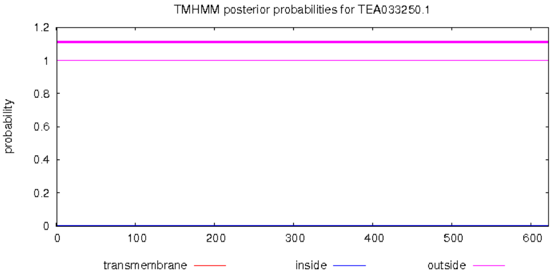

S3 Fig. Transmembrane helices of MAPKKKs of ZIK subfamily in *C. sinensis*. TMHMM Server, v.2.0 (http://www.cbs.dtu.dk/services/TMHMM/), was used to predict the presence of transmembrane helices. The plot shows the posterior probabilities of inside/outside/TM helix. The plot is obtained by calculating the total probability that a residue sits in helix, inside, or outside summed over all possible paths through the model. The red peaks represent the possible transmembrane helices present. If the red peak crosses the transmembrane line (represented in pink colour), then its calculated as 1 transmembrane helix.

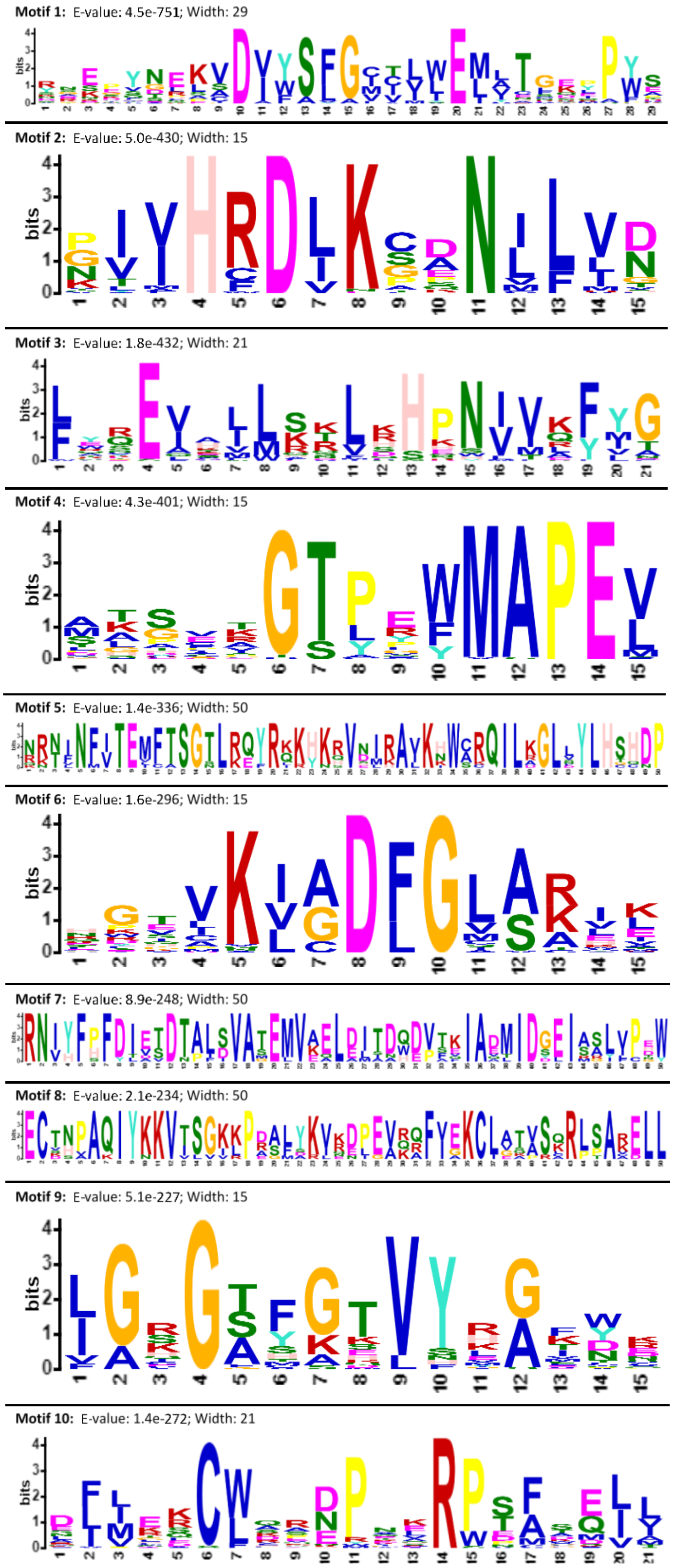

S4 Fig. Motif logos of the 10 identified motifs in 59 MAPKKKs of *C. sinensis*. The motif logos were generated by MEME suite.

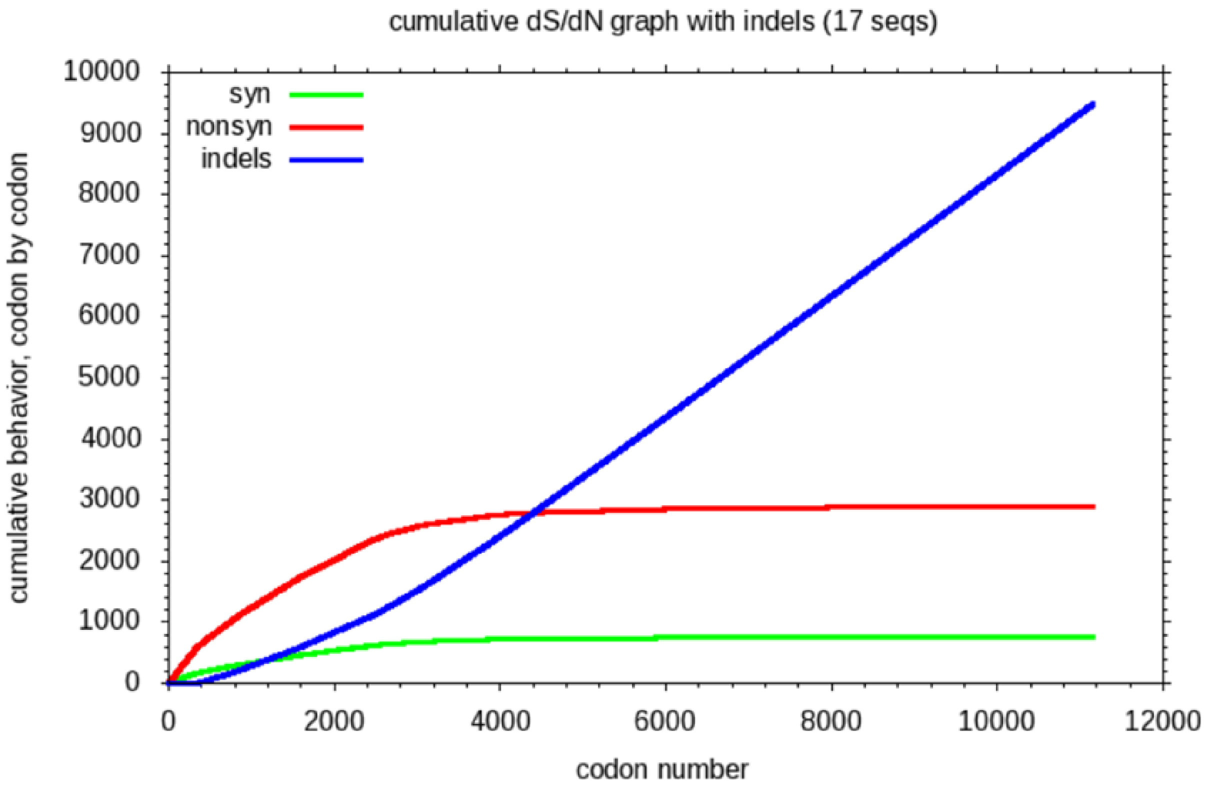

S5 Fig. The ds/dn cumulative graph of MAPKKKs of MEKK subfamily in *C. sinensis*. SNAP server (https://www.hiv.lanl.gov/content/sequence/SNAP/SNAP.html) has been used to generate the graph.

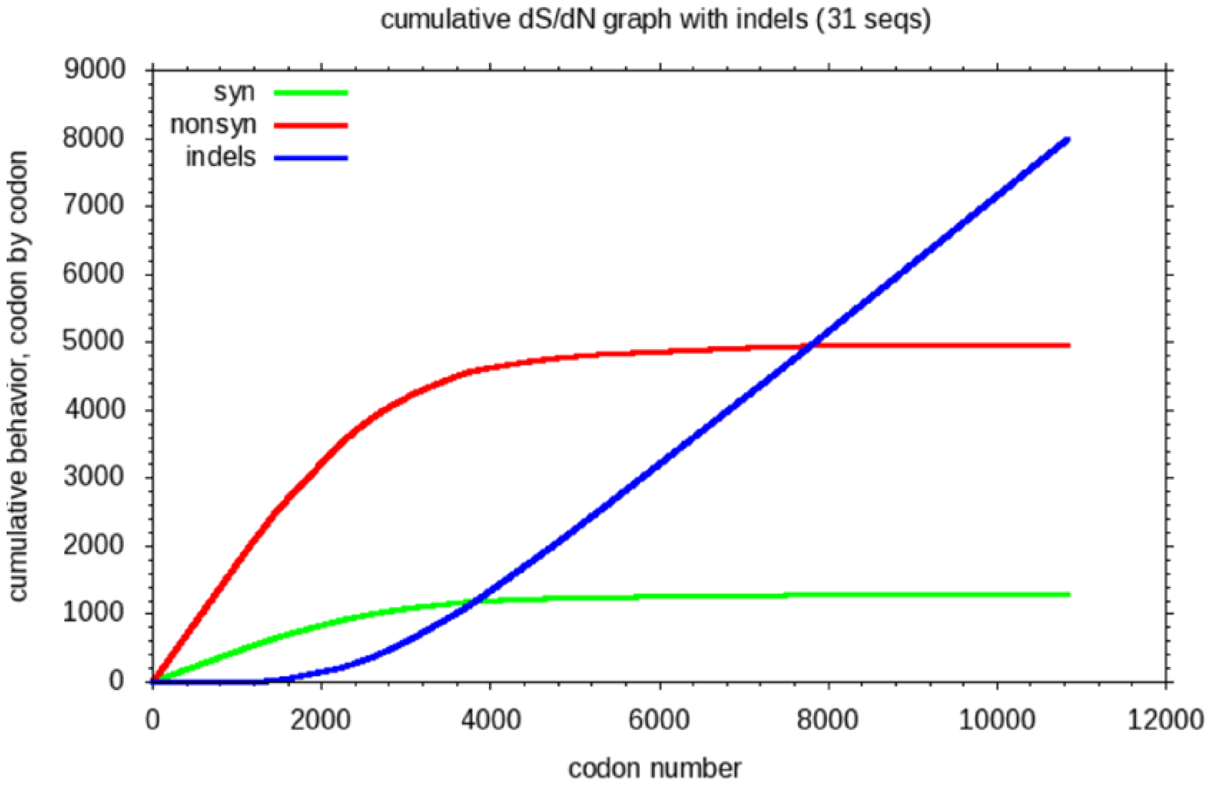

S6 Fig. The ds/dn cumulative graph of MAPKKKs of Raf subfamily in *C. sinensis*. SNAP server (https://www.hiv.lanl.gov/content/sequence/SNAP/SNAP.html) has been used to generate the graph.

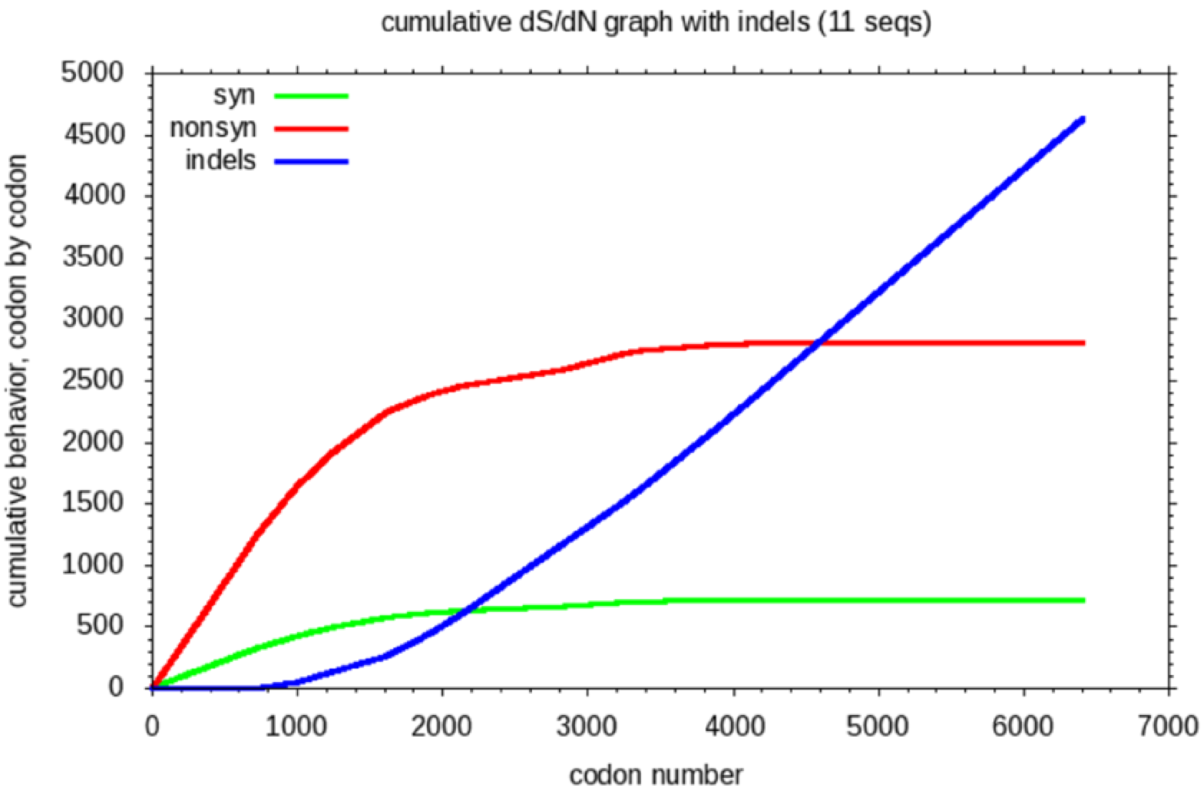

S7 Fig. The ds/dn cumulative graph of MAPKKKs of ZIK subfamily in *C. sinensis*. SNAP server (https://www.hiv.lanl.gov/content/sequence/SNAP/SNAP.html) has been used to generate the graph.

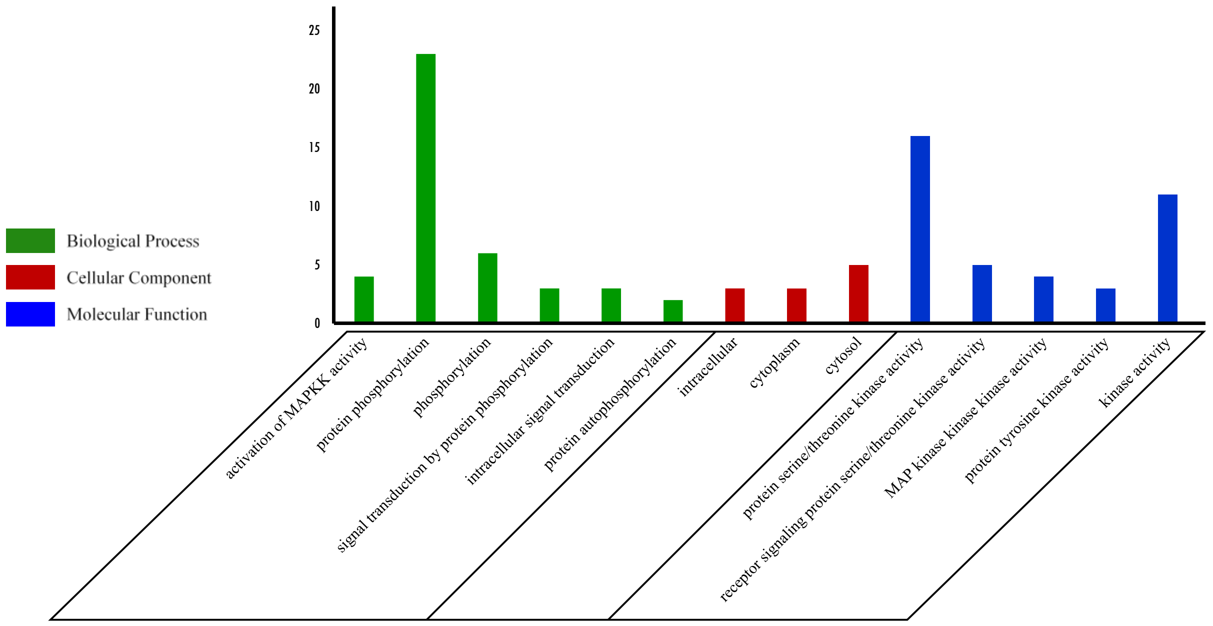

S8 Fig. GO analysis of all the 59 MAPKKKs in *C. sinensis*. The results have been grouped into three main categories: Biological Process, Cellular Component and Molecular function. The y-axis represents the frequency of genes while the x-axis represents the putative functions.

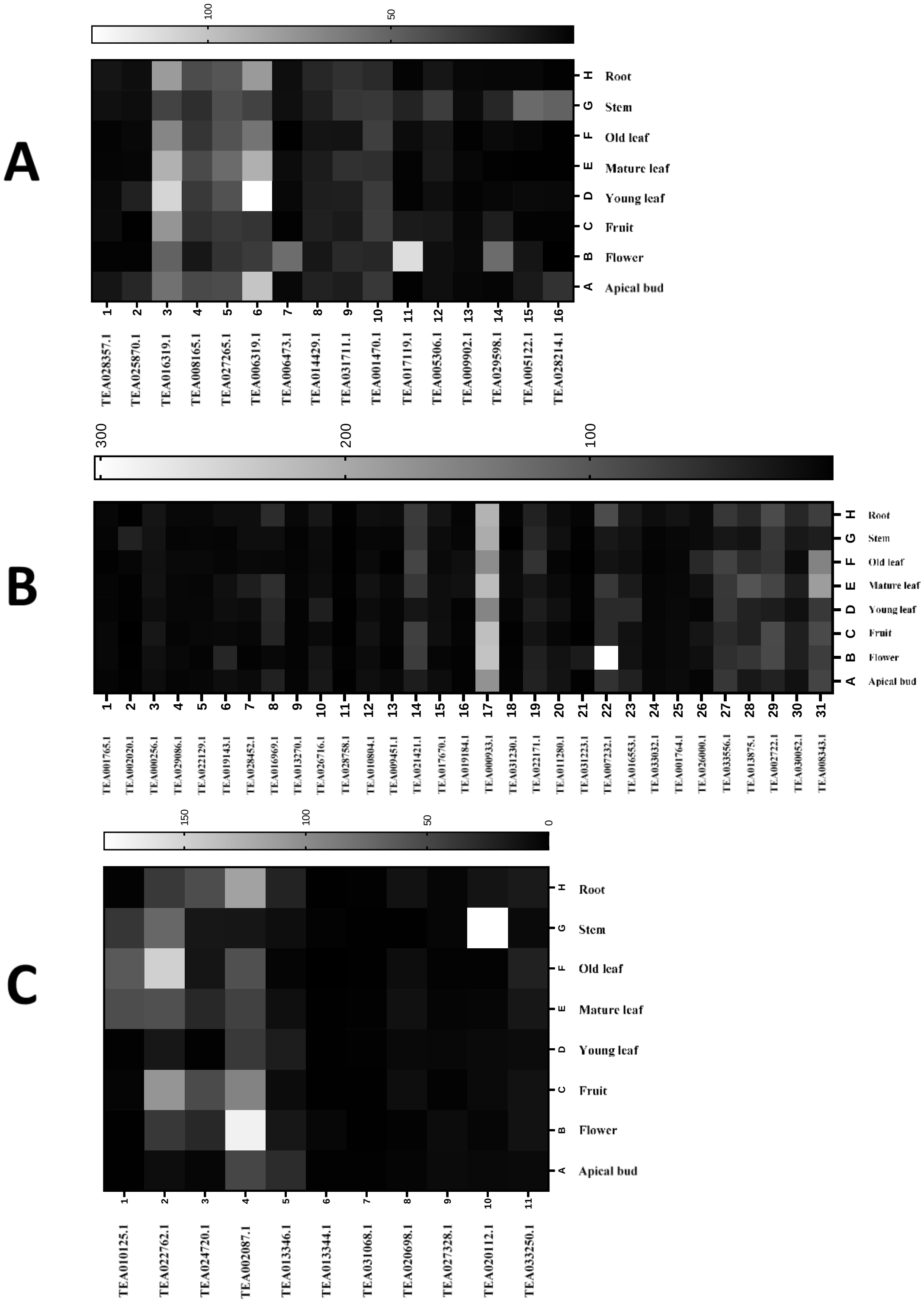

S9 Fig. Heat maps for tissue-specific expression patterns of A) MEKK; B) Raf and; C) ZIK genes in *C. sinensis*. The relative expression of these genes were analysed in different tissues, by using GraphPad Prism 8 software. The level of expression was in transcript per million (TPM). The 8 different tissues are represented on the right and the tea genes are marked below.

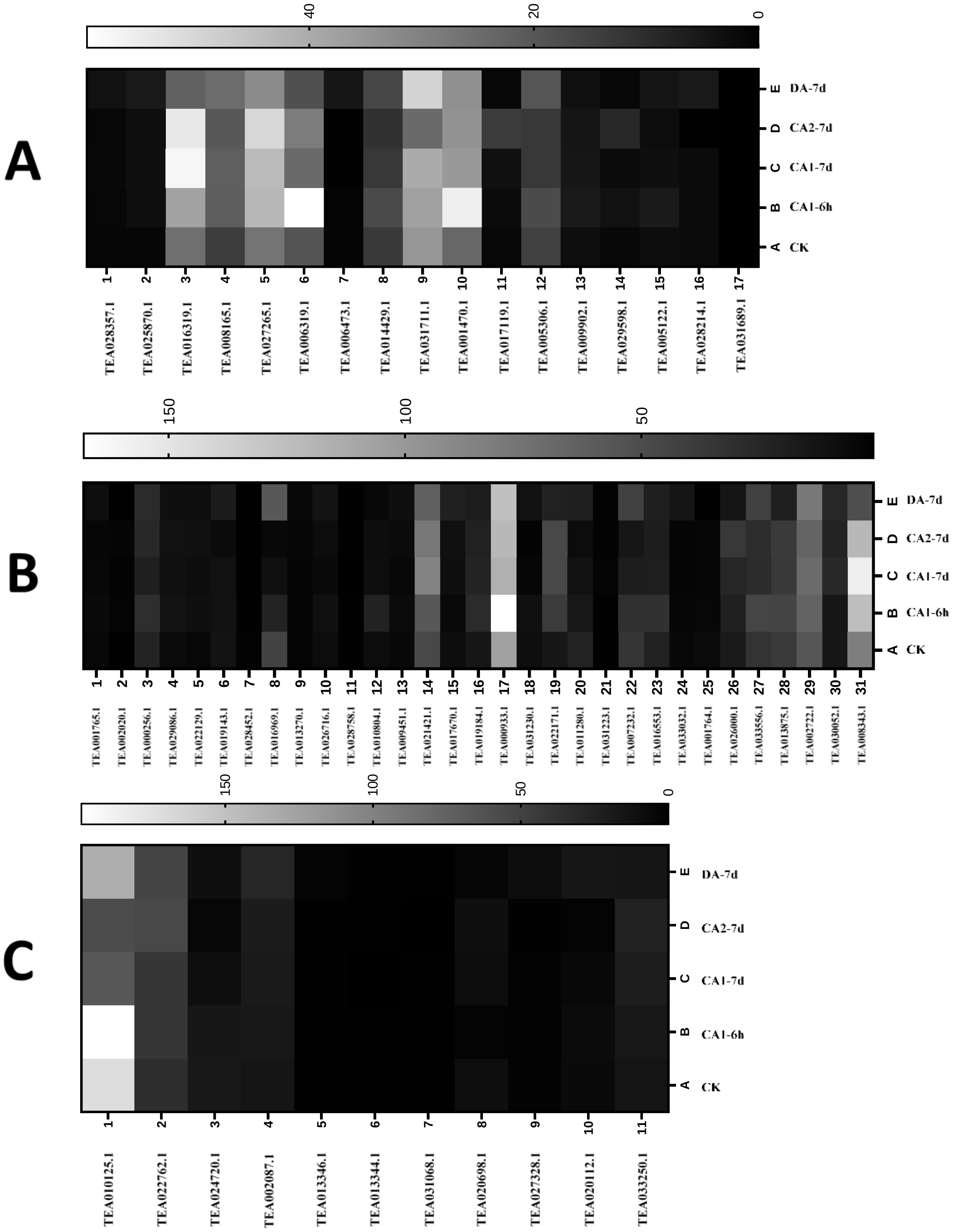

S10 Fig. Heat maps for cold stress expression patterns of A) MEKK; B) Raf and; C) ZIK genes in *C. sinensis*. The relative expression of these genes were analysed in different stages, by using GraphPad Prism 8 software. The level of expression was in transcript per million (TPM). The different stages are represented on the right and the tea genes are marked below.

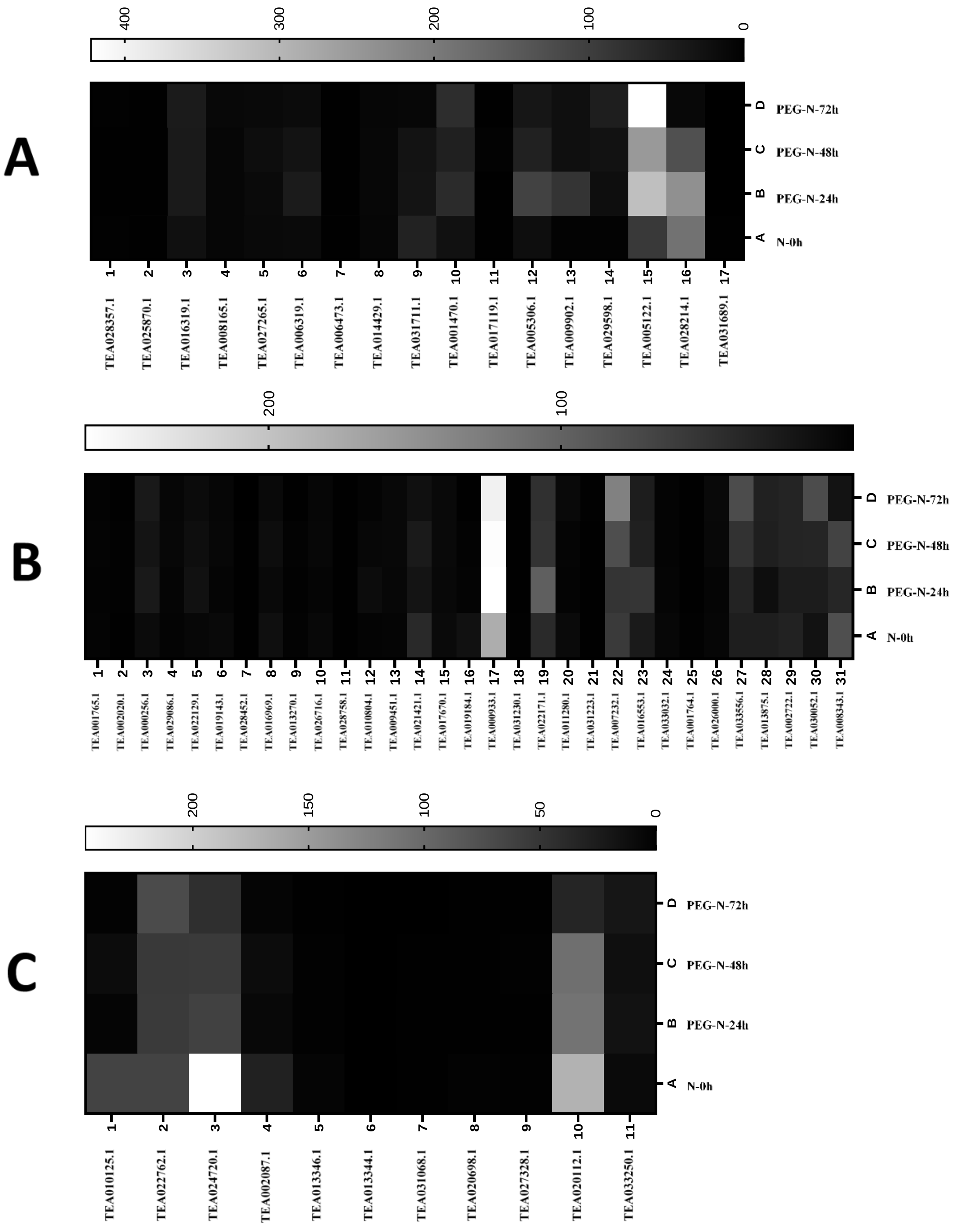

S11 Fig. Heat maps for drought stress expression patterns of A) MEKK; B) Raf and; C) ZIK genes in *C. sinensis*. The relative expression of these genes were analysed in different stages, by using GraphPad Prism 8 software. The level of expression was in transcript per million (TPM). The different stages are represented on the right and the tea genes are marked below.

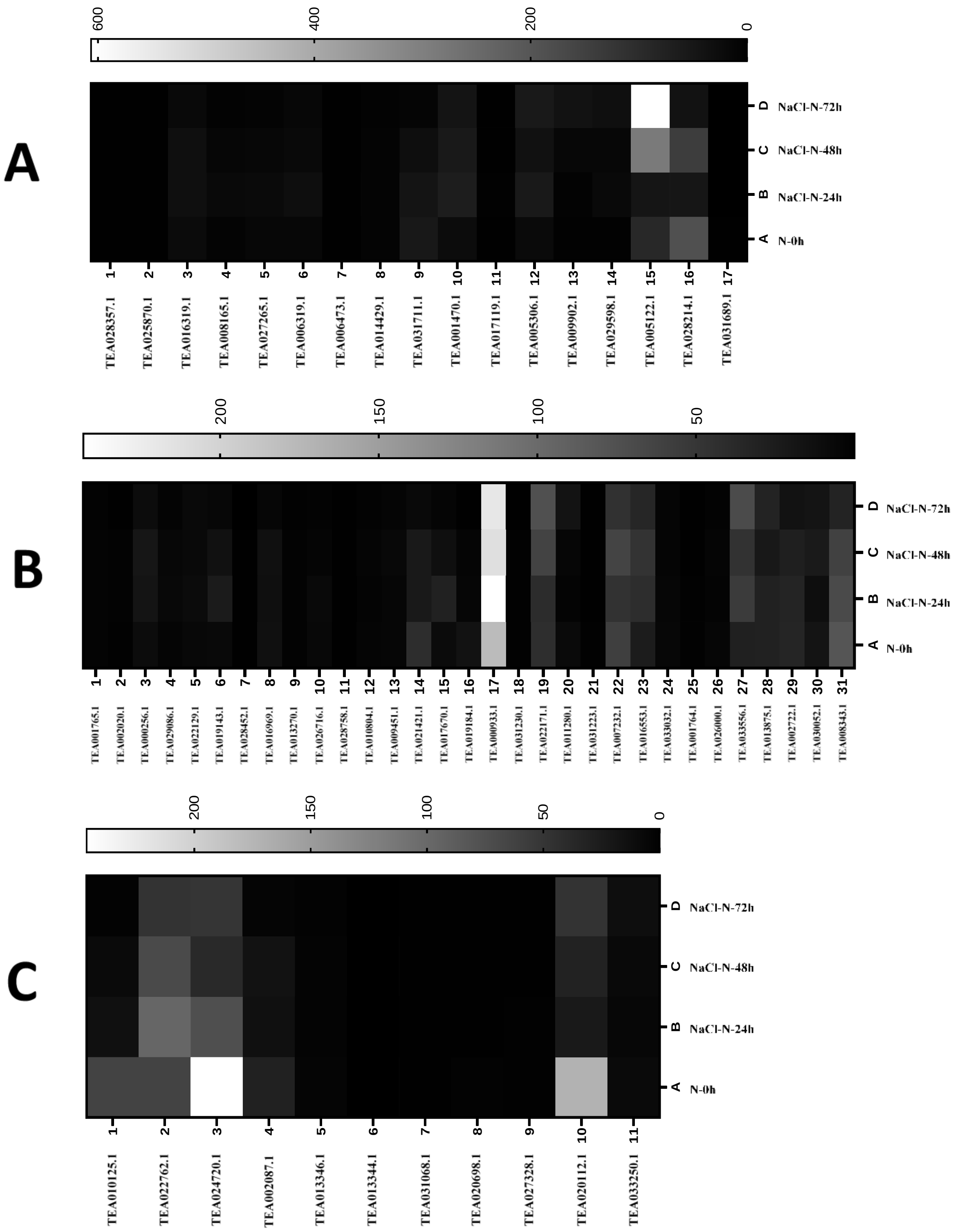

S12 Fig. Heat maps for salt stress expression patterns of (A) MEKK (B) Raf and (C) ZIK genes in *C. sinensis*. The relative expression of these genes were analysed in different stages, by using GraphPad Prism 8 software. The level of expression was in transcript per million (TPM). The different stages are represented on the right and the tea genes are marked below.

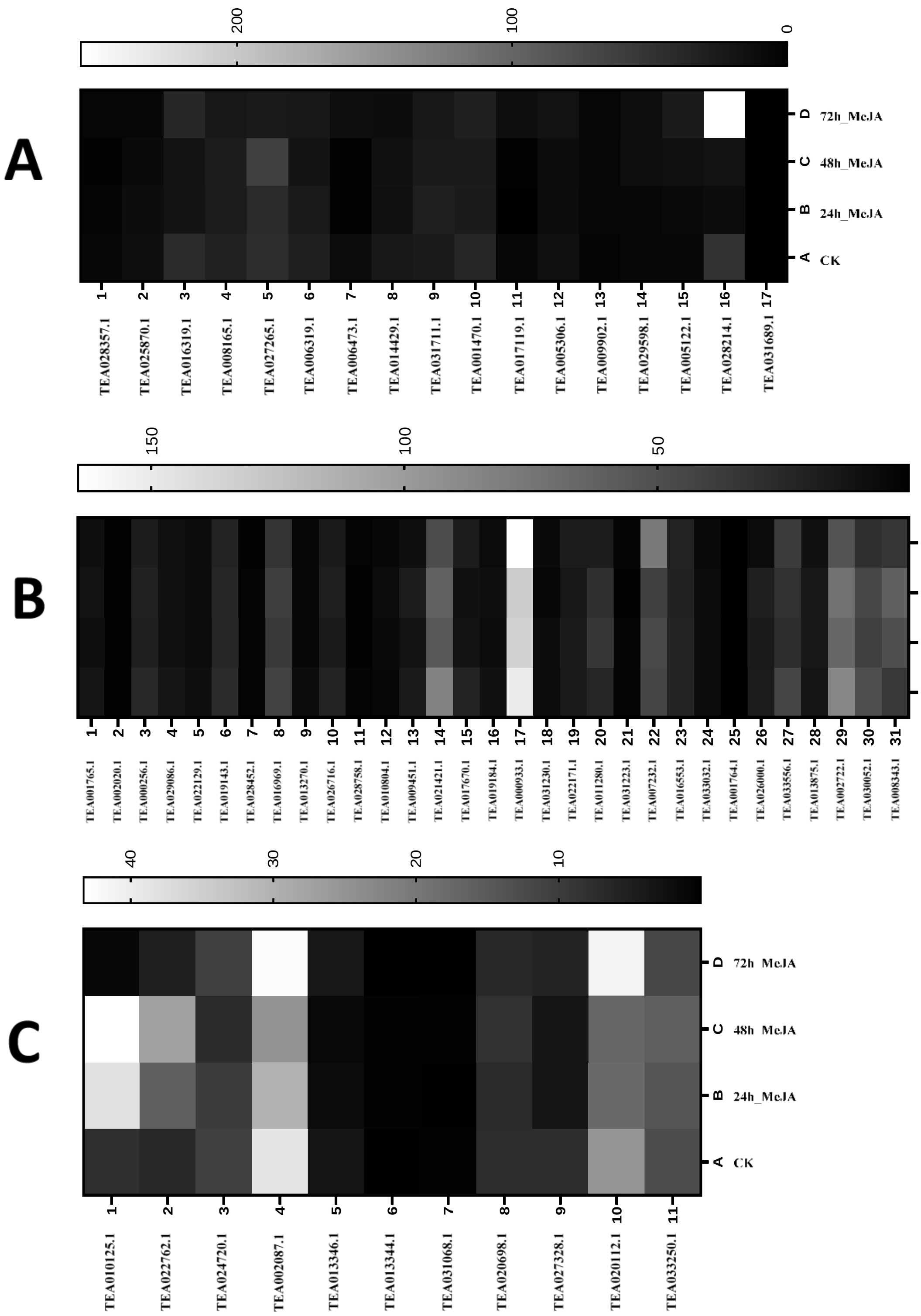

S13 Fig. Heat maps for MeJA treatment expression patterns of (A) MEKK (B) Raf and (C) ZIK genes in *C. sinensis*. The relative expression of these genes were analysed in different stages, by using GraphPad Prism 8 software. The level of expression was in transcript per million (TPM). The different stages are represented on the right and the tea genes are marked below.
