# Supplementary material for: In-silico genome wide analysis of Mitogen activated protein kinase kinase kinase gene family in C. sinensis
Source: PLoS One. 2021 Nov 4;16(11):e0258657. doi: 10.1371/journal.pone.0258657 (PMC8568164; doi:10.1371/journal.pone.0258657)
Supplement: Attachment — Submitted filename: PONE-D-21-05595_reviewer.pdf [file pone.0258657.s005.pdf]

PLOS ONE

In-silico identification, expressional profile and regulatory network analysis of Mitogen Activated Protein Kinase Kinase Kinase gene family in C. sinensis

--Manuscript Draft--

**Manuscript Number:**	PONE-D-21-05595
**Article Type:**	Research Article
**Full Title:**	In-silico identification, expressional profile and regulatory network analysis of Mitogen Activated Protein Kinase Kinase Kinase gene family in C. sinensis
**Short Title:**	MAPKKK gene family in C. sinensis
**Corresponding Author:**	Neelam Mishra<br>Saint Joseph's College: Saint Joseph's University<br>bengaluru, Karnataka INDIA
**Keywords:**	Keywords: Mitogen Activated Protein Kinase; Arabidopsis thaliana; Phylogenetic relationship; Functional interaction; Abiotic stress
**Abstract:**	Mitogen activated protein kinase kinase kinase (MAPKKK) form the upstream component of MAPK cascade. It is well characterized in several plants such as Arabidopsis and rice however the knowledge about MAPKKKs in tea plant is largely unknown. In the present study, MAPKKK genes of tea were obtained through a genome wide search using Arabidopsis thaliana as the reference genome. Among 59 candidate MAPKKK genes in tea, 17 genes were MEKK-like, 31 genes were Raf-like and 11 genes were ZIK- like. Additionally, phylogenetic relationships were established along with structural analysis which includes gene structure, its location as well as conserved motifs and functional domain signatures that were systematically examined and further, predictions were validated by the results. Also, on the basis of orthologous genes in Arabidopsis, functional interaction was carried out in C. sinensis. The expressional profiles indicated major involvement of MAPKKKK genes from tea in response to various abiotic stress factors. Taken together, this study provides the targets for additional inclusive identification, functional study, and also might provide comprehensive knowledge for a better understanding of the MAPKKK cascade regulatory network in C. sinensis .
**Order of Authors:**	Neelam Mishra
	Guoxin Shen
	Anurag srivastava
	shreya Subrahmanya
	Abhirup Paul
**Additional Information:**	
**Question**	**Response**
**Financial Disclosure**<br><br>Enter a financial disclosure statement that describes the sources of funding for the work included in this submission. Review the submission guidelines for detailed requirements. View published research articles from *PLOS ONE* for specific examples.<br><br>This statement is required for submission and **will appear in the published article** if the submission is accepted. Please make	This project was supported by the Key Technologies R & D Program for Crop Breeding of Zhejiang Province (2016C02054-19,2017C02010), the Natural Science Foundation of China (31670303), and the Joint Laboratory of Olive Oil Quality and Nutrition among China, Australia and Spain. The authors are thankful to DBT-eLibrary Consortium (DeLCON) for providing access to e-resources.

Powered by Editorial Manager® and ProduXion Manager® from Aries Systems Corporation

sure it is accurate.<br><br>**Unfunded studies**<br>Enter: *The author(s) received no specific funding for this work.*<br><br>**Funded studies**<br>Enter a statement with the following details:<br>• Initials of the authors who received each award<br>• Grant numbers awarded to each author<br>• The full name of each funder<br>• URL of each funder website<br>• Did the sponsors or funders play any role in the study design, data collection and analysis, decision to publish, or preparation of the manuscript?<br>• **NO** - Include this sentence at the end of your statement: *The funders had no role in study design, data collection and analysis, decision to publish, or preparation of the manuscript.*<br>• **YES** - Specify the role(s) played.<br><br>* typeset	
**Competing Interests**<br><br>Use the instructions below to enter a competing interest statement for this submission. On behalf of all authors, disclose any competing interests that could be perceived to bias this work—acknowledging all financial support and any other relevant financial or non-financial competing interests.<br><br>This statement is required for submission and **will appear in the published article** if the submission is accepted. Please make sure it is accurate and that any funding sources listed in your Funding Information later in the submission form are also declared in your Financial Disclosure statement.<br><br>View published research articles from *PLOS ONE* for specific examples.	The authors have declared that no competing interests exist.

**NO authors have competing interests**<br><br>Enter: *The authors have declared that no competing interests exist.*<br><br>**Authors with competing interests**<br><br>Enter competing interest details beginning with this statement:<br><br>*I have read the journal's policy and the authors of this manuscript have the following competing interests: [insert competing interests here]*<br><br>* typeset	
**Ethics Statement**<br><br>Enter an ethics statement for this submission. This statement is required if the study involved:<br><br>• Human participants<br>• Human specimens or tissue<br>• Vertebrate animals or cephalopods<br>• Vertebrate embryos or tissues<br>• Field research<br><br>Write "N/A" if the submission does not require an ethics statement.<br><br>General guidance is provided below. Consult the submission guidelines for detailed instructions. **Make sure that all information entered here is included in the Methods section of the manuscript.**	N/A

Powered by Editorial Manager® and ProduXion Manager® from Aries Systems Corporation

**Format for specific study types**<br><br>**Human Subject Research (involving human participants and/or tissue)**<br>• Give the name of the institutional review board or ethics committee that approved the study<br>• Include the approval number and/or a statement indicating approval of this research<br>• Indicate the form of consent obtained (written/oral) or the reason that consent was not obtained (e.g. the data were analyzed anonymously)<br><br>**Animal Research (involving vertebrate animals, embryos or tissues)**<br>• Provide the name of the Institutional Animal Care and Use Committee (IACUC) or other relevant ethics board that reviewed the study protocol, and indicate whether they approved this research or granted a formal waiver of ethical approval<br>• Include an approval number if one was obtained<br>• If the study involved *non-human primates*, add *additional details* about animal welfare and steps taken to ameliorate suffering<br>• If anesthesia, euthanasia, or any kind of animal sacrifice is part of the study, include briefly which substances and/or methods were applied<br><br>**Field Research**<br><br>Include the following details if this study involves the collection of plant, animal, or other materials from a natural setting:<br>• Field permit number<br>• Name of the institution or relevant body that granted permission	
**Data Availability**<br><br>Authors are required to make all data underlying the findings described fully available, without restriction, and from the time of publication. PLOS allows rare exceptions to address legal and ethical concerns. See the PLOS Data Policy and FAQ for detailed information.	Yes - all data are fully available without restriction

A Data Availability Statement describing where the data can be found is required at submission. Your answers to this question constitute the Data Availability Statement and **will be published in the article**, if accepted.<br><br>**Important:** Stating 'data available on request from the author' is not sufficient. If your data are only available upon request, select 'No' for the first question and explain your exceptional situation in the text box.<br><br>Do the authors confirm that all data underlying the findings described in their manuscript are fully available without restriction?	
**Describe where the data may be found in full sentences. If you are copying our sample text, replace any instances of XXX with the appropriate details.**<br><br>• If the data are **held or will be held in a public repository**, include URLs, accession numbers or DOIs. If this information will only be available after acceptance, indicate this by ticking the box below. For example: *All XXX files are available from the XXX database (accession number(s) XXX, XXX.).*<br>• If the data are all contained **within the manuscript and/or Supporting Information files**, enter the following: *All relevant data are within the manuscript and its Supporting Information files.*<br>• If neither of these applies but you are able to provide **details of access elsewhere**, with or without limitations, please do so. For example:<br><br>*Data cannot be shared publicly because of [XXX]. Data are available from the XXX Institutional Data Access / Ethics Committee (contact via XXX) for researchers who meet the criteria for access to confidential data.*<br><br>*The data underlying the results presented in the study are available from (include the name of the third party*	All relevant data are within the manuscript and its Supporting Information files.

and contact information or URL).<br>• This text is appropriate if the data are owned by a third party and authors do not have permission to share the data.<br><br>* typeset	
Additional data availability information:	

Powered by Editorial Manager® and ProduXion Manager® from Aries Systems Corporation

Manuscript

Click here to access/download;Manuscript;Paul et al._TEA_MAPKKK_Manuscript.docx

In-silico identification, expressional profile and regulatory network analysis of Mitogen Activated Protein Kinase Kinase Kinase gene family in *C. sinensis*

Abhirup Paul[1†], Anurag P. Srivastava[2†], Shreya Subrahmanya[3], Guoxin Shen[4†*], Neelam Mishra[3*]

[1]Department of Biochemistry

REVA University

Bangalore, Karnataka,

India

[2]Department of life Sciences

Garden City University

Bangalore, Karnataka,

India

[3]Department of Botany

St. Joseph's College autonomous

Bangalore, Karnataka,

India

[4]Sericultural Research Institute,

Zhejiang Academy of Agricultural Sciences

Hangzhou 310021, China

[†]These authors contributed equally to this work.

[*]Corresponding authors:

Guoxin Shen, Ph.D., Professor, Tel: +86-571-86404298; Fax: +86-571-86404298

Email address: guoxin.shen@ttu.edu

Neelam Mishra, Ph.D., Assistant professor

Email address: neelamiitkgp@gmail.com; neelammishra@sjc.ac.in

Orcid id:

1. Abhirup Paul: 0000-0003-2143-7511
2. Neelam Mishra: 0000-0001-6191-5392

Abstract

Mitogen activated protein kinase kinase kinase (MAPKKK) form the upstream component of MAPK cascade. It is well characterized in several plants such as Arabidopsis and rice however the knowledge about MAPKKKs in tea plant is largely unknown. In the present study, MAPKKK genes of tea were obtained through a genome wide search using *Arabidopsis thaliana* as the reference genome. Among 59 candidate MAPKKK genes in tea, 17 genes were MEKK-like, 31 genes were Raf-like and 11 genes were ZIK- like. Additionally, phylogenetic relationships were established along with structural analysis which includes gene structure, its location as well as conserved motifs and functional domain signatures that were systematically examined and further, predictions were validated by the results. Also, on the basis of orthologous genes in Arabidopsis, functional interaction was carried out in *C. sinensis.* The expressional profiles indicated major involvement of MAPKKKK genes from tea in response to various abiotic stress factors. Taken together, this study provides the targets for additional inclusive identification, functional study, and also ~~might~~ provide comprehensive knowledge for a better understanding of the MAPKKK cascade regulatory network in *C. sinensis*.

Keywords: Mitogen Activated Protein Kinase; *Arabidopsis thaliana*; Phylogenetic relationship; Functional interaction; Abiotic stress

Introduction

Mitogen-activated protein kinase (MAPK) cascades are universal signal transduction modules existing in eukaryotes, including yeasts, animals and plants. MAPKKKs, which form the upstream component of three tier kinase module are usually activated by G- proteins but sometimes activation is also done via an upstream MAP4K [1]. MAPKKKs are the first component of this phosphorelay cascade, which phosphorylates two serine/threonine residues in a conserved S/T-X_{3-5}-S/T motif of the MKK activation loop. Then, MKKs are dual-specificity kinases that activate the downstream MAPK through TDY or TEY phosphorylation motif in the activation loop (T-loop) [2, 3]. The activated MAPK ultimately phosphorylates various downstream substrates, including transcription factors and other signalling components that regulate the expression of downstream genes [4]. They form the largest group among MAPK cascade, with 80 members in Arabidopsis, 75 members in rice, 74 members in maize and 89 members in tomato [5,6]. This largest group is further subdivided into three smaller groups on the basis of sequence similarities 1) MEKK family 2) Raf family 3) ZIK family [6,7]. Compared to MAPKs and MAPKKs, the MAPKKKs have more members and greater variety in primary structures and domain composition [8]. Phylogenetic analysis of the MAPKKK genes in various species shows that the diversity exists in plants. Among the MAPKKKs, the Raf subfamily is the largest group and comprises of 46 members from maize, 43 from rice, 27 from grapevines, and 48 from Arabidopsis. It is followed by the MEKK family which is the second largest family and comprises of 22 members from maize, 22 in rice, 9 in grapevine, and 21 in Arabidopsis. The ZIK family is the smallest among the three subfamilies and comprises of 6 members from maize, 10 from rice, 9 from grapevines, and 11 from Arabidopsis [5, 6, 9]. The MEKK subfamily comprises a conserved kinase domain of G(T/S)Px(W/Y/F)MAPEV [5]. The ZIK subfamily contains TPEFMAPE(L/V)Y while the Raf subfamily has GTxx(W/Y)MAPE [5]. All the MAPKKK

proteins have a kinase domain, and most of them have a serine/threonine protein kinase active site [10]. Structural domains of MAPKKKs in Arabidopsis, rice and cucumber show that most Raf proteins have a C-terminal kinase domain and a long N-terminal regulatory domain. In contrast, members of the ZIK group have the N-terminal kinase domain, whilst members of the MEKK group have less conserved kinase domain which consists of either N- or C-terminals or lie in the central part of the protein [6, 9, 11]. MAPKKKs play a significant role in distinct biological and physiological processes, and they have potential applications for the development of stress-tolerant transgenic plants. Two of the best studied Arabidopsis MAPKKKs are EDR1 (Enhanced disease resistance) and CTR1 (Constitutive triple response) are known to participate in defense responses and ethylene signalling respectively [2, 12, 13].

C. sinensis, more commonly known as tea is the second most important beverage in the world besides water. Tea plant is an important commercial crop potentially rich in variety of bioactive ingredients. Although many stress physiology studies have been carried out in tea plant however, the roles of MAPKKK genes in tea plant have not been studied in detail. In the present study, the MAPKKK family of genes was thoroughly defined on the basis of *in-silico* genome-wide search in tea using *Arabidopsis thaliana* as the reference genome. Gene locations on scaffolds, their structures and their evolutionary aspect were systematically studied. Further, we analyzed the interaction networks of proteins based on orthologous genes in Arabidopsis. This study might provide more insights on functional analysis and also highlight the MAPK signalling cascade-mediated pathway of *C. sinensis* and beyond.

Materials and methods

Identification of MAPKKK gene family in Tea

The predicted peptide sequences of tea were downloaded from the Tea Plant Information Archive (TPIA) database (http://tpia.teaplant.org/) [14]. To identify tea MAPKKK genes, 415 previously known MAPKKK genes were retrieved from *Arabidopsis thaliana* (80), *Oryza sativa* (75), *Solanum lycopersicum* (71), *Solanum tuberosum* (81), *Capsicum annum* (60) and *Coffee canephora* (48) using TAIR database (https://www.arabidopsis.org/) [15] Rice Genome Annotation Project database (http://rice.plantbiology.msu.edu/) [16] and Sol Genomics Network database (https://solgenomics.net/) [17]. The retrieved Arabidopsis and rice MAPKKK sequences were used as query sequences to search against the tea plant genome database deploying the BLASTp algorithm with an e value set to 1e-5 and identity percentage of 50% as threshold. The obtained genes were then aligned and uploaded to SMART (http://smart.embl-heidelberg.de/) [18] and Pfam web tool (https://pfam.xfam.org/) to confirm the existence of kinase domains. Self-BLAST of the identified sequences were done to remove any chances of redundancy and were considered potential *C. sinensis* MAPKKK genes. The physicochemical properties of the identified tea MAPKKK genes were predicted using ProtParam tool incorporated in ExPASy database (https://expasy.org/) [19]. Subcellular localization of the peptides was predicted using the BaCelLo (Balanced subcellular localization predictor) online server (http://gpcr.biocomp.unibo.it/bacello/index.htm) [20]. Furthermore, TMHMM server v2.0 (http://www.cbs.dtu.dk/services/TMHMM/) [21] was employed to predict the presence of trans-membrane helices in tea MAPKKK peptide sequences.

Estimation of K_a/K_s ratios

K_a and K_s ratios were ~~also~~ calculated using the SNAP v.2.1.1 online tool (https://www.hiv.lanl.gov/content/sequence/SNAP/SNAP.html) [22] to assess the

synonymous and non-synonymous groups. The dN/dS values ~~resemble~~ the selective pressure of duplicate genes and the dS values represent the time of divergence of duplication events.

Multiple sequence alignment and Phylogeny analysis

The tea MAPKKK protein sequences were subjected to multiple sequence alignment, using CLUSTALW (https://www.ebi.ac.uk/Tools/msa/clustalo/) [23] to check for conserved MAPKKK specific domains for each subfamily. Phylogenetic analysis were done separately for MEKK, Raf and ZIK sub-families, using the identified tea sequences, coupled with Arabidopsis, rice, tomato, potato, capsicum and coffee peptide sequences ~~respectively~~. The phylogenetic trees were constructed by the Neighbor-Joining algorithm of MEGA 7.0.14 [24] keeping all the parameters at default values. The consistencies of the obtained trees were assessed by the bootstrap method and replicate was set to 1000. Furthermore, the network of functionally interacting orthologous genes between tea and Arabidopsis was identified and constructed using STRING online tool ((https://string-db.org/) [25] with default parameters.

Intron exon structures and conserved motifs

The intron exon distribution pattern for tea MEKK, Raf and ZIK peptide sequences were analysed and visualised using the Gene Structure Display Server v2.0 (http://gsds.cbi.pku.edu.cn/) [26]. The full-length peptide sequences were uploaded to MEME suite (http://meme-suite.org/) [27] in-order to identify the conserved motifs.

Mapping of tea MAPKKK genes onto scaffolds and gene duplication

TPIA database has incomplete genome assembly information. As a result, the tea MAPKKK genes were mapped onto their respective scaffolds using MapGene2chromosome web v2 (MG2C) software tool (http://mg2c.iask.in/mg2c_v2.0/) [28]. The genes were mapped according to their scaffold positional information available in TPIA database, which includes

scaffold IDs for each gene, scaffold dimensions and the starting and ending position of the each gene on the scaffolds.

Expression profiles of tea MAPKKK genes

The tissue specific expression profiles, which include expression levels in apical bud, flower, fruit, young leaf, mature leaf, old leaf, root, and stem were retrieved from TPIA database. Furthermore, the abiotic stress tolerance data (cold, drought, salt) under different parameters, were retrieved from TPIA database along with the expression data for treatment with methyl jasmonates (MeJA). This data was fed into GraphPad Prism 8 (https://www.graphpad.com/scientific-software/prism/) to generate the respective graphs for MEKK, Raf and ZIK sub-families.

Results

Identification of MAPKKK gene family in *C. sinensis*

In order to identify the MAPKKK gene family in tea (*C. sinensis*), 415 known MAPKKK peptide sequences from *Arabidopsis thaliana* (80), *Oryza sativa* (75), *Solanum lycopersicum* (71), *Solanum tuberosum* (81), *Capsicum annum* (60) and *Coffee canephora* (48) were retrieved from their respective databases. MAPKKK gene family is divided into three ~~other~~ sub-families, which include MEKK-like, Raf-like and ZIK-like genes. To ~~classify~~ and categorize the MAPKKK genes in tea, BLASTp searches were conducted against the tea protein database, using the retrieved peptide sequences from Arabidopsis and rice as query sequences. For all BLASTp searches, e value and identity percentage were set to 1e-5 and 50% as threshold, respectively (Supplementary Table S1, Supplementary Table S2 and Supplementary Table S3). The identified tea peptides were again screened with a Hidden Markov Model (HMM) search to confirm the presence of serine/threonine-protein kinase-like domain (PF00069). The screened peptides were again subjected to self-BLAST to remove

any chances of redundant data. The final screened tea MAPKKK genes yielded 59 total potential genes, which included 17 MEKK-like, 31 Raf-like and 11 ZIK-like genes and were incorporated into the final dataset.

The physicochemical properties of the identified tea MAPKKK protein sequences were evaluated using ExPASy ProtParam tool (Table 1, Table 2 and Table 3). The length and molecular weight of the 17 MEKK proteins ranged from 311 to 1191 amino acid residues and 34828.88 to 130956.46 kDa respectively (Table 1). For the Raf proteins, it ranged from 305 to 1436 amino acid residues and 35012.57 to 159263.21 kDa (Table 2), and for the ZIK proteins, it ranged from 300 to 831 amino acid residues and 34181.96 to 94422.51 kDa (Table 3). The theoretical pI values ranged from 4.58 to 9.50 for MEKK, 4.88 to 9.61 for Raf and 5.14 to 6.33 for ZIK proteins, indicating that most of the MEKK and Raf proteins have a basic nature while the ZIK proteins being acidic. The grand average of hydropathy (GRAVY index) in all the extracted MEKK, Raf and ZIK were negative values, ranging from -0.605 to -0.060, -0.661 to -0.182 and -0.582 to -0.350 respectively. This indicates that all the identified 59 tea MAPKKKs are hydrophilic in nature. 52 of the 59 putative tea MAPKKKs had instability index values above 40, while 6 Raf genes (TEA000933.1, TEA022171.1, TEA011280.1, TEA031223.1, TEA007232.1 and TEA013875.1) and 1 ZIK gene (TEA020112.1) had instability index values less than 40 (Table 1, Table 2 and Table 3). This signifies the unstable nature of most of the identified tea MAPKKKs ~~[29]~~. Subcellular localization of the peptides was predicted using the BaCelLo online server with 49 genes being localized in the nucleus, 9 genes in chloroplast and 2 genes in cytoplasm (Table 1, Table 2, and Table 3). TMHMM server v2.0 was employed to predict the presence of trans-membrane helices in the putative peptide sequences and one of the ZIK genes (TEA027328.1) had one trans-membrane helix (Supplementary Fig. S1, Supplementary Fig. S2, Supplementary Fig. S3).

Table 1: Sequence characteristics and physicochemical properties of MAPKKKs belonging to MEKK subfamily in *C. sinensis*. Locus position, gene length, protein length, molecular weight and pI value, no. of negative and positive residues, GRAVY index, instability index, aliphatic index and subcellular localizations were analysed.

Gene ID	Locus position	Gene length (bp)	Protein length (aa)	Mol. Wt. (kDa)	pI value	No. of negative residues	No. of positive residues	GRAVY index	Instability index	Aliphatic index	Subcellular localization
TEA028357.1	Scaffold856:196999-204246-	7247	628	68667.76	5.60	77	67	-0.380	58.36	76.85	Nucleus
TEA025870.1	Scaffold790:521648-539960+	18312	776	85271.15	6.76	94	92	-0.379	45.58	81.08	Nucleus
TEA016319.1	Scaffold3144:371539-383072-	11533	627	68238.67	9.50	53	71	-0.535	50.61	68.23	Nucleus
TEA008165.1	Scaffold3102:729210-737275+	8065	1032	112285.36	9.04	84	102	-0.423	53.62	72.95	Nucleus
TEA027265.1	Scaffold1289:966535-975893+	9358	939	101539.85	9.35	80	104	-0.605	63.34	65.88	Nucleus
TEA006319.1	Scaffold2905:735285-744378+	9093	683	75479.57	9.32	62	78	-0.505	67.84	72.55	Chloroplast
TEA006473.1	Scaffold1374:1527992-1535696-	7704	710	78857.59	9.09	65	79	-0.516	69.53	71.59	Nucleus
TEA014429.1	Scaffold41:2381991-2415462+	33471	1191	130956.46	6.13	145	128	-0.350	45.47	89.93	Chloroplast
TEA031711.1	Scaffold5399:986467-998883-	12416	562	62129.83	6.31	72	69	-0.484	48.47	75.62	Nucleus
TEA001470.1	Scaffold558:920549-933450+	12901	789	87423.22	8.34	90	95	-0.313	49.68	84.13	Nucleus
TEA017119.1	Scaffold5354:234291-239017-	4726	506	56190.19	4.66	80	49	-0.481	47.12	69.53	Nucleus
TEA005306.1	Scaffold2184:2097399-2125258+	27859	1097	121164.56	5.40	162	127	-0.540	49.78	72.63	Nucleus
TEA009902.1	Scaffold438:521469-522821-	1352	450	49874.37	4.58	65	34	-0.060	44.64	91.60	Chloroplast
TEA029598.1	Scaffold944:301732-304329+	2597	423	46235.51	4.94	62	43	-0.433	51.54	74.18	Nucleus
TEA005122.1	Scaffold1857:297670-298674-	1004	334	36588.08	6.01	40	34	-0.381	46.55	78.23	Chloroplast
TEA028214.1	Scaffold613:628014-629048+	1034	344	38088.50	6.33	44	41	-0.322	45.18	79.36	Nucleus
TEA031689.1	Scaffold1549:309791-310726-	935	311	34828.88	6.04	44	40	-0.340	48.20	90.64	Nucleus

Table 2: Sequence characteristics and physicochemical properties of MAPKKKs belonging to Raf subfamily in *C. sinensis*. Locus position, gene length, protein length, molecular weight and pI value, no. of negative and positive residues, GRAVY index, instability index, aliphatic

Gene ID	Locus position	Gene length (bp)	Protein length (aa)	Mol. Wt. (kDa)	pI value	No. of negative residues	No. of positive residues	GRAVY index	Instability index	Aliphatic index	Subcellular localization
TEA001765.1	Scaffold1670:382409-407933-	25524	842	93193.15	5.86	107	92	-0.248	46.33	89.69	Nucleus
TEA002020.1	Scaffold3595:726640-735244+	8604	896	99135.31	6.37	111	103	-0.382	42.96	81.80	Nucleus
TEA000256.1	Scaffold3876:193108-215389+	22281	1086	119081.90	6.63	118	112	-0.441	44.80	78.36	Nucleus
TEA029086.1	Scaffold106:745738-778269+	32531	919	101696.51	5.17	119	85	-0.182	41.82	91.44	Chloroplast
TEA022129.1	Scaffold3036:784237-806418+	22181	940	104852.31	6.01	114	102	-0.217	48.52	89.81	Nucleus
TEA019143.1	Scaffold1695:623368-630213+	6845	724	79987.28	7.68	86	87	-0.609	41.33	70.98	Nucleus

index and subcellular localizations were analysed.

Gene ID	Locus position	Gene length (bp)	Protein length (aa)	Mol. Wt. (kDa)	pI value	No. of negative residues	No. of positive residues	GRAVY index	Instability index	Aliphatic index	Subcellular localization
TEA028452.1	Scaffold433:2415340-2426547+	11207	846	93141.05	6.10	107	93	-0.523	46.31	70.89	Nucleus
TEA016969.1	Scaffold4925:453439-477111+	23672	1107	124661.25	8.46	145	153	-0.506	46.58	77.06	Nucleus
TEA013270.1	Scaffold344:585774-608400+	22626	755	85320.07	5.83	104	84	-0.374	53.97	80.97	Nucleus
TEA026716.1	Scaffold1930:511463-522712-	11249	368	41783.40	5.63	52	44	-0.487	46.26	73.89	Nucleus
TEA028758.1	Scaffold9739:380569-387825-	7256	1213	135047.30	5.63	159	123	-0.661	51.62	66.13	Nucleus
TEA010804.1	Scaffold35:1009695-1024064+	14369	305	35012.57	6.50	44	41	-0.644	44.62	74.16	Nucleus
TEA017670.1	Scaffold1963:483241-501001+	13760	561	62970.57	5.67	78	62	-0.383	49.24	89.63	Nucleus
TEA010125.1	Scaffold52:664232-675917-	11685	675	76706.84	5.67	91	68	-0.546	47.51	72.79	Nucleus
TEA019184.1	Scaffold4191:163416-174412+	10996	601	67890.36	5.90	76	65	-0.328	49.47	89.02	Nucleus
TEA022762.1	Scaffold9600:223976-236388+	12412	732	83655.02	5.87	103	83	-0.419	50.69	89.07	Nucleus
TEA000933.1	Scaffold1397:63694-76812+	13118	407	45660.50	7.66	45	46	-0.291	38.27	81.89	Nucleus
TEA024720.1	Scaffold1050:31289-41391+	10102	695	74571.17	6.33	89	81	-0.518	51.00	77.68	Nucleus
TEA002087.1	Scaffold754:205321-211058-	5737	719	81783.51	5.54	108	80	-0.582	42.80	75.41	Nucleus
TEA031230.1	Scaffold2248:916305-925818+	9313	489	54633.73	9.20	56	67	-0.311	43.48	87.32	Chloroplast
TEA013346.1	Scaffold5883:262251-272009+	9758	831	94422.51	5.53	124	93	-0.504	43.60	79.06	Nucleus
TEA022171.1	Scaffold382:2039496-2046332+	6836	404	44640.23	8.60	48	54	-0.379	26.52	78.89	Nucleus
TEA013344.1	Scaffold5883:191507-194485+	2978	481	55933.38	5.65	72	58	-0.472	40.70	80.04	Nucleus
TEA011280.1	Scaffold3804:571784-578194+	6410	368	41126.17	7.52	48	49	-0.312	25.58	82.91	Nucleus
TEA031068.1	Scaffold1571:837990-857219+	19229	762	86343.00	5.92	98	76	-0.375	40.49	85.07	Chloroplast
TEA031223.1	Scaffold2248:107085-111327-	4242	434	48234.42	6.42	57	55	-0.280	26.84	84.22	Nucleus
TEA020698.1	Scaffold2762:535605-540535-	4930	664	75716.56	5.63	95	74	-0.439	46.00	81.91	Nucleus
TEA007232.1	Scaffold3038:2387807-2395630-	7823	368	41118.03	7.02	48	48	-0.424	35.65	77.34	Nucleus
TEA016553.1	Scaffold1761:1968012-1984037-	16025	432	49062.23	8.45	65	69	-0.513	43.90	83.06	Nucleus
TEA033032.1	Scaffold858:331961-346154-	14193	415	46688.59	6.05	59	52	-0.355	43.89	86.53	Nucleus
TEA001764.1	Scaffold619:1545624-1550286+	4662	351	39474.64	6.47	42	39	-0.191	43.90	86.72	Cytoplasm
TEA026000.1	Scaffold3457:1062923-1073461-	10538	1296	144765.39	5.40	177	122	-0.507	42.22	73.72	Nucleus
TEA033556.1	Scaffold192:400250-403690-	3440	541	61612.16	9.27	57	72	-0.371	46.58	86.19	Chloroplast
TEA013875.1	Scaffold5449:126808-131150+	4342	341	39047.30	6.76	44	42	-0.256	36.12	91.52	Cytoplasm
TEA002722.1	Scaffold1369:145416-156759+	11343	1436	159263.21	5.41	203	153	-0.566	45.20	72.12	Nucleus
TEA030052.1	Scaffold319:1148438-1156900+	8462	1357	148194.52	5.00	166	110	-0.444	50.17	73.96	Nucleus
TEA008343.1	Scaffold142:344598-352450+	7852	334	37992.05	9.61	35	46	-0.257	46.78	86.17	Nucleus

Table 3: Sequence characteristics and physicochemical properties of MAPKKKs belonging to ZIK subfamily in *C. sinensis*. Locus position, gene length, protein length, molecular weight and pI value, no. of negative and positive residues, GRAVY index, instability index, aliphatic index and subcellular localizations were analysed.

TEA027328.1	Scaffold688:688353-693133+	4780	748	84782.46	5.48	100	81	-0.350	42.68	80.16	Chloroplast
TEA020112.1	Scaffold1093:624579-626760+	2181	300	34181.96	5.60	47	40	-0.428	33.33	85.47	Nucleus
TEA033250.1	Scaffold4160:2129637-2136050+	6415	622	69665.68	5.14	99	74	-0.449	43.80	84.00	Nucleus

Phylogenetic analysis of tea MAPKKKs

A phylogenetic analysis of the putative tea MAPKKK genes was carried out to evaluate the evolutionary relationships. MEGA 7.0.14 was used to generate the phylogenetic trees, using the Neighbor-Joining (NJ) algorithm, at default parameters and 1000 bootstrap replicates. Three different phylogenetic trees were constructed for MEKK, Raf and ZIK proteins, comprising of the identified tea sequences and already known 415 MAPKKK sequences from Arabidopsis, rice, tomato, potato, capsicum and coffee. For MEKK, the NJ tree was generated using 17 sequences from tea, 21 sequences from Arabidopsis, 22 sequences from rice, 17 sequences from tomato, 22 sequences from potato, 17 sequences from capsicum and 12 sequences from coffee (Fig. 1A). The NJ tree was divided into 4 distinct clades, with an uniform distribution of genes in Clade A. Clade B consisted of only 6 capsicum genes while clade D had only 2 genes of potato. Clade C however, had a share of tomato and potato gene clusters. Clade A also featured an orthologous pair (AtMEKK15 and TEA005306.1) depending on their phylogenetic relationship. For Raf, the NJ tree was generated using 31 sequences from tea, 48 sequences from Arabidopsis, 43 sequences from rice, 44 sequences from tomato, 43 sequences from potato, 37 sequences from capsicum and 28 sequences from coffee (Fig. 1B). Unlike the MEKK tree, the Raf tree was divided into 11 different clades, with an uniform clustering of genes in all the clades. However, the Raf tree did not feature any orthologous gene pair. The NJ tree for ZIK was generated using 11 sequences from tea, 11 sequences from Arabidopsis, 10 sequences from rice, 10 sequences from tomato, 16 sequences from potato, 6 sequences from capsicum and 8 sequences from coffee (Fig. 1C).

The ZIK tree was divided into 7 clades and had a uniform clustering of genes in all the clades with only clade E consisting of 2 genes each of Arabidopsis and rice. Similar to the Raf tree, the ZIK tree also did not feature any orthologous gene pair.

Fig. 1: Phylogenetic tree of (A) MEKK-like (B) Raf-like and (C) ZIK-like genes from *Arabidopsis thaliana* (black), *C. sinensis* (red), *Oryza sativa* (blue), *Solanum lycopersicum* (grey), *Solanum tuberosum* (green), *Capsicum annum* (brown), *Coffee canephora* (teal). The full-length MEKK, Raf and ZIK protein sequences were aligned using Clustal W, and the phylogenetic trees were constructed using MEGA 7.0.14 by the Neighbor-Joining (NJ) method with default parameters and 1000 bootstrap replicates.

Domain analysis of tea MAPKKKs

The MAPKKK domain architecture as reported in known literature reveals that proteins belonging to Raf family in Arabidopsis and other plant species, possess a C-terminal kinase domain and a long N-terminal regulatory domain. The proteins belonging to ZIK family on the contrary possess a N-terminal kinase domain. The proteins belonging to the MEKK family however has less conserved domain architecture and is either located either at the N- or C- terminal or at a much central region of the protein [6].

Out of the 3 subgroups of plant MAPKKKs, the MEKK subfamily is fairly well known and characterized. Most MEKKs are known to be a part of the recognized MAP Kinase cascades, which activates the downstream MKKs. MEKK1 and MEKK2 from Arabidopsis, have been proven to play a significant role in plant innate immunity [30, 31, 32]. Similar to other plant MAPKKKs, 16 out of 17 members of MEKK subfamily in tea ~~structured~~ a characteristic conserved signature G(T/S)Px(W/Y/F)MAPEV, except TEA014429.1 (Fig. 2A). Two of the most widely studied Arabidopsis Raf subfamily MAPKKKs, namely CTR1 and EDR1 are known to actively participate in ethylene mediated signalling and defense response mechanisms. All 31 members of the Raf subfamily in tea featured a conserved GTxx(W/Y)

MAPE signature in its kinase domain with no exceptions (Fig. 2B). The ZIK-like MAPKKKs are also known by the name WNK or with no lysine (K). They are not proven to be involved with the phosphorylation of the MKKs in plants however, have specific functions. Arabidopsis ZIK1 is known to phosphorylate APRR3 *in-vitro*, which is a putative component of the circadian clock in plants and is believed to be involved in signal transduction pathway, regulating its biological activity [33]. Another ZIK cascade, involving ZIK2, ZIK5 and ZIK8 in Arabidopsis is known to regulate the flowering time by modulating the photoperiod [34]. The ZIK subfamily featured a characteristic GTPEFMAPE(L/V/M)(Y/F/L) conserved signature across all its 11 members in tea (Fig. 2C) [5, 6]. The presence of these distinctive conserved signatures across the tea MAPKKKs further confirms identity and the subfamily they belong. The largest subfamily was found to be the Raf subfamily with 31 members, while the smallest was found to be the ZIK subfamily with only 11 members. This result showed consistency when compared with known literature on other plant MAPKKKs.

Fig. 2: Alignment of MAPKKKs of (A) MEKK subfamily (B) Raf subfamily and (C) ZIK subfamily in *C. sinensis*. ClustalX program was used for aligning the obtained sequences. The highlighted part (G(T/S)Px(F/Y/W)MAPEV) shows the conserved signature for the MEKK proteins. The highlighted section (GTxx(W/Y)MAPE) shows the conserved signature for the Raf proteins and the highlighted part (GTxx(W/Y)MAPE) shows the conserved signature for the ZIK proteins.

Motif composition of tea MAPKKKs

To understand the evolution and comprehend sequential characteristics of the MAPKKK proteins in tea, a conserved motif search was carried out using the MEME suite (Fig. 3). Ten conserved motifs were identified in each of the three subfamilies. Almost all the tea MAPKKK proteins featured the protein kinase domain of motif 1, motif 2 and motif 3. Motif 4 was conserved across all the proteins with only one exception of TEA031230.1. Motif 5, motif 7 and motif 8 were only obtained for the ZIK subfamily with one exception of a

MEKK-like TEA014429.1, which also featured motif 8. Motif 6 and motif 9 were harboured by almost all the protein sequences. However, motif 10 was only specific to the MEKK and Raf subfamilies. Motif annotation revealed that motif 2 harboured a protein kinase ATP-binding site. Motif 6 contained a tyrosine kinase phosphorylation site. Motif 9 featured a serine/threonine protein kinase activation site (Supplementary Fig. S4). The results suggested that proteins belonging to a same group harboured similar conserved motifs, further indicating that the classification of the tea MAPKKK subfamilies was backed by motif analyses.

Fig. 3: The motif analysis of 59 identified MAPKKKs in *C. sinensis*. The motif figures were generated by MEME suite. A total of 10 motifs were identified and are marked individually.

Gene structure analysis of tea MAPKKKs

The intron-exon distribution pattern for tea MAPKKKs were analysed and visualised using the Gene Structure Display Server v2.0. Study of gene structure revealed differences in number of introns and exons, which contributes to variation in gene length. Introns or non-coding sequences are found abundantly within a genome and are regarded as an indicator of genome complexity [35, 36]. Analysis of the intron patterns could help to comprehend and provide insights into the evolution, function and regulation of the genes [35, 37, 38, 39, 40]. The analysis of the intron-exon architecture in tea revealed significant variation in the number of introns and exons among the three subfamilies of MAPKKKs (Fig. 4). However, genes belonging to the same clades had similar intron-exon distribution. The MEKK subfamily had 10 out of 17 genes (59% of the MEKK genes) possessing 6 to 16 exons (Fig. 4A). TEA025870.1 had 19 exons and 18 introns in its gene. Two genes possessed 2 exons and 1 intron and the remaining 4 genes had no introns. Only 9 out of 17 genes featured UTR segments and 5 out of these 9 genes featured both 5’ and 3’ UTRs. 3 genes contained only the

5’ UTR segments and 1 gene only had the 3’ UTR segment. The genes belonging to the Raf subfamily had exons ranging from 6 to 18 and was featured by 27 out of 31 genes (87% of the Raf genes) (Fig. 4B). TEA016969.1 featured a staggering 28 exons and was the highest among all the Raf genes. Three genes namely TEA000933.1, TEA013875.1 and TEA033556.1 had 2, 3 and 4 exons respectively and were the lowest among the all Raf genes. 29 out of 31 genes possessed UTR segments. However, only 17 of the 29 genes had both 5’ and 3’ UTRs. 7 genes featured only the 5’ UTR segment and remaining 5 genes only had the 3’ UTR. Unlike the MEKK and Raf subfamilies, ZIK subfamily displayed a certain level of conservancy with respect to the number of exons and introns. 10 out of 11 ZIK genes (91% of the ZIK genes) had exons ranging from 7 to 10 (Fig. 4C). TEA020112.1 however featured only 2 exons. 9 out of 11 genes possessed UTR segments and 5 of them had both 5’ and 3’ UTRs. 4 genes featured only the 5’ UTR segment. However, no ZIK subfamily gene in tea featured only the 3’ UTR segment like the MEKK and Raf subfamilies.

Fig. 4: The intron/exon architecture of (A) MEKK (B) Raf and (C) ZIK genes in *C. sinensis.* Gene structure maps were drawn using the Gene Structure Display Server 2.0. Black boxes represent exons, blue boxes represent the UTRs and black lines represent introns. The gene length can be estimated by using the scale (in kb) given at the bottom.

Genomic distribution map and evolutionary pressure ~~of~~ tea MAPKKKs

The tea MAPKKKs ~~was~~ mapped onto the genomic scaffolds to understand their distribution pattern. Due to the lack of chromosome-level assembly data in the TPIA database, the genes were mapped onto their respective scaffolds instead of the chromosomes. All 59 tea MAPKKKs were extensively distributed across 58 different genomic scaffolds. 17 MEKK

genes were distributed across 17 different scaffolds (Fig. 5A). Similarly, 31 Raf genes were distributed across 31 genomic scaffolds (Fig. 5B). 11 ZIK genes were mapped onto 10 genomic scaffolds (Fig. 5C). Two ZIK genes namely, TEA013344.1 and TEA013346.1 were mapped on the same genomic scaffold 5883 and thus featured a duplication event. Additionally, both these genes possessed similar intron-exon architecture. This result is conclusive evidence that duplication events were of significant importance and played a crucial role in the expansion of the MAPKKK genes in *C. sinensis* genome. Further, the ratio of non-synonymous substitution rates (K_a) and synonymous substitution rates (K_s) was evaluated to illuminate the mechanism of gene divergence and evolutionary pressure ~~of~~ the tea MAPKKKs. The ratio determines the selective pressure acting on the respective proteins. If the K_a/K_s ratio is <1, it determines negative or purifying selection. If the K_a/K_s ratio is =1, it indicates neutral selection and if the K_a/K_s ratio is >1, it signifies positive selection [41]. For the MEKK subfamily, pair wise comparisons revealed that 72 gene pairs had K_a/K_s ratios above 1, indicating that they are under positive selection, 24 gene pairs had values less than 1, indicating a negative selection and remaining 40 were not a number (Nan) (Supplementary Table S4). Similarly, K_a/K_s ratios of the Raf subfamily revealed 341 gene pairs in positive selection, 96 in negative selection and 28 pairs as Nan (Supplementary Table S5). K_a/K_s ratios of ZIK subfamily uncovered 30 pairs in positive selection, 21 in negative selection and the remaining 4 as Nan (Supplementary Table S6). The K_a/K_s cumulative graphs of tea MAPKKKs were also generated (Supplementary Fig. S5, Supplementary Fig. S6 and Supplementary Fig. S7). The results suggest strong positive selection pressures would have occurred, enabling different factors to regulate the MAPKKKs in *C. sinensis*.

Fig. 5: The scaffold distribution of (A) MEKK subfamily (B) Raf subfamily and (C) ZIK subfamily genes in *C. sinensis*. MapGene2chromosome web v2 (MG2C) software tool (http://mg2c.iask.in/mg2c_v2.1/) was used to map genes onto their respective scaffolds. The scaffolds are drawn to scale and the scaffold numbers are indicated on the top.

Functional interaction network of tea MAPKKKs

For better understanding of the interactions of ~~tea~~ MAPKKKs in *C. sinensis*, an interaction network was constructed based on the orthologous genes in Arabidopsis, using the STRING server (Fig. 6). The functional interaction network of the genes has been built using that of Arabidopsis because tea database is not included in the STRING online server. TEA005306.1 in tea was found to be orthologous to AT5G55100 in Arabidopsis. This orthologous gene was identified using the TPIA database and AT5G55100 was used to build the interaction network. Additionally, tea proteins, homologous to the Arabidopsis proteins participating in the network were incorporated in the figure. These homologous proteins were designated as STRING proteins and were selected on the basis of high bit scores. Similarity searching programs such as BLAST produce accurate statistical estimates that help determine that protein sequences sharing substantial degree of similarities tend to have similar structures [42]. Proteins that have high sequence and structural similarity generally tend to possess similar functions [43]. AT5G55100 is involved in RNA processing and is expressed during 15 growth stages in 24 different plant structures. It shows interactions with AT4G33690 which is involved in biological process of protein binding. AT2G29210 is involved with RNA splicing, mRNA processing and is expressed during 13 different growth stages in 23 plant structures. ATO (AT5G06160) encodes for a protein similar to pre-mRNA splicing factor SF3a60 and is involved in gametic cell fate determination. Loss of function results in the ectopic expression of egg cell makers, thereby suggesting a role in restriction of gametic cell fate. TK1 (AT2G36960) is a TSL-kinase interacting protein and is involved in protein binding. It is expressed in 14 developmental stages in 25 different plant structures. GPT (AT2G41490) is an integral component of membrane and has a UDP-N-acetylglucosamine-dolichyl-phosphate N-acetylglucosamine phosphotransferase activity. It is expressed during

15 developmental stages in 23 plant structures. AT3G57220 is located in the endoplasmic reticulum and has a UDP-N-acetylglucosamine-dolichyl-phosphate N-acetylglucosamine phosphotransferase activity. It is also linked with polysaccharide biosynthesis and is expressed during 10 growth stages in 16 different plant structures.

Fig. 6: Functional interaction network of tea MAPKKK proteins. The interaction network was build according to the ortholog in Arabidopsis. TEA005306.1 in tea is orthologous to AT5G55100 in Arabidopsis. The orthologous protein (red) and homologous proteins (black) are shown within brackets.

Tissue specific ~~developmental~~ gene expression of tea MAPKKKs

The tissue specific expression pattern of the tea MAPKKK genes in various plant tissues were retrieved from the TPIA database where levels of expression were expressed using transcripts per million (TPM). The TPIA database houses tissue specific expression data for 7 different plant tissues which includes apical bud, flower, fruit, young leaf, mature leaf, old leaf, root and stem (Supplementary Table S7). Among the 59 tea MAPKKK genes, expression data for 58 genes were retrieved with an exception of 1 MEKK gene, TEA031689.1. All 58 genes displayed varied levels of expression, with few of the transcripts barely readable (Fig. 7). For the MEKK genes, the maximum level of expression in apical bud was shown by TEA006319.1. This gene also marked the highest level of expression in young leaf. TEA017119.1 showed highest level of expression in flower. TEA016319.1 displayed highest expression levels in fruit, mature leaf, old leaf and stem. TEA005122.1 was expressed maximum in root. TEA028357.1 and TEA009902.1 had negligible levels of expression in all of the 7 plant tissues (Fig. 7A). For the Raf genes, TEA000933.1 showed highest levels of expression in apical bud, fruit, young leaf, mature leaf, old leaf, root and stem. TEA007232.1 was expressed maximum in flower. However, TEA001765.1, TEA013270.1, TEA028758.1 and TEA031230.1 had negligible levels of expression (Fig.

7B). Finally, for the ZIK genes, TEA002087.1 displayed highest levels of expression in apical bud, flower, young leaf and stem. TEA022762.1 had highest levels of expression in fruit, mature leaf and old leaf. TEA020112.1 showed maximum expression in root. However, TEA013344.1, TEA031068.1, TEA020698.1 and TEA027328.1 showed minor levels of expression (Fig. 7C). Heat maps for all the 58 genes, representing the tissue specific expression levels were also being generated (Supplementary Fig. S8).

Fig. 7: Tissue-specific expression patterns of (A) MEKK (B) Raf and (C) ZIK genes in *C. sinensis*. The relative expression of these genes was analysed in different ~~developmental stages~~, by using GraphPad Prism 8 software. The level of expression was in transcript per million (TPM). 58 out of the 59 identified genes had expression data in TPIA database with an exception of 1 MEKK gene (TEA031689.1).

Abiotic stress induced differential expression levels of tea MAPKKKs

To check the effect of various abiotic stress ~~tolerance levels of tea MAPKKKs~~, the expression data was retrieved from the TPIA database (Supplementary Table S8; Supplementary Table S9; Supplementary Table S10 and Supplementary Table S11) and expression graphs were generated (Fig. 8, Fig. 9, Fig. 10 and Fig. 11). The Tea Plant Information Archive database houses stress ~~tolerance data~~ for cold stress, drought stress, salt stress and methyl jasmonate (MeJA) treatment. The cold acclimated (CA) data (unpublished), present in the TPIA database consists of 5 stages of expression. These are: 1. 25~20 °C (CK), 2. Fully acclimated at 10 °C for 6 h (CA1-6h) 3. 10~4 °C for 7 days (CA 1-7d), 4. Cold response at 4~0 °C for 7 days (CA 2-7d) and 5. Recovering under 25~20 °C for 7 days (DA-7d), where CK is the control [44]. Expression of MEKK genes revealed that 15 out of 17 genes were upregulated under CA 1-6h. TEA006473.1 was downregulated while TEA031689.1 displayed no expression levels. Expression levels under the CA 1-7d condition showed that 12 genes were upregulated, 4 genes were downregulated and remaining 1 gene showed no data. Under the CA 2-7d condition, expression levels revealed that 10 genes were

upregulated, 6 genes were downregulated and remaining 1 gene displayed no expression data. Lastly, under the DA-7d condition, data revealed that 13 genes showed upregulation, 3 genes showed downregulation and 1 gene had no data (Fig. 8A). ~~Expression~~ of the Raf and ZIK genes were also ~~carried~~ based on the same 5 conditions. For the Raf genes, under CA 1-6h condition, 22 genes out of 31 were upregulated and 9 genes were downregulated. Under CA 1-7d condition, 16 genes were upregulated and 15 genes were downregulated. Expression levels under CA 2-7d revealed that 17 genes showed upregulation and remaining 14 genes showed downregulation. Under DA-7d condition, 21 genes were upregulated and 10 genes were downregulated (Fig. 8B). Expression data of the ZIK genes revealed that under CA 1-6h, 7 out of 11 genes were upregulated and 4 genes were downregulated. CA 1-7d condition revealed that 5 genes were upregulated, 5 genes were downregulated and remaining 1 gene displayed no expression. Under CA 2-7d condition, 4 genes were upregulated, 6 genes were downregulated and 1 gene had no expression. Finally, under DA-7d, 8 genes showed upregulation and remaining 3 showed downregulation (Fig. 8C). Heat maps for the retrieved expression data were also generated (Supplementary Fig. S9).

Further, expression levels of all tea MAPKKKs were checked under the effects of drought stress conditions. Drought stress levels are recorded in the TPIA database with respect to 25% polyethylene glycol (PEG) treatment and it includes 4 different stages: 1. 0h; 2. 24h; 3. 48h; and 4. 72h [45], where 0h was taken as the control. The expression levels of MEKK genes revealed that under PEG-N-24h condition, 12 genes were upregulated, 4 were downregulated and 1 gene did not show any expression. Under PEG-N-48h, 12 genes were upregulated, 4 were downregulated and 1 gene showed no expression. PEG-N-72h revealed 11 genes showing upregulation, 5 genes showing downregulation and 1 gene with no expression (Fig. 9A). Expression of Raf genes showed that under the PEG-N-24h condition, 11 genes were upregulated, 20 genes were downregulated. Under PEG-N-48h, 16 genes

showed upregulation while the remaining 15 genes were downregulated. PEG-N-72h revealed that 15 genes were upregulated and 16 genes were downregulated (Fig. 9B). Finally, the expression data of ZIK genes revealed 10 out of 11 genes had different expression levels under the given conditions while 1 gene (TEA013344.1) had no data. Under the PEG-N-24h condition, expression data showed that only 1 gene was upregulated while the rest of the genes were downregulated. PEG-N-48h condition too revealed the same result with only 1 gene being upregulated. However, PEG-N-72h showed that 2 genes were upregulated and the rest of the genes were downregulated (Fig. 9C). Heat maps for the afore-mentioned data were also generated (Supplementary Fig. S10).

The expression levels of the tea MAPKKKs under salt stress condition were studied. Similar to the drought stress parameters, the salt stress data in TPIA database is recorded based on treatment with 200 mM NaCl under 4 stages: 1. 0h; 2. 24h; 3. 48h; and 4. 72h where 0h was taken as the control. Analysis of the MEKK genes revealed that under NaCl-N-24h, 9 genes were upregulated and 8 genes were downregulated. For NaCl-N-48h condition, 9 genes showed upregulation and remaining 8 genes were downregulated. Expression levels under NaCl-N-72h revealed 5 genes being upregulated and the rest being downregulated (Fig. 10A). For the Raf genes, expression data concluded that under NaCl-N-24h condition, 15 genes were upregulated and 16 genes were downregulated. Under the NaCl-N-48h condition, 16 genes showed upregulation and 15 genes were downregulated. Expression levels under NaCl-N-72h showed that 8 genes were upregulated and remaining 23 were downregulated (Fig. 10B). For ZIK genes, 10 out of 11 genes had expression levels with 1 gene (TEA013344.1) showing no effect under the given conditions. Expression levels determine that under NaCl-N-24h condition, only 2 genes showed upregulation and the rest of the genes were downregulated. For NaCl-N-48h condition, only 1 gene was upregulated while the remaining 9 were downregulated. NaCl-N-72h condition too revealed a similar result with 2 genes being

upregulated and remaining 8 being downregulated (Fig. 10C). Heat maps were generated for the above-mentioned data as well (Supplementary Fig. S11).

Finally, the expression levels of the tea MAPKKKs under MeJA treatment were studied and analysed. The hormonal treatment data is recorded based on the results of exposing the plant parts to water solution of MeJA, under 4 stages: 1. 0h: 2. 12h: 3. 24h and 4. 48h where 0h was used as the control. For the MEKK genes, under the 12h_MeJA condition, 3 genes showed upregulation, 13 genes were downregulated and remaining 1 gene had no expression at all. Under the 24h_MeJA condition, 4 genes were upregulated, 12 were downregulated and 1 gene was not expressed at all. Under 48h_MeJA condition, 8 genes were upregulated and 9 genes were downregulated (Fig. 11A). Similarly, for the Raf genes, treatment under 12h_MeJA condition revealed that 10 out of 31 genes were upregulated and remaining 21 genes were downregulated. Under 24h_MeJA condition, 8 genes showed upregulation while 23 genes were downregulated. 48h_MeJA revealed that only 4 genes were upregulated and rest of the genes were downregulated (Fig. 11B). ~~Treatment~~ of the ZIK genes under the 12h_MeJA condition revealed that 4 genes were upregulated and 7 genes were downregulated. 24h_MeJA condition showed 5 genes being upregulated and remaining 6 being downregulated. 48h_MeJA condition ~~concluded~~ that 3 genes were upregulated and remaining 8 being downregulated (Fig. 11C). Heat maps for these data were also generated (Supplementary Fig. S12).

Fig. 8: Gene expression patterns of (A) MEKK (B) Raf and (C) ZIK genes, under cold stress conditions in *C. sinensis*. The relative expression of these genes was analysed in different ~~developmental~~ stages, by using GraphPad Prism 8 software. The level of expression was in transcript per million (TPM).

Fig. 9: Gene expression patterns of (A) MEKK (B) Raf and (C) ZIK genes, under drought stress conditions in *C. sinensis*. The relative expression of these genes was analysed in different ~~developmental~~ stages, by using GraphPad Prism 8 software. The level of expression was in transcript per million (TPM).

Fig. 10: Gene expression patterns of (A) MEKK (B) Raf and (C) ZIK genes, under salt stress conditions in *C. sinensis*. The relative expression of these genes was analysed in different ~~developmental~~ stages, by using GraphPad Prism 8 software. The level of expression was in transcript per million (TPM).

Fig. 11: Gene expression patterns of (A) MEKK (B) Raf and (C) ZIK genes, under Methyl jasmonate (MeJA) treatment in *C. sinensis*. The relative expression of these genes was analysed in different ~~developmental~~ stages, by using GraphPad Prism 8 software. The level of expression was in transcript per million (TPM).

Discussion

The MAPKKK-MAPKK-MAPK signalling cascade is a key component in response to various environmental stresses and plant developmental stages [5, 32, 46]. Investigation of the MAPKKK genes, which form a significant component of this core regulatory network ~~in the pathway~~, would certainly aid to a better understanding of the signalling genes. Genome wide studies have previously identified MAPKKK genes in various plant species, which include 80 genes in Arabidopsis [31], 75 genes in *Oryza sativa* [6], 71 genes in *Zea mays* [37], 89 genes in tomato [9], 59 genes in *Cucumis sativus* [10], 150 genes in *Glycine max* [47], 77 genes in banana [48], 155 genes in *Triticum aestivum* [49], 62 genes in cassava [50] and 73 genes in *Medicago truncatula* [51]. However, tea plant has been explored the least and MAPKKK signalling genes have not been studied yet. This ~~conducted~~ study provided a comprehensive synopsis of the phylogenetic relationship, intron-exon architecture, motifs, functional domains, genomic distribution and expression patterns of the MAPKKK genes in tea. Herein, a grand total of 59 MAPKKK proteins were screened and identified from tea plant genome. The identified genes were then classified into 3 subfamilies based on their phylogenetic relationships (Fig. 1). Previous studies on MAPKKK gene families in Arabidopsis, cucumber and rice also yielded consistent results with respect to the classification of the identified genes into 3 subfamilies [6, 10, 31]. The classification of the

MAPKKK genes was further backed by domain analyses, motifs and gene structure studies. All the identified tea MAPKKKs had their respective subfamily specific domains. The MEKK subfamily genes featured a less conserved domain signature G(T/S)Px(W/Y/F) MAPE, with one exception (Fig. 2A). The Raf subfamily featured a conserved GTxx(W/Y)MAPE signature (Fig. 2B) and ZIK subfamily genes had a characteristic GTPEFMAPE(L/V/M)(Y/F/L) conserved signature (Fig. 2C). Motif analyses revealed that all MAPKKK proteins had protein kinase domains and proteins belonging to the same subfamily shared similar motifs (Fig. 3). This result is consistent to previous studies conducted on other plants like cucumber [10], Arabidopsis [31] and banana [48]. The intron-exon architecture of the tea MAPKKK genes revealed a significant variation in the number of introns and exons (Fig. 4). The analysis also proposed that genes belonging to the same subgroup featured similar intron-exon organisation. MEKK genes displayed exons ranging from 6 to 16 on an average. Highest number of exons found among the MEKK genes was 18. Raf genes had an average of 6 to 18 exons with the highest being a staggering 28 and ZIK genes had 7 to 10 exons on an average. Raf subfamily thereby featured more number of introns than MEKK and ZIK subfamilies. Reports suggest that the rate at which introns are lost is faster compared to the rate at which introns are gained after segmental duplication [52]. This is a conclusive evidence to infer that Raf subfamily might contain the original set of genes, from which genes of other subfamilies have been derived. The MAPKKK genes also displayed a significant variation with respect to the ~~number of~~ UTR segments ~~present~~. Most genes possessed both 5' and 3' UTRs while few had only the 5' UTR or 3' UTR segment. These variations of the gene structures in large scale suggest that the tea genome has been variable during its evolutionary history. Similar occurrence was also observed in plants like cassava [50], grapevine [53] and maize [37]. All the identified genes were mapped onto their respective scaffolds (Fig. 5). Duplication events were observed among the ZIK genes and

further the ratio of non-synonymous substitution rates (K_a) and synonymous substitution rates (K_s) was evaluated which indicated strong positive selection pressures to have occurred, enabling different factors to regulate the MAPKKKs in *C. sinensis* genome. Functional interaction network was also constructed based on the tea orthologs in Arabidopsis (Fig. 6). The genes participating in the interaction network revealed genes responsible for various biological processes during developmental stages. Additionally, tea proteins homologous to the Arabidopsis proteins participating in the interaction network were also added. These homologous proteins were considered STRING proteins because of the fact that proteins possessing high sequence and structural similarity tend to have similar functions.

Tea plant is a major plantation crop grown all over the world due to its high commercial value. Leaves obtained from tea plant are rich sources of nutrients and tea is the second most important beverage in the world besides water. However, being a thermophilic crop, tea plant is largely affected by various environmental stress factors [44, 48]. Plants undergo many biochemical, molecular and physiological changes to defend themselves against environmental damage by evolving numerous signalling pathways to react to the external stimuli into intracellular reactions [54, 55]. MAPKKKs function at the highest level of the MAPK signalling cascade, helping with developmental and stress tolerance in plants. Previous studies have established the positive role of plant MAPKKKs in tolerance to various abiotic stress factors like cold, salt and drought [56, 57, 58, 59]. Through this cascade, these external stress stimuli are being converted into cellular responses [59]. The simplest MAPK pathway comprises of 3 kinases, namely: mitogen-activated protein kinase kinase kinase (MAPKKK/MEKK), mitogen-activated protein kinase kinase (MAPKK/MEK) and mitogen-activated protein kinase (MAPK/MPK). These 3 kinases are sequentially activated. A receptor mediated activation of MAPKKK further activating a MAPKK, and MAPKKs phosphorylate specific MAPKs [60]. MAPKKKs have been extensively studied in

Arabidopsis and have been characterised. Previous literatures have conveyed that MEKK1-MKK1-MPK4 cascade is activated following a wounding stress response [61]. The MEKK1-MKK2-MPK4/MPK6 cascade is stimulated in salt and cold stress conditions [62]. MEKK1-MKK4/MKK5-MPK3/MPK6 cascade is involved with plant protection to pathogen infections [63]. MKK3 encodes for a Mitogen Activated Protein Kinase Kinase, that stimulates MPK8, and is a target of MPKKK20, regulating ROS accumulation. MKK3-MAPKKK17-MAPKKK18 form an element of the ABA signalling pathway. MAPKKK17 and MAPKKK18 belong to Ser/Thr protein kinase family and help in the ABA-dependent activation of the MKK3-MPK7 pathway.

Examination of gene expression in different plant parts and in response to various environmental stress stimuli is a key to understand the functionality of the genes. Here in this study, tissue specific gene expression data and abiotic stress ~~tolerance~~ data were retrieved from the TPIA database and analysed. With respect to the tissue specific gene expression data, 58 genes out of the 59 identified genes had expression data in TPIA database. The database stores expression data for ~~7~~ different plant tissues which includes apical bud, flower, fruit, young leaf, mature leaf, old leaf, root and stem. Among the MEKK genes, TEA016319.1 was expressed consistently in all the ~~7~~ plant tissues than other genes (Fig. 7A). While for the Raf and ZIK genes, TEA000933.1 and TEA002087.1 were the consistently expressed genes (Fig. 7B and Fig. 7C).

Reactive oxygen species (ROS) are oxygen derivatives, which are highly reactive by-products of the aerobic metabolism [64]. Plants consist of a complicated network of ROS scavenging antioxidant enzymes that help regulate the ROS levels under normal physiological conditions [64, 65]. Although a shift from normal physiological conditions to adverse environmental conditions shifts the equilibrium, resulting in increased ROS production. ROS are highly toxic to the cellular machinery and increased ROS levels can lead

to serious oxidative damage and cell death [65]. Studies have suggested that the MAPK signalling cascade comprising of the MAPKKK-MAPKK-MAPK module is stimulated when excess ROS levels are detected under multiple stress conditions such as salt stress, cold stress and drought stress [64, 65]. In tomato, most of the MAPKKK genes were upregulated by drought, salt, cold, and heat stress conditions out of which SlMAPKKK51, SlMAPKKK53, and SlMAPKKK55 were upregulated more [9]. MAPKKK members of some plants including GhRaf19, AtMAPKKK18, AtRaf43, and DSM1 were found to be involved in plants resistance to various abiotic stress stimuli [55, 56, 57]. Gene expression analysis ~~for~~ the tea MAPKKK genes ~~against~~ cold stress demonstrated that TEA016319.1 among the MEKK genes, TEA008343.1 among the Raf genes and TEA010125.1 among the ZIK genes were expressed the most during the CA2-7d condition (Fig. 8). Expression data ~~studied for drought stress tolerance~~ revealed that TEA005122.1 among the MEKK genes, TEA000933.1 among the Raf genes and TEA022762.1 among the ZIK genes had the highest levels of expression at PEG-N-72h condition (Fig. 9). Salt stress ~~tolerance~~ data suggested that TEA005122.1 among the MEKK genes and TEA000933.1 among Raf genes displayed the highest levels of expression at NaCl-N-72h condition. TEA020112.1 among the ZIK genes was upregulated the most under NaCl-N-72h condition (Fig. 10). Expression data for treatment under Methyl jasmonate (MeJA) was also analysed. Data revealed that TEA028214.1 among the MEKK genes, TEA000933.1 among the Raf genes and TEA002087.1 among the ZIK genes were expressed the most under the 72_MeJA condition (Fig. 11). Collectively, these findings suggest the involvement of a number of MAPKKK genes, being upregulated and expressed under the stress conditions. In general, this study provides a detailed and comprehensive analysis of the MAPKKK genes in tea. Further extensive studies needs to be conducted on MAPK genes in tea that could underwrite a better understanding of various functions of these set of genes in developmental processes and expression under various abiotic stress stimuli.

Conclusion

Mitogen activated protein kinases (MAPK) signalling cascade plays significant roles in different biological processes. The signalling components are linked to the upstream and downstream regulators by phosphorylation. There has been substantial development in identifying the different MAPKKK genes and understand their physiological roles in various plants. However, these genes had yet not been explored and studied in tea plant. *In-silico* genome wide analysis had identified 59 MAPKKK genes from *C. sinensis* genome. The classification of the identified MAPKKK genes in 3 subfamilies were conducted based on their phylogenetic relationships. The genes were further investigated under domains signatures, conserved protein motifs and intron-exon architecture. The classification of the identified MAPKKK genes into their subfamilies was backed by the results obtained from the above analyses made. The 59 genes were mapped onto their respective genomic scaffolds and a network of functionally interacting genes was constructed. Further, expression profile analyses were conducted to reveal the involvement of the tea MAPKKK genes in various tissues during development and also check the expression of these genes under various abiotic stress stimuli and plant hormonal treatment. These data will provide detailed information about the tea MAPKKK genes for further characterization of the MAPK signalling cascade and lay a concrete foothold for further exploration and research on *C. sinensis*.

Acknowledgements

This project was supported by the Key Technologies R & D Program for Crop Breeding of Zhejiang Province (2016C02054-19,2017C02010), the Natural Science Foundation of China (31670303), and the Joint Laboratory of Olive Oil Quality and Nutrition among China,

Australia and Spain. The authors are thankful to DBT-eLibrary Consortium (DeLCON) for providing access to e-resources.

Author contributions: A.P., A.P.S. and S.S., designed and performed experiments, A.P.S., N.M., and G.S., devised the experiments, helped in data analysis and writing the manuscript.

Author details: Abhirup Paul: Department of Biochemistry, REVA University, Bangalore, Karnataka, India (Email: abhirupm16@gmail.com); Anurag P. Srivastava: Department of Life Sciences, Garden City University, Bangalore, Karnataka, India (Email: anurag.srivastava@gardencity.university, anuiitkgp@gmail.com); Shreya Subrahmanya: Department of Botany, St. Joseph's college autonomous, Bengaluru, Karnataka, India (Email: shreyasub916@gmail.com); Guoxin Shen: Sericultural Research Institute, Zhejiang Academy of Agricultural Sciences, Hangzhou 310021, China (Email: guoxin.shen@ttu.edu); Neelam Mishra: Department of Botany, St. Joseph's college autonomous, Bengaluru, Karnataka, India (Email: neelamiitkgp@gmail.com, neelammishra@sjc.ac.in).

Funding: Not Applicable

Data availability: All data generated or analysed during this study are included in this article and are provided in the Electronic Supplemental Materials (ESM_1 and ESM_2)

Compliance with ethical standards

Competing Financial Interests: There are no competing financial interests

Ethics approval: Not applicable

References

1. Champion, A., Picaud, A. & Henry, Y. Reassessing the MAP3K and MAP4K relationships. *Trends Plant Sci.* (2004). **9**, 123–129
2. Rodriguez, M. C., Petersen, M. & Mundy, J. Mitogen-activated protein kinase signaling in plants. *Annu. Rev. Plant Biol.* (2010). **61**, 621–649
3. Doczi, R., Okresz, L., Romero, A. E., Paccanaro, A. & Bogre, L. Exploring the evolutionary path of plant MAPK networks. *Trends Plant Sci.* (2012). **17**, 518–525

4. Colcombet, J. & Hirt, H. Arabidopsis MAPKs: A complex signalling network involved in multiple biological processes. *Biochem. J.* (2008). **413**, 217–226
5. Jonak, C., Okresz, L., Bogre, L. & Hirt, H. Complexity, cross talk and integration of plant MAPKinase signalling. *Current opinion in plant biology*. (2002). **5**, 415–424
6. Rao, K.P., Richa, T., Kumar, K., Raghuram, B. & Sinha, A. K. In silico analysis reveals 75 members of mitogen-activated protein kinase kinase kinase gene family in rice. *DNA Research.* (2010). **17,** 139–153
7. Sinha, A. K, Jaggi, M., Raghuram, B. & Tuteja, N. Mitogen-activated protein kinase signaling in plants under abiotic stress. *Plant signaling & behavior.* (2011). **6**, 196–203
8. Hamel, L. P., Sheen, J. & Seguin, A. Ancient signals: comparative genomics of green plant CDPKs. *Trends in plant science.* (2014). **19,** 79–89
9. Wu, J., Wang, J., Pan, C., Guan, X., Wang, Y., Liu, S., He, Y., Chen, J., Chen, L. & Lu, G. Genome-wide identification of MAPKK and MAPKKK gene families in tomato and transcriptional profiling analysis during development and stress response. *PloS one.* (2014). **9**,e103032. https://doi.org/10.1371/journal.pone.0103032.
10. Wang, J., Pan, C., Wang, Y., Ye, L., Wu, J., Chen, L., Zou, T. & Lu, G. Genome-wide identification of MAPK, MAPKK, and MAPKKK gene families and transcriptional profiling analysis during development and stress response in cucumber. *BMC Genomics.* (2015). **16,** 386
11. Popescu, S. C., Popescu, G. V., Bachan, S., Zhang, Z., Gerstein, M., Snyder, M. & Dinesh Kumar, S. P. MAPK target networks in Arabidopsis thaliana revealed using functional protein microarrays. *Genes Dev.* (2009). **23,** 80-92
12. Huang, X., Luo, T., Fu, X., Fan, Q. & Liu J. Cloning and molecular characterization of a dehydration/drought tolerance in transgenic tobacco. *J. Exp. Bot.* (2011). **62**, 5191–5206
13. Frye, C. A., Tang, D. & Innes, R. W. Negative regulation of defense responses in plants by a conserved MAPKK kinase. *Proc. Natl. Acad. Sci. U.S.A.* (2001). **98,** 373–378
14. Xia, E. H., Li, F. D., Tong, W., Li, P. H., Wu, Q., Zhao, H. J., Ge, R. H., Li, R. P., Li, Y. Y., Zhang, Z. Z., Wei, C. L. & Wan, X. C. Tea Plant Information Archive (TPIA): A comprehensive genomics and bioinformatics platform for tea plant. *Plant Biotechnology Journal.* (2019). **17,** 1938–1953
15. Berardini, T. Z., Reiser, L., Li, D., Mezheritsky, Y., Muller, R., Strait, E. & Huala, E. The Arabidopsis Information Resource: Making and mining the "gold standard" annotated reference plant genome. *Genesis*. (2015). **53,** 474–485
16. Kawahara, Y., de la Bastide, M., Hamilton, J. P., Kanamori, H., McCombie, W. R., Ouyang, S., Schwartz, D. C., Tanaka, T., Wu, J., Zhou, S., Childs, K. L., Davidson, R. M., Lin, H., Quesada-Ocampo, L., Vaillancourt, B., Sakai, H., Lee, S. S., Kim, J., Numa, H., Itoh, T., Buell, C. R. & Matsumoto, T. Improvement of the Oryza sativa Nipponbare reference genome using next generation sequence and optical map data. *Rice.* (2013). **6,** 4
17. Fernandez-Pozo, N., Menda, N., Edwards, J. D., Saha, S., Tecle, I. Y., Strickler, S. R., Bombarely, A., Fisher-York, T., Pujar, A., Foerster, H., Yan, A. & Mueller, L. A.

The Sol Genomics Network (SGN)--from genotype to phenotype to breeding. *Nucleic Acids Res.* (2015). **43**, (Database issue):D1036-D1041 doi:10.1093/nar/gku1195
18. Letunic, I., Doerks, T. & Bork, P. SMART: recent updates, new developments and status in 2015. *Nucleic Acids Res.* (2015). **43,** (Database issue):D257-D260 doi:10.1093/nar/gku949.
19. Gasteiger, E., Hoogland, C., Gattiker, A., Duvaud, S., Wilkins, M. R., Appel, R. D. & Bairoch, A. Protein Identification and Analysis Tools on the ExPASy Server; (In) John M. Walker (ed): The Proteomics Protocols Handbook, Humana Press (2005). pp. 571-607.
20. Pierleoni, A., Martelli, P. L., Fariselli, P. & Casadio, R. BaCelLo: a balanced subcellular localization predictor, *Bioinformatics.* (2006). **22,** 408-416. https://doi.org/10.1093/bioinformatics/btl222.
21. Sonnhammer, E. L. L., von Heijne, G. & Krogh, A. A hidden Markov model for predicting transmembrane helices in protein sequences. In Proc. of Sixth Int. Conf. on Intelligent Systems for Molecular Biology, (1998). p 175-182 Ed J. Glasgow, T. Littlejohn, F. Major, R. Lathrop, D. Sankoff, and C. Sensen. Menlo Park, CA: AAAI Press,
22. Korber, B. HIV Signature and Sequence Variation Analysis. Computational Analysis of HIV Molecular Sequences, Chapter 4, pages 55-72. (2000). Allen G. Rodrigo and Gerald H. Learn, eds. Dordrecht, Netherlands: Kluwer Academic Publishers.
23. Madeira, F., Park, Y. M., Lee, J., Buso, N., Gur, T., Madhusoodanan, N., Basutkar, P., Tivey, A. R. N., Potter, S. C., Finn, R. D. & Lopez, R. The EMBL-EBI search and sequence analysis tools APIs. *Nucleic Acids Res.* (2019). **47**, W636-W641 (2019). doi: 10.1093/nar/gkz268.
24. Kumar, S., Stecher, G. & Tamura, K. MEGA7: Molecular Evolutionary Genetics Analysis Version 7.0 for Bigger Datasets. *Mol Biol Evol.* (2016). **33,** 1870-1874. doi:10.1093/molbev/msw054.
25. Szklarczyk, D., Gable, A. L., Lyon, D., Junge, A., Wyder, S., Cepas, J. H., Simonovic, M., Doncheva, N. T., Morris, J. H. , Bork, P., Jensen, L. J. & von Mering, C. STRING v11: protein-protein association networks with increased coverage, supporting functional discovery in genome wide experimental datasets. *Nucleic Acids Res.* (2019). **47**, (D1):D607-D613
26. Hu, B., Jin, J., Guo, A. Y., Zhang, H., Luo, H. & Gao, G. GSDS 2.0: an upgraded gene feature visualization server. *Bioinformatics.* (2015). **31,** 1296-1297
27. Bailey, T. L., Boden, M., Buske, F.A., Frith, M., Grant, C. E, Clementi, L., Ren, J., Li, W. W. & Noble, W. S. MEME SUITE: tools for motif discovery and searching. *Nucleic Acids Res.* (2009). **37**(Web Server issue), W202-208. doi: 10.1093/nar/gkp335.
28. Jiangtao, C., Yingzhen, K., Qian, W., Yuhe, S., Daping, G, Jing, L.V. & Guanshan, L. Mapgene2chrom, a tool to draw gene physical map based on perl and svg languages. *Hereditas.* (2015). **37,** 91-97.
29. Wang, Y. X., Liu, Z. W, Wu, Z. J., Li, H., Wang, W. L., Cui, X. & Zhuang, J. Genome-wide identification and expression analysis of GRAS family transcription

factors in tea plant (Camellia sinensis). *Sci Rep.* (2018). **8,** 3949. doi: 10.1038/s41598-018-22275-z.

30. Gao, M., Liu, J., Bi, D., Zhang, Z., Cheng, F., Chen, S. & Zhang, Y. MEKK1, MKK1/MKK2 and MPK4 function together in a mitogen-activated protein kinase cascade to regulate innate immunity in plants. *Cell Res.* (2008). **18,** 1190–1198 (2008).
31. Kong, Q., Qu, N., Gao, M., Zhang, Z., Ding, X., Yang, F., Li, Y., Dong, O. X, Chen, S., Li, X. & Zhang, Y. The MEKK1-MKK1/ MKK2-MPK4 kinase cascade negatively regulates immunity mediated by a mitogen-activated protein kinase kinase kinase in Arabidopsis. *Plant Cell.* (2012). **24,** 2225–2236
32. Pitzschke, A., Djamei, A., Bitton, F. & Hirt, H. A major role of the MEKK1-MKK1/2-MPK4 pathway in ROS signalling. *Mol Plant.* (2009). **2,** 120–137
33. Murakami-Kojima, M., Nakamichi, N., Yamashino, T. & Mizuno, T. The APRR3 component of the clock-associated APRR1/TOC1 quintet is phosphorylated by a novel protein kinase belonging to the WNK family, the gene for which is also transcribed rhythmically in Arabidopsis thaliana. *Plant Cell Physiol.* (2002). **43,** 675–683
34. Wang, Y., Liu, K., Liao, H., Zhuang, C., Ma, H. & Yan, X. The plant WNK gene family and regulation of flowering time in Arabidopsis. *Plant Biol.* (2008). **10,** 548–562
35. Goyal, R. K., Tulpan, D., Chomistek, N., González-Peña Fundora, D., West, C., Ellis, B. E., Frick, M., Laroche, A. & Foroud, N. A. Analysis of MAPK and MAPKK gene families in wheat and related Triticeae species. *BMC Genomics*. (2018). **19,** 178
36. Taft, R. J., Pheasant, M. & Mattick, J. S. The relationship between non-proteincoding DNA and eukaryotic complexity. *BioEssays.* (2007). **29,** 288–299
37. Liu, Z., Shi, L., Liu, Y., Tang, Q., Shen, L., Yang, S., Cai, J., Yu, H., Wang, R., Wen, J., Lin, Y., Hu, J., Liu, C., Zhang,Y., Mou, S. & He, S. Genome wide identification and transcriptional expression analysis of mitogen-activated protein kinase and mitogen-activated protein kinase kinase genes in *Capsicum annuum*. *Front. Plant Sci.* (2015). **6**, 780 doi: 10.3389/fpls.2015.00780.
38. Zhang, G., Li, C., Li, Q., Li, B., Larkin, D. M., Lee, C., Storz, J. F., Antunes, A., Greenwold, M. J., Meredith, R.W. et al. Comparative genomics reveals insights into avian genome evolution and adaptation. *Science.* (2014). **346,** 1311–20
39. Fedorova, L. & Fedorov, A. Introns in gene evolution. In: Long M, editor. Origin and evolution of new gene functions. Dordrecht: Springer Netherlands, (2003). pp. 123–131
40. Deutsch, M. & Long, M. Intron-exon structures of eukaryotic model organisms. *Nucleic Acids Res.* (1999). **27,** 3219-3228
41. Liu, W., Li, W., He, Q., Daud, M. K., Chen, J. & Zhu, S. Genome-wide survey and expression analysis of Calcium-Dependent Protein Kinase in *Gossypium raimondii*. *PLoS One.* (2014). **9,** e98189
42. Pearson, W. R. An introduction to sequence similarity (“homology”) searching. *Current protocols in bioinformatics.* (2013). doi:10.1002/0471250953.bi0301s42

43. Gan, H. H., Perlow, R. A., Roy, S., Ko, J., Wu, M., Huang, J., Yan, S., Nicoletta, A., Vafai, J., Sun, D., Wang, L., Noah, J. E., Pasquali, S. & Schlick, T. Analysis of protein sequence/structure similarity relationship. *Biophysical Journal.* (2002). **83,** 2781-2791
44. Wang, X. C., Zhao, Q. Y., Ma, C. L., Zhang, Z. H., Cao, H. L, Kong, Y. M., Yue, C., Hao, X. Y., Chen, L., Ma, J. Q., Jin, J. Q., Li, X. & Yang, Y. J. Global transcriptome profiles of Camellia sinensis during cold acclimation. *BMC Genomics.* (2013). **14,** 415
45. Zhang, Q., Cai, M., Yu, X., Wang, L., Guo, C., Ming, R. & Zhang, J. Transcriptome dynamics of Camellia sinensis in response to continuous salinity and drought stress. *Tree Genetics & Genomes.* (2017). **13,** 78
46. Moustafa, K., AbuQamar, S., Jarrar, M., Al-Rajab, A. J. & Trémouillaux-Guiller, J. MAPK cascades and major abiotic stresses. *Plant Cell Rep.* (2014). **33,** 1217–1225
47. Neupane, A., Nepal, M. P., Piya, S., Subramanian, S., Rohila, J. S., Reese, R. N. & Benson, B. V. Identification, nomenclature, and evolutionary relationships of mitogen-activated protein kinase (MAPK) genes in soybean. *Evol Bioinform Online.* (2013). **9,** 363-386
48. Wang, L., Hu, W., Tie, W., Ding, Z., Ding, X., Liu, Y., Yan, Y., Wu, C., Peng, M., Xu, B. & Jin, Z. The MAPKKK and MAPKK gene families in banana: identification, phylogeny and expression during development, ripening and abiotic stress. (2017). *Sci Rep.* **7,** 1159
49. Wang, M., Yue, H., Feng, K., Deng, P., Song, W. & Nie, X. Genome-wide identification, phylogeny and expressional profiles of mitogen activated protein kinase kinase kinase (MAPKKK) gene family in bread wheat (*Triticum aestivum L.). BMC Genomics.* (2016). **17,** 668
50. Ye, J., Yang, H., Shi, H., Wei, Y., Tie, W., Ding, Z., Yan, Y., Luo, Y., Xia, Z., Wang, W., Peng, M., Li, K., Zhang, H. & Hu, W. The MAPKKK gene family in cassava: Genome-wide identification and expression analysis against drought stress. (2017). *Sci Rep.* **7,** 14939. https://doi.org/10.1038/s41598-017-13988-8.
51. Li,W., Xu, H., Liu, Y., Song, L., Guo, C. & Shu, Y. Bioinformatics Analysis of MAPKKK Family Genes in Medicago truncatula. *Genes (Basel).* (2016). **7,** 13. doi: 10.3390/genes7040013. PMID: 27049397; PMCID: PMC4846843.
52. Nuruzzaman, M., Manimekalai, R, Sharoni, A. M., Satoh, K., Kondoh, H., Ooka, H. & Kikuchi, S. Genome-wide analysis of NAC transcription factor family in rice. *Genes.* (2010). **465,** 30-44
53. Wang, G., Lovato, A., Polverari, A., Wang, M., Liang, Y. H., Ma, Y. C. & Cheng, Z. M. Genome-wide identifcation and analysis of mitogen activated protein kinase kinase kinase gene family in grapevine (Vitis vinifera). *BMC Plant Biol.* (2014). **14,** 219
54. Tuteja, N. Abscisic acid and abiotic stress signaling. *Plant Signal Behav.* (2007). **2,** 135–138
55. Ning, J., Li, X., Hicks, L. M. & Xiong, L. A Raf-like MAPKKK gene DSM1 mediates drought resistance through reactive oxygen species scavenging in rice. *Plant Physiol.* (2010). **152,** 876–890

56. Virk, N., Li, D., Tian, L., Huang, L., Hong, Y., Li, X., Zhang, Y., Liu, B., Zhang, H. & Song, F. Arabidopsis Raf-like mitogen-activated protein kinase kinase kinase gene Raf43 is required for tolerance to multiple abiotic stresses. *PLoS One.* (2015). **10,** e0133975
57. Jia, H., Hao, L., Guo, X., Liu, S., Yan, Y. & Guo, X. A Raf-like MAPKKK gene, GhRaf19, negatively regulates tolerance to drought and salt and positively regulates resistance to cold stress by modulating reactive oxygen species in cotton. *Plant Sci.* (2016). **252,** 267–281
58. Shitamichi, N., Matsuoka, D., Sasayama, D., Furuya, T. & Nanmori, T. Over-expression of MAP3Kδ4, an ABA-inducible Raf-like MAP3K that confers salt tolerance in Arabidopsis. Plant *Biotechnol.* (2013). **30,** 111–118
59. Shou, H., Bordallo, P. & Wang, K. Expression of the Nicotiana protein kinase (NPK1) enhanced drought tolerance in transgenic maize. *J. Exp. Bot.* (2004). **55,** 1013–1019
60. Chang, L. & Karin, M. Mammalian MAP kinase signaling cascades. *Nature.* (2001). **410,** 37-40 doi: 10.1038/35065000.
61. Hadiarto, T., Nanmori, T., Matsuoka, D., Iwasaki, T., Sato, K., Fukami, Y., Azuma, T. & Yasuda, T. Activation of Arabidopsis MAPK kinase kinase (AtMEKK1) and induction of AtMEKK1–AtMEK1 pathway by wounding. *Planta.* (2006). **223,** 708–713
62. Teige, M., Scheikl, E., Eulgem, T., Dóczi, R., Ichimura, K., Shinozaki, K., Dangl, J. L. & Hirt, H. The MKK2 pathway mediates cold and salt stress signaling in Arabidopsis. *Mol Cell.* (2004). **15,** 141–152
63. Asai, T., Tena, G., Plotnikova, J., Willmann, M. R., Chiu, W. L., GomezGomez, L., Boller, T., Ausubel, F. M. & Sheen J. MAP kinase signalling cascade in Arabidopsis innate immunity. *Nature.* (2002). **415,** 977–983
64. Liu, Y. & He, C. A review of redox signaling and the control of MAP kinase pathway in plants. *Redox Biology.* (2016). **11,** 192–204
65. Pitzschke, A. & Hirt, H. Mitogen-Activated Protein Kinases and Reactive Oxygen Species Signaling in Plants. *Plant Physiology.* (2006). **141,** 351–356

.

Figure

Click here to access/download;Figure;Paul et al. _TEA MAPKKK _Fig. 1.docx

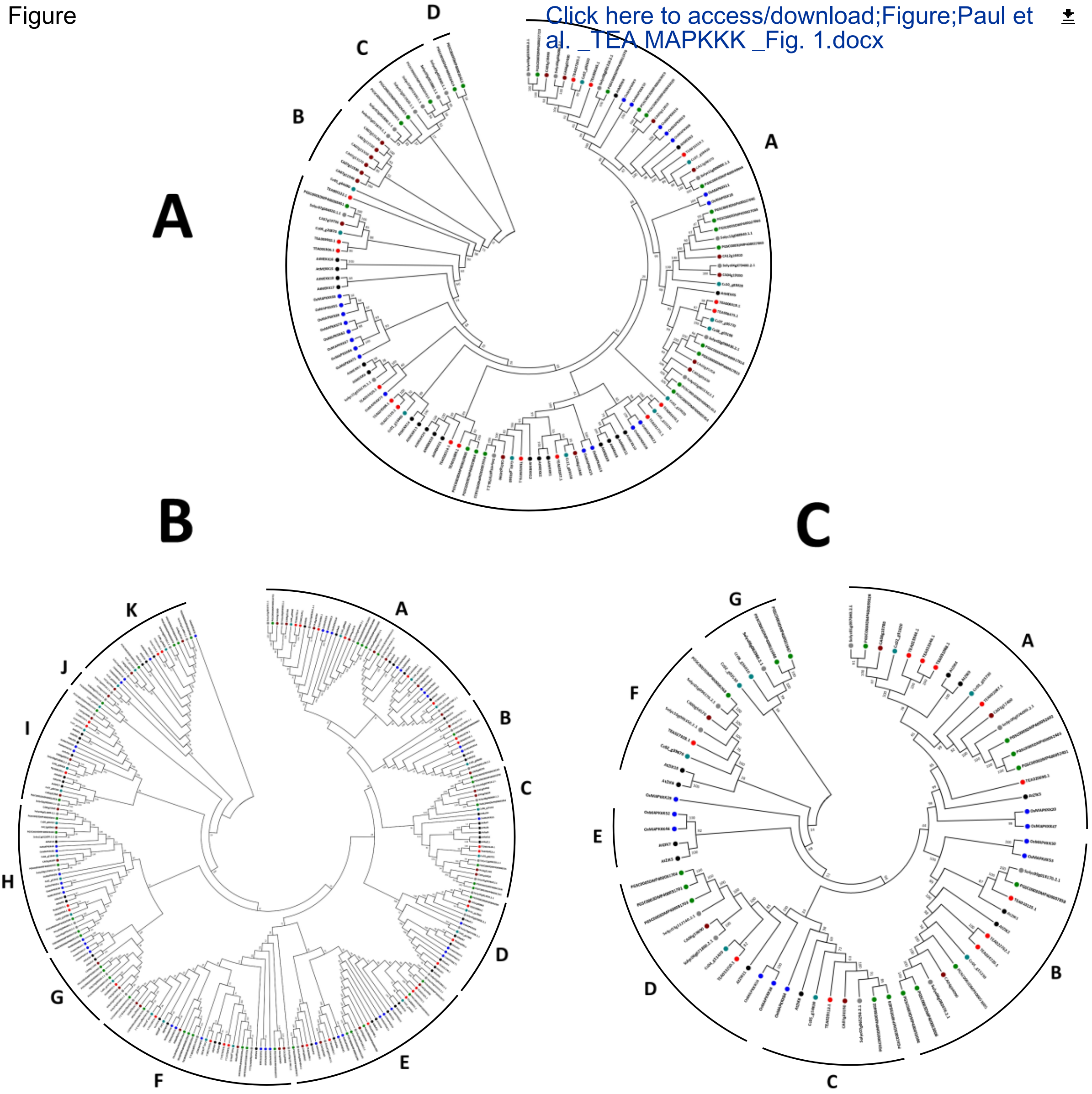

Figure

Click here to access/download;Figure;Paul et al._TEA MAPKKK_Fig. 2.docx

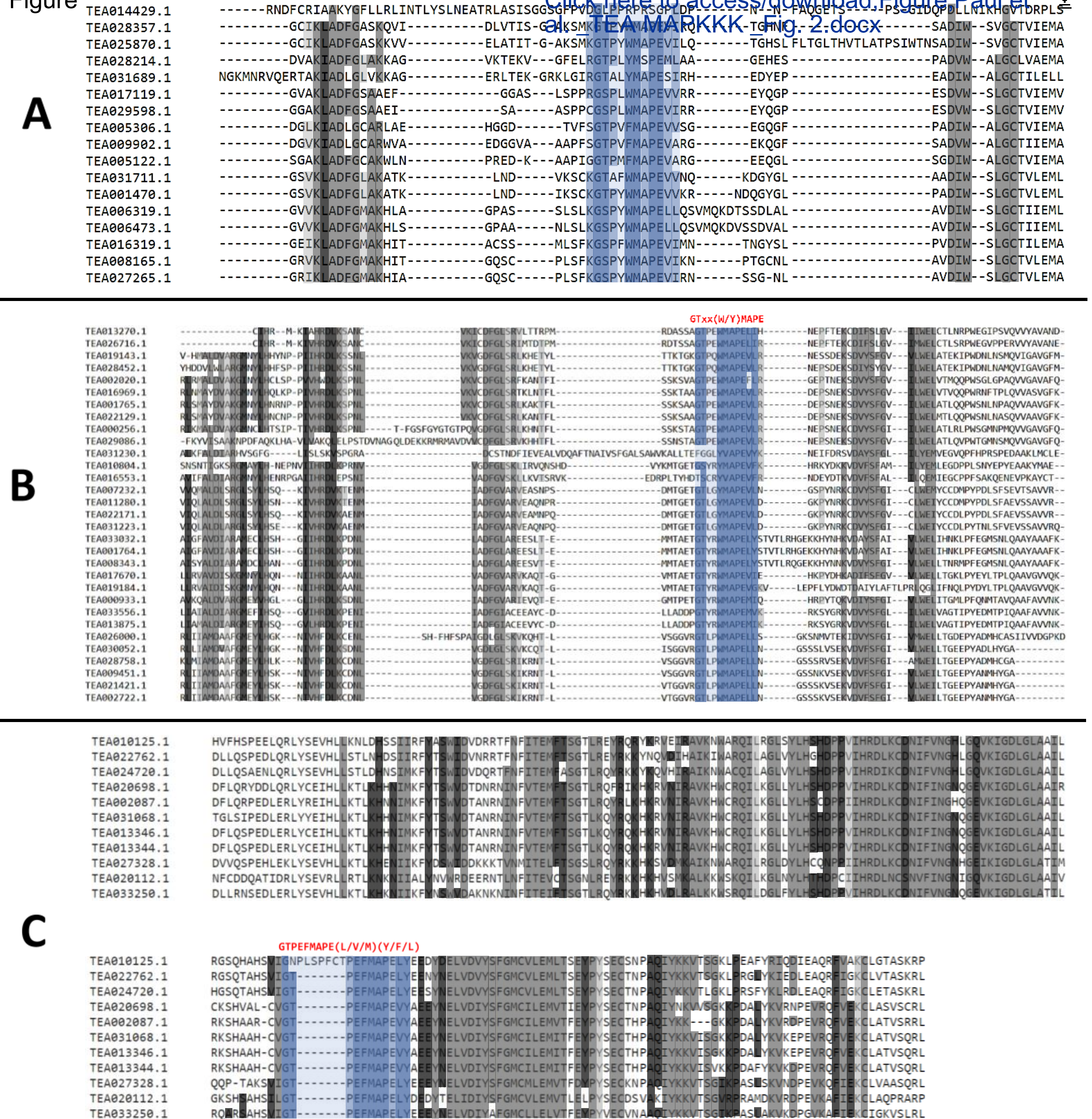

Figure
Click here to access/download;Figure;Paul et al. _TEA MAPKKK _Fig. 3.docx

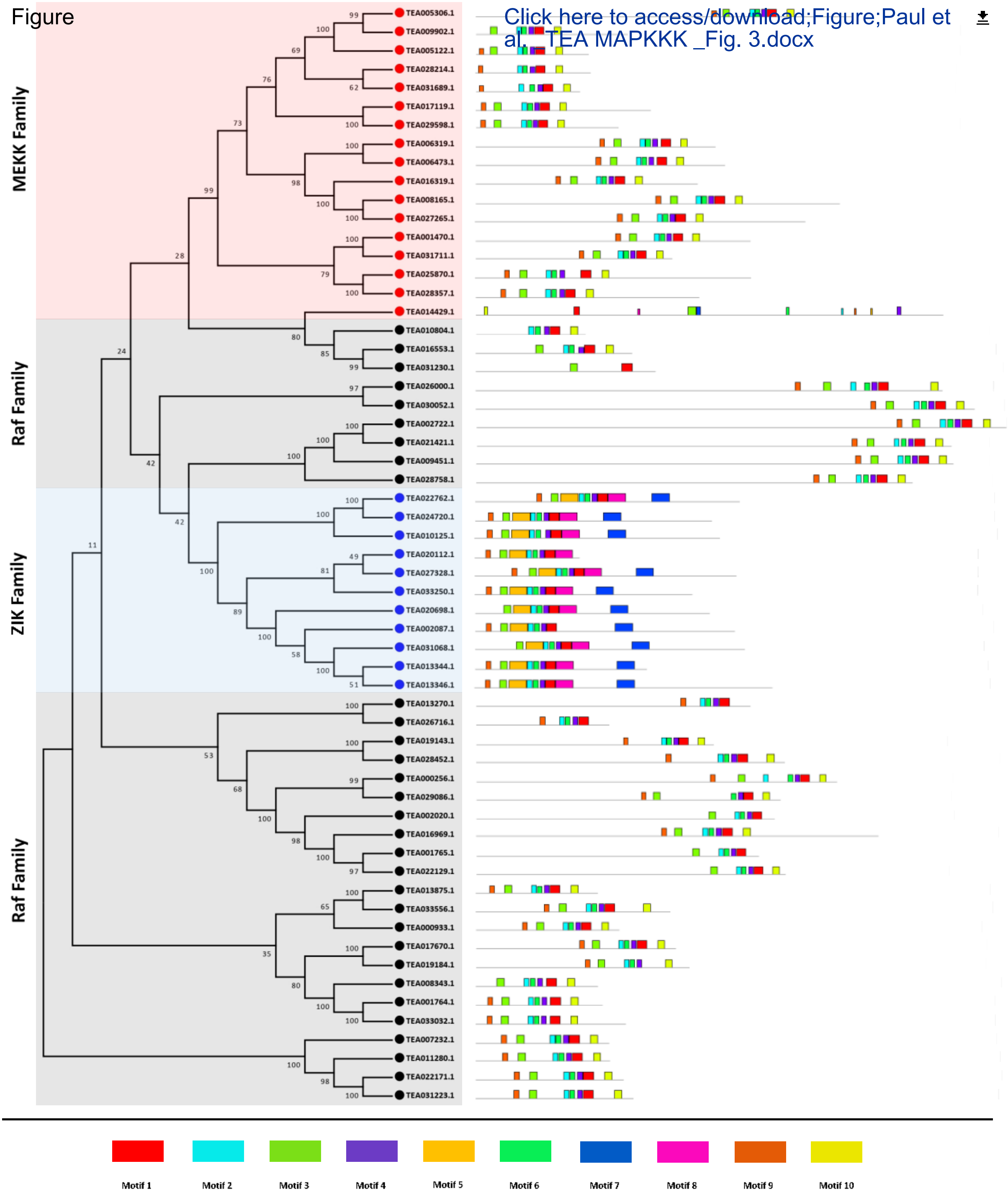

Figure
Click here to access/download;Figure;Paul et al. _TEA MAPKKK _Fig. 4.docx

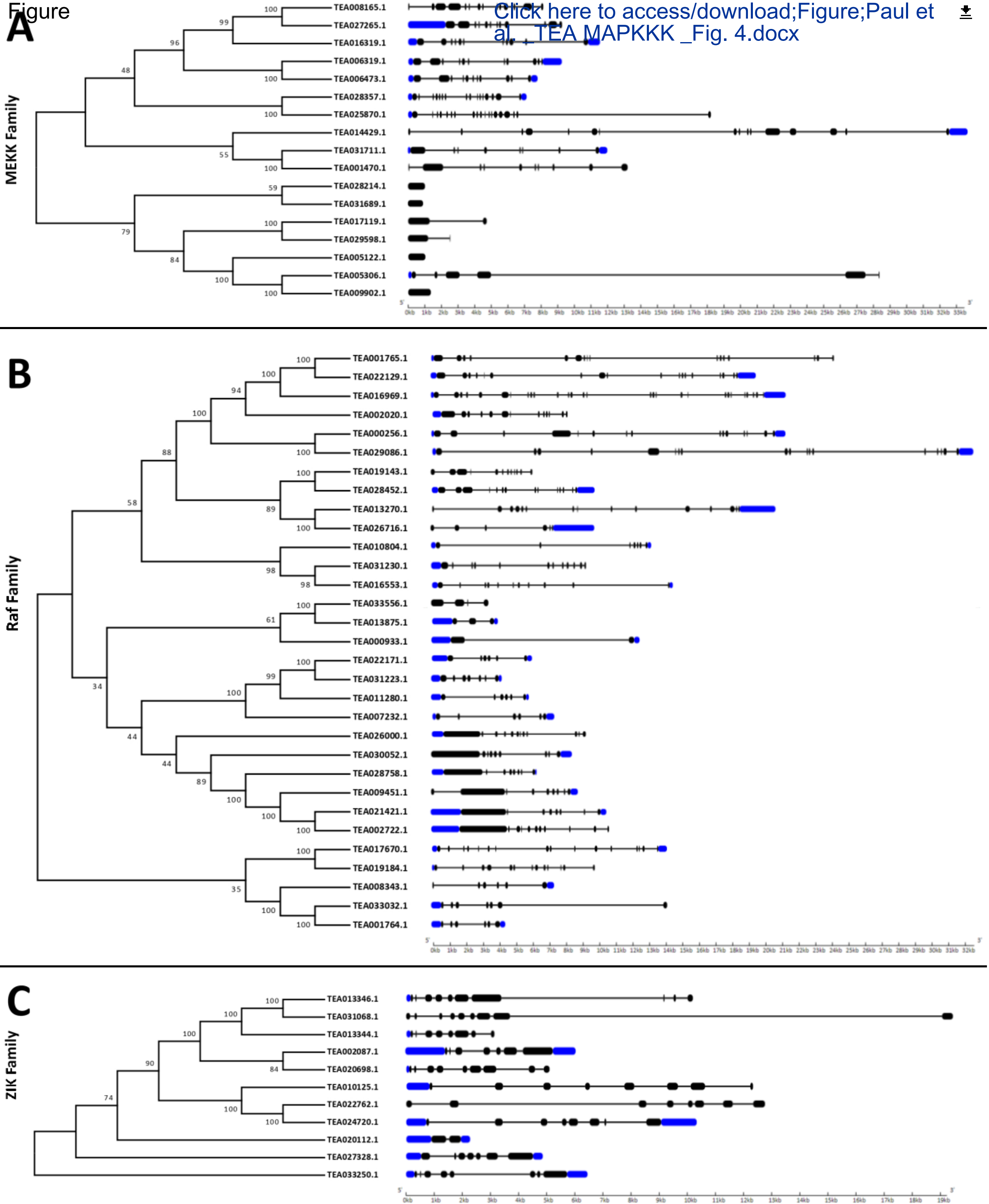

Figure

Click here to access/download;Figure;Paul et al. _TEA MAPKKK _Fig. 5.docx

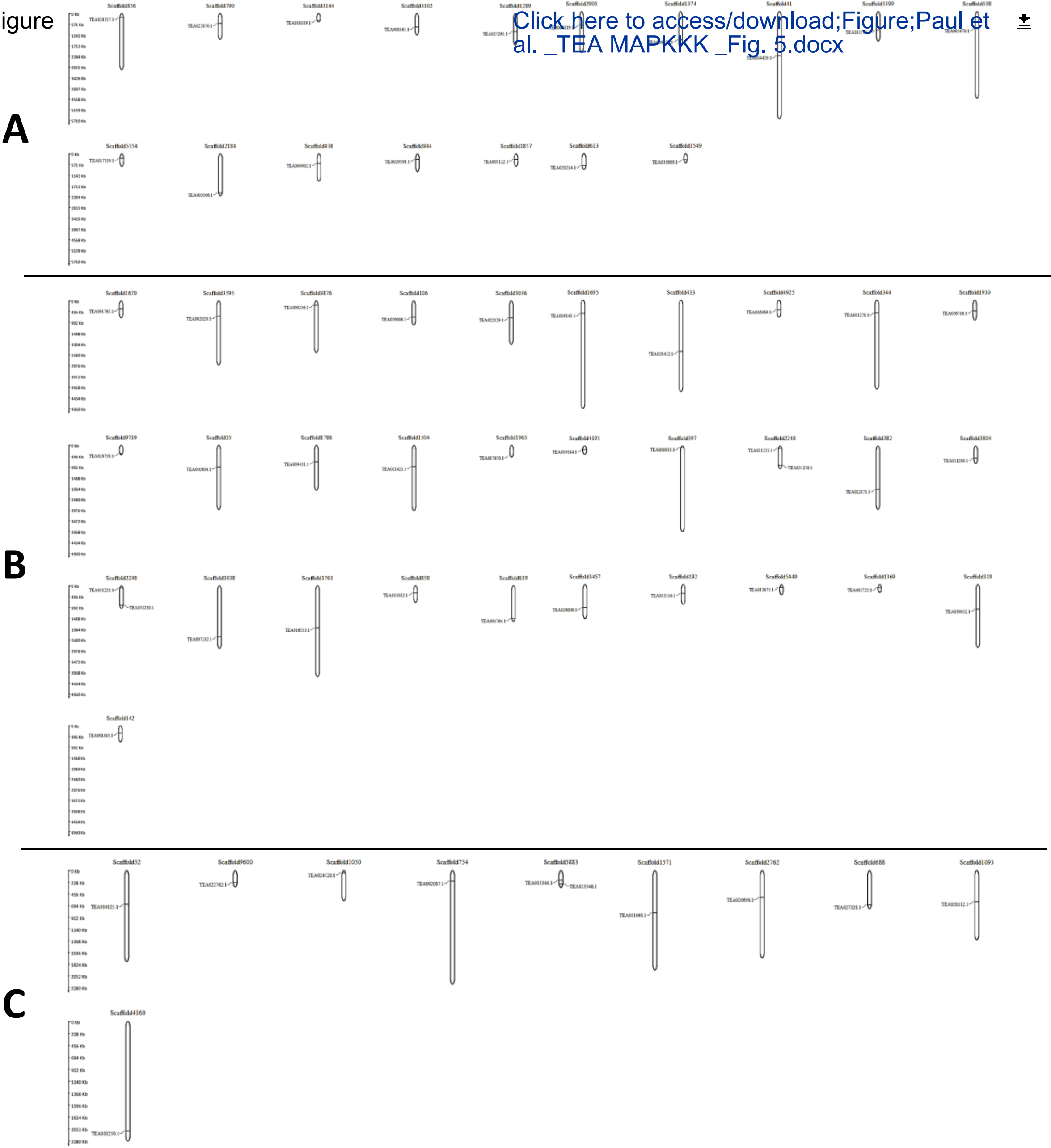

Figure

Click here to access/download;Figure;Paul et al. _TEA MAPKKK _Fig. 6.docx

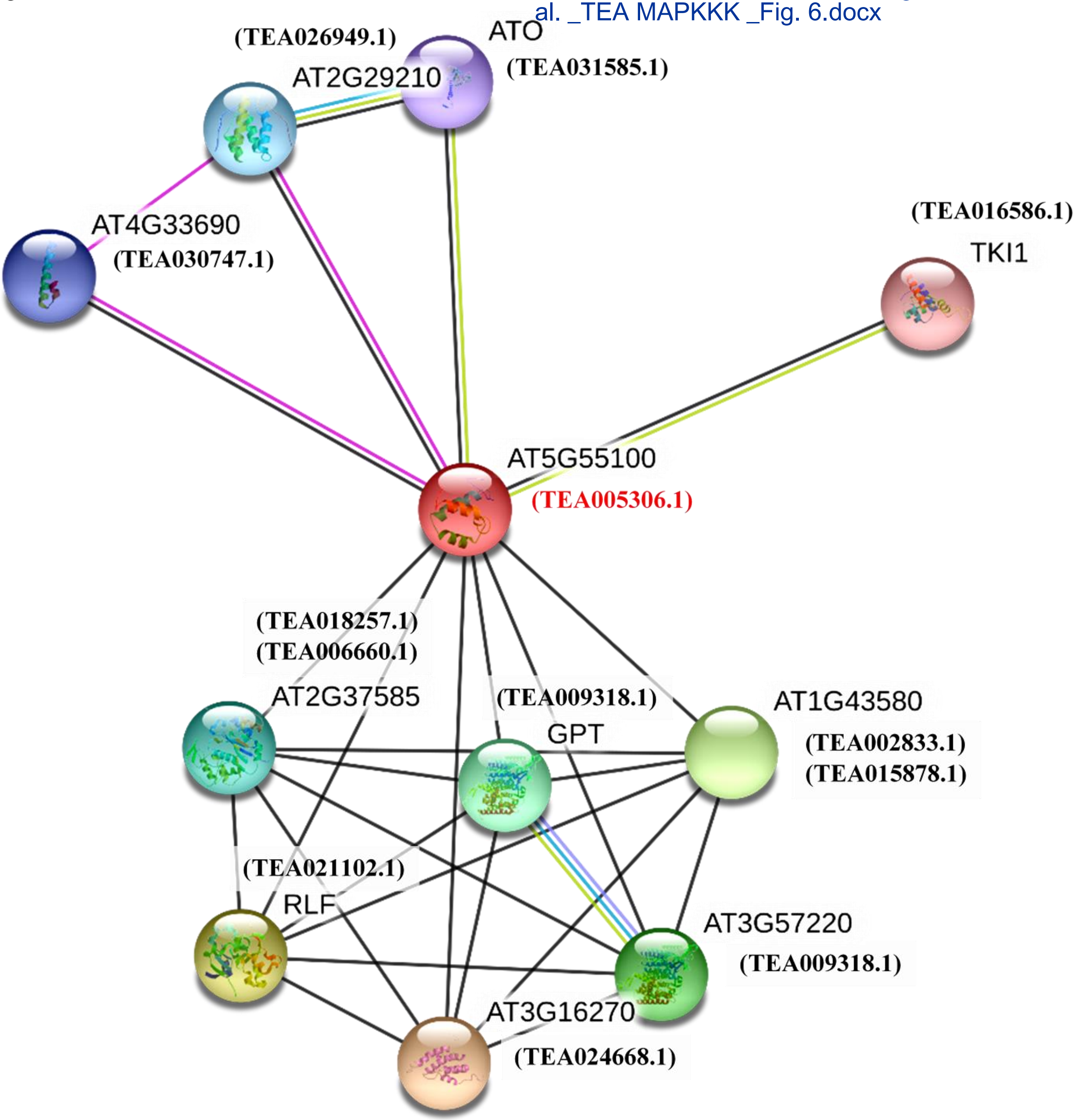

Figure
Click here to access/download;Figure;Paul et al. _TEA MAPKKK _Fig. 7.docx

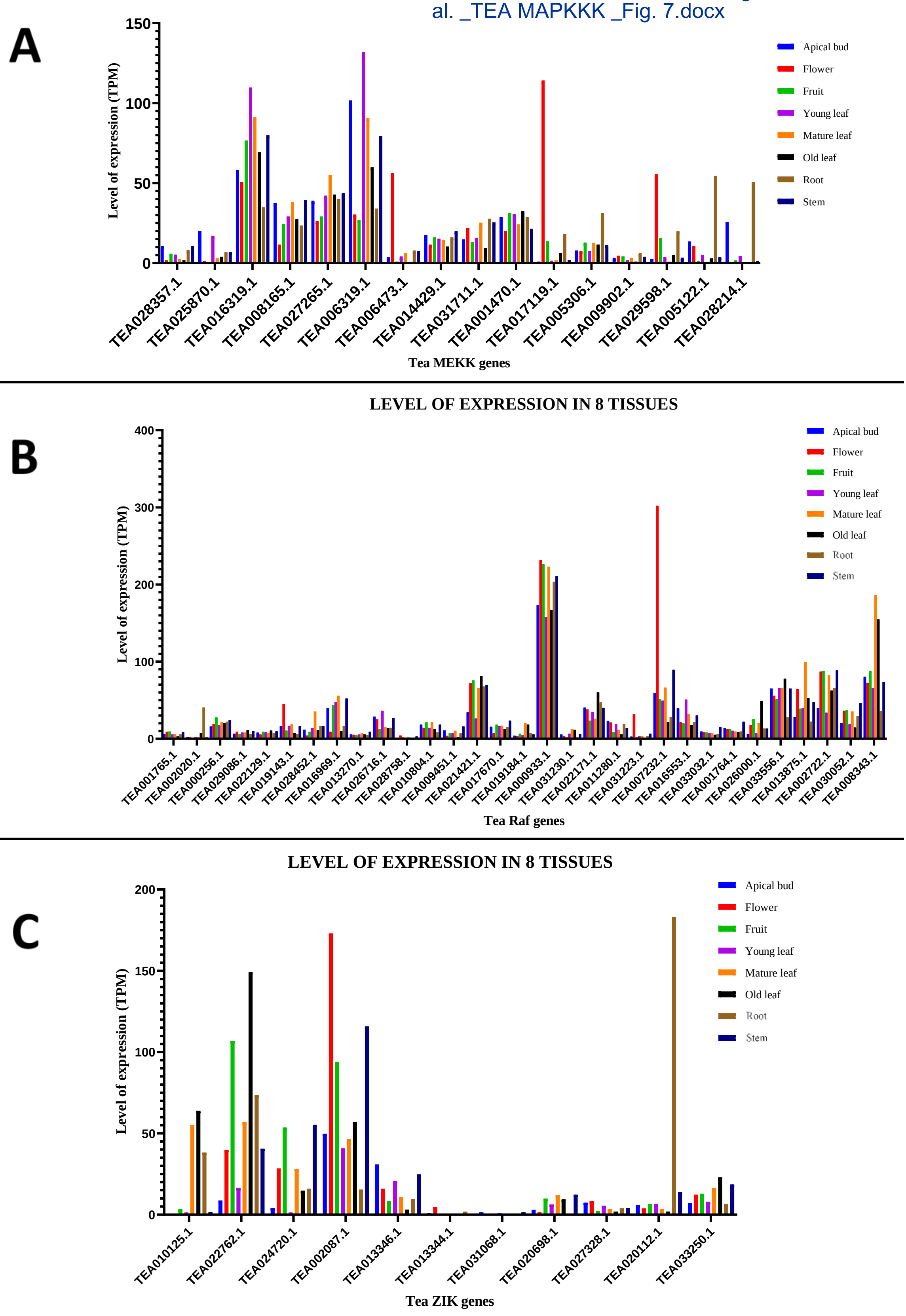

Figure

Click here to access/download;Figure;Paul et al. _TEA MAPKKK _Fig. 8.docx

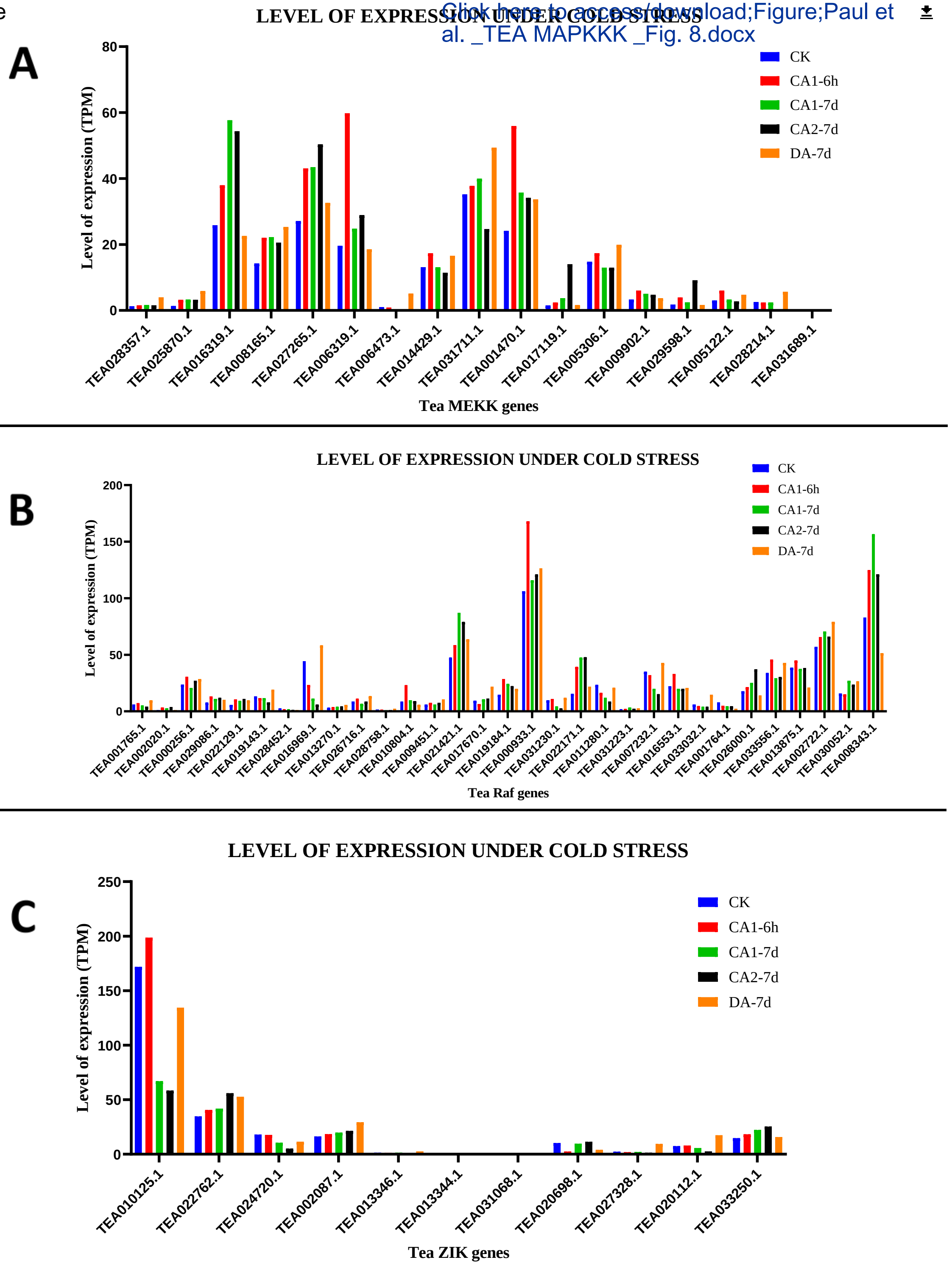

Figure

Click here to access/download;Figure;Paul et al. _TEA MAPKKK _Fig. 9.docx

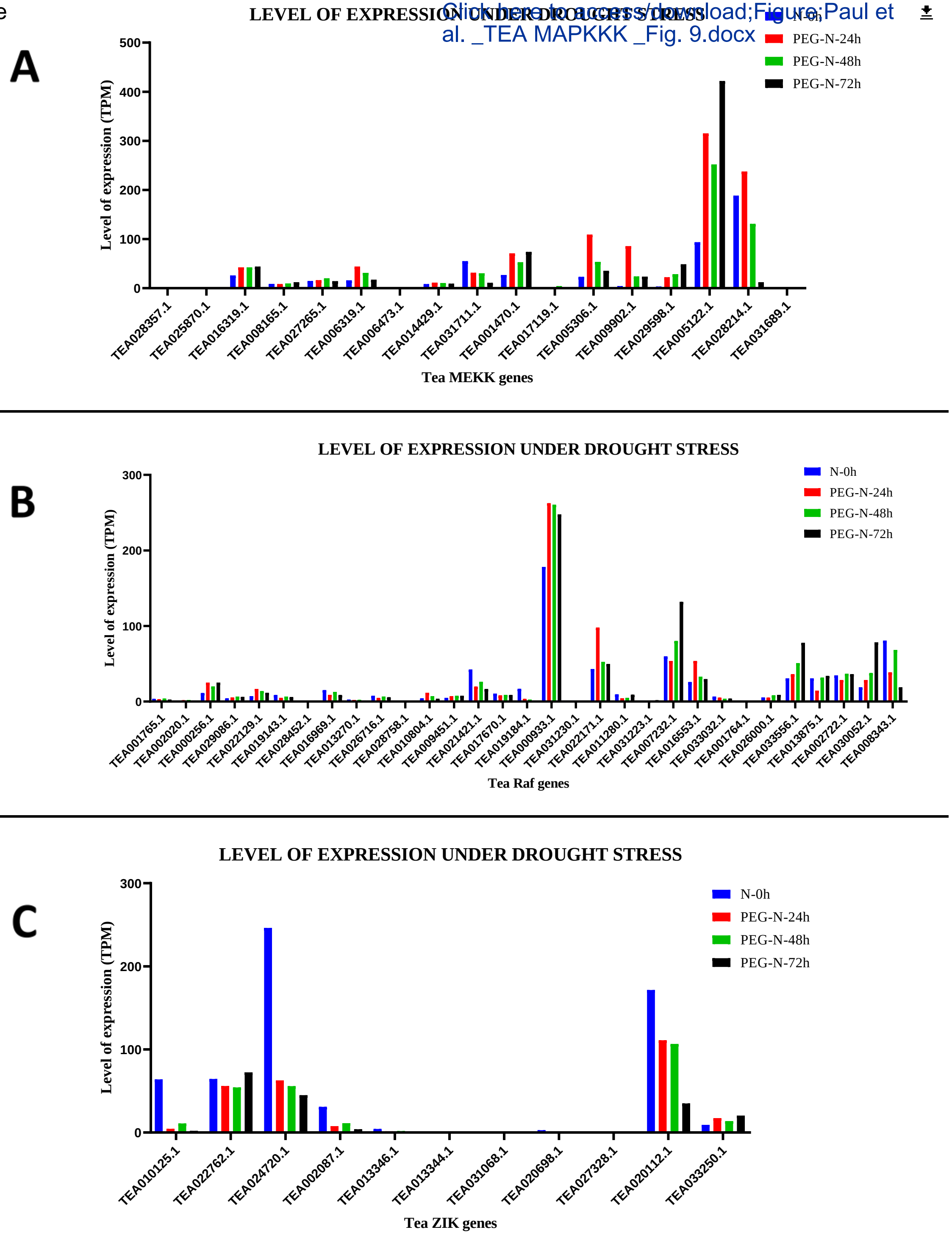

Figure

Click here to access/download;Figure;Paul et al. _TEA MAPKKK _Fig. 10.docx

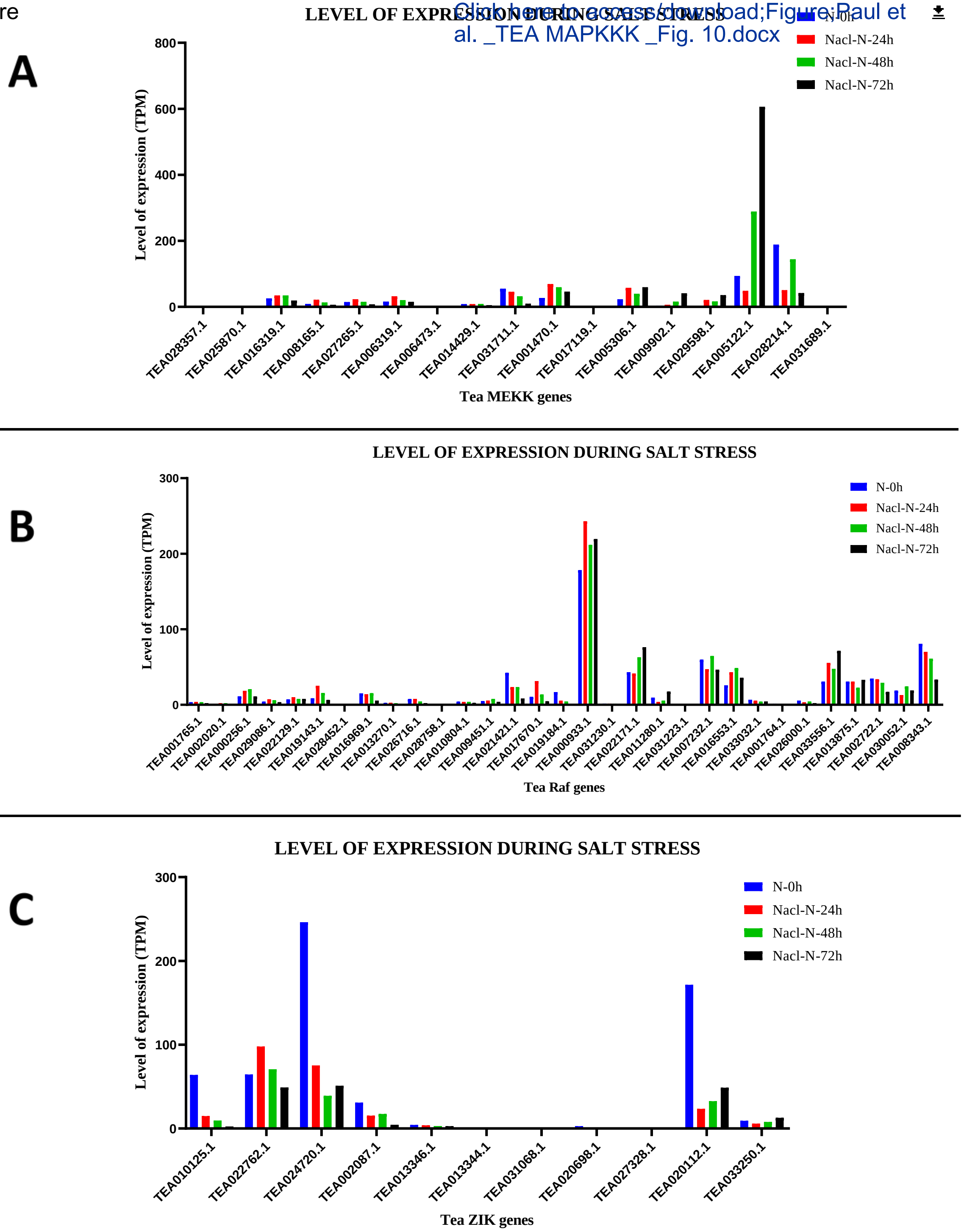

Figure

Click here to access/download;Figure;Paul et al. _TEA MAPKKK _Fig. 11.docx

A

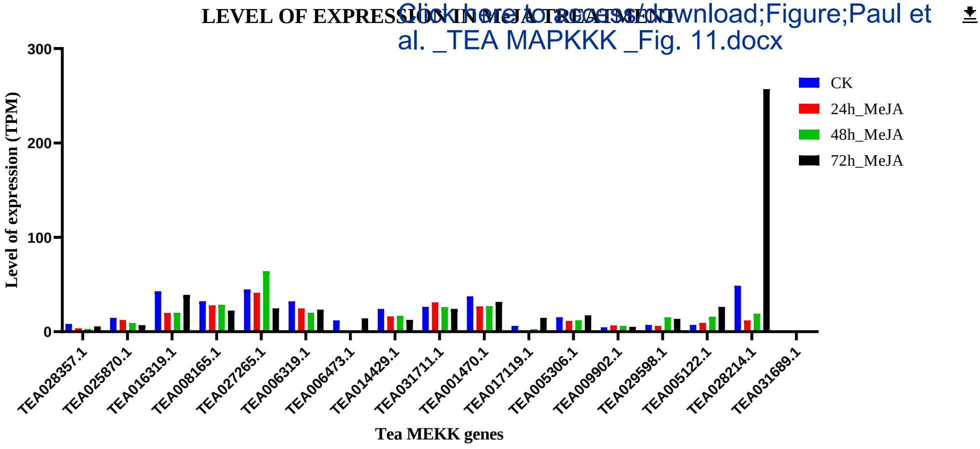

B

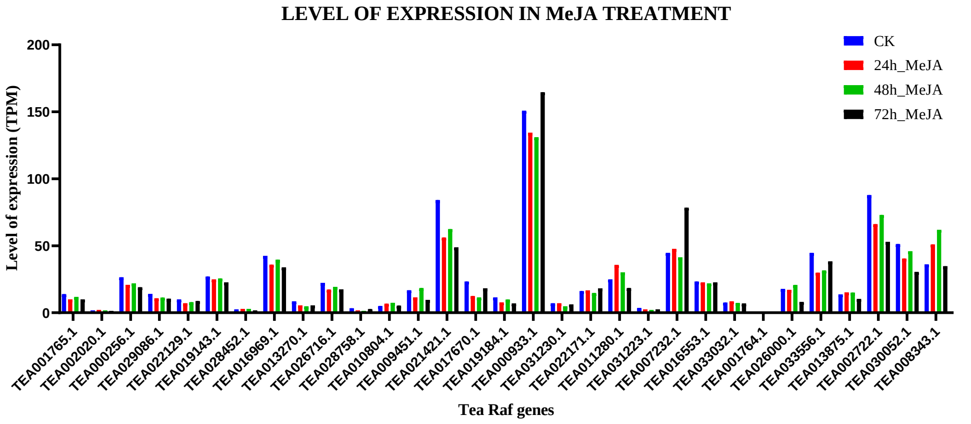

C

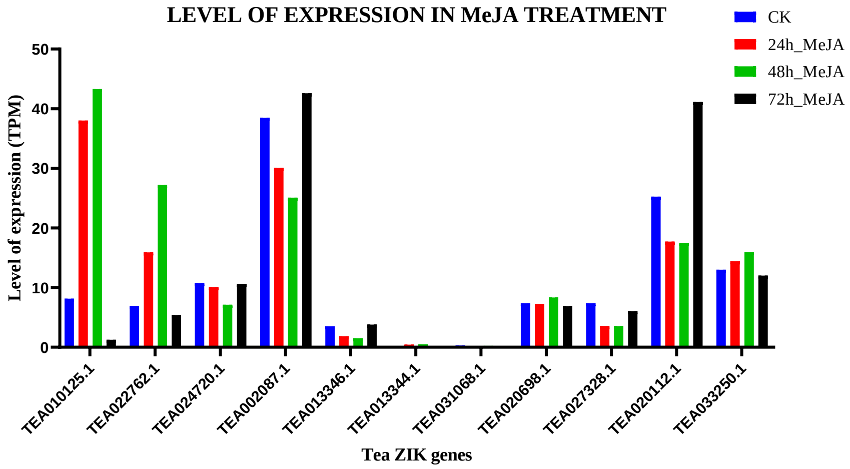

Click here to access/download
Supporting Information
Supporting information_2.pdf

Supporting information

Click here to access/download

Supporting Information

Supporting information_1.pdf
