# Supplementary material for: In-silico genome wide analysis of Mitogen activated protein kinase kinase kinase gene family in C. sinensis
Source: PLoS One. 2021 Nov 4;16(11):e0258657. doi: 10.1371/journal.pone.0258657 (PMC8568164; doi:10.1371/journal.pone.0258657)
Supplement: Attachment — Submitted filename: Supporting information_1.pdf [file pone.0258657.s006.pdf]

In-silico identification, expressional profile and regulatory network analysis of Mitogen Activated Protein Kinase Kinase Kinase gene family in *C. sinensis*

Abhirup Paul[1†], Anurag P. Srivastava[1†], Shreya Subrahmanya[2], Guoxin Shen[3†*], Neelam Mishra[2*]

[1]Department of Life Sciences

Garden City University

Bangalore, Karnataka,

India

[2]Department of Botany

St. Joseph's College autonomous

Bangalore, Karnataka,

India

[3]Sericultural Research Institute,

Zhejiang Academy of Agricultural Sciences

Hangzhou 310021, China

[†]These authors contributed equally to this work.

[*]Corresponding authors:

Guoxin Shen, Ph.D., Professor, Tel: +86-571-86404298; Fax: +86-571-86404298

Email address: guoxin.shen@ttu.edu

Neelam Mishra, Ph.D., Assistant professor

Email address: neelamiitkgp@gmail.com; neelammishra@sjc.ac.in

Supplementary Table S1: BLAST positives table for MEKK genes of C. sinensis.

AtMEKK	Tea MEKK	Identities	Positives	Gaps
AtMEKK1	TEA028357.1	58%	69%	7%
	TEA025870.1	53%	62%	16%
AtMEKK2	TEA028357.1	56%	67%	9%
	TEA025870.1	52%	63%	19%
AtMEKK3	TEA016319.1	57%	67%	10%
AtMEKK4	TEA008165.1	61%	71%	5%
	TEA027265.1	60%	72%	5%
AtMEKK5	TEA006319.1	55%	66%	10%
	TEA006473.1	56%	68%	9%
AtMEKK6	TEA014429.1	60%	71%	12%
AtMEKK7	TEA014429.1	64%	72%	12%
AtMEKK8	TEA031711.1	49%	61%	12%
AtMEKK9	TEA001470.1	55%	68%	7%
AtMEKK10	TEA001470.1	56%	69%	7%
AtMEKK11	TEA031711.1	62%	73%	6%
AtMEKK12	TEA025870.1	55%	66%	12%
	TEA028357.1	54%	66%	10%
AtMEKK13	TEA017119.1	54%	68%	5%
AtMEKK14	TEA017119.1	54%	69%	5%
AtMEKK15	TEA005306.1	51%	66%	6%
AtMEKK16	TEA009902.1	56%	69%	5%
AtMEKK17	TEA029598.1	44%	62%	6%
AtMEKK18	TEA005122.1	54%	66%	8%
AtMEKK19	TEA028214.1	52%	67%	5%
AtMEKK20	TEA028214.1	50%	66%	3%
AtMEKK21	TEA031689.1	43%	61%	10%

Supplementary Table S2: BLAST positives table for Raf genes of C. sinensis.

AtRaf	Tea Raf	Identities	Positives	Gaps
AtRaf1	TEA001765.1	60%	71%	13%
	TEA002020.1	47%	59%	14%
AtRaf2	TEA000256.1	53%	63%	14%
AtRaf3	TEA029086.1	48%	59%	17%
AtRaf4	TEA022129.1	66%	79%	1%
AtRaf5	TEA002020.1	61%	73%	10%
AtRaf6	TEA002020.1	61%	72%	8%
AtRaf7	TEA019143.1	54%	68%	9%
	TEA028452.1	53%	66%	13%
AtRaf8	TEA028452.1	50%	63%	15%
	TEA019143.1	53%	67%	5%
AtRaf9	TEA019143.1	51%	66%	6%
	TEA028452.1	52%	66%	12%
AtRaf10	TEA028452.1	51%	65%	16%
	TEA019143.1	50%	63%	15%
AtRaf11	TEA028452.1	52%	65%	14%
	TEA019143.1	54%	68%	9%
AtRaf12	TEA016969.1	46%	63%	3%
AtRaf13	TEA013270.1	54%	65%	17%
AtRaf14	TEA026716.1	50%	60%	20%
AtRaf15	TEA013270.1	50%	62%	15%
AtRaf16	TEA028758.1	72%	83%	1%

AtRaf17	TEA010804.1	83%	92%	0%
AtRaf18	TEA009451.1	68%	78%	9%
	TEA021421.1	66%	75%	10%
AtRaf19	TEA013789.1	86%	92%	2%
AtRaf20	TEA009451.1	49%	60%	12%
	TEA021421.1	68%	76%	3%
AtRaf21	TEA017670.1	63%	77%	2%
	TEA019184.1	56%	67%	12%
AtRaf22	TEA000933.1	78%	87%	4%
AtRaf23	TEA031230.1	59%	79%	20%
AtRaf24	TEA009451.1	46%	59%	10%
	TEA021421.1	67%	78%	4%
AtRaf25	TEA031230.1	59%	69%	20%
AtRaf26	TEA022171.1	74%	86%	1%
	TEA011280.1	73%	83%	3%
	TEA031223.1	69%	79%	9%
	TEA007232.1	66%	80%	4%
AtRaf27	TEA016553.1	62%	73%	8%
AtRaf28	TEA000933.1	77%	86%	4%
AtRaf29	TEA017670.1	65%	78%	3%
	TEA019184.1	57%	68%	13%
AtRaf30	TEA017670.1	70%	80%	5%
	TEA019184.1	64%	72%	13%
AtRaf31	TEA033032.1	68%	83%	0%

AtRaf32	TEA001764.1	80%	88%	3%
	TEA033032.1	79%	88%	2%
AtRaf33	TEA007232.1	82%	89%	5%
	TEA022171.1	65%	78%	9%
	TEA011280.1	63%	75%	11%
AtRaf34	TEA001764.1	79%	88%	1%
	TEA033032.1	80%	89%	0%
AtRaf35	TEA026000.1	52%	61%	16%
AtRaf36	TEA033556.1	55%	69%	14%
	TEA013875.1	69%	84%	3%
AtRaf37	TEA033032.1	53%	73%	1%
AtRaf38	TEA007232.1	77%	85%	10%
	TEA022171.1	63%	75%	13%
	TEA031223.1	59%	69%	20%
AtRaf39	TEA022171.1	76%	89%	1%
	TEA031223.1	72%	83%	8%
	TEA011280.1	78%	88%	3%
	TEA007232.1	69%	83%	5%
AtRaf40	TEA002722.1	82%	89%	0%
AtRaf41	TEA033032.1	77%	87%	1%
	TEA001764.1	78%	88%	2%
AtRaf42	TEA030052.1	44%	56%	14%
AtRaf43	TEA033556.1	47%	62%	15%
AtRaf44	TEA033032.1	49%	69%	1%

AtRaf45	TEA008343.1	48%	68%	2%
AtRaf46	TEA031230.1	57%	68%	20%
AtRaf47	TEA016553.1	61%	71%	8%
AtRaf48	TEA022171.1	64%	76%	5%
	TEA011280.1	64%	76%	6%

Supplementary Table S3: BLAST positives table for ZIK genes of C. sinensis.

AtZIK	Tea ZIK	Identities	Positives	Gaps
AtZIK1	TEA010125.1	71%	81%	3%
	TEA022762.1	59%	70%	8%
	TEA024720.1	70%	82%	2%
AtZIK2	TEA024720.1	57%	69%	10%
	TEA022762.1	60%	73%	6%
	TEA010125.1	52%	65%	13%
AtZIK3	TEA002087.1	51%	61%	24%
	TEA013346.1	47%	57%	24%
	TEA013344.1	58%	68%	18%
AtZIK4	TEA013346.1	59%	69%	13%
	TEA002087.1	60%	71%	12%
	TEA013344.1	74%	84%	5%
	TEA031068.1	63%	72%	10%
	TEA020698.1	58%	67%	14%
AtZIK5	TEA027328.1	65%	77%	7%

AtZIK6	TEA013344.1	51%	68%	7%
AtZIK7	TEA027328.1	63%	76%	7%
AtZIK8	TEA020112.1	81%	90%	1%
AtZIK9	TEA013346.1	65%	77%	9%
	TEA013344.1	70%	81%	5%
	TEA002087.1	64%	75%	8%
	TEA020698.1	59%	71%	10%
	TEA031068.1	59%	71%	12%
AtZIK10	TEA013344.1	49%	67%	5%
AtZIK11	TEA033250.1	68%	77%	7%

Supplementary Table S4: Ka/Ks ratios of MAPKKKs of MEKK subfamily in C. sinensis.

Compare	Sequence names	Sd	Sn	S	N	ps	pn	ds	dn	dn/ds	ds/dn	ps/pn
0 1	TEA028357.1 TEA025870.1	1055.667	4166.333	1483.5	5761.5	0.7116	0.7231	2.2291	2.4969	1.1201	0.8928	0.9841
0 2	TEA028357.1 TEA016319.1	1069.667	4190.333	1489.667	5755.333	0.7181	0.7281	2.3671	2.6494	1.1192	0.8934	0.9862
0 3	TEA028357.1 TEA008165.1	1132.167	4190.833	1522.333	5722.667	0.7437	0.7323	3.5852	2.8108	0.784	1.2755	1.0155
0 4	TEA028357.1 TEA027265.1	1094.167	4198.833	1478.333	5766.667	0.7401	0.7281	3.2483	2.6509	0.8161	1.2254	1.0165
0 5	TEA028357.1 TEA006319.1	1081	4199	1505	5740	0.7183	0.7315	2.3722	2.7781	1.1711	0.8539	0.9819
0 6	TEA028357.1 TEA006473.1	1079.5	4199.5	1507.833	5737.167	0.7159	0.732	2.3187	2.7965	1.2061	0.8291	0.9781
0 7	TEA028357.1 TEA014429.1	1053.167	4154.833	1487.5	5757.5	0.708	0.7216	2.162	2.4563	1.1361	0.8802	0.9811
0 8	TEA028357.1 TEA031711.1	1127.333	4218.667	1503.167	5741.833	0.75	0.7347	7.6543	2.9204	0.3815	2.621	1.0208
0 9	TEA028357.1 TEA001470.1	1092.333	4238.667	1484.833	5760.167	0.7357	0.7359	2.9678	2.9782	1.0035	0.9965	0.9997
0 10	TEA028357.1 TEA017119.1	682.3333	2761.667	984	3741	0.6934	0.7382	1.9384	3.115	1.6069	0.6223	0.9393
0 11	TEA028357.1 TEA005306.1	1089.333	4188.667	1498.833	5746.167	0.7268	0.7289	2.6065	2.6799	1.0281	0.9726	0.997
0 12	TEA028357.1 TEA009902.1	192.6667	761.3333	293	1057	0.6576	0.7203	1.5702	2.4211	1.5419	0.6485	0.9129
0 13	TEA028357.1 TEA029598.1	401.8333	1503.167	550.5	2044.5	0.7299	0.7352	2.7161	2.9453	1.0844	0.9222	0.9928
0 14	TEA028357.1 TEA005122.1	165.6667	566.3333	229.3333	772.6667	0.7224	0.733	2.4763	2.8384	1.1462	0.8724	0.9856
0 15	TEA028357.1 TEA028214.1	182.1667	592.8333	237.6667	794.3333	0.7665	0.7463	nan	3.9895	nan	nan	1.027
0 16	TEA028357.1 TEA031689.1	154.1667	526.8333	211.1667	721.8333	0.7301	0.7299	2.7209	2.7128	0.997	1.003	1.0003
1 2	TEA025870.1 TEA016319.1	1665.167	6641.833	2349.333	9182.667	0.7088	0.7233	2.1759	2.5016	1.1497	0.8698	0.9799
1 3	TEA025870.1 TEA008165.1	1276.333	4658.667	1683.167	6380.833	0.7583	0.7301	nan	2.7221	nan	nan	1.0386
1 4	TEA025870.1 TEA027265.1	1349.833	5390.167	1898.667	7458.333	0.7109	0.7227	2.2162	2.485	1.1212	0.8918	0.9837
1 5	TEA025870.1 TEA006319.1	1388.333	5358.667	1880.5	7212.5	0.7383	0.743	3.119	3.5023	1.1223	0.8905	0.9937
1 6	TEA025870.1 TEA006473.1	1154.5	4452.5	1600.333	6103.667	0.7214	0.7295	2.4503	2.699	1.1015	0.9079	0.9889
1 7	TEA025870.1 TEA014429.1	2640.5	10600.5	3726.5	14585.5	0.7086	0.7268	2.1721	2.6064	1.1999	0.8334	0.9749
1 8	TEA025870.1 TEA031711.1	1785.333	7181.667	2530.667	9883.333	0.7055	0.7266	2.1181	2.6019	1.2284	0.814	0.9709
1 9	TEA025870.1 TEA001470.1	1862.5	7457.5	2619.167	10280.83	0.7111	0.7254	2.2194	2.5624	1.1545	0.8662	0.9803
1 10	TEA025870.1 TEA017119.1	704.5	2738.5	980.6667	3744.333	0.7184	0.7314	2.3749	2.7715	1.1669	0.8569	0.9822
1 11	TEA025870.1 TEA005306.1	2574	10405	3653.5	14457.5	0.7045	0.7197	2.1023	2.4066	1.1447	0.8735	0.9789
1 12	TEA025870.1 TEA009902.1	201.1667	774.8333	282.3333	1067.667	0.7125	0.7257	2.2471	2.573	1.145	0.8733	0.9818
1 13	TEA025870.1 TEA029598.1	391	1512	536.5	2058.5	0.7288	0.7345	2.6745	2.9102	1.0881	0.919	0.9922
1 14	TEA025870.1 TEA005122.1	161.8333	579.1667	220.1667	781.8333	0.735	0.7408	2.9365	3.299	1.1234	0.8901	0.9923
1 15	TEA025870.1 TEA028214.1	159	584	228.8333	803.1667	0.6948	0.7271	1.9572	2.6174	1.3373	0.7478	0.9556

1 16	TEA025870.1 TEA031689.1	154.6667	537.3333	204	729	0.7582	0.7371	nan	3.0461	nan	nan	1.0286
2 3	TEA016319.1 TEA008165.1	1220.833	4634.167	1690	6374	0.7224	0.727	2.4763	2.6148	1.0559	0.947	0.9936
2 4	TEA016319.1 TEA027265.1	1460	5389	1922.667	7434.333	0.7594	0.7249	nan	2.5473	nan	nan	1.0476
2 5	TEA016319.1 TEA006319.1	1431.167	5236.833	1902.833	7190.167	0.7521	0.7283	nan	2.6582	nan	nan	1.0327
2 6	TEA016319.1 TEA006473.1	1180.333	4479.667	1606.167	6097.833	0.7349	0.7346	2.9278	2.9159	0.9958	1.0041	1.0003
2 7	TEA016319.1 TEA014429.1	1696.333	6646.667	2372.667	9159.333	0.7149	0.7257	2.2974	2.5713	1.1192	0.8935	0.9852
2 8	TEA016319.1 TEA031711.1	1715.5	6657.5	2382.667	9149.333	0.72	0.7276	2.4139	2.6349	1.0915	0.9161	0.9895
2 9	TEA016319.1 TEA001470.1	1716.167	6739.833	2360.667	9171.333	0.727	0.7349	2.6129	2.9281	1.1206	0.8924	0.9893
2 10	TEA016319.1 TEA017119.1	677.8333	2744.167	983	3742	0.6896	0.7333	1.8888	2.8554	1.5117	0.6615	0.9403
2 11	TEA016319.1 TEA005306.1	1691.667	6564.333	2322.167	9008.833	0.7285	0.7287	2.6635	2.6694	1.0022	0.9978	0.9998
2 12	TEA016319.1 TEA009902.1	221.5	780.5	292.8333	1057.167	0.7564	0.7383	nan	3.12	nan	nan	1.0245
2 13	TEA016319.1 TEA029598.1	400.5	1490.5	552.6667	2042.333	0.7247	0.7298	2.541	2.7109	1.0668	0.9373	0.993
2 14	TEA016319.1 TEA005122.1	166	569	221.6667	780.3333	0.7489	0.7292	4.8748	2.688	0.5514	1.8136	1.027
2 15	TEA016319.1 TEA028214.1	169.6667	591.3333	230.8333	801.1667	0.735	0.7381	2.9349	3.107	1.0586	0.9446	0.9958
2 16	TEA016319.1 TEA031689.1	160.8333	548.1667	205	728	0.7846	0.753	nan	nan	nan	nan	1.0419
3 4	TEA008165.1 TEA027265.1	1265	4649	1682.5	6381.5	0.7519	0.7285	nan	2.6644	nan	nan	1.032
3 5	TEA008165.1 TEA006319.1	1283.667	4632.333	1720.5	6343.5	0.7461	0.7302	3.9445	2.7276	0.6915	1.4461	1.0217
3 6	TEA008165.1 TEA006473.1	1197.667	4464.333	1642.667	6061.333	0.7291	0.7365	2.6852	3.0145	1.1226	0.8908	0.9899
3 7	TEA008165.1 TEA014429.1	1232.833	4650.167	1690.5	6373.5	0.7293	0.7296	2.6914	2.7038	1.0046	0.9954	0.9995
3 8	TEA008165.1 TEA031711.1	1231.167	4629.833	1704.333	6359.667	0.7224	0.728	2.476	2.6468	1.0689	0.9355	0.9923
3 9	TEA008165.1 TEA001470.1	1250.5	4663.5	1692.667	6371.333	0.7388	0.732	3.1515	2.7952	0.8869	1.1274	1.0093
3 10	TEA008165.1 TEA017119.1	766.5	2790.5	1009	3716	0.7597	0.7509	nan	nan	nan	nan	1.0116
3 11	TEA008165.1 TEA005306.1	1227.833	4680.167	1700.333	6363.667	0.7221	0.7355	2.4689	2.9569	1.1976	0.835	0.9819
3 12	TEA008165.1 TEA009902.1	200	781	293.8333	1056.167	0.6807	0.7395	1.7858	3.1991	1.7914	0.5582	0.9205
3 13	TEA008165.1 TEA029598.1	437.3333	1513.667	559.1667	2035.833	0.7821	0.7435	nan	3.5626	nan	nan	1.0519
3 14	TEA008165.1 TEA005122.1	177.1667	568.8333	223.6667	778.3333	0.7921	0.7308	nan	2.7502	nan	nan	1.0838
3 15	TEA008165.1 TEA028214.1	180.8333	588.1667	233.3333	798.6667	0.775	0.7364	nan	3.0095	nan	nan	1.0524
3 16	TEA008165.1 TEA031689.1	157.5	537.5	202.6667	730.3333	0.7771	0.736	nan	2.9839	nan	nan	1.0559
4 5	TEA027265.1 TEA006319.1	1397.833	5282.167	1897	7196	0.7369	0.734	3.0336	2.8876	0.9519	1.0506	1.0038
4 6	TEA027265.1 TEA006473.1	1178.167	4492.833	1599.5	6104.5	0.7366	0.736	3.0177	2.9851	0.9892	1.0109	1.0008
4 7	TEA027265.1 TEA014429.1	1337.333	5436.667	1909.5	7447.5	0.7004	0.73	2.0364	2.7182	1.3348	0.7492	0.9594
4 8	TEA027265.1 TEA031711.1	1393.333	5419.667	1935.333	7421.667	0.7199	0.7302	2.4128	2.7277	1.1305	0.8846	0.9859
4 9	TEA027265.1 TEA001470.1	1392.833	5387.167	1912	7445	0.7285	0.7236	2.6629	2.5099	0.9425	1.061	1.0067

4 10	TEA027265.1 TEA017119.1	728.5	2734.5	974	3751	0.7479	0.729	4.4254	2.6819	0.606	1.6501	1.026
4 11	TEA027265.1 TEA005306.1	1391.167	5476.833	1921.833	7435.167	0.7239	0.7366	2.5179	3.0193	1.1991	0.8339	0.9827
4 12	TEA027265.1 TEA009902.1	213.5	780.5	289.5	1060.5	0.7375	0.736	3.0695	2.9843	0.9722	1.0285	1.002
4 13	TEA027265.1 TEA029598.1	381.3333	1499.667	536.1667	2058.833	0.7112	0.7284	2.2217	2.6607	1.1976	0.835	0.9764
4 14	TEA027265.1 TEA005122.1	154.6667	593.3333	221.6667	780.3333	0.6977	0.7604	1.9979	nan	nan	nan	0.9177
4 15	TEA027265.1 TEA028214.1	163.1667	599.8333	230.5	801.5	0.7079	0.7484	2.1597	4.6072	2.1332	0.4688	0.9459
4 16	TEA027265.1 TEA031689.1	136	553	204.3333	728.6667	0.6656	0.7589	1.6382	nan	nan	nan	0.877
5 6	TEA006319.1 TEA006473.1	1187.333	4496.667	1627.5	6076.5	0.7295	0.74	2.7014	3.2388	1.1989	0.8341	0.9859
5 7	TEA006319.1 TEA014429.1	1348.833	5220.167	1891	7202	0.7133	0.7248	2.2628	2.5456	1.1249	0.8889	0.9841
5 8	TEA006319.1 TEA031711.1	1416	5233	1913.833	7179.167	0.7399	0.7289	3.2289	2.6786	0.8296	1.2054	1.015
5 9	TEA006319.1 TEA001470.1	1357.667	5249.333	1892.333	7200.667	0.7175	0.729	2.3531	2.6819	1.1397	0.8774	0.9842
5 10	TEA006319.1 TEA017119.1	703.6667	2742.333	999.8333	3725.167	0.7038	0.7362	2.0901	2.9946	1.4327	0.6979	0.956
5 11	TEA006319.1 TEA005306.1	1381	5322	1902.167	7190.833	0.726	0.7401	2.582	3.2463	1.2572	0.7953	0.981
5 12	TEA006319.1 TEA009902.1	218.6667	815.3333	304.1667	1045.833	0.7189	0.7796	2.3872	nan	nan	nan	0.9221
5 13	TEA006319.1 TEA029598.1	415.3333	1507.667	560.8333	2034.167	0.7406	0.7412	3.2817	3.3316	1.0152	0.985	0.9992
5 14	TEA006319.1 TEA005122.1	170	599	232.5	769.5	0.7312	0.7784	2.764	nan	nan	nan	0.9393
5 15	TEA006319.1 TEA028214.1	173.5	578.5	241.3333	790.6667	0.7189	0.7317	2.3877	2.7833	1.1657	0.8579	0.9826
5 16	TEA006319.1 TEA031689.1	153.8333	558.1667	214.1667	718.8333	0.7183	0.7765	2.3725	nan	nan	nan	0.925
6 7	TEA006473.1 TEA014429.1	1154.667	4462.333	1607.167	6096.833	0.7184	0.7319	2.3763	2.7935	1.1755	0.8507	0.9816
6 8	TEA006473.1 TEA031711.1	1156.167	4423.833	1620	6084	0.7137	0.7271	2.2708	2.6175	1.1526	0.8675	0.9815
6 9	TEA006473.1 TEA001470.1	1173.833	4542.167	1606.667	6097.333	0.7306	0.7449	2.7412	3.7495	1.3678	0.7311	0.9807
6 10	TEA006473.1 TEA017119.1	702.8333	2687.167	992.3333	3732.667	0.7083	0.7199	2.1665	2.4118	1.1132	0.8983	0.9838
6 11	TEA006473.1 TEA005306.1	1191.667	4519.333	1614.833	6089.167	0.738	0.7422	3.0983	3.4237	1.105	0.9049	0.9943
6 12	TEA006473.1 TEA009902.1	221	774	302.1667	1047.833	0.7314	0.7387	2.7721	3.1443	1.1343	0.8816	0.9901
6 13	TEA006473.1 TEA029598.1	407.6667	1525.333	558.8333	2036.167	0.7295	0.7491	2.6996	5.061	1.8747	0.5334	0.9738
6 14	TEA006473.1 TEA005122.1	164.6667	595.3333	231.3333	770.6667	0.7118	0.7725	2.2332	nan	nan	nan	0.9215
6 15	TEA006473.1 TEA028214.1	180.6667	591.3333	241	791	0.7497	0.7476	5.7615	4.3013	0.7465	1.3395	1.0028
6 16	TEA006473.1 TEA031689.1	160.1667	534.8333	214.5	718.5	0.7467	0.7444	4.0691	3.6696	0.9018	1.1089	1.0031
7 8	TEA014429.1 TEA031711.1	1806.5	7228.5	2554.167	9859.833	0.7073	0.7331	2.149	2.8457	1.3242	0.7552	0.9647
7 9	TEA014429.1 TEA001470.1	1846.333	7468.667	2640.667	10259.33	0.6992	0.728	2.019	2.6463	1.3106	0.7629	0.9604
7 10	TEA014429.1 TEA017119.1	692.8333	2747.167	972.6667	3752.333	0.7123	0.7321	2.2429	2.8024	1.2494	0.8003	0.9729
7 11	TEA014429.1 TEA005306.1	4130	15996	5660.667	21996.33	0.7296	0.7272	2.7033	2.6204	0.9693	1.0316	1.0033
7 12	TEA014429.1 TEA009902.1	219.6667	802.3333	287.5	1062.5	0.7641	0.7551	nan	nan	nan	nan	1.0118

7 13	TEA014429.1 TEA029598.1	400.1667	1492.833	541.1667	2053.833	0.7395	0.7269	3.1981	2.6086	0.8157	1.226	1.0173
7 14	TEA014429.1 TEA005122.1	158.3333	582.6667	217.8333	784.1667	0.7269	0.743	2.6087	3.5098	1.3454	0.7433	0.9782
7 15	TEA014429.1 TEA028214.1	158.5	583.5	227.5	804.5	0.6967	0.7253	1.9831	2.5598	1.2908	0.7747	0.9606
7 16	TEA014429.1 TEA031689.1	152	551	197.8333	735.1667	0.7683	0.7495	nan	5.4699	nan	nan	1.0251
8 9	TEA031711.1 TEA001470.1	1790.667	7192.333	2542.833	9871.167	0.7042	0.7286	2.0969	2.6682	1.2724	0.7859	0.9665
8 10	TEA031711.1 TEA017119.1	708.5	2751.5	992.3333	3732.667	0.714	0.7371	2.2769	3.0495	1.3393	0.7466	0.9686
8 11	TEA031711.1 TEA005306.1	1767	7056	2508	9705	0.7045	0.727	2.1025	2.615	1.2437	0.804	0.969
8 12	TEA031711.1 TEA009902.1	216.1667	795.8333	307	1043	0.7041	0.763	2.0956	nan	nan	nan	0.9228
8 13	TEA031711.1 TEA029598.1	408.5	1489.5	554	2041	0.7374	0.7298	3.0627	2.7104	0.8849	1.13	1.0104
8 14	TEA031711.1 TEA005122.1	172.1667	578.8333	236	766	0.7295	0.7557	2.7005	nan	nan	nan	0.9654
8 15	TEA031711.1 TEA028214.1	175.3333	582.6667	246.5	785.5	0.7113	0.7418	2.223	3.385	1.5227	0.6567	0.9589
8 16	TEA031711.1 TEA031689.1	173.8333	535.1667	218	715	0.7974	0.7485	nan	4.6534	nan	nan	1.0654
9 10	TEA001470.1 TEA017119.1	732.8333	2713.167	991.5	3733.5	0.7391	0.7267	3.1746	2.604	0.8203	1.2191	1.0171
9 11	TEA001470.1 TEA005306.1	1861.167	7342.833	2599.333	10099.67	0.716	0.727	2.3207	2.6146	1.1266	0.8876	0.9848
9 12	TEA001470.1 TEA009902.1	225.3333	777.6667	305	1045	0.7388	0.7442	3.153	3.6439	1.1557	0.8653	0.9928
9 13	TEA001470.1 TEA029598.1	426.1667	1519.833	562	2033	0.7583	0.7476	nan	4.3027	nan	nan	1.0143
9 14	TEA001470.1 TEA005122.1	163.6667	588.3333	225.6667	776.3333	0.7253	0.7578	2.5587	nan	nan	nan	0.957
9 15	TEA001470.1 TEA028214.1	178.1667	614.8333	236	796	0.7549	0.7724	nan	nan	nan	nan	0.9774
9 16	TEA001470.1 TEA031689.1	155.5	544.5	206.8333	726.1667	0.7518	0.7498	nan	6.2847	nan	nan	1.0026
10 11	TEA017119.1 TEA005306.1	729.5	2761.5	1000.667	3724.333	0.729	0.7415	2.6822	3.3578	1.2519	0.7988	0.9832
10 12	TEA017119.1 TEA009902.1	208.6667	766.3333	300.5	1049.5	0.6944	0.7302	1.9514	2.7254	1.3966	0.716	0.951
10 13	TEA017119.1 TEA029598.1	394.1667	1452.833	548	2047	0.7193	0.7097	2.3964	2.1935	0.9153	1.0925	1.0134
10 14	TEA017119.1 TEA005122.1	160.6667	535.3333	231	771	0.6955	0.6943	1.9668	1.9506	0.9918	1.0083	1.0017
10 15	TEA017119.1 TEA028214.1	159	573	240.8333	791.1667	0.6602	0.7242	1.5919	2.5286	1.5884	0.6296	0.9116
10 16	TEA017119.1 TEA031689.1	164	537	212.6667	720.3333	0.7712	0.7455	nan	3.835	nan	nan	1.0344
11 12	TEA005306.1 TEA009902.1	219.3333	782.6667	290	1060	0.7563	0.7384	nan	3.1245	nan	nan	1.0243
11 13	TEA005306.1 TEA029598.1	420.3333	1520.667	551	2044	0.7629	0.744	nan	3.617	nan	nan	1.0254
11 14	TEA005306.1 TEA005122.1	161.6667	587.3333	219.8333	782.1667	0.7354	0.7509	2.9546	nan	nan	nan	0.9794
11 15	TEA005306.1 TEA028214.1	181.1667	602.8333	229.3333	802.6667	0.79	0.751	nan	nan	nan	nan	1.0518
11 16	TEA005306.1 TEA031689.1	136.1667	547.8333	203.3333	729.6667	0.6697	0.7508	1.6755	nan	nan	nan	0.8919
12 13	TEA009902.1 TEA029598.1	232.6667	795.3333	310.5	1039.5	0.7493	0.7651	5.2643	nan	nan	nan	0.9794
12 14	TEA009902.1 TEA005122.1	152.1667	470.8333	229.1667	772.8333	0.664	0.6092	1.6243	1.2547	0.7724	1.2946	1.0899
12 15	TEA009902.1 TEA028214.1	179.6667	604.3333	238	794	0.7549	0.7611	nan	nan	nan	nan	0.9918

12 16	TEA009902.1 TEA031689.1	155.1667	510.8333	212.1667	720.8333	0.7313	0.7087	2.7704	2.1739	0.7847	1.2744	1.032
13 14	TEA029598.1 TEA005122.1	179	566	237.8333	764.1667	0.7526	0.7407	nan	3.2906	nan	nan	1.0161
13 15	TEA029598.1 TEA028214.1	182.5	580.5	247.1667	784.8333	0.7384	0.7396	3.1247	3.2121	1.0279	0.9728	0.9983
13 16	TEA029598.1 TEA031689.1	174.6667	549.3333	219.8333	713.1667	0.7945	0.7703	nan	nan	nan	nan	1.0315
14 15	TEA005122.1 TEA028214.1	176.6667	575.3333	231.6667	770.3333	0.7626	0.7469	nan	4.1076	nan	nan	1.0211
14 16	TEA005122.1 TEA031689.1	162.6667	523.3333	213	720	0.7637	0.7269	nan	2.6086	nan	nan	1.0507
15 16	TEA028214.1 TEA031689.1	157	530	211.3333	721.6667	0.7429	0.7344	3.4952	2.9051	0.8312	1.2031	1.0116

Supplementary Table S5: Ka/Ks ratios of MAPKKKs of Raf subfamily in C. sinensis.

Compare	Sequence names	Sd	Sn	S	N	ps	pn	ds	dn	dn/ds	ds/dn	ps/pn
0 1	TEA001765.1 TEA002020.1	1296.667	5003.333	1789.833	6814.167	0.7245	0.7343	2.5349	2.8976	1.1431	0.8748	0.9867
0 2	TEA001765.1 TEA000256.1	3345.167	12874.83	4635.333	17645.67	0.7217	0.7296	2.457	2.7046	1.1001	0.9085	0.9891
0 3	TEA001765.1 TEA029086.1	3879.667	14816.33	5277.833	20195.17	0.7351	0.7337	2.9384	2.8697	0.9766	1.0239	1.0019
0 4	TEA001765.1 TEA022129.1	3298.833	12814.17	4545.833	17633.17	0.7257	0.7267	2.5717	2.604	1.1025	0.9876	0.9986
0 5	TEA001765.1 TEA019143.1	1029.333	4005.667	1400	5443	0.7352	0.7359	2.946	2.982	1.0122	0.9879	0.9991
0 6	TEA001765.1 TEA028452.1	1660.167	6538.833	2310	8895	0.7187	0.7351	2.382	2.9397	1.2341	0.8103	0.9777
0 7	TEA001765.1 TEA016969.1	3499.167	13627.83	4833.167	18836.83	0.724	0.7235	2.5212	2.5063	0.9941	1.006	1.0007
0 8	TEA001765.1 TEA013270.1	3347.167	13028.83	4624.333	18001.67	0.7238	0.7238	2.5162	2.5145	0.9993	1.0007	1.0001
0 9	TEA001765.1 TEA026716.1	1680	6533	2336.5	8910.5	0.719	0.7332	2.3901	2.8481	1.1916	0.8392	0.9807
0 10	TEA001765.1 TEA028758.1	1065.667	4251.333	1497.833	5756.167	0.7115	0.7386	2.2265	3.1379	1.4093	0.7096	0.9633
0 11	TEA001765.1 TEA010804.1	2160.333	8319.667	2940.667	11426.33	0.7346	0.7281	2.9163	2.6506	0.9089	1.1002	1.009
0 12	TEA001765.1 TEA009451.1	1447.833	5565.167	1953.667	7568.333	0.7411	0.7353	3.3243	2.9503	0.8875	1.1267	1.0078
0 13	TEA001765.1 TEA021421.1	1785.667	6922.333	2460.667	9434.333	0.7257	0.7337	2.5717	2.8735	1.1173	0.895	0.989
0 14	TEA001765.1 TEA017670.1	2231.667	9020.333	3203.833	12555.17	0.6966	0.7185	1.9812	2.3765	1.1995	0.8336	0.9695
0 15	TEA001765.1 TEA019184.1	1615.333	6381.667	2260.833	8734.167	0.7145	0.7307	2.2876	2.7432	1.1992	0.8339	0.9779
0 16	TEA001765.1 TEA000933.1	1903.833	7586.167	2668.167	10447.83	0.7135	0.7261	2.2678	2.5846	1.1397	0.8774	0.9827
0 17	TEA001765.1 TEA031230.1	1410.333	5420.667	1920	7392	0.7345	0.7333	2.9118	2.8542	0.9802	1.0202	1.0017
0 18	TEA001765.1 TEA022171.1	1020	3932	1411.5	5422.5	0.7226	0.7251	2.4831	2.5547	1.0288	0.972	0.9966
0 19	TEA001765.1 TEA011280.1	969.5	3700.5	1329.833	5078.167	0.729	0.7287	2.683	2.6713	0.9956	1.0044	1.0005
0 20	TEA001765.1 TEA031223.1	604.3333	2464.667	880.6667	3361.333	0.6862	0.7332	1.8485	2.8508	1.5422	0.6484	0.9359
0 21	TEA001765.1 TEA007232.1	1170.5	4481.5	1631.5	6189.5	0.7174	0.724	2.3527	2.5229	1.0723	0.9325	0.9909
0 22	TEA001765.1 TEA016553.1	2400.5	9287.5	3331	12692	0.7207	0.7318	2.4307	2.7873	1.1467	0.872	0.9848

0 23	TEA001765.1 TEA033032.1	2066.333	8222.667	2910.667	11282.33	0.7099	0.7288	2.1969	2.6749	1.2176	0.8213	0.9741
0 24	TEA001765.1 TEA001764.1	678.8333	2775.167	957.3333	3704.667	0.7091	0.7491	2.1815	5.0443	2.3123	0.4325	0.9466
0 25	TEA001765.1 TEA026000.1	1557	6167	2164.5	8371.5	0.7193	0.7367	2.3977	3.0223	1.2605	0.7933	0.9765
0 26	TEA001765.1 TEA033556.1	536.6667	2015.333	728.5	2709.5	0.7367	0.7438	3.0227	3.597	1.19	0.8404	0.9904
0 27	TEA001765.1 TEA013875.1	652.8333	2519.167	911	3430	0.7166	0.7345	2.3339	2.9071	1.2456	0.8028	0.9757
0 28	TEA001765.1 TEA002722.1	1693	6628	2339.667	9003.333	0.7236	0.7362	2.5102	2.995	1.1931	0.8381	0.9829
0 29	TEA001765.1 TEA030052.1	1292.667	4960.333	1764	6696	0.7328	0.7408	2.8316	3.2999	1.1654	0.8581	0.9892
0 30	TEA001765.1 TEA008343.1	1154.833	4601.167	1611	6240	0.7168	0.7374	2.3391	3.0628	1.3094	0.7637	0.9722
1 2	TEA002020.1 TEA000256.1	1341	4994	1807.167	6796.833	0.742	0.7348	3.4098	2.9218	0.8569	1.167	1.0099
1 3	TEA002020.1 TEA029086.1	1444.5	4973.5	1857.333	6746.667	0.7777	0.7372	nan	3.0517	nan	nan	1.055
1 4	TEA002020.1 TEA022129.1	1303.5	4953.5	1798.333	6805.667	0.7248	0.7278	2.546	2.6417	1.0376	0.9638	0.9959
1 5	TEA002020.1 TEA019143.1	1016.333	4041.667	1410.667	5432.333	0.7205	0.744	2.4258	3.6215	1.4929	0.6698	0.9684
1 6	TEA002020.1 TEA028452.1	1253.333	5005.667	1797.5	6806.5	0.6973	0.7354	1.9911	2.9555	1.4843	0.6737	0.9481
1 7	TEA002020.1 TEA016969.1	1306.833	5004.167	1807.167	6796.833	0.7231	0.7362	2.4971	2.9993	1.2011	0.8326	0.9822
1 8	TEA002020.1 TEA013270.1	1272.333	5035.667	1809.167	6794.833	0.7033	0.7411	2.0818	3.3257	1.5975	0.626	0.949
1 9	TEA002020.1 TEA026716.1	1313.167	4927.833	1786.667	6817.333	0.735	0.7228	2.9331	2.4887	0.8485	1.1786	1.0168
1 10	TEA002020.1 TEA028758.1	1073.833	4242.167	1515.833	5738.167	0.7084	0.7393	2.1692	3.1866	1.469	0.6807	0.9582
1 11	TEA002020.1 TEA010804.1	1280	4983	1789.667	6814.333	0.7152	0.7313	2.3032	2.7668	1.2013	0.8325	0.9781
1 12	TEA002020.1 TEA009451.1	1285.5	5051.5	1790	6814	0.7182	0.7413	2.3694	3.3461	1.4122	0.7081	0.9687
1 13	TEA002020.1 TEA021421.1	1315.167	5104.833	1817.167	6786.833	0.7237	0.7522	2.5142	nan	nan	nan	0.9622
1 14	TEA002020.1 TEA017670.1	1349	5062	1778.5	6825.5	0.7585	0.7416	nan	3.3716	nan	nan	1.0228
1 15	TEA002020.1 TEA019184.1	1272.333	4963.667	1797	6807	0.708	0.7292	2.1624	2.6889	1.2435	0.8042	0.971
1 16	TEA002020.1 TEA000933.1	1317.167	4910.833	1799.833	6804.167	0.7318	0.7217	2.7901	2.4589	0.8813	1.1347	1.014
1 17	TEA002020.1 TEA031230.1	1301.667	4949.333	1801.667	6802.333	0.7225	0.7276	2.4788	2.633	1.0622	0.9414	0.993
1 18	TEA002020.1 TEA022171.1	1028.667	3886.333	1422.167	5411.833	0.7233	0.7181	2.5018	2.3685	0.9467	1.0563	1.0072
1 19	TEA002020.1 TEA011280.1	973.8333	3719.167	1339.833	5068.167	0.7268	0.7338	2.608	2.8776	1.1034	0.9063	0.9905
1 20	TEA002020.1 TEA031223.1	643.6667	2409.333	889.5	3352.5	0.7236	0.7187	2.5108	2.3816	0.9485	1.0543	1.0069
1 21	TEA002020.1 TEA007232.1	1183	4545	1652	6169	0.7161	0.7367	2.3225	3.027	1.3033	0.7673	0.972
1 22	TEA002020.1 TEA016553.1	1294.667	4978.333	1781	6823	0.7269	0.7296	2.6112	2.7049	1.0359	0.9654	0.9963
1 23	TEA002020.1 TEA033032.1	1279.833	4969.167	1772.333	6831.667	0.7221	0.7274	2.4691	2.6257	1.0634	0.9404	0.9928
1 24	TEA002020.1 TEA001764.1	710	2748	969.6667	3692.333	0.7322	0.7442	2.8061	3.6525	1.3016	0.7683	0.9838
1 25	TEA002020.1 TEA026000.1	1292.833	5040.167	1805.5	6798.5	0.7161	0.7414	2.3215	3.3481	1.4422	0.6934	0.9659
1 26	TEA002020.1 TEA033556.1	506	2032	729.5	2708.5	0.6936	0.7502	1.941	nan	nan	nan	0.9245

1 27	TEA002020.1 TEA013875.1	672.8333	2515.167	918.8333	3422.167	0.7323	0.735	2.8086	2.9322	1.044	0.9578	0.9963
1 28	TEA002020.1 TEA002722.1	1318.333	5016.667	1809.833	6794.167	0.7284	0.7384	2.6615	3.1254	1.1743	0.8516	0.9865
1 29	TEA002020.1 TEA030052.1	1308.5	4826.5	1790.333	6669.667	0.7309	0.7236	2.7516	2.5114	0.9127	1.0956	1.01
1 30	TEA002020.1 TEA008343.1	1171	4605	1631.667	6219.333	0.7177	0.7404	2.3581	3.2713	1.3873	0.7208	0.9693
2 3	TEA000256.1 TEA029086.1	3420.167	12815.83	4714.167	17515.83	0.7255	0.7317	2.5663	2.7837	1.0847	0.9219	0.9916
2 4	TEA000256.1 TEA022129.1	3309.167	12861.83	4639.667	17539.33	0.7132	0.7333	2.2616	2.8541	1.262	0.7924	0.9726
2 5	TEA000256.1 TEA019143.1	1003.333	3986.667	1404	5439	0.7146	0.733	2.2905	2.8392	1.2395	0.8068	0.975
2 6	TEA000256.1 TEA028452.1	1725.833	6527.167	2348.667	8856.333	0.7348	0.737	2.9248	3.0417	1.04	0.9616	0.997
2 7	TEA000256.1 TEA016969.1	3296.667	12940.33	4635.167	17645.83	0.7112	0.7333	2.2218	2.8551	1.285	0.7782	0.9699
2 8	TEA000256.1 TEA013270.1	3364.667	12887.33	4646.167	17634.83	0.7242	0.7308	2.5267	2.7484	1.0877	0.9193	0.991
2 9	TEA000256.1 TEA026716.1	1738	6479	2375.333	8871.667	0.7317	0.7303	2.7843	2.7297	0.982	1.02	1.0019
2 10	TEA000256.1 TEA028758.1	1100.5	4228.5	1509.667	5744.333	0.729	0.7361	2.6806	2.992	1.1162	0.8959	0.9903
2 11	TEA000256.1 TEA010804.1	2131	8342	3007.167	11359.83	0.7086	0.7343	2.1733	2.9018	1.3352	0.749	0.965
2 12	TEA000256.1 TEA009451.1	1437.833	5562.167	1980.167	7541.833	0.7261	0.7375	2.5852	3.0713	1.188	0.8417	0.9846
2 13	TEA000256.1 TEA021421.1	1796.167	6922.833	2508.167	9386.833	0.7161	0.7375	2.3231	3.071	1.3219	0.7565	0.971
2 14	TEA000256.1 TEA017670.1	2358	9123	3296.667	12462.33	0.7153	0.732	2.3043	2.7992	1.2148	0.8232	0.9771
2 15	TEA000256.1 TEA019184.1	1645.833	6325.167	2300.667	8694.333	0.7154	0.7275	2.3066	2.6301	1.1402	0.877	0.9833
2 16	TEA000256.1 TEA000933.1	1969.167	7568.833	2715.333	10400.67	0.7252	0.7277	2.557	2.6375	1.0315	0.9695	0.9965
2 17	TEA000256.1 TEA031230.1	1379.833	5412.167	1946.667	7365.333	0.7088	0.7348	2.1766	2.9249	1.3438	0.7442	0.9646
2 18	TEA000256.1 TEA022171.1	1016.167	3952.833	1415	5419	0.7181	0.7294	2.369	2.6975	1.1387	0.8782	0.9845
2 19	TEA000256.1 TEA011280.1	949.6667	3728.333	1336	5072	0.7108	0.7351	2.2141	2.9381	1.327	0.7536	0.967
2 20	TEA000256.1 TEA031223.1	650.5	2444.5	884.8333	3357.167	0.7352	0.7281	2.9424	2.6517	0.9012	1.1096	1.0096
2 21	TEA000256.1 TEA007232.1	1198.833	4475.167	1639.667	6181.333	0.7311	0.724	2.7625	2.5209	0.1925	1.0958	1.0099
2 22	TEA000256.1 TEA016553.1	2552.5	9218.5	3429.833	12593.17	0.7442	0.732	3.6473	2.7983	0.6772	1.3034	1.0166
2 23	TEA000256.1 TEA033032.1	2184.5	8164.5	2976.5	11216.5	0.7339	0.7279	2.8817	2.6434	0.9173	1.0901	1.0083
2 24	TEA000256.1 TEA001764.1	713	2705	964.1667	3697.833	0.7395	0.7315	3.2014	2.7771	0.8675	1.1528	1.0109
2 25	TEA000256.1 TEA026000.1	1587	6122	2198.167	8337.833	0.722	0.7342	2.465	2.8971	1.1753	0.8508	0.9833
2 26	TEA000256.1 TEA033556.1	547.3333	2002.667	729.6667	2708.333	0.7501	0.7394	nan	3.1977	nan	nan	1.0144
2 27	TEA000256.1 TEA013875.1	673.6667	2492.333	914.1667	3426.833	0.7369	0.7273	3.0367	2.6233	0.8639	1.1576	1.0132
2 28	TEA000256.1 TEA002722.1	1749.833	6600.167	2376.667	8966.333	0.7363	0.7361	2.9996	2.9914	0.9973	1.0027	1.0002
2 29	TEA000256.1 TEA030052.1	1306.5	4916.5	1785.667	6674.333	0.7317	0.7366	2.7832	3.0202	1.0851	0.9215	0.9933
2 30	TEA000256.1 TEA008343.1	1193.167	4493.833	1619.833	6231.167	0.7366	0.7212	3.0185	2.4444	0.8098	1.2349	1.0214
3 4	TEA029086.1 TEA022129.1	3412.167	12803.83	4624	17504	0.7379	0.7315	3.0967	2.7759	0.8964	1.1156	1.0088

3 5	TEA029086.1 TEA019143.1	1135.167	3995.833	1459.833	5383.167	0.7776	0.7423	nan	3.4325	nan	nan	1.0476
3 6	TEA029086.1 TEA028452.1	1737	6424	2392.833	8812.167	0.7259	0.729	2.5789	2.6814	1.0397	0.9618	0.9958
3 7	TEA029086.1 TEA016969.1	3568.167	13776.83	4903.333	18715.67	0.7277	0.7361	2.6367	2.9918	1.1347	0.8813	0.9886
3 8	TEA029086.1 TEA013270.1	3462.833	13124.17	4701.5	17873.5	0.7365	0.7343	3.0152	2.8989	0.9614	1.0401	1.0031
3 9	TEA029086.1 TEA026716.1	1822.333	6594.667	2419.667	8827.333	0.7531	0.7471	nan	4.1597	nan	nan	1.0081
3 10	TEA029086.1 TEA028758.1	1178.667	4186.333	1561.167	5692.833	0.755	0.7354	nan	2.9527	nan	nan	1.0267
3 11	TEA029086.1 TEA010804.1	2247.5	8250.5	3032.833	11334.17	0.7411	0.7279	3.3218	2.6445	0.7961	1.2562	1.018
3 12	TEA029086.1 TEA009451.1	1485.833	5556.167	2025.333	7496.667	0.7336	0.7412	2.8682	3.3299	1.161	0.8614	0.9898
3 13	TEA029086.1 TEA021421.1	1938.5	6888.5	2550.667	9344.333	0.76	0.7372	nan	3.0521	nan	nan	1.0309
3 14	TEA029086.1 TEA017670.1	2436.667	9181.333	3297.667	12461.33	0.7389	0.7368	3.1603	3.0291	0.9585	1.0433	1.0029
3 15	TEA029086.1 TEA019184.1	1715.667	6344.333	2342	8653	0.7326	0.7332	2.8212	2.8488	1.0098	0.9903	0.9991
3 16	TEA029086.1 TEA000933.1	2034.833	7547.167	2766.333	10349.67	0.7356	0.7292	2.9631	2.6895	0.9077	1.1017	1.0087
3 17	TEA029086.1 TEA031230.1	1501.167	5375.833	1992	7320	0.7536	0.7344	nan	2.9048	nan	nan	1.0261
3 18	TEA029086.1 TEA022171.1	1124.667	4013.333	1471	5363	0.7646	0.7483	nan	4.5838	nan	nan	1.0217
3 19	TEA029086.1 TEA011280.1	1078.667	3696.333	1391.667	5016.333	0.7751	0.7369	nan	3.0333	nan	nan	1.0519
3 20	TEA029086.1 TEA031223.1	720	2441	914.5	3327.5	0.7873	0.7336	nan	2.8664	nan	nan	1.0732
3 21	TEA029086.1 TEA007232.1	1230.833	4552.167	1700.167	6120.833	0.7239	0.7437	2.52	3.5866	1.4232	0.7026	0.9734
3 22	TEA029086.1 TEA016553.1	2552.667	9318.333	3418.833	12604.17	0.7466	0.7393	4.058	3.1878	0.7855	1.273	1.0099
3 23	TEA029086.1 TEA033032.1	2185.667	8311.333	3001.833	11191.17	0.7281	0.7427	2.6506	3.471	1.3095	0.7636	0.9804
3 24	TEA029086.1 TEA001764.1	762.1667	2747.833	1001.833	3660.167	0.7608	0.7507	nan	nan	nan	nan	1.0134
3 25	TEA029086.1 TEA026000.1	1654.667	6115.333	2243.667	8292.333	0.7375	0.7375	3.0698	3.0689	0.9997	1.0003	1
3 26	TEA029086.1 TEA033556.1	552.6667	2005.333	740.6667	2697.333	0.7462	0.7435	3.9588	3.5555	0.8981	1.1134	1.0037
3 27	TEA029086.1 TEA013875.1	693.5	2522.5	944.8333	3396.167	0.734	0.7427	2.8852	3.4792	1.2058	0.8293	0.9882
3 28	TEA029086.1 TEA002722.1	1773.5	6590.5	2419.5	8923.5	0.733	0.7386	2.8403	3.1369	1.1044	0.9054	0.9925
3 29	TEA029086.1 TEA030052.1	1385.833	4918.167	1834.333	6625.667	0.7555	0.7423	nan	3.4332	nan	nan	1.0178
3 30	TEA029086.1 TEA008343.1	1278.167	4530.833	1679	6172	0.7613	0.7341	nan	2.8901	nan	nan	1.037
4 5	TEA022129.1 TEA019143.1	1006.833	3970.167	1397	5446	0.7207	0.729	2.4321	2.6819	1.1027	0.9069	0.9886
4 6	TEA022129.1 TEA028452.1	1687.333	6449.667	2322.333	8882.667	0.7266	0.7261	2.5995	2.5845	0.9942	1.0058	1.0007
4 7	TEA022129.1 TEA016969.1	3216.5	12888.5	4544	17635	0.7079	0.7308	2.1592	2.7507	1.2739	0.785	0.9685
4 8	TEA022129.1 TEA013270.1	3283.167	12831.83	4554.167	17624.83	0.7209	0.7281	2.4374	2.6486	1.0866	0.9202	0.9902
4 9	TEA022129.1 TEA026716.1	1695.167	6547.833	2349.667	8897.333	0.7214	0.7359	2.4513	2.9821	1.2165	0.822	0.9803
4 10	TEA022129.1 TEA028758.1	1098.167	4268.833	1504	5750	0.7302	0.7424	2.7244	3.4445	1.2643	0.7909	0.9835
4 11	TEA022129.1 TEA010804.1	2049.667	8354.333	2970.833	11396.17	0.6899	0.7331	1.8934	2.8438	1.5019	0.6658	0.9411

4	12	TEA022129.1 TEA009451.1	1410	5616	1964.667	7557.333	0.7177	0.7431	2.3583	3.5185	1.492	0.6702	0.9658
4	13	TEA022129.1 TEA021421.1	1795.5	6947.5	2475.5	9419.5	0.7253	0.7376	2.5602	3.0747	1.201	0.8327	0.9834
4	14	TEA022129.1 TEA017670.1	2312.167	9132.833	3239.5	12519.5	0.7137	0.7295	2.2721	2.6993	1.188	0.8417	0.9784
4	15	TEA022129.1 TEA019184.1	1648.333	6355.667	2272.5	8722.5	0.7253	0.7287	2.5611	2.6693	1.0422	0.9595	0.9955
4	16	TEA022129.1 TEA000933.1	1931.333	7662.667	2697	10419	0.7161	0.7355	2.3226	2.9569	1.273	0.7855	0.9737
4	17	TEA022129.1 TEA031230.1	1394.833	5344.167	1927	7385	0.7238	0.7237	2.5168	2.5115	0.9979	1.0021	1.0003
4	18	TEA022129.1 TEA022171.1	971.6667	3935.333	1408.167	5425.833	0.69	0.7253	1.8946	2.5598	1.3511	0.7401	0.9514
4	19	TEA022129.1 TEA011280.1	936.3333	3693.667	1329.167	5078.833	0.7045	0.7273	2.101	2.6222	1.2481	0.8012	0.9686
4	20	TEA022129.1 TEA031223.1	623.8333	2459.167	874.1667	3367.833	0.7136	0.7302	2.2698	2.7255	1.2008	0.8328	0.9773
4	21	TEA022129.1 TEA007232.1	1182.333	4458.667	1634.167	6186.833	0.7235	0.7207	2.5074	2.4311	0.9696	1.0314	1.0039
4	22	TEA022129.1 TEA016553.1	2494	9214	3367.333	12655.67	0.7406	0.7281	3.2882	2.6486	0.8055	1.2415	1.0173
4	23	TEA022129.1 TEA033032.1	2093	8163	2941.833	11251.17	0.7115	0.7255	2.2263	2.5668	1.1529	0.8673	0.9806
4	24	TEA022129.1 TEA001764.1	689.6667	2710.333	955.1667	3706.833	0.722	0.7312	2.4669	2.7636	1.1203	0.8927	0.9875
4	25	TEA022129.1 TEA026000.1	1533.333	6209.667	2176.167	8359.833	0.7046	0.7428	2.1035	3.4843	1.6564	0.6037	0.9486
4	26	TEA022129.1 TEA033556.1	524.1667	2009.833	716.8333	2721.167	0.7312	0.7386	2.7657	3.1394	1.1351	0.881	0.99
4	27	TEA022129.1 TEA013875.1	679.8333	2533.167	903.8333	3437.167	0.7522	0.737	nan	3.0409	nan	nan	1.0206
4	28	TEA022129.1 TEA002722.1	1748.167	6639.833	2350.667	8992.333	0.7437	0.7384	3.5834	3.126	0.8723	1.1463	1.0072
4	29	TEA022129.1 TEA030052.1	1312	4843	1775	6685	0.7392	0.7245	3.1773	2.5348	0.7978	1.2535	1.0203
4	30	TEA022129.1 TEA008343.1	1149.5	4519.5	1614	6237	0.7122	0.7246	2.2409	2.5398	1.1334	0.8823	0.9829
5	6	TEA019143.1 TEA028452.1	1009.333	3966.667	1395	5448	0.7235	0.7281	2.5082	2.6501	1.0566	0.9465	0.9937
5	7	TEA019143.1 TEA016969.1	1013.333	3980.667	1398.167	5444.833	0.7248	0.7311	2.5437	2.7603	1.0851	0.9215	0.9913
5	8	TEA019143.1 TEA013270.1	1014.5	3926.5	1408.667	5434.333	0.7202	0.7225	2.4188	2.4804	1.0255	0.9752	0.9967
5	9	TEA019143.1 TEA026716.1	993.6667	3983.333	1386.333	5456.667	0.7168	0.73	2.3372	2.718	1.1629	0.8599	0.9819
5	10	TEA019143.1 TEA028758.1	1028.167	3993.833	1399.333	5443.667	0.7348	0.7337	2.9218	2.8701	0.9823	1.018	1.0015
5	11	TEA019143.1 TEA010804.1	978.6667	4013.333	1394	5449	0.7021	0.7365	2.0625	3.0145	1.4616	0.6842	0.9532
5	12	TEA019143.1 TEA009451.1	1052.833	4026.167	1389.667	5453.333	0.7576	0.7383	nan	3.12	nan	nan	1.0262
5	13	TEA019143.1 TEA021421.1	1020.167	3983.833	1412	5431	0.7225	0.7335	2.4794	2.8642	1.1552	0.8656	0.985
5	14	TEA019143.1 TEA017670.1	1025.333	4026.667	1383	5460	0.7414	0.7375	3.3498	3.0698	0.9164	1.0912	1.0053
5	15	TEA019143.1 TEA019184.1	1003.333	3956.667	1391.5	5451.5	0.721	0.7258	2.4407	2.5751	1.0551	0.9478	0.9935
5	16	TEA019143.1 TEA000933.1	1028.833	4048.167	1401.333	5441.667	0.7342	0.7439	2.8942	3.6113	1.2478	0.8014	0.9869
5	17	TEA019143.1 TEA031230.1	994.8333	4025.167	1397.667	5445.333	0.7118	0.7392	2.2326	3.1801	1.4244	0.702	0.9629
5	18	TEA019143.1 TEA022171.1	983.5	4066.5	1388.5	5445.5	0.7083	0.7468	2.1675	4.0842	1.8823	0.5307	0.9485
5	19	TEA019143.1 TEA011280.1	956.5	3711.5	1307	5101	0.7318	0.7276	2.7902	2.6333	0.9438	1.0596	1.0058

5 20	TEA019143.1 TEA031223.1	637.1667	2436.833	853	3389	0.747	0.719	4.134	2.3906	0.5783	1.7293	1.0388
5 21	TEA019143.1 TEA007232.1	1057	3943	1411	5432	0.7491	0.7259	5.0559	2.5779	0.5099	1.9613	1.032
5 22	TEA019143.1 TEA016553.1	1007.5	3979.5	1384.5	5458.5	0.7277	0.729	2.6366	2.6833	1.0177	0.9826	0.9982
5 23	TEA019143.1 TEA033032.1	994.5	3998.5	1370.833	5472.167	0.7255	0.7307	2.5652	2.7449	1.07	0.9345	0.9928
5 24	TEA019143.1 TEA001764.1	668.5	2729.5	935.5	3726.5	0.7146	0.7325	2.2898	2.8166	1.2301	0.813	0.9756
5 25	TEA019143.1 TEA026000.1	1037.667	3971.333	1411.333	5431.667	0.7352	0.7311	2.946	2.7625	0.9377	1.0665	1.0056
5 26	TEA019143.1 TEA033556.1	527.1667	2040.833	708.1667	2729.833	0.7444	0.7476	3.6744	4.3096	1.1729	0.8526	0.9957
5 27	TEA019143.1 TEA013875.1	651.6667	2561.333	883.1667	3457.833	0.7379	0.7407	3.0936	3.2953	1.0652	0.9388	0.9961
5 28	TEA019143.1 TEA002722.1	1029	4021	1397	5446	0.7366	0.7383	3.0174	3.1229	1.035	0.9662	0.9976
5 29	TEA019143.1 TEA030052.1	1044.167	3974.833	1415.833	5427.167	0.7375	0.7324	3.0703	2.8139	0.9165	1.0911	1.007
5 30	TEA019143.1 TEA008343.1	1008.167	3970.833	1384.667	5458.333	0.7281	0.7275	2.65	2.6293	0.9923	1.0079	1.0008
6 7	TEA028452.1 TEA016969.1	1710.333	6485.667	2335	8870	0.7325	0.7312	2.8174	2.7643	0.9811	1.0192	1.0018
6 8	TEA028452.1 TEA013270.1	1674.167	6475.833	2332.833	8872.167	0.7177	0.7299	2.3577	2.7147	1.1514	0.8685	0.9832
6 9	TEA028452.1 TEA026716.1	1734.333	6507.667	2354	8851	0.7368	0.7352	3.0276	2.9464	0.9732	1.0276	1.0021
6 10	TEA028452.1 TEA028758.1	1095.667	4209.333	1502.667	5751.333	0.7291	0.7319	2.687	2.7926	1.0393	0.9622	0.9963
6 11	TEA028452.1 TEA010804.1	1687.833	6458.167	2332.5	8872.5	0.7236	0.7279	2.5105	2.6429	1.0527	0.9499	0.9941
6 12	TEA028452.1 TEA009451.1	1484.333	5559.667	1966.5	7555.5	0.7548	0.7358	nan	2.9774	nan	nan	1.0258
6 13	TEA028452.1 TEA021421.1	1685.5	6561.5	2349.667	8855.333	0.7173	0.741	2.3503	3.3143	1.4102	0.7092	0.9681
6 14	TEA028452.1 TEA017670.1	1693.5	6500.5	2311	8894	0.7328	0.7309	2.8314	2.7522	0.972	1.0287	1.0026
6 15	TEA028452.1 TEA019184.1	1646.333	6336.667	2285.5	8709.5	0.7203	0.7276	2.4227	2.6319	1.0863	0.9205	0.9901
6 16	TEA028452.1 TEA000933.1	1647.5	6569.5	2317.667	8887.333	0.7108	0.7392	2.2144	3.1803	1.4362	0.6963	0.9616
6 17	TEA028452.1 TEA031230.1	1369.667	5423.333	1929.333	7382.667	0.7099	0.7346	2.1968	2.9145	1.3267	0.7538	0.9664
6 18	TEA028452.1 TEA022171.1	1007.833	3955.167	1406.167	5427.833	0.7167	0.7287	2.3364	2.6704	1.1429	0.8749	0.9836
6 19	TEA028452.1 TEA011280.1	974.3333	3680.667	1323.5	5084.5	0.7362	0.7239	2.9954	2.5186	0.8408	1.1893	1.017
6 20	TEA028452.1 TEA031223.1	655.6667	2468.333	882.6667	3359.333	0.7428	0.7348	3.4871	2.9225	0.8381	1.1932	1.011
6 21	TEA028452.1 TEA007232.1	1179.333	4555.667	1634.5	6186.5	0.7215	0.7364	2.4533	3.0069	1.2256	0.8159	0.9798
6 22	TEA028452.1 TEA016553.1	1689.167	6477.833	2333.667	8871.333	0.7238	0.7302	2.5165	2.7257	1.0831	0.9232	0.9913
6 23	TEA028452.1 TEA033032.1	1669.833	6501.167	2318	8887	0.7204	0.7315	2.4236	2.7782	1.1463	0.8724	0.9847
6 24	TEA028452.1 TEA001764.1	711.5	2712.5	957.6667	3704.333	0.743	0.7323	3.5005	2.8078	0.8024	1.2467	1.0146
6 25	TEA028452.1 TEA026000.1	1516.333	6097.667	2186	8350	0.6937	0.7303	1.9415	2.728	1.4051	0.7117	0.9499
6 26	TEA028452.1 TEA033556.1	521.8333	2006.167	725	2713	0.7198	0.7395	2.4084	3.199	1.3283	0.7529	0.9734
6 27	TEA028452.1 TEA013875.1	676.5	2485.5	910.5	3430.5	0.743	0.7245	3.5054	2.5369	0.7237	1.3818	1.0255
6 28	TEA028452.1 TEA002722.1	1693.167	6492.833	2336.833	8868.167	0.7246	0.7322	2.5377	2.8036	1.1048	0.9052	0.9896

6 29	TEA028452.1 TEA030052.1	1297.833	4905.167	1772.167	6687.833	0.7323	0.7334	2.8117	2.8601	1.0172	0.9831	0.9985
6 30	TEA028452.1 TEA008343.1	1172.833	4526.167	1613.667	6237.333	0.7268	0.7257	2.6073	2.5709	0.986	1.0142	1.0016
7 8	TEA016969.1 TEA013270.1	3293.333	13246.67	4621.833	18004.17	0.7126	0.7358	2.248	2.9728	1.3224	0.7562	0.9685
7 9	TEA016969.1 TEA026716.1	1715.333	6605.667	2363	8884	0.7259	0.7435	2.5788	3.5666	1.383	0.723	0.9763
7 10	TEA016969.1 TEA028758.1	1047.667	4273.333	1507.667	5746.333	0.6949	0.7437	1.9581	3.5802	1.8284	0.5469	0.9344
7 11	TEA016969.1 TEA010804.1	2151.167	8349.833	2973	11394	0.7236	0.7328	2.5091	2.8326	1.1289	0.8858	0.9874
7 12	TEA016969.1 TEA009451.1	1462.167	5562.833	1972.5	7549.5	0.7413	0.7368	3.3405	3.0326	0.9078	1.1015	1.006
7 13	TEA016969.1 TEA021421.1	1736.833	6848.167	2485.833	9409.167	0.6987	0.7278	2.0117	2.6406	1.3126	0.7618	0.96
7 14	TEA016969.1 TEA017670.1	2308.667	9051.333	3232.333	12526.67	0.7142	0.7226	2.2825	2.4812	1.087	0.9199	0.9885
7 15	TEA016969.1 TEA019184.1	1593.833	6332.167	2285.167	8709.833	0.6975	0.727	1.994	2.6139	1.3109	0.7629	0.9594
7 16	TEA016969.1 TEA000933.1	1898	7584	2692.5	10423.5	0.7049	0.7276	2.1087	2.6328	1.2485	0.8009	0.9688
7 17	TEA016969.1 TEA031230.1	1396.5	5336.5	1939.667	7372.333	0.72	0.7239	2.4134	2.5173	1.043	0.9587	0.9946
7 18	TEA016969.1 TEA022171.1	988.3333	4029.667	1409.667	5424.333	0.7011	0.7429	2.0479	3.4936	1.7059	0.5862	0.9438
7 19	TEA016969.1 TEA011280.1	936.3333	3677.667	1328.5	5079.5	0.7048	0.724	2.1068	2.5221	1.1971	0.8353	0.9735
7 20	TEA016969.1 TEA031223.1	610.1667	2461.833	880.6667	3361.333	0.6928	0.7324	1.9307	2.814	1.4575	0.6861	0.946
7 21	TEA016969.1 TEA007232.1	1185.833	4538.167	1641.333	6179.667	0.7225	0.7344	2.4789	2.9032	1.1712	0.8539	0.9838
7 22	TEA016969.1 TEA016553.1	2439.167	9273.833	3356.5	12666.5	0.7267	0.7322	2.6037	2.8037	1.0768	0.9287	0.9925
7 23	TEA016969.1 TEA033032.1	2122.167	8235.833	2938.667	11254.33	0.7222	0.7318	2.47	2.7887	1.129	0.8857	0.9868
7 24	TEA016969.1 TEA001764.1	686.8333	2709.167	958.8333	3703.167	0.7163	0.7316	2.3274	2.78	1.1945	0.8372	0.9791
7 25	TEA016969.1 TEA026000.1	1575	6149	2187.667	8348.333	0.7199	0.7366	2.4128	3.0161	1.25	0.8	0.9775
7 26	TEA016969.1 TEA033556.1	509.6667	1968.333	722.6667	2715.333	0.7053	0.7249	2.1144	2.5478	1.205	0.8299	0.9729
7 27	TEA016969.1 TEA013875.1	664.8333	2517.167	911.6667	3429.333	0.7293	0.734	2.6907	2.8861	1.0726	0.9323	0.9935
7 28	TEA016969.1 TEA002722.1	1721	6566	2365.833	8977.167	0.7274	0.7314	2.6279	2.7731	1.0552	0.9476	0.9946
7 29	TEA016969.1 TEA030052.1	1296.5	4867.5	1783.5	6676.5	0.7269	0.729	2.6115	2.6834	1.0275	0.9732	0.9971
7 30	TEA016969.1 TEA008343.1	1176.667	4541.333	1621	6230	0.7259	0.7289	2.5781	2.6797	1.0394	0.9621	0.9958
8 9	TEA013270.1 TEA026716.1	1679.333	6452.667	2359.833	8887.167	0.7116	0.7261	2.2296	2.5836	1.1588	0.863	0.9801
8 10	TEA013270.1 TEA028758.1	1098	4211	1512.5	5741.5	0.726	0.7334	2.58	2.8595	1.1083	0.9023	0.9898
8 11	TEA013270.1 TEA010804.1	2057.833	8390.167	2954.667	11412.33	0.6965	0.7352	1.9799	2.9433	1.4866	0.6727	0.9473
8 12	TEA013270.1 TEA009451.1	1417.333	5575.667	1973	7549	0.7184	0.7386	2.3743	3.1396	1.3223	0.7563	0.9726
8 13	TEA013270.1 TEA021421.1	1784.167	6954.833	2480	9415	0.7194	0.7387	2.3998	3.1463	1.3111	0.7628	0.9739
8 14	TEA013270.1 TEA017670.1	2337.167	9118.833	3209.5	12549.5	0.7282	0.7266	2.6537	2.6014	0.9803	1.0201	1.0022
8 15	TEA013270.1 TEA019184.1	1635.333	6325.667	2278.667	8716.333	0.7177	0.7257	2.3581	2.573	1.0911	0.9165	0.9889
8 16	TEA013270.1 TEA000933.1	1884.667	7605.333	2685.5	10430.5	0.7018	0.7291	2.0584	2.6868	1.3053	0.7661	0.9625

8	17	TEA013270.1 TEA031230.1	1416.333	5400.667	1937.833	7374.167	0.7309	0.7324	2.7522	2.8131	1.0221	0.9783	0.998
8	18	TEA013270.1 TEA022171.1	1005.5	3925.5	1419.667	5414.333	0.7083	0.725	2.1665	2.5515	1.1777	0.8491	0.9769
8	19	TEA013270.1 TEA011280.1	961.1667	3750.833	1340.5	5067.5	0.717	0.7402	2.3432	3.2513	1.3875	0.7207	0.9687
8	20	TEA013270.1 TEA031223.1	645.5	2424.5	888.8333	3353.167	0.7262	0.723	2.5888	2.4945	0.9636	1.0378	1.0044
8	21	TEA013270.1 TEA007232.1	1174.167	4468.833	1646.667	6174.333	0.7131	0.7238	2.258	2.515	1.1138	0.8978	0.9852
8	22	TEA013270.1 TEA016553.1	2416.833	9221.167	3337.667	12685.33	0.7241	0.7269	2.5246	2.6107	1.0341	0.967	0.9961
8	23	TEA013270.1 TEA033032.1	2104.5	8192.5	2923.333	11269.67	0.7199	0.727	2.4116	2.6119	1.083	0.9233	0.9903
8	24	TEA013270.1 TEA001764.1	686.8333	2685.167	970.3333	3691.667	0.7078	0.7274	2.1588	2.6252	1.216	0.8223	0.9732
8	25	TEA013270.1 TEA026000.1	1591.667	6071.333	2184.333	8351.667	0.7287	0.727	2.6701	2.6122	0.9783	1.0222	1.0024
8	26	TEA013270.1 TEA033556.1	520.6667	1990.333	730.3333	2707.667	0.7129	0.7351	2.2552	2.9377	1.3026	0.7677	0.9699
8	27	TEA013270.1 TEA013875.1	661.1667	2472.833	918.6667	3422.333	0.7197	0.7226	2.4068	2.481	1.0308	0.9701	0.996
8	28	TEA013270.1 TEA002722.1	1717.667	6622.333	2360.167	8982.833	0.7278	0.7372	2.6391	3.0542	1.1573	0.8641	0.9872
8	29	TEA013270.1 TEA030052.1	1348.333	4848.667	1787	6673	0.7545	0.7266	nan	2.6008	nan	nan	1.0384
8	30	TEA013270.1 TEA008343.1	1193.667	4543.333	1626.167	6224.833	0.734	0.7299	2.8874	2.7135	0.9398	1.0641	1.0057
9	10	TEA026716.1 TEA028758.1	1065.5	4194.5	1493.833	5760.167	0.7133	0.7282	2.2623	2.6533	1.1728	0.8526	0.9795
9	11	TEA026716.1 TEA010804.1	1677.667	6580.333	2361.667	8885.333	0.7104	0.7406	2.2054	3.2832	1.4887	0.6717	0.9592
9	12	TEA026716.1 TEA009451.1	1425	5479	1965	7557	0.7252	0.725	2.5566	2.5516	0.998	1.002	1.0002
9	13	TEA026716.1 TEA021421.1	1700.5	6562.5	2377.333	8869.667	0.7153	0.7399	2.3049	3.2293	1.401	0.7138	0.9668
9	14	TEA026716.1 TEA017670.1	1725.333	6500.667	2338	8909	0.738	0.7297	3.0984	2.7061	0.8734	1.145	1.0113
9	15	TEA026716.1 TEA019184.1	1653.833	6314.167	2303.667	8691.333	0.7179	0.7265	2.3637	2.597	1.0987	0.9102	0.9882
9	16	TEA026716.1 TEA000933.1	1710	6496	2345	8902	0.7292	0.7297	2.6892	2.708	1.007	0.9931	0.9993
9	17	TEA026716.1 TEA031230.1	1397	5428	1930.5	7381.5	0.7236	0.7354	2.5114	2.9518	1.1754	0.8508	0.9841
9	18	TEA026716.1 TEA022171.1	994	3940	1397.5	5436.5	0.7113	0.7247	2.2226	2.5429	1.1441	0.8741	0.9814
9	19	TEA026716.1 TEA011280.1	937	3653	1316.667	5091.333	0.7116	0.7175	2.2299	2.354	1.0556	0.9473	0.9918
9	20	TEA026716.1 TEA031223.1	644.8333	2466.167	862.3333	3379.667	0.7478	0.7297	4.366	2.7074	0.6201	1.6127	1.0248
9	21	TEA026716.1 TEA007232.1	1139.167	4471.833	1626	6195	0.7006	0.7218	2.04	2.4618	1.2068	0.8287	0.9706
9	22	TEA026716.1 TEA016553.1	1726.667	6464.333	2362.667	8884.333	0.7308	0.7276	2.7494	2.6336	0.9579	1.044	1.0044
9	23	TEA026716.1 TEA033032.1	1716.5	6505.5	2345.167	8901.833	0.7319	0.7308	2.7944	2.749	0.9837	1.0165	1.0015
9	24	TEA026716.1 TEA001764.1	698	2716	942	3720	0.741	0.7301	3.3152	2.7223	0.8211	1.2178	1.0149
9	25	TEA026716.1 TEA026000.1	1553.167	6175.833	2195	8341	0.7076	0.7404	2.1546	3.2702	1.5178	0.6588	0.9557
9	26	TEA026716.1 TEA033556.1	514.5	2004.5	709	2729	0.7257	0.7345	2.5713	2.9103	1.1318	0.8835	0.988
9	27	TEA026716.1 TEA013875.1	654.3333	2547.667	890	3451	0.7352	0.7382	2.9444	3.1165	1.0584	0.9448	0.9959
9	28	TEA026716.1 TEA002722.1	1739	6570	2363.333	8883.667	0.7358	0.7396	2.9765	3.2058	1.077	0.9285	0.995

9	29	TEA026716.1 TEA030052.1	1296.5	4933.5	1762.667	6697.333	0.7355	0.7366	2.9612	3.0207	1.02	0.9803	0.9985
9	30	TEA026716.1 TEA008343.1	1142.333	4546.667	1605.667	6245.333	0.7114	0.728	2.2259	2.6471	1.1892	0.8409	0.9772
10	11	TEA028758.1 TEA010804.1	1097.5	4235.5	1502.667	5751.333	0.7304	0.7364	2.7322	3.0096	1.1015	0.9078	0.9918
10	12	TEA028758.1 TEA009451.1	1076.833	4293.167	1495.5	5758.5	0.72	0.7455	2.4154	3.843	1.591	0.6285	0.9658
10	13	TEA028758.1 TEA021421.1	1092	4328	1518.333	5735.667	0.7192	0.7546	2.3947	nan	nan	nan	0.9531
10	14	TEA028758.1 TEA017670.1	1049.5	4271.5	1483.333	5770.667	0.7075	0.7402	2.1534	3.254	1.5111	0.6618	0.9558
10	15	TEA028758.1 TEA019184.1	1031	4220	1501	5753	0.6869	0.7335	1.8562	2.8639	1.5429	0.6481	0.9364
10	16	TEA028758.1 TEA000933.1	1113.667	4214.333	1508.667	5745.333	0.7382	0.7335	3.1127	2.8636	0.92	1.087	1.0063
10	17	TEA028758.1 TEA031230.1	1070.833	4227.167	1505.167	5748.833	0.7114	0.7353	2.2259	2.9496	1.3251	0.7546	0.9675
10	18	TEA028758.1 TEA022171.1	975.1667	4002.833	1410.5	5423.5	0.6914	0.7381	1.9115	3.1047	1.6242	0.6157	0.9367
10	19	TEA028758.1 TEA011280.1	959.5	3738.5	1326.167	5081.833	0.7235	0.7357	2.5076	2.9677	1.1835	0.8449	0.9835
10	20	TEA028758.1 TEA031223.1	623.6667	2487.333	874.3333	3367.667	0.7133	0.7386	2.2631	3.1394	1.3872	0.7209	0.9658
10	21	TEA028758.1 TEA007232.1	1061.5	4207.5	1520.167	5733.833	0.6983	0.7338	2.0057	2.8764	1.4341	0.6973	0.9516
10	22	TEA028758.1 TEA016553.1	1078.167	4215.833	1487.167	5766.833	0.725	0.731	2.5503	2.7586	1.0817	0.9245	0.9917
10	23	TEA028758.1 TEA033032.1	1044.167	4201.833	1475.333	5778.667	0.7077	0.7271	2.1573	2.6176	1.2134	0.8242	0.9733
10	24	TEA028758.1 TEA001764.1	665.6667	2704.333	951.3333	3710.667	0.6997	0.7288	2.0268	2.6745	1.3156	0.7578	0.9601
10	25	TEA028758.1 TEA026000.1	1085.333	4157.667	1518.333	5735.667	0.7148	0.7249	2.2947	2.5473	1.1101	0.9008	0.9861
10	26	TEA028758.1 TEA033556.1	518.5	1985.5	721.3333	2716.667	0.7188	0.7309	2.3849	2.7512	1.1537	0.8669	0.9835
10	27	TEA028758.1 TEA013875.1	661	2562	902.5	3438.5	0.7324	0.7451	2.8146	3.772	1.3401	0.7462	0.983
10	28	TEA028758.1 TEA002722.1	1086.167	4244.833	1505.5	5748.5	0.7215	0.7384	2.4517	3.1284	1.276	0.7837	0.977
10	29	TEA028758.1 TEA030052.1	1110	4213	1524.667	5729.333	0.728	0.7353	2.6477	2.9511	1.1146	0.8972	0.9901
10	30	TEA028758.1 TEA008343.1	1053.167	4212.833	1492.667	5761.333	0.7056	0.7312	2.1195	2.7657	1.3049	0.7663	0.9649
11	12	TEA010804.1 TEA009451.1	1402	5531	1961.167	7560.833	0.7149	0.7315	2.296	2.7781	1.21	0.8265	0.9772
11	13	TEA010804.1 TEA021421.1	1809.667	6902.333	2493.333	9401.667	0.7258	0.7342	2.5754	2.8932	1.1234	0.8901	0.9886
11	14	TEA010804.1 TEA017670.1	2083.5	8296.5	2955.667	11411.33	0.7049	0.727	2.1087	2.6148	1.24	0.8065	0.9696
11	15	TEA010804.1 TEA019184.1	1647.333	6341.667	2280.5	8714.5	0.7224	0.7277	2.4755	2.6371	1.0653	0.9387	0.9926
11	16	TEA010804.1 TEA000933.1	1950.167	7561.833	2707.333	10408.67	0.7203	0.7265	2.4224	2.5971	1.0721	0.9327	0.9915
11	17	TEA010804.1 TEA031230.1	1368	5297	1922.5	7389.5	0.7116	0.7168	2.2285	2.3388	1.0495	0.9528	0.9927
11	18	TEA010804.1 TEA022171.1	1000.833	3975.167	1405.667	5428.333	0.712	0.7323	2.2368	2.8099	1.2575	0.7961	0.9723
11	19	TEA010804.1 TEA011280.1	943.8333	3793.167	1325.833	5082.167	0.7119	0.7464	2.2345	3.9977	1.7891	0.5589	0.9538
11	20	TEA010804.1 TEA031223.1	638.6667	2522.333	877.6667	3364.333	0.7277	0.7497	2.6362	5.9402	2.2533	0.4438	0.9706
11	21	TEA010804.1 TEA007232.1	1147.833	4536.167	1630.5	6190.5	0.704	0.7328	2.0932	2.8297	1.3518	0.7397	0.9607
11	22	TEA010804.1 TEA016553.1	2172.333	8358.667	3026.5	11340.5	0.7178	0.7371	2.3604	3.045	1.26	0.7752	0.9738

11 23	TEA010804.1 TEA033032.1	2111.833	8242.167	2948	11245	0.7164	0.733	2.3283	2.8385	1.2191	0.8203	0.9774
11 24	TEA010804.1 TEA001764.1	677	2682	959.6667	3702.333	0.7055	0.7244	2.1177	2.5333	1.1962	0.8359	0.9738
11 25	TEA010804.1 TEA026000.1	1537.167	6166.833	2177.167	8358.833	0.706	0.7378	2.1276	3.0867	1.4508	0.6893	0.957
11 26	TEA010804.1 TEA033556.1	486.1667	2001.833	715.1667	2722.833	0.6798	0.7352	1.7765	2.9442	1.6573	0.6034	0.9246
11 27	TEA010804.1 TEA013875.1	668.3333	2496.667	909.5	3431.5	0.7348	0.7276	2.9259	2.6324	0.8997	1.1115	1.01
11 28	TEA010804.1 TEA002722.1	1722.167	6582.833	2365.167	8977.833	0.7281	0.7332	2.6515	2.8504	1.075	0.9302	0.9931
11 29	TEA010804.1 TEA030052.1	1304	4943	1768.5	6691.5	0.7373	0.7387	3.0617	3.1463	1.0276	0.9731	0.9982
11 30	TEA010804.1 TEA008343.1	1164	4609	1610	6241	0.723	0.7385	2.4927	3.1335	1.2517	0.7955	0.979
12 13	TEA009451.1 TEA021421.1	1472	5555	1984.167	7537.833	0.7419	0.7369	3.3937	3.0384	0.8953	1.1169	1.0067
12 14	TEA009451.1 TEA017670.1	1398.333	5598.667	1943	7579	0.7197	0.7387	2.4061	3.147	1.3079	0.7646	0.9742
12 15	TEA009451.1 TEA019184.1	1403.167	5565.833	1965.5	7556.5	0.7139	0.7366	2.2753	3.0165	1.3257	0.7543	0.9692
12 16	TEA009451.1 TEA000933.1	1437.5	5587.5	1966.833	7555.167	0.7309	0.7396	2.7516	3.2058	1.1651	0.8583	0.9882
12 17	TEA009451.1 TEA031230.1	1361.5	5467.5	1924.167	7387.833	0.7076	0.7401	2.1543	3.2432	1.5054	0.6642	0.9561
12 18	TEA009451.1 TEA022171.1	986.6667	3993.333	1400.333	5433.667	0.7046	0.7349	2.1033	2.9302	1.3931	0.7178	0.9587
12 19	TEA009451.1 TEA011280.1	985.6667	3785.333	1316.833	5091.167	0.7485	0.7435	4.6674	3.5624	0.7632	1.3102	1.0067
12 20	TEA009451.1 TEA031223.1	603.6667	2515.333	861.6667	3380.333	0.7006	0.7441	2.0398	3.6349	1.782	0.5612	0.9415
12 21	TEA009451.1 TEA007232.1	1183.5	4577.5	1629.333	6191.667	0.7264	0.7393	2.5932	3.1874	1.2291	0.8136	0.9825
12 22	TEA009451.1 TEA016553.1	1408.167	5542.833	1950.333	7571.667	0.722	0.732	2.4663	2.7993	1.135	0.881	0.9863
12 23	TEA009451.1 TEA033032.1	1419.167	5532.833	1945.5	7576.5	0.7295	0.7303	2.6983	2.7282	1.0111	0.9891	0.9989
12 24	TEA009451.1 TEA001764.1	705.6667	2729.333	944.1667	3717.833	0.7474	0.7341	4.2474	2.8912	0.6807	1.469	1.0181
12 25	TEA009451.1 TEA026000.1	1449.667	5585.333	1968.333	7553.667	0.7365	0.7394	3.0127	3.1958	1.0608	0.9427	0.996
12 26	TEA009451.1 TEA033556.1	518	2028	704.8333	2733.167	0.7349	0.742	2.9303	3.4051	1.162	0.8606	0.9905
12 27	TEA009451.1 TEA013875.1	652.6667	2527.333	892.3333	3448.667	0.7314	0.7328	2.7733	2.8333	1.0216	0.9788	0.9981
12 28	TEA009451.1 TEA002722.1	1419.333	5539.667	1977	7545	0.7179	0.7342	2.3639	2.8959	1.225	0.8163	0.9778
12 29	TEA009451.1 TEA030052.1	1334.167	4874.833	1767.333	6692.667	0.7549	0.7284	nan	2.66	nan	nan	1.0364
12 30	TEA009451.1 TEA008343.1	1152.167	4563.833	1609.5	6241.5	0.7159	0.7312	2.3171	2.765	1.1933	0.838	0.979
13 14	TEA021421.1 TEA017670.1	1793.5	6838.5	2463.667	9431.333	0.728	0.7251	2.6461	2.5534	0.965	1.0363	1.004
13 15	TEA021421.1 TEA019184.1	1619	6431	2298.5	8696.5	0.7044	0.7395	2.0997	3.201	1.5245	0.6559	0.9525
13 16	TEA021421.1 TEA000933.1	1738.667	6904.333	2471.5	9423.5	0.7035	0.7327	2.0852	2.8258	1.3552	0.7379	0.9602
13 17	TEA021421.1 TEA031230.1	1361.667	5443.333	1951.167	7360.833	0.6979	0.7395	1.9998	3.2015	1.6009	0.6246	0.9437
13 18	TEA021421.1 TEA022171.1	980.6667	3945.333	1422.667	5411.333	0.6893	0.7291	1.8858	2.6848	1.4237	0.7024	0.9455
13 19	TEA021421.1 TEA011280.1	917.8333	3735.167	1334.667	5073.333	0.6877	0.7362	1.8659	2.9985	1.607	0.6223	0.9341
13 20	TEA021421.1 TEA031223.1	636.5	2457.5	875.5	3366.5	0.727	0.73	2.6139	2.7178	1.0397	0.9618	0.9959

13 21	TEA021421.1 TEA007232.1	1197.333	4534.667	1650.833	6170.167	0.7253	0.7349	2.5597	2.9307	1.1449	0.8734	0.9869
13 22	TEA021421.1 TEA016553.1	1805	6874	2507.5	9387.5	0.7198	0.7323	2.4102	2.8078	1.1653	0.8584	0.9831
13 23	TEA021421.1 TEA033032.1	1744.5	6913.5	2476.333	9418.667	0.7045	0.734	2.1013	2.8866	1.3737	0.7279	0.9597
13 24	TEA021421.1 TEA001764.1	671.6667	2703.333	959.3333	3702.667	0.7001	0.7301	2.0331	2.7222	1.3389	0.7469	0.959
13 25	TEA021421.1 TEA026000.1	1556.667	6131.333	2199.5	8336.5	0.7077	0.7355	2.1571	2.9584	1.3715	0.7291	0.9623
13 26	TEA021421.1 TEA033556.1	500	2020	718.3333	2719.667	0.6961	0.7427	1.9741	3.4781	1.7619	0.5676	0.9371
13 27	TEA021421.1 TEA013875.1	635	2563	905.8333	3435.167	0.701	0.7461	2.0464	3.9456	1.9281	0.5187	0.9396
13 28	TEA021421.1 TEA002722.1	1719.5	6633.5	2377.167	8965.833	0.7233	0.7399	2.5027	3.228	1.2898	0.7753	0.9777
13 29	TEA021421.1 TEA030052.1	1288.333	4961.667	1794.5	6665.5	0.7179	0.7444	2.3642	3.6703	1.5524	0.6441	0.9645
13 30	TEA021421.1 TEA008343.1	1125.167	4596.833	1630.833	6220.167	0.6899	0.739	1.8935	3.1681	1.6731	0.5977	0.9336
14 15	TEA017670.1 TEA019184.1	1598.333	6409.667	2261.167	8733.833	0.7069	0.7339	2.1418	2.8804	1.3448	0.7436	0.9632
14 16	TEA017670.1 TEA000933.1	1910.167	7575.833	2672.667	10443.33	0.7147	0.7254	2.2922	2.5637	1.1184	0.8941	0.9852
14 17	TEA017670.1 TEA031230.1	1386.167	5399.833	1906.5	7405.5	0.7271	0.7292	2.6159	2.6876	1.0274	0.9733	0.9971
14 18	TEA017670.1 TEA022171.1	988.6667	3920.333	1394.333	5439.667	0.7091	0.7207	2.181	2.4317	1.1149	0.8969	0.9839
14 19	TEA017670.1 TEA011280.1	929.1667	3724.833	1312.333	5095.667	0.708	0.731	2.1623	2.756	1.2746	0.7846	0.9686
14 20	TEA017670.1 TEA031223.1	615.3333	2468.667	862	3380	0.7138	0.7304	2.2742	2.7324	1.2015	0.8323	0.9774
14 21	TEA017670.1 TEA007232.1	1132.667	4521.333	1615.167	6205.833	0.7013	0.7286	2.0503	2.6662	1.3004	0.769	0.9625
14 22	TEA017670.1 TEA016553.1	2426	9044	3304.5	12454.5	0.7342	0.7262	2.8927	2.5866	0.8942	1.1183	1.011
14 23	TEA017670.1 TEA033032.1	2155.167	8208.833	2924.167	11268.83	0.737	0.7285	3.0424	2.6624	0.8751	1.1427	1.0118
14 24	TEA017670.1 TEA001764.1	693.8333	2734.167	941.8333	3720.167	0.7367	0.735	3.0233	2.9319	0.9698	1.0312	1.0023
14 25	TEA017670.1 TEA026000.1	1520.167	6115.833	2160.5	8375.5	0.7036	0.7302	2.0874	2.726	1.3059	0.7657	0.9636
14 26	TEA017670.1 TEA033556.1	520	1966	713	2725	0.7293	0.7215	2.6929	2.4518	0.9105	1.0984	1.0109
14 27	TEA017670.1 TEA013875.1	635.8333	2492.167	890.8333	3450.167	0.7138	0.7223	2.2722	2.4749	1.0892	0.9181	0.9881
14 28	TEA017670.1 TEA002722.1	1686.167	6574.833	2338	9005	0.7212	0.7301	2.4448	2.7232	1.1139	0.8978	0.9878
14 29	TEA017670.1 TEA030052.1	1294.833	4957.167	1755.5	6704.5	0.7376	0.7394	3.076	3.1929	1.038	0.9634	0.9976
14 30	TEA017670.1 TEA008343.1	1159.5	4490.5	1595	6256	0.727	0.7178	2.6121	2.3609	0.9038	1.1064	1.0128
15 16	TEA019184.1 TEA000933.1	1631	6373	2268.5	8726.5	0.719	0.7303	2.389	2.7298	1.1426	0.8752	0.9845
15 17	TEA019184.1 TEA031230.1	1377.833	5335.167	1931	7381	0.7135	0.7228	2.2678	2.4883	1.0972	0.9114	0.9871
15 18	TEA019184.1 TEA022171.1	981.3333	3882.667	1402.667	5431.333	0.6996	0.7149	2.0254	2.2956	1.1334	0.8823	0.9787
15 19	TEA019184.1 TEA011280.1	918.6667	3669.333	1323.5	5084.5	0.6941	0.7217	1.9476	2.4571	1.2616	0.7926	0.9618
15 20	TEA019184.1 TEA031223.1	604.1667	2475.833	869.5	3372.5	0.6948	0.7341	1.9574	2.8915	1.4772	0.677	0.9465
15 21	TEA019184.1 TEA007232.1	1158.667	4483.333	1630.833	6190.167	0.7105	0.7243	2.2074	2.5292	1.1459	0.8727	0.981
15 22	TEA019184.1 TEA016553.1	1620.833	6418.167	2279.667	8715.333	0.711	0.7364	2.2173	3.0087	1.3569	0.737	0.9655

15 23	TEA019184.1 TEA033032.1	1591.167	6314.833	2268.667	8726.333	0.7014	0.7237	2.0518	2.5115	1.224	0.817	0.9692
15 24	TEA019184.1 TEA001764.1	667.1667	2708.833	948.6667	3713.333	0.7033	0.7295	2.0817	2.6993	1.2967	0.7712	0.9641
15 25	TEA019184.1 TEA026000.1	1557	6086	2184.833	8351.167	0.7126	0.7288	2.2496	2.6732	1.1883	0.8416	0.9779
15 26	TEA019184.1 TEA033556.1	495.1667	2025.833	716.1667	2721.833	0.6914	0.7443	1.9122	3.6584	1.9132	0.5227	0.929
15 27	TEA019184.1 TEA013875.1	651.1667	2485.833	898.5	3442.5	0.7247	0.7221	2.5427	2.4686	0.9708	1.03	1.0036
15 28	TEA019184.1 TEA002722.1	1636	6359	2287.5	8707.5	0.7152	0.7303	2.3027	2.7292	1.1852	0.8437	0.9793
15 29	TEA019184.1 TEA030052.1	1292.167	4871.833	1772	6688	0.7292	0.7284	2.6893	2.6621	0.9899	1.0102	1.0011
15 30	TEA019184.1 TEA008343.1	1115.667	4525.333	1611.333	6239.667	0.6924	0.7253	1.9247	2.5585	1.3293	0.7523	0.9547
16 17	TEA000933.1 TEA031230.1	1369	5368	1930	7382	0.7093	0.7272	2.1859	2.6191	1.1982	0.8346	0.9755
16 18	TEA000933.1 TEA022171.1	990.3333	3912.667	1412.5	5421.5	0.7011	0.7217	2.048	2.4578	1.2001	0.8333	0.9715
16 19	TEA000933.1 TEA011280.1	977.3333	3657.667	1336.833	5071.167	0.7311	0.7213	2.7599	2.4465	0.8864	1.1281	1.0136
16 20	TEA000933.1 TEA031223.1	620.3333	2438.667	880.3333	3361.667	0.7047	0.7254	2.1044	2.564	1.2184	0.8207	0.9714
16 21	TEA000933.1 TEA007232.1	1132	4536	1641.833	6179.167	0.6895	0.7341	1.8877	2.8894	1.5306	0.6533	0.9392
16 22	TEA000933.1 TEA016553.1	1963.167	7558.833	2740.5	10375.5	0.7164	0.7285	2.3281	2.665	1.1447	0.8736	0.9833
16 23	TEA000933.1 TEA033032.1	1906.5	7547.5	2694.5	10421.5	0.7076	0.7242	2.1539	2.528	1.1737	0.852	0.977
16 24	TEA000933.1 TEA001764.1	698.6667	2702.333	962	3700	0.7263	0.7304	2.5898	2.7319	1.0549	0.948	0.9944
16 25	TEA000933.1 TEA026000.1	1534.333	6135.667	2178.5	8357.5	0.7043	0.7342	2.0986	2.8927	1.3784	0.7255	0.9593
16 26	TEA000933.1 TEA033556.1	527.6667	2011.333	723.3333	2714.667	0.7295	0.7409	2.6995	3.31	1.2261	0.8156	0.9846
16 27	TEA000933.1 TEA013875.1	654	2501	910	3431	0.7187	0.7289	2.3819	2.6796	1.1249	0.8889	0.9859
16 28	TEA000933.1 TEA002722.1	1698.167	6586.833	2345.167	8997.833	0.7241	0.732	2.5248	2.7992	1.1087	0.902	0.9892
16 29	TEA000933.1 TEA030052.1	1292	4858	1777	6683	0.7271	0.7269	2.6157	2.6108	0.9981	1.0019	1.0002
16 30	TEA000933.1 TEA008343.1	1205	4549	1621.167	6229.833	0.7433	0.7302	3.5376	2.7256	0.7705	1.2979	1.0179
17 18	TEA031230.1 TEA022171.1	989.8333	3969.167	1408.833	5425.167	0.7026	0.7316	2.0709	2.7817	1.3432	0.7445	0.9603
17 19	TEA031230.1 TEA011280.1	929.3333	3682.667	1331	5077	0.6982	0.7254	2.0048	2.5619	1.2779	0.7826	0.9626
17 20	TEA031230.1 TEA031223.1	585	2406	873.1667	3368.833	0.67	0.7142	1.6783	2.2815	1.3594	0.7356	0.9381
17 21	TEA031230.1 TEA007232.1	1162.167	4480.833	1639	6182	0.7091	0.7248	2.1812	2.5455	1.167	0.8569	0.9783
17 22	TEA031230.1 TEA016553.1	1356.167	5434.833	1911.667	7400.333	0.7094	0.7344	2.1875	2.9048	1.3279	0.7531	0.966
17 23	TEA031230.1 TEA033032.1	1379.5	5367.5	1910.167	7401.833	0.7222	0.7252	2.471	2.5557	1.0343	0.9669	0.9959
17 24	TEA031230.1 TEA001764.1	688	2672	955.1667	3706.833	0.7203	0.7208	2.4215	2.4352	1.0056	0.9944	0.9993
17 25	TEA031230.1 TEA026000.1	1376.167	5483.833	1934.333	7377.667	0.7114	0.7433	2.2259	3.5387	1.5898	0.629	0.9571
17 26	TEA031230.1 TEA033556.1	503.1667	1997.833	719.5	2718.5	0.6993	0.7349	2.021	2.9292	1.4494	0.69	0.9516
17 27	TEA031230.1 TEA013875.1	632.6667	2490.333	904.1667	3436.833	0.6997	0.7246	2.0269	2.539	1.2526	0.7983	0.9657
17 28	TEA031230.1 TEA002722.1	1451	5361	1941	7371	0.7476	0.7273	4.2938	2.6236	0.611	1.6366	1.0278

17	29	TEA031230.1 TEA030052.1	1327.5	4866.5	1777	6683	0.747	0.7282	4.1526	2.6533	0.6389	1.5651	1.0259
17	30	TEA031230.1 TEA008343.1	1139	4521	1618.333	6232.667	0.7038	0.7254	2.0905	2.5621	1.2256	0.8159	0.9703
18	19	TEA022171.1 TEA011280.1	906.1667	3708.833	1322.167	5085.833	0.6854	0.7292	1.8385	2.6906	1.4635	0.6833	0.9398
18	20	TEA022171.1 TEA031223.1	607.8333	2455.167	862.8333	3379.167	0.7045	0.7266	2.1011	2.5992	1.2371	0.8084	0.9696
18	21	TEA022171.1 TEA007232.1	994.5	3956.5	1422.167	5411.833	0.6993	0.7311	2.0204	2.76	1.3661	0.732	0.9565
18	22	TEA022171.1 TEA016553.1	967.1667	3901.833	1395.833	5438.167	0.6929	0.7175	1.9314	2.3539	1.2187	0.8205	0.9657
18	23	TEA022171.1 TEA033032.1	989	3981	1382	5452	0.7156	0.7302	2.3122	2.7254	1.1787	0.8484	0.9801
18	24	TEA022171.1 TEA001764.1	671.8333	2676.167	942.5	3719.5	0.7128	0.7195	2.2532	2.4017	1.0659	0.9382	0.9907
18	25	TEA022171.1 TEA026000.1	1002	3950	1422.667	5411.333	0.7043	0.7299	2.0987	2.7164	1.2943	0.7726	0.9649
18	26	TEA022171.1 TEA033556.1	507	2029	707	2731	0.7171	0.743	2.3453	3.5004	1.4925	0.67	0.9652
18	27	TEA022171.1 TEA013875.1	648	2473	892.5	3448.5	0.7261	0.7171	2.5831	2.3455	0.908	1.1013	1.0124
18	28	TEA022171.1 TEA002722.1	1036.333	3977.667	1408.333	5425.667	0.7359	0.7331	2.9782	2.8455	0.9554	1.0466	1.0037
18	29	TEA022171.1 TEA030052.1	1030.333	3914.667	1426.667	5407.333	0.7222	0.724	2.4712	2.5202	1.0198	0.9806	0.9976
18	30	TEA022171.1 TEA008343.1	981.5	3896.5	1395.333	5438.667	0.7034	0.7164	2.0841	2.3301	1.118	0.8944	0.9818
19	20	TEA011280.1 TEA031223.1	603.1667	2420.833	869.3333	3372.667	0.6938	0.7178	1.9437	2.3606	1.2145	0.8234	0.9666
19	21	TEA011280.1 TEA007232.1	955.1667	3731.833	1345.333	5062.667	0.71	0.7371	2.1981	3.0488	1.387	0.721	0.9632
19	22	TEA011280.1 TEA016553.1	918.6667	3661.333	1316.667	5091.333	0.6977	0.7191	1.9976	2.3927	1.1978	0.8349	0.9702
19	23	TEA011280.1 TEA033032.1	931.8333	3711.167	1306.833	5101.167	0.713	0.7275	2.2578	2.6304	1.165	0.8584	0.9801
19	24	TEA011280.1 TEA001764.1	662.3333	2680.667	950	3712	0.6972	0.7222	1.9901	2.4703	1.2413	0.8056	0.9654
19	25	TEA011280.1 TEA026000.1	957.5	3740.5	1341.167	5066.833	0.7139	0.7382	2.276	3.116	1.3691	0.7304	0.9671
19	26	TEA011280.1 TEA033556.1	510.8333	1941.167	711.8333	2726.167	0.7176	0.712	2.3571	2.2379	0.9494	1.0533	1.0078
19	27	TEA011280.1 TEA013875.1	648.5	2514.5	898.5	3442.5	0.7218	0.7304	2.4595	2.7345	1.1118	0.8994	0.9881
19	28	TEA011280.1 TEA002722.1	987.8333	3770.167	1324.833	5083.167	0.7456	0.7417	3.8587	3.3775	0.8753	1.1425	1.0053
19	29	TEA011280.1 TEA030052.1	1022.667	3637.333	1347.833	5060.167	0.7587	0.7188	nan	2.3851	nan	nan	1.0556
19	30	TEA011280.1 TEA008343.1	940.1667	3719.833	1319.333	5088.667	0.7126	0.731	2.2489	2.7569	1.2259	0.8158	0.9748
20	21	TEA031223.1 TEA007232.1	646	2427	887.1667	3354.833	0.7282	0.7234	2.6523	2.5053	0.9446	1.0587	1.0065
20	22	TEA031223.1 TEA016553.1	627	2464	868.6667	3373.333	0.7218	0.7304	2.4605	2.7347	1.1114	0.8997	0.9882
20	23	TEA031223.1 TEA033032.1	611.1667	2439.833	853.8333	3388.167	0.7158	0.7201	2.3157	2.4168	1.0465	0.9582	0.994
20	24	TEA031223.1 TEA001764.1	618.1667	2432.833	856.8333	3385.167	0.7215	0.7187	2.4514	2.3817	0.9716	1.0293	1.0039
20	25	TEA031223.1 TEA026000.1	658.8333	2493.167	888.3333	3353.667	0.7417	0.7434	3.3735	3.5515	1.0528	0.9499	0.9976
20	26	TEA031223.1 TEA033556.1	484.5	2045.5	707.6667	2730.333	0.6846	0.7492	1.8302	5.1102	2.7921	0.3581	0.9139
20	27	TEA031223.1 TEA013875.1	606.5	2446.5	872.3333	3369.667	0.6953	0.726	1.9631	2.5826	1.3156	0.7601	0.9576
20	28	TEA031223.1 TEA002722.1	623.6667	2455.333	864.5	3377.5	0.7214	0.727	2.4505	2.6124	1.0661	0.938	0.9924

20 29	TEA031223.1 TEA030052.1	667.8333	2447.167	885.5	3356.5	0.7542	0.7291	nan	2.6846	nan	nan	1.0344
20 30	TEA031223.1 TEA008343.1	615.6667	2461.333	859.6667	3382.333	0.7162	0.7277	2.324	2.6367	1.1345	0.8814	0.9842
21 22	TEA007232.1 TEA016553.1	1161	4450	1618.5	6202.5	0.7173	0.7175	2.3502	2.353	1.0012	0.9988	0.9998
21 23	TEA007232.1 TEA033032.1	1155.167	4564.833	1605.833	6215.167	0.7194	0.7345	2.3982	2.9078	1.2125	0.8248	0.9794
21 24	TEA007232.1 TEA001764.1	688	2682	970.6667	3691.333	0.7088	0.7266	2.1761	2.5994	1.1943	0.8371	0.9755
21 25	TEA007232.1 TEA026000.1	1193.333	4558.667	1651.333	6169.667	0.7226	0.7389	2.4835	3.1588	1.2719	0.7862	0.978
21 26	TEA007232.1 TEA033556.1	504.3333	1981.667	730	2708	0.6909	0.7318	1.9052	2.7883	1.4635	0.6833	0.9441
21 27	TEA007232.1 TEA013875.1	640.5	2513.5	917.5	3423.5	0.6981	0.7342	2.003	2.8946	1.4451	0.692	0.9508
21 28	TEA007232.1 TEA002722.1	1217	4527	1643.833	6177.167	0.7403	0.7329	3.2643	2.834	0.8682	1.1518	1.0102
21 29	TEA007232.1 TEA030052.1	1202	4516	1656.333	6164.667	0.7257	0.7326	2.5722	2.8211	1.0968	0.9118	0.9906
21 30	TEA007232.1 TEA008343.1	1195	4541	1625.167	6195.833	0.7353	0.7329	2.9496	2.8363	0.9616	1.04	1.0033
22 23	TEA016553.1 TEA033032.1	2165.167	8170.833	2996.833	11196.17	0.7225	0.7298	2.479	2.7104	1.0933	0.9146	0.99
22 24	TEA016553.1 TEA001764.1	690.8333	2713.167	947.8333	3714.167	0.7289	0.7305	2.6765	2.7369	1.0226	0.9779	0.9978
22 25	TEA016553.1 TEA026000.1	1553.167	6053.833	2175	8361	0.7141	0.7241	2.2795	2.5231	1.1069	0.9034	0.9862
22 26	TEA016553.1 TEA033556.1	513.3333	2013.667	716	2722	0.7169	0.7398	2.3414	3.2214	1.3758	0.7268	0.9691
22 27	TEA016553.1 TEA013875.1	637.3333	2512.667	897.3333	3443.667	0.7103	0.7296	2.2031	2.7052	1.2279	0.8144	0.9734
22 28	TEA016553.1 TEA002722.1	1710	6589	2366.667	8976.333	0.7225	0.734	2.4804	2.8876	1.1642	0.859	0.9843
22 29	TEA016553.1 TEA030052.1	1310	4946	1754.167	6705.833	0.7468	0.7376	4.0911	3.0748	0.7516	1.3305	1.0125
22 30	TEA016553.1 TEA008343.1	1128.333	4551.667	1597.667	6253.333	0.7062	0.7279	2.131	2.6426	1.2401	0.8064	0.9703
23 24	TEA033032.1 TEA001764.1	667.1667	2652.833	935	3727	0.7135	0.7118	2.268	2.2327	0.9844	1.0158	1.0025
23 25	TEA033032.1 TEA026000.1	1548.333	6164.667	2170.167	8365.833	0.7135	0.7369	2.2663	3.0348	1.3391	0.7468	0.9682
23 26	TEA033032.1 TEA033556.1	519.3333	1953.667	696.6667	2741.333	0.7455	0.7127	3.8295	2.2502	0.5876	1.7018	1.046
23 27	TEA033032.1 TEA013875.1	630.3333	2503.667	885.1667	3455.833	0.7121	0.7245	2.239	2.5353	1.1323	0.8831	0.9829
23 28	TEA033032.1 TEA002722.1	1689.833	6498.167	2347	8996	0.72	0.7223	2.4141	2.4751	1.0253	0.9754	0.9968
23 29	TEA033032.1 TEA030052.1	1259.833	4940.167	1745.333	6714.667	0.7218	0.7357	2.4614	2.9713	1.2071	0.8284	0.9811
23 30	TEA033032.1 TEA008343.1	1107.5	4563.5	1585.167	6265.833	0.6987	0.7283	2.0113	2.6576	1.3213	0.7568	0.9593
24 25	TEA001764.1 TEA026000.1	681.8333	2790.167	972.5	3689.5	0.7011	0.7562	2.0479	nan	nan	nan	0.9271
24 26	TEA001764.1 TEA033556.1	495.5	1991.5	700.8333	2737.167	0.707	0.7276	2.1444	2.6325	1.2272	0.8146	0.9717
24 27	TEA001764.1 TEA013875.1	650.8333	2510.167	888.8333	3452.167	0.7322	0.7271	2.8071	2.6176	0.9325	1.0724	1.007
24 28	TEA001764.1 TEA002722.1	670.1667	2749.833	951.3333	3710.667	0.7044	0.7411	2.1009	3.3223	1.5814	0.6324	0.9506
24 29	TEA001764.1 TEA030052.1	708.5	2692.5	964	3698	0.735	0.7281	2.9319	2.6501	0.9039	1.1064	1.0094
24 30	TEA001764.1 TEA008343.1	704	2694	942.1667	3719.833	0.7472	0.7242	4.1966	2.528	0.6024	1.66	1.0317
25 26	TEA026000.1 TEA033556.1	520.8333	2026.167	728.1667	2709.833	0.7153	0.7477	2.3043	4.3433	1.8849	0.5305	0.9566

25 27	TEA026000.1 TEA013875.1	664.8333	2571.167	918.1667	3422.833	0.7241	0.7512	2.524	nan	nan	nan	0.9639
25 28	TEA026000.1 TEA002722.1	1583	6179	2191.5	8344.5	0.7223	0.7405	2.475	3.2756	1.3235	0.7556	0.9755
25 29	TEA026000.1 TEA030052.1	1292.5	4976.5	1781.667	6678.333	0.7254	0.7452	2.5643	3.7841	1.4757	0.6777	0.9735
25 30	TEA026000.1 TEA008343.1	1186	4602	1631.167	6219.833	0.7271	0.7399	2.6163	3.23	1.2346	0.81	0.9827
26 27	TEA033556.1 TEA013875.1	506.1667	1973.833	715.5	2722.5	0.7074	0.725	2.1517	2.5511	1.1856	0.8434	0.9758
26 28	TEA033556.1 TEA002722.1	537.5	2017.5	709.8333	2728.167	0.7572	0.7395	nan	3.2021	nan	nan	1.024
26 29	TEA033556.1 TEA030052.1	554.8333	1929.167	725.3333	2712.667	0.7649	0.7112	nan	2.2207	nan	nan	1.0756
26 30	TEA033556.1 TEA008343.1	499.8333	1989.167	705.5	2732.5	0.7085	0.728	2.1704	2.6456	1.2189	0.8204	0.9732
27 28	TEA013875.1 TEA002722.1	667	2535	896.1667	3444.833	0.7443	0.7359	3.6572	2.9796	0.8147	1.2274	1.0114
27 29	TEA013875.1 TEA030052.1	667.8333	2482.167	916.3333	3424.667	0.7288	0.7248	2.6749	2.5446	0.9529	1.0512	1.0055
27 30	TEA013875.1 TEA008343.1	642.1667	2528.833	890	3451	0.7215	0.7328	2.4536	2.8306	1.1536	0.8668	0.9847
28 29	TEA002722.1 TEA030052.1	1311.333	4937.667	1784.333	6675.667	0.7349	0.7397	2.9298	3.2124	1.0744	0.912	0.9936
28 30	TEA002722.1 TEA008343.1	1198.833	4603.167	1624.333	6226.667	0.738	0.7393	3.1043	3.185	1.026	0.9746	0.9983
29 30	TEA030052.1 TEA008343.1	1187	4619	1636.833	6214.167	0.7252	0.7433	2.5563	3.5387	1.3843	0.7224	0.9756

Supplementary Table S6: Ka/Ks ratios of MAPKKKs of ZIK subfamily in C. sinensis.

Compare	Sequence names	Sd	Sn	S	N	ps	pn	ds	dn	dn/ds	ds/dn	ps/pn
0 1	TEA010125.1 TEA022762.1	1691.667	6732.333	2360.833	9324.167	0.7166	0.722	2.3326	2.4667	1.0575	0.9456	0.9924
0 2	TEA010125.1 TEA024720.1	1437.333	5815.667	2052.833	8048.167	0.7002	0.7226	2.0336	2.4824	1.2207	0.8192	0.9689
0 3	TEA010125.1 TEA002087.1	842.6667	3302.333	1151.667	4584.333	0.7317	0.7204	2.7846	2.423	0.8701	1.1492	1.0157
0 4	TEA010125.1 TEA013346.1	1408.833	5667.167	1998.167	7757.833	0.7051	0.7305	2.1111	2.7376	1.2968	0.7712	0.9652
0 5	TEA010125.1 TEA013344.1	441.5	1697.5	605.3333	2370.667	0.7294	0.716	2.6943	2.3212	0.8615	1.1607	1.0186
0 6	TEA010125.1 TEA031068.1	957	4004	1365.167	5546.833	0.701	0.7219	2.0464	2.462	1.2031	0.8312	0.9711
0 7	TEA010125.1 TEA020698.1	714	2845	988	3941	0.7227	0.7219	2.4841	2.4632	0.9916	1.0085	1.0011
0 8	TEA010125.1 TEA027328.1	699.3333	2752.667	966.6667	3812.333	0.7234	0.722	2.5057	2.467	0.9845	1.0157	1.0019
0 9	TEA010125.1 TEA020112.1	312	1296	446.1667	1734.833	0.6993	0.747	2.0205	4.1526	2.0552	0.4865	0.9361
0 10	TEA010125.1 TEA033250.1	930.1667	3733.833	1290.167	5120.833	0.721	0.7291	2.4387	2.6869	1.1018	0.9076	0.9888
1 2	TEA022762.1 TEA024720.1	1473.333	5804.667	2070.667	8030.333	0.7115	0.7228	2.2276	2.4888	1.1175	0.895	0.9843
1 3	TEA022762.1 TEA002087.1	836	3341	1165.833	4570.167	0.7171	0.731	2.3446	2.7585	1.17653	0.8499	0.9809
1 4	TEA022762.1 TEA013346.1	1459.333	5610.667	2014.667	7741.333	0.7244	0.7248	2.5318	2.544	1.0048	0.9952	0.9994
1 5	TEA022762.1 TEA013344.1	468.1667	1715.833	618	2358	0.7576	0.7277	nan	2.6354	nan	nan	1.0411
1 6	TEA022762.1 TEA031068.1	1094.333	4497.667	1531.5	6106.5	0.7146	0.7365	2.289	3.0151	1.3172	0.7592	0.9701

1 7	TEA022762.1 TEA020698.1	702	2859	995.3333	3933.667	0.7053	0.7268	2.1149	2.607	1.2327	0.8112	0.9704
1 8	TEA022762.1 TEA027328.1	680.8333	2755.167	977.3333	3801.667	0.6966	0.7247	1.982	2.5427	1.2829	0.7795	0.9612
1 9	TEA022762.1 TEA020112.1	301.8333	1249.167	446.6667	1734.333	0.6757	0.7203	1.7344	2.4206	1.3956	0.7165	0.9382
1 10	TEA022762.1 TEA033250.1	958.3333	3710.667	1310	5101	0.7316	0.7274	2.7788	2.6279	0.9457	1.0574	1.0057
2 3	TEA024720.1 TEA002087.1	877.8333	3286.167	1183.667	4552.333	0.7416	0.7219	3.3709	2.4623	0.7304	1.369	1.0274
2 4	TEA024720.1 TEA013346.1	1498.333	5602.667	2028.667	7727.333	0.7386	0.725	3.1386	2.5523	0.8132	1.2297	1.0187
2 5	TEA024720.1 TEA013344.1	438.5	1723.5	627.1667	2348.833	0.6992	0.7338	2.0188	2.8748	1.424	0.7022	0.9529
2 6	TEA024720.1 TEA031068.1	782.5	3087.5	1086.167	4241.833	0.7204	0.7279	2.4248	2.6423	1.897	0.9177	0.9898
2 7	TEA024720.1 TEA020698.1	732.6667	2853.333	1010.5	3918.5	0.7251	0.7282	2.5525	2.6526	1.0392	0.9623	0.9957
2 8	TEA024720.1 TEA027328.1	722.1667	2729.833	990.1667	3788.833	0.7293	0.7205	2.6939	2.4266	0.9008	1.1101	1.0123
2 9	TEA024720.1 TEA020112.1	340.5	1288.5	456.1667	1724.833	0.7464	0.747	4.0123	4.1483	1.0339	0.9672	0.9992
2 10	TEA024720.1 TEA033250.1	946.6667	3733.333	1325.667	5085.333	0.7141	0.7341	2.2796	2.8921	1.2687	0.7882	0.9727
3 4	TEA002087.1 TEA013346.1	848.3333	3376.667	1178.5	4557.5	0.7198	0.7409	2.4102	3.3091	1.3729	0.7284	0.9716
3 5	TEA002087.1 TEA013344.1	472.1667	1750.833	618.3333	2357.667	0.7636	0.7426	nan	3.4652	nan	nan	1.0283
3 6	TEA002087.1 TEA031068.1	535.6667	2145.333	755	2935	0.7095	0.7309	2.1889	2.7547	1.2585	0.7946	0.9706
3 7	TEA002087.1 TEA020698.1	750.6667	2886.333	1015.5	3913.5	0.7392	0.7375	3.181	3.0727	0.966	1.0352	1.0023
3 8	TEA002087.1 TEA027328.1	735.5	2805.5	995	3784	0.7392	0.7414	3.1801	3.3522	1.0541	0.9487	0.997
3 9	TEA002087.1 TEA020112.1	326.3333	1248.667	446.1667	1734.833	0.7314	0.7198	2.7733	2.4082	0.8683	1.1516	1.0162
3 10	TEA002087.1 TEA033250.1	891.8333	3355.167	1192	4544	0.7482	0.7384	4.5169	3.1251	0.6919	1.4454	1.0133
4 5	TEA013346.1 TEA013344.1	428.8333	1722.167	621	2355	0.6906	0.7313	1.9012	2.7679	1.4559	0.6869	0.9443
4 6	TEA013346.1 TEA031068.1	743.5	2872.5	1027.5	3955.5	0.7236	0.7262	2.5101	2.5879	1.031	0.9699	0.9964
4 7	TEA013346.1 TEA020698.1	724.6667	2862.333	1006.833	3922.167	0.7197	0.7298	2.4079	2.7102	1.1255	0.8885	0.9862
4 8	TEA013346.1 TEA027328.1	735	2760	989.8333	3789.167	0.7425	0.7284	3.4588	2.6603	0.7691	1.3002	1.0194
4 9	TEA013346.1 TEA020112.1	322.3333	1236.667	446.3333	1734.667	0.7222	0.7129	2.4708	2.2551	0.9127	1.0956	1.013
4 10	TEA013346.1 TEA033250.1	955.5	3713.5	1318.833	5092.167	0.7245	0.7293	2.5362	2.6909	1.061	0.9425	0.9935
5 6	TEA013344.1 TEA031068.1	470.3333	1712.667	620.8333	2355.167	0.7576	0.7272	nan	2.6198	nan	nan	1.0418
5 7	TEA013344.1 TEA020698.1	441.6667	1694.333	618.1667	2357.833	0.7145	0.7186	2.2874	2.3799	1.0404	0.9612	0.9943
5 8	TEA013344.1 TEA027328.1	452.5	1701.5	620.1667	2355.833	0.7296	0.7222	2.705	2.4726	0.9141	1.094	1.0102
5 9	TEA013344.1 TEA020112.1	308.5	1257.5	455.1667	1725.833	0.6778	0.7286	1.7552	2.6687	1.5204	0.6577	0.9302
5 10	TEA013344.1 TEA033250.1	467.6667	1689.333	627.3333	2348.667	0.7455	0.7193	3.8343	2.3962	0.6249	1.6001	1.0364
6 7	TEA031068.1 TEA020698.1	549.1667	2114.833	753.5	2936.5	0.7288	0.7202	2.6753	2.4189	0.9042	1.106	1.012
6 8	TEA031068.1 TEA027328.1	542.1667	2114.833	762.1667	2927.833	0.7113	0.7223	2.2241	2.4745	1.1126	0.8988	0.9848
6 9	TEA031068.1 TEA020112.1	328.3333	1244.667	452.6667	1728.333	0.7253	0.7202	2.5609	2.418	0.9442	1.0591	1.0072

6 10	TEA031068.1 TEA033250.1	558.6667	2136.333	758	2932	0.737	0.7286	3.0429	2.6684	0.8769	1.1403	1.0115
7 8	TEA020698.1 TEA027328.1	728.3333	2751.667	985.5	3793.5	0.739	0.7254	3.17	2.5619	0.8082	1.2374	1.0189
7 9	TEA020698.1 TEA020112.1	322	1235	449.8333	1731.167	0.7158	0.7134	2.3163	2.2648	0.9778	1.0227	1.0034
7 10	TEA020698.1 TEA033250.1	781.8333	2832.167	1016.833	3912.167	0.7689	0.7239	nan	2.5197	nan	nan	1.0621
8 9	TEA027328.1 TEA020112.1	314.1667	1253.833	450.5	1730.5	0.6974	0.7245	1.9926	2.5375	1.2735	0.7853	0.9625
8 10	TEA027328.1 TEA033250.1	736	2742	998	3781	0.7375	0.7252	3.0693	2.5571	0.8331	1.2003	1.0169
9 10	TEA020112.1 TEA033250.1	330.3333	1284.667	453.1667	1727.833	0.7289	0.7435	2.6797	3.5627	1.3295	0.7521	0.9804

Supplementary Table S7: Tissue specific expression data of tea MAPKKKs

MEKK	Gene	Apical bud	Flower	Fruit	Young leaf	Mature leaf	Old leaf	Root	Stem
	TEA028357.1	10.60070377	1.657135892	5.858041183	5.227850598	2.589275721	1.714886623	7.980895909	10.58941704
	TEA025870.1	19.97183129	1.215232988	0.292445114	17.02051031	2.960498047	3.925958717	6.737247099	6.880238296
	TEA016319.1	58.03599874	50.65814207	76.57492523	109.7248708	91.04227536	69.21352052	34.65569402	79.8626543
	TEA008165.1	37.51964083	11.55977825	24.36433353	29.07027742	37.94820224	27.23797079	23.48243974	39.17699935
	TEA027265.1	38.85460933	26.13253086	29.0891499	42.01135021	54.95946531	42.79382051	40.02198967	43.52999928
	TEA006319.1	101.6332593	30.40091119	26.82270027	131.6389921	90.6339308	59.85563667	33.96042578	79.29570729
	TEA006473.1	3.846817108	55.97102017	0.950446619	4.028016034	6.292218419	0.235035223	7.765460682	7.226172065
	TEA014429.1	17.36337311	11.45934578	16.10275907	15.1179155	14.46839014	10.3502548	16.09888696	19.93923808
	TEA031711.1	14.7110015	21.66328557	13.29711376	15.64070056	25.22455703	9.514574007	27.67363416	25.37808567
	TEA001470.1	28.7896495	19.97601994	31.10884897	30.46722766	23.99952335	32.16500545	28.52558256	21.45750295
	TEA017119.1	0.895834121	113.9506899	13.55300324	1.465512217	1.69834214	6.023865703	17.98884145	1.787324474
	TEA005306.1	7.834157215	7.592695361	12.82189045	7.456114787	12.5937174	11.53413592	31.316448	11.25245677
	TEA009902.1	3.205680923	4.53954802	4.16734287	1.791181598	3.211073117	0.809565766	5.993016315	3.901364173
	TEA029598.1	2.441587114	55.45881453	15.49959102	3.625214431	0.825969674	4.927034664	19.80045586	3.411291334
	TEA005122.1	13.35846763	10.73623193	1.261169553	4.850759735	0.528991814	2.87265272	54.62262255	3.613086033
	TEA028214.1	25.67179545	0.110475726	1.535336847	4.250842453	0.343380651	0.113165107	50.53914575	1.066629121
**ZIK**	**Gene**	**Apical bud**	**Flower**	**Fruit**	**Young leaf**	**Mature leaf**	**Old leaf**	**Root**	**Stem**
	TEA010125.1	0.175653749	0.311340683	3.271729709	1.285537032	55.12651535	63.85123545	38.26913032	1.595139046
	TEA022762.1	8.712425961	39.90182364	106.7790221	16.3606013	56.88054084	149.1342012	73.39486483	40.65682714
	TEA024720.1	4.101515044	28.42239136	53.59970599	1.311247773	28.02728558	14.84203905	15.95199931	55.25331034
	TEA002087.1	49.7275764	172.9447277	93.96627059	40.68296195	46.43063238	56.80017877	15.38403371	115.6764087
	TEA013346.1	30.90627718	15.87837482	8.352963559	20.58573301	10.74688633	3.133802967	9.498735008	24.67660886
	TEA013344.1	1.027574433	4.660066994	0.091389098	0.685619751	0.204172279	0	1.752859347	0.134529799
	TEA031068.1	1.378881931	0.220951452	0.612306957	1.156983329	0.733164093	0.156690148	0.548380578	1.354907262
	TEA020698.1	2.977331049	1.43618444	9.815189128	6.290561211	12.12040893	9.314358818	0.127302634	12.22299317
	TEA027328.1	7.359892092	8.225419974	2.083671435	5.424966276	3.368842605	1.906396805	3.917004127	4.074331058
	TEA020112.1	5.779008349	3.886736911	6.534320509	6.547668617	3.619417675	1.915101813	182.9926403	13.87578785
	TEA033250.1	6.903192344	12.27284885	12.91327955	8.038891575	16.38018512	22.98992676	6.580566934	18.54589373
**Raf**	**Gene**	**Apical bud**	**Flower**	**Fruit**	**Young leaf**	**Mature leaf**	**Old leaf**	**Root**	**Stem**

	TEA001765.1	5.945879411	9.259874501	9.431354916	5.536379485	5.98596	2.977112818	5.728618536	8.792483297
	TEA002020.1	2.099062303	1.737481875	0.749390604	2.245404683	2.329420093	7.22515684	40.62912531	1.825761559
	TEA000256.1	16.06353537	18.80095994	27.72745234	17.10621278	21.7721894	20.3871293	22.09190328	24.72465522
	TEA029086.1	6.595798283	9.028879801	6.104791748	8.10745355	8.046243908	10.9857204	5.944053763	9.686145534
	TEA022129.1	7.948332152	5.353051095	9.26685454	8.441693178	7.03466307	10.71586514	7.4031378	9.503569378
	TEA019143.1	16.39727749	45.06405302	10.51888518	16.51486574	18.67248298	7.616882211	6.179074011	16.39341695
	TEA028452.1	11.81271464	4.288466824	9.001826155	13.86665945	35.21971814	11.12500053	16.57871997	16.22045006
	TEA016969.1	39.46939745	9.048966297	43.80279468	47.81340735	55.87824056	10.1413346	16.76477766	51.99576735
	TEA013270.1	5.5330931	5.152186138	4.414093435	5.416396029	7.062504745	5.571205274	4.377252112	9.196072695
	TEA026716.1	28.83356293	25.19850881	12.17302786	36.414979	15.10874865	13.64074791	14.1403849	26.99244326
	TEA028758.1	1.370099244	4.157904602	1.745531772	1.336958513	2.023161675	1.610426525	1.419913996	2.825125781
	TEA010804.1	18.18016304	14.33171466	21.29365984	14.05520489	21.40096707	12.40463674	8.049443481	18.3537083
	TEA009451.1	10.8554017	3.806390928	7.420794759	7.079023924	10.33854177	2.533157398	6.844964712	16.00904609
	TEA021421.1	34.09439272	72.13060593	75.65189534	26.30208768	65.85484056	81.49628715	67.88168152	69.58073394
	TEA017670.1	15.46631262	6.849495021	18.10418032	16.56628722	17.01126307	12.47427681	14.66918046	23.49466848
	TEA019184.1	3.881947858	3.454877254	6.570876148	4.627933316	20.52859461	18.3762724	6.844964712	5.794390632
	TEA000933.1	173.0804218	231.3462138	226.0600729	158.0096418	223.3273511	167.2406183	203.615667	211.3559236
	TEA031230.1	5.322308601	3.434790759	2.275588541	6.684792567	12.62155907	11.47320086	1.664726754	6.246026386
	TEA022171.1	40.19836051	38.11412552	23.52355383	34.65807839	25.89275721	60.12549192	46.96487948	40.11870794
	TEA011280.1	23.13359877	21.09082045	8.526602846	18.80312166	11.54501433	5.754010447	18.94850747	13.81813222
	TEA031223.1	2.76654655	32.00783084	1.919171058	3.633784678	3.573014884	1.558196475	3.025885688	6.591960155
	TEA007232.1	59.30948842	302.311803	51.26928399	49.37319228	66.39311293	22.04108087	28.29056231	89.49114421
	TEA016553.1	39.28496101	21.76371805	19.83143427	50.88155573	32.12001172	17.50577157	22.01356319	30.10584718
	TEA033032.1	9.327214084	8.576933649	8.279852281	7.370412318	7.025382512	5.162069887	6.120318949	15.23069511
	TEA001764.1	13.75368856	12.2628056	12.09991658	10.38713922	9.336241489	8.617958159	9.410602416	22.09171486
	TEA026000.1	6.270838847	17.98745687	25.56153072	7.113304911	20.29658065	48.93955633	13.17092638	13.21274812
	TEA033556.1	65.12362752	55.64963624	51.08650579	65.63095062	65.98476838	77.85759371	27.82052181	65.18929693
	TEA013875.1	28.04312106	64.58812681	39.13281177	39.92878022	99.37621657	52.4999047	22.19962089	47.29683365
	TEA002722.1	39.91731451	87.0749587	87.96200685	33.9296074	82.20718401	62.319154	65.45313897	88.66474687
	TEA030052.1	20.40218297	36.29629767	36.99430688	18.76884067	35.46101265	14.53736376	29.12292569	46.7106681
	TEA008343.1	80.28254608	72.49216285	87.93459012	65.87091753	186.0102268	154.6531764	35.85038027	73.57819083

Supplementary Table S8: Expression data of tea MAPKKKs under cold stress

MEKK	Gene	CK	CA1-6h	CA1-7d	CA2-7d	DA-7d
	TEA028357.1	1.271072595	1.55171842	1.645887675	1.562930846	3.949646057
	TEA025870.1	1.321020027	3.211394941	3.285005403	3.240964525	5.840108057
	TEA016319.1	25.83248455	37.96145705	57.65756766	54.34010206	22.55043625
	TEA008165.1	14.23324926	22.01910122	22.20661545	20.59298427	25.29883386
	TEA027265.1	27.13755191	43.06801379	43.43988621	50.33351452	32.58297138
	TEA006319.1	19.59668365	59.79544209	24.78367394	28.88540925	18.54500203
	TEA006473.1	0.979312377	0.821693392	0.315717982	0.243672007	5.066356705
	TEA014429.1	13.1429846	17.32021333	13.0728358	11.3941215	16.56420423
	TEA031711.1	35.14793487	37.72018613	39.94401141	24.69761268	49.37117673
	TEA001470.1	24.08189229	55.92295268	35.76363426	34.12262383	33.69491635
	TEA017119.1	1.517039928	2.363361085	3.684550886	14.025674	1.617121236
	TEA005306.1	14.80137642	17.36384872	12.98211323	12.93104296	19.88123095
	TEA009902.1	3.272419096	6.052180734	5.041938668	4.75777809	3.670281021
	TEA029598.1	1.765362021	3.913949118	2.454145731	9.155077523	1.653309296
	TEA005122.1	3.012555366	5.967656907	3.296042789	2.733067624	4.770304716
	TEA028214.1	2.525109113	2.389189836	2.438156009	0.200257988	5.667341535
	TEA031689.1	0	0	0	0	0
**ZIK**	**Gene**	**CK**	**CA1-6h**	**CA1-7d**	**CA2-7d**	**DA-7d**
	TEA010125.1	171.8985422	198.6455799	67.03776552	58.4380675	134.4104312
	TEA022762.1	34.69843009	40.57471889	41.80350807	55.99906299	52.72398141
	TEA024720.1	17.99146738	17.73906451	10.55262381	5.150869612	11.51088597
	TEA002087.1	16.2822216	18.40246837	19.78023441	21.45024694	29.34570263
	TEA013346.1	1.462217149	1.348576814	1.487123171	0.941019794	2.648841558
	TEA013344.1	0.035063252	0.037266937	0	0.040398429	0.083085608
	TEA031068.1	0.024118043	0.02436739	0.024563716	0	0.083886441
	TEA020698.1	10.16986316	2.418859474	9.686900219	11.30147051	4.049612215
	TEA027328.1	2.309864204	1.904314294	1.998636221	1.446316947	9.453017073
	TEA020112.1	7.383707412	7.956495028	5.684372506	2.646901124	17.44338035
	TEA033250.1	14.81022059	18.34825838	22.21544009	25.31524634	15.73664453
**Raf**	**Gene**	**CK**	**CA1-6h**	**CA1-7d**	**CA2-7d**	**DA-7d**
	TEA001765.1	5.98819739	7.330697821	5.140419023	4.202133859	9.682731356
	TEA002020.1	1.068593135	3.296417112	2.710732035	3.767767608	1.022671186
	TEA000256.1	23.62431721	30.55547298	20.7512083	27.01989621	28.52945611
	TEA029086.1	7.718669173	13.14653029	10.99510709	11.88940635	10.26734035
	TEA022129.1	5.674218414	10.54603089	9.152495787	10.96184933	9.683353581
	TEA019143.1	13.2198183	11.6500307	11.6662276	8.007975589	19.03868128
	TEA028452.1	2.514153402	1.715345076	1.703142471	1.25401883	1.230877684
	TEA016969.1	44.37837064	23.30300284	11.2324109	5.973580018	58.38198957
	TEA013270.1	3.201444658	3.683921302	3.970311189	4.303720378	5.650230338
	TEA026716.1	8.640684616	11.11269545	6.565005785	8.652903638	13.33303046
	TEA028758.1	1.680015812	1.371585673	0.904885236	1.022729242	2.204912061
	TEA010804.1	8.511581891	23.11970987	9.737885568	9.012692201	5.857487942
	TEA009451.1	5.984659723	7.485488006	6.002678611	7.565958819	10.42732297
	TEA021421.1	47.53805977	58.44955147	87.13950661	79.01034897	63.63781389

	TEA017670.1	9.351324472	6.439381057	10.72772048	11.24945174	21.70647242
	TEA019184.1	14.71580911	28.51178066	24.17424689	22.25515769	19.77361425
	TEA000933.1	106.1721217	167.9367145	115.8115543	120.9495902	126.4237447
	TEA031230.1	9.796163951	10.94440139	4.363310632	2.56082981	11.94556694
	TEA022171.1	15.56285918	39.20719289	47.45799534	47.82859418	21.87390761
	TEA011280.1	23.42576566	16.28791413	11.9340752	8.687800951	20.84016505
	TEA031223.1	1.765129862	2.119792927	3.375016737	2.308172311	2.679825552
	TEA007232.1	35.03130242	32.03445763	19.84464111	15.19645328	42.76484059
	TEA016553.1	22.13849719	32.98228163	19.91892615	19.89991625	20.7530535
	TEA033032.1	6.035237302	4.686259154	4.034151537	3.959268547	14.57110333
	TEA001764.1	7.838983007	4.947354399	4.516451075	4.489450709	2.202479725
	TEA026000.1	17.75565979	21.38559793	25.23728681	37.1521342	14.02252684
	TEA033556.1	33.90068625	45.68710989	29.27805024	30.2740439	42.83568943
	TEA013875.1	38.54431602	44.93704297	37.60008149	38.37359965	20.93364027
	TEA002722.1	57.00983074	65.66057405	70.59435912	66.17318251	79.07041648
	TEA030052.1	15.72360191	14.98869757	27.011303	23.59953118	26.51966826
	TEA008343.1	82.93727953	124.9005461	156.5899552	121.0959376	51.44544975

Supplementary Table S9: Expression data of tea MAPKKKs under drought stress

MEKK	Gene	N-0h	PEG-N-24h	PEG-N-48h	PEG-N-72h
	TEA028357.1	1.969760999	1.007381388	0.755394409	1.762346314
	TEA025870.1	1.359530902	0.242087427	1.016585514	0.398656363
	TEA016319.1	25.72727117	42.39527597	42.53548502	43.89221425
	TEA008165.1	8.655158588	8.458566691	9.73334579	12.38867232
	TEA027265.1	14.65454244	16.31446877	20.07384967	14.20478351
	TEA006319.1	15.83294983	44.29714607	31.23570374	17.36990608
	TEA006473.1	0.393086314	0.870839902	0.783628993	1.12071024
	TEA014429.1	8.288017307	11.21299062	10.55392409	9.167519726
	TEA031711.1	54.80053122	31.60098573	30.23921131	10.74219789
	TEA001470.1	27.05864215	71.11695005	52.85332582	73.92211168
	TEA017119.1	1.054000831	1.316463151	4.035825978	1.081105046
	TEA005306.1	22.91729997	109.1272767	53.5457457	35.37724722
	TEA009902.1	4.134833705	85.58677092	23.99047618	23.68378626
	TEA029598.1	3.366904888	22.13577483	28.34391434	48.66778839
	TEA005122.1	93.71449651	315.2021484	252.0221097	421.7489153
	TEA028214.1	188.5251126	237.5176148	130.9599842	12.21105265
	TEA031689.1	0.15952941	0	0	0
**ZIK**	**Gene**	**N-0h**	**PEG-N-24h**	**PEG-N-48h**	**PEG-N-72h**
	TEA010125.1	63.9128316	4.461579293	10.97038833	2.132174407
	TEA022762.1	64.39365622	55.96012156	54.26322025	72.36055542
	TEA024720.1	246.269942	62.77468207	55.74536829	44.93258652
	TEA002087.1	30.93692137	7.633918638	11.31252372	3.915655465
	TEA013346.1	4.343434751	1.588774818	1.788523595	0.678359439
	TEA013344.1	0	0	0	0
	TEA031068.1	0.27620321	0.068879938	0.110185237	0
	TEA020698.1	2.802401872	0.227958209	0.911625441	0.312565708
	TEA027328.1	0.98741938	0.442875901	1.259553778	0.89190817
	TEA020112.1	171.5901655	110.985121	106.6031785	34.95984101
	TEA033250.1	9.210945403	17.29668832	13.79246755	20.44711689
**Raf**	**Gene**	**N-0h**	**PEG-N-24h**	**PEG-N-48h**	**PEG-N-72h**
	TEA001765.1	3.479068628	3.006349474	3.976947511	2.380472729
	TEA002020.1	1.056631461	1.739429596	1.880200899	1.200808726
	TEA000256.1	11.29211034	24.95718171	20.02247495	25.15743077
	TEA029086.1	4.191646704	5.259402309	6.56171601	6.211858639
	TEA022129.1	7.034031279	16.74847011	13.89572314	11.39060946
	TEA019143.1	8.593743779	4.957694587	6.472759289	5.879021339
	TEA028452.1	0.663591021	0.251483016	0.25864135	0.177716458
	TEA016969.1	15.1300628	8.865742995	12.76067081	8.601691326
	TEA013270.1	2.514365846	1.871791473	2.271749664	1.117706671

	TEA026716.1	7.509078552	4.637750846	6.713435936	5.628268273
	TEA028758.1	0.316071227	0.295140252	0.314786581	0.216328778
	TEA010804.1	4.116971228	11.54352558	7.037982449	3.659583787
	TEA009451.1	4.9105899	7.049501419	7.643457577	7.603418912
	TEA021421.1	42.22368444	19.76224823	26.06786236	16.52631245
	TEA017670.1	10.41236184	8.059421971	8.72159279	8.636995729
	TEA019184.1	16.86083074	3.519494913	2.68398751	0.875098165
	TEA000933.1	178.127316	262.5292884	260.4407308	247.6477156
	TEA031230.1	1.541118894	0.845792962	1.168463614	0.442777886
	TEA022171.1	43.19522543	97.82435736	52.46447232	49.51317817
	TEA011280.1	9.573341516	4.283269017	5.006896779	9.009747561
	TEA031223.1	1.126609076	0.858800489	1.061540925	1.798229478
	TEA007232.1	59.64117648	53.60036014	80.18619578	131.9165291
	TEA016553.1	25.89901473	53.85962333	33.02546742	29.73582711
	TEA033032.1	6.710212956	5.146129665	3.697271629	4.016000597
	TEA001764.1	1.349088665	0.801414477	0.833271399	0.659170528
	TEA026000.1	5.510602832	5.2013269	8.372396535	8.920418839
	TEA033556.1	30.56017507	36.12756853	50.80668854	77.62208897
	TEA013875.1	30.58856006	14.52658801	31.55280978	33.71331019
	TEA002722.1	34.72574485	28.62156368	36.60689285	36.3817962
	TEA030052.1	18.6624178	28.49437797	37.62173339	78.31829819
	TEA008343.1	80.76821471	38.7553528	68.40607351	18.73308712

Supplementary Table S10: Expression data of tea MAPKKKs under salt stress

MEKK	Gene	N-0h	NaCl-N-24h	NaCl-N-48h	NaCl-N-72h
	TEA028357.1	1.969755742	1.014062774	1.381679455	0.559062736
	TEA025870.1	1.359530422	0.324980117	0.581132907	0.268773073
	TEA016319.1	25.72724067	34.69530182	34.58511306	19.27636458
	TEA008165.1	8.655145753	21.32454464	13.51012975	6.252564685
	TEA027265.1	14.65449931	23.07523008	15.00398821	7.576675511
	TEA006319.1	15.83303294	31.89324732	20.68181694	14.86963468
	TEA006473.1	0.39308625	0.162434187	0.522774756	0.172764494
	TEA014429.1	8.2880152	8.174044487	8.557338026	5.103081623
	TEA031711.1	54.80064905	45.50419113	31.83294847	9.582807161
	TEA001470.1	27.05851483	68.69865495	59.44146826	46.30786188
	TEA017119.1	1.054001329	2.609057914	0.450340554	1.453704651
	TEA005306.1	22.91733926	57.71466026	39.75845275	59.36188402
	TEA009902.1	4.134827152	6.362870333	15.85473173	41.44379162
	TEA029598.1	3.366901976	20.95456391	16.33092471	35.5458555
	TEA005122.1	93.714391	48.41995361	288.4361818	606.34795
	TEA028214.1	188.5246115	50.80376902	144.3694997	41.68165584
	TEA031689.1	0.159530122	0	0	0
**ZIK**	**Gene**	**N-0h**	**NaCl-N-24h**	**NaCl-N-48h**	**NaCl-N-72h**
	TEA010125.1	63.91280784	14.89255311	9.560445924	2.517514419
	TEA022762.1	64.39359831	97.8907851	70.79303025	49.01031994
	TEA024720.1	246.2689738	75.34516899	39.01729443	51.00146277
	TEA002087.1	30.93687186	15.37034767	17.37018455	4.520938466
	TEA013346.1	4.343436632	3.862277064	2.946402181	2.875044586
	TEA013344.1	0	0	0	0
	TEA031068.1	0.276204077	0.246428285	0.188888641	0.305609131
	TEA020698.1	2.802402712	0.724572809	0.833203682	0.096249817
	TEA027328.1	0.987420149	1.380974858	0.810462582	0.500123019
	TEA020112.1	171.5906529	23.45319246	32.57249342	48.77785406
	TEA033250.1	9.210952349	5.895378257	7.948215595	12.71063731
**Raf**	**Gene**	**N-0h**	**NaCl-N-24h**	**NaCl-N-48h**	**NaCl-N-72h**
	TEA001765.1	3.479076711	3.864008471	3.489897599	2.054607029
	TEA002020.1	1.056632427	1.993327958	2.000444887	0.418171204
	TEA000256.1	11.29211112	18.58879933	20.52457522	10.95778044
	TEA029086.1	4.191642549	7.43439002	6.090318338	3.419365645
	TEA022129.1	7.034043184	9.834193319	7.876966601	7.949337891
	TEA019143.1	8.593743773	25.12000905	15.6415184	6.374305617
	TEA028452.1	0.663591315	0.386491715	0.246708174	0.068562337
	TEA016969.1	15.13004797	13.90282704	15.27714452	5.467393451
	TEA013270.1	2.514365249	2.64313347	1.99490978	1.170663099

	TEA026716.1	7.509227828	7.870999205	4.498652299	2.242578807
	TEA028758.1	0.316070955	0.470489497	0.382891672	0.08347364
	TEA010804.1	4.116975877	3.872112928	3.987014775	2.450785914
	TEA009451.1	4.910600483	5.631946577	7.640955084	4.018015904
	TEA021421.1	42.2236538	23.40972311	23.336931	8.438106689
	TEA017670.1	10.41237522	31.36375615	13.61673641	4.624932366
	TEA019184.1	16.86086454	5.266042637	4.261821685	0.932486788
	TEA000933.1	178.1268988	243.0538836	211.7426775	219.444926
	TEA031230.1	1.541106723	1.374650926	1.211927889	0.389542305
	TEA022171.1	43.19528154	41.40395002	62.98542624	76.15551971
	TEA011280.1	9.573350712	3.582010217	5.530391346	17.51633805
	TEA031223.1	1.126609135	0.790883228	0.588496089	0.395481319
	TEA007232.1	59.64116945	47.14411566	64.69696582	46.51924051
	TEA016553.1	25.89890995	42.92267609	48.54748342	35.69844286
	TEA033032.1	6.710215806	5.349948323	4.122202946	4.248339322
	TEA001764.1	1.349089028	1.050462836	0.768761877	0.761002711
	TEA026000.1	5.510598449	3.338446735	4.339623452	2.233907729
	TEA033556.1	30.56018956	55.4215902	47.57958427	71.4634371
	TEA013875.1	30.5885827	30.58339521	22.56127133	33.03352011
	TEA002722.1	34.72582691	33.82542347	29.02133733	17.10893231
	TEA030052.1	18.66244554	12.84224837	24.23905421	19.03251158
	TEA008343.1	80.76828496	70.0572566	61.09505056	33.26337117

Supplementary Table S11: Expression data of tea MAPKKKs under MeJA treatment

MEKK	Gene	CK	12h_MeJA	24h_MeJA	48h_MeJA
	TEA028357.1	8.048422021	3.56286023	2.839287487	5.528577132
	TEA025870.1	14.53557989	12.25822641	9.139104694	6.828215876
	TEA016319.1	42.74652531	19.67998631	20.07019515	38.89322812
	TEA008165.1	32.26873397	27.84660988	28.56353058	22.23714971
	TEA027265.1	44.69974942	41.30224131	64.1161308	24.63706572
	TEA006319.1	32.08544754	24.48233954	20.00415114	23.26617171
	TEA006473.1	11.93705767	1.05795052	1.052881437	14.04771839
	TEA014429.1	23.90115051	16.04747595	16.78526183	12.45573579
	TEA031711.1	26.30102086	31.12006087	25.97231146	23.89882652
	TEA001470.1	37.48249226	26.73502849	27.10274797	31.49139386
	TEA017119.1	6.019369596	0.984618838	2.555600732	14.67466825
	TEA005306.1	15.35347688	11.51450952	12.04431015	17.40368396
	TEA009902.1	4.58197777	6.495389355	5.997754084	5.179424903
	TEA029598.1	7.099960497	6.254316048	15.07757041	13.49690567
	TEA005122.1	7.0569606	9.473209524	15.76876384	26.24268631
	TEA028214.1	48.69725821	11.82583931	19.10146563	256.8482043
	TEA031689.1	0	0	0	0.100254359
**ZIK**	**Gene**	**CK**	**12h_MeJA**	**24h_MeJA**	**48h_MeJA**
	TEA010125.1	8.156196443	38.02219965	43.28353065	1.263135498
	TEA022762.1	6.926163005	15.89536342	27.1926132	5.395674208
	TEA024720.1	10.77133263	10.09665998	7.120514873	10.61570085
	TEA002087.1	38.46817708	30.09573988	25.09369924	42.59109409
	TEA013346.1	3.503068243	1.846216342	1.508299006	3.809808121
	TEA013344.1	0.092411156	0.428701603	0.48522434	0.048939781
	TEA031068.1	0.298983293	0.085973366	0.251785229	0.165275039
	TEA020698.1	7.383813086	7.270780974	8.35369772	6.902720985
	TEA027328.1	7.386360119	3.56494288	3.534892251	6.065113259
	TEA020112.1	25.26755837	17.70805965	17.50846896	41.13476756
	TEA033250.1	13.00388128	14.40139171	15.9260595	12.02644445
**Raf**	**Gene**	**CK**	**12h_MeJA**	**24h_MeJA**	**48h_MeJA**
	TEA001765.1	13.87568213	10.02475583	11.85419415	9.840038798
	TEA002020.1	1.712671071	2.110198798	1.648239207	1.263105696
	TEA000256.1	26.49629333	20.82003937	21.90757987	19.02633429
	TEA029086.1	13.96587703	10.83302168	11.25124006	10.42677008
	TEA022129.1	9.896268919	7.089274088	7.826832884	8.583286614
	TEA019143.1	26.93195236	24.88713812	25.46525982	22.62982712
	TEA028452.1	2.577813013	2.714773587	2.839589979	1.680395331
	TEA016969.1	42.36746698	35.87206143	39.54322136	33.8250358
	TEA013270.1	8.491189444	5.463027747	4.812264087	5.51181703
	TEA026716.1	22.30650857	17.24486778	19.28661573	17.50046965

	TEA028758.1	3.306630465	1.646664218	1.446754558	2.637367338
	TEA010804.1	4.932669147	6.721488674	7.36470126	5.256966982
	TEA009451.1	16.8122438	11.32127125	18.32128924	9.521412376
	TEA021421.1	84.08585693	56.06071519	62.36407394	48.69497839
	TEA017670.1	23.18332025	12.39491678	11.31023853	18.27787725
	TEA019184.1	11.30446143	7.682143858	9.8064643	7.001152878
	TEA000933.1	150.7615002	134.3902295	130.9309913	164.4405816
	TEA031230.1	7.005254181	7.099977326	4.797681469	6.177779176
	TEA022171.1	16.11006197	16.65500335	14.60215437	18.1197845
	TEA011280.1	24.74133819	35.5889265	30.11858975	18.35323384
	TEA031223.1	3.494727961	2.401558872	2.076592595	2.57736589
	TEA007232.1	44.69225815	47.60990219	41.35577655	78.45756989
	TEA016553.1	23.15152397	22.54376163	21.88627941	22.50210407
	TEA033032.1	7.499428372	8.48843251	7.115448138	6.811526732
	TEA001764.1	0.520881405	0.929503751	0.52997672	0.382455304
	TEA026000.1	17.83388672	17.07377414	20.57573432	8.066093432
	TEA033556.1	44.57988908	29.8974927	31.49656527	38.19217052
	TEA013875.1	13.70674564	15.18502166	14.93325669	10.34702833
	TEA002722.1	87.76740055	66.08419243	72.90061445	52.86485197
	TEA030052.1	51.20533581	40.34527689	45.85168479	30.4362596
	TEA008343.1	36.005713	50.8398548	61.96503704	34.75187936
