# Supplementary material for: In-silico genome wide analysis of Mitogen activated protein kinase kinase kinase gene family in C. sinensis
Source: PLoS One. 2021 Nov 4;16(11):e0258657. doi: 10.1371/journal.pone.0258657 (PMC8568164; doi:10.1371/journal.pone.0258657)
Supplement: Attachment — Submitted filename: Supporting information_2.pdf [file pone.0258657.s007.pdf]

```
# TEA028357.1 Length: 628
# TEA028357.1 Number of predicted TMHs:  0
# TEA028357.1 Exp number of AAs in TMHs: 0.02198
# TEA028357.1 Exp number, first 60 AAs:  0.00018
# TEA028357.1 Total prob of N-in:        0.00262
TEA028357.1     TMHMM2.0        outside      1   628
```

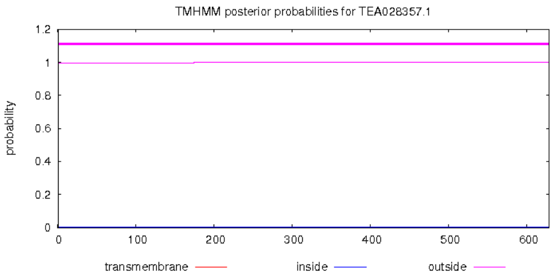


```
# TEA025870.1 Length: 776
# TEA025870.1 Number of predicted TMHs:  0
# TEA025870.1 Exp number of AAs in TMHs: 0.19882
# TEA025870.1 Exp number, first 60 AAs:  0.00141
# TEA025870.1 Total prob of N-in:        0.00426
TEA025870.1     TMHMM2.0        outside      1   776
```

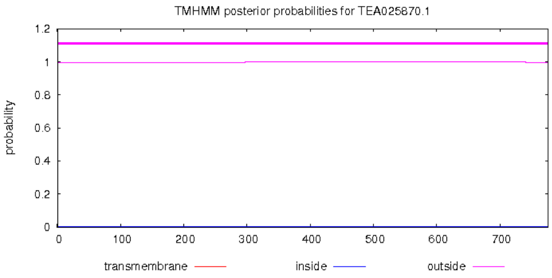


```
# TEA016319.1 Length: 627
# TEA016319.1 Number of predicted TMHs:  0
# TEA016319.1 Exp number of AAs in TMHs: 0.11408
# TEA016319.1 Exp number, first 60 AAs:  3e-05
# TEA016319.1 Total prob of N-in:        0.00653
TEA016319.1     TMHMM2.0        outside      1   627
```

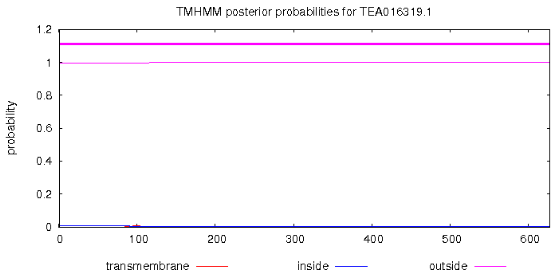


```
# TEA029598.1 Length: 423
# TEA029598.1 Number of predicted TMHs:  0
# TEA029598.1 Exp number of AAs in TMHs: 0.02545
# TEA029598.1 Exp number, first 60 AAs:  0.00568
# TEA029598.1 Total prob of N-in:        0.01343
TEA029598.1     TMHMM2.0        outside      1   423
```

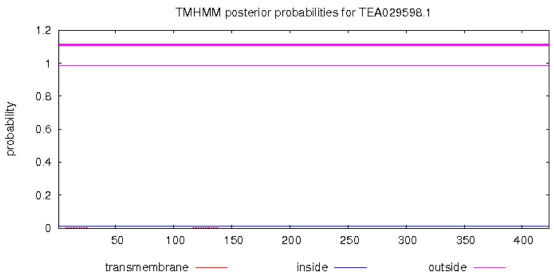


```
# TEA008165.1 Length: 1032
# TEA008165.1 Number of predicted TMHs:  0
# TEA008165.1 Exp number of AAs in TMHs: 6.76661
# TEA008165.1 Exp number, first 60 AAs:  3.17779
# TEA008165.1 Total prob of N-in:        0.04338
TEA008165.1     TMHMM2.0        outside      1  1032
```

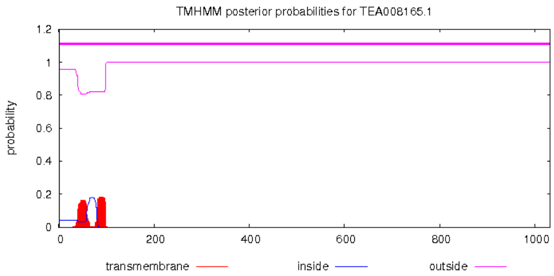


```
# TEA027265.1 Length: 939
# TEA027265.1 Number of predicted TMHs:  0
# TEA027265.1 Exp number of AAs in TMHs: 0
# TEA027265.1 Exp number, first 60 AAs:  0
# TEA027265.1 Total prob of N-in:        0.00001
TEA027265.1     TMHMM2.0        outside      1   939
```

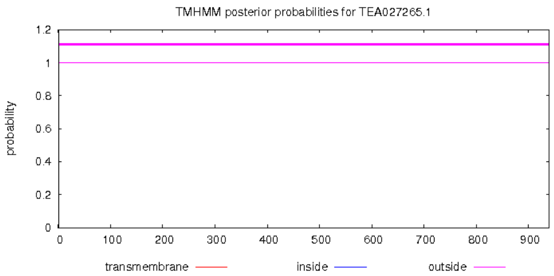


```
# TEA006319.1 Length: 683
# TEA006319.1 Number of predicted TMHs:  0
# TEA006319.1 Exp number of AAs in TMHs: 0.00042
# TEA006319.1 Exp number, first 60 AAs:  0
# TEA006319.1 Total prob of N-in:        0.00017
TEA006319.1     TMHMM2.0        outside      1   683
```

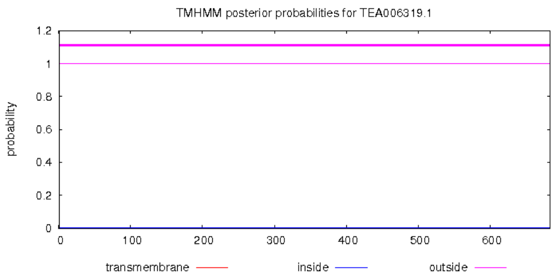


```
# TEA006473.1 Length: 710
# TEA006473.1 Number of predicted TMHs:  0
# TEA006473.1 Exp number of AAs in TMHs: 0.00515
# TEA006473.1 Exp number, first 60 AAs:  0
# TEA006473.1 Total prob of N-in:        0.00031
TEA006473.1     TMHMM2.0        outside      1   710
```

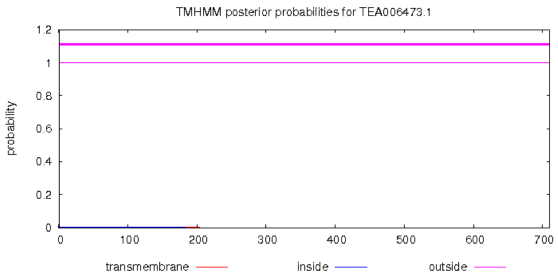

TEA014429.1 Length: 1191
TEA014429.1 Number of predicted TMHs: 0
TEA014429.1 Exp number of AAs in TMHs: 0.02164
TEA014429.1 Exp number, first 60 AAs: 0.00074
TEA014429.1 Total prob of N-in: 0.00023
TEA014429.1   TMHMM2.0   outside   1   1191

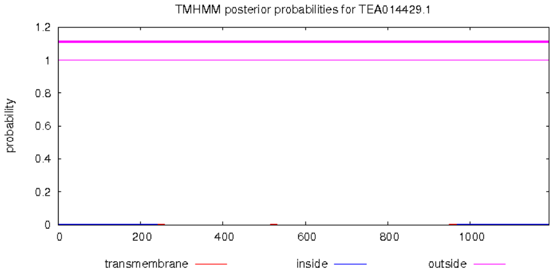

TEA031711.1 Length: 562
TEA031711.1 Number of predicted TMHs: 0
TEA031711.1 Exp number of AAs in TMHs: 0.0069899
TEA031711.1 Exp number, first 60 AAs: 0.00016
TEA031711.1 Total prob of N-in: 0.00833
TEA031711.1   TMHMM2.0   outside   1   562

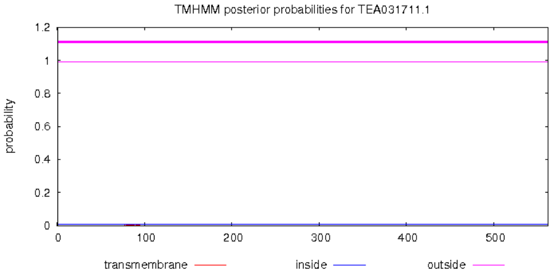

TEA001470.1 Length: 789
TEA001470.1 Number of predicted TMHs: 0
TEA001470.1 Exp number of AAs in TMHs: 3.06197
TEA001470.1 Exp number, first 60 AAs: 0.21965
TEA001470.1 Total prob of N-in: 0.01032
TEA001470.1   TMHMM2.0   outside   1   789

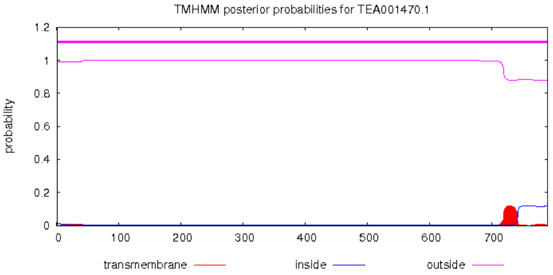

TEA017119.1 Length: 506
TEA017119.1 Number of predicted TMHs: 0
TEA017119.1 Exp number of AAs in TMHs: 0.04644
TEA017119.1 Exp number, first 60 AAs: 0.00226
TEA017119.1 Total prob of N-in: 0.01372
TEA017119.1   TMHMM2.0   outside   1   506

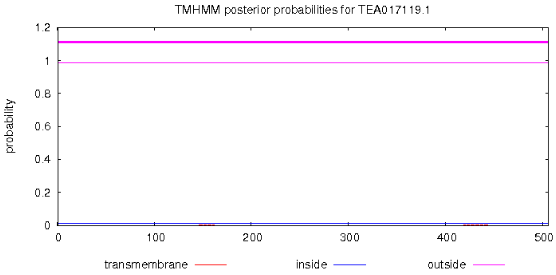

TEA005306.1 Length: 1097
TEA005306.1 Number of predicted TMHs: 0
TEA005306.1 Exp number of AAs in TMHs: 0.03434
TEA005306.1 Exp number, first 60 AAs: 0
TEA005306.1 Total prob of N-in: 0.00020
TEA005306.1   TMHMM2.0   outside   1   1097

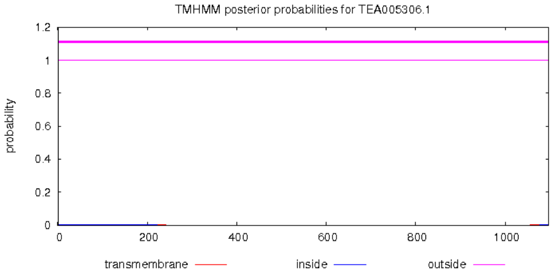

TEA009902.1 Length: 450
TEA009902.1 Number of predicted TMHs: 0
TEA009902.1 Exp number of AAs in TMHs: 0.71758
TEA009902.1 Exp number, first 60 AAs: 0.10658
TEA009902.1 Total prob of N-in: 0.00748
TEA009902.1   TMHMM2.0   outside   1   450

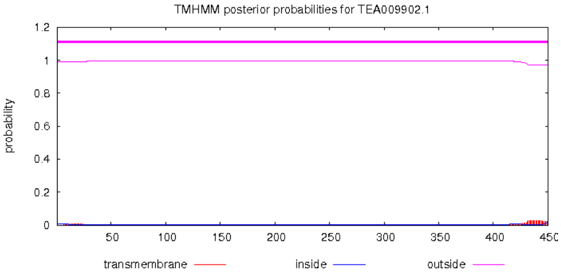

TEA005122.1 Length: 334
TEA005122.1 Number of predicted TMHs: 0
TEA005122.1 Exp number of AAs in TMHs: 0.01241
TEA005122.1 Exp number, first 60 AAs: 0.00929
TEA005122.1 Total prob of N-in: 0.04268
TEA005122.1   TMHMM2.0   outside   1   334

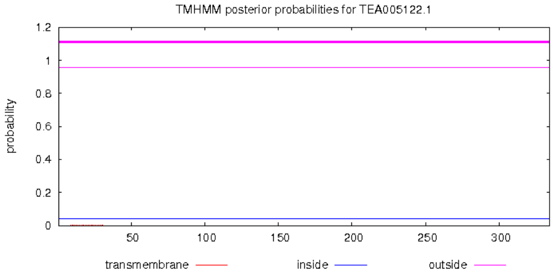

TEA028214.1 Length: 344
TEA028214.1 Number of predicted TMHs: 0
TEA028214.1 Exp number of AAs in TMHs: 0.00766
TEA028214.1 Exp number, first 60 AAs: 0.00225
TEA028214.1 Total prob of N-in: 0.02030
TEA028214.1   TMHMM2.0   outside   1   344

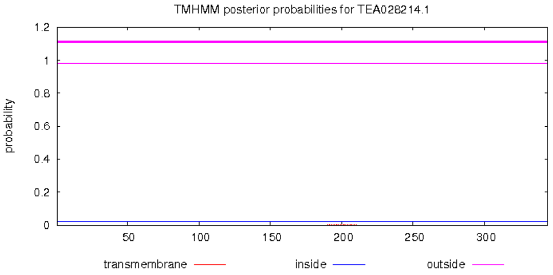

```
# TEA031689.1 Length: 311
# TEA031689.1 Number of predicted TMHs:  0
# TEA031689.1 Exp number of AAs in TMHs: 0.28379
# TEA031689.1 Exp number, first 60 AAs:  0.25957
# TEA031689.1 Total prob of N-in:        0.04973
TEA031689.1     TMHMM2.0        outside      1   311
```

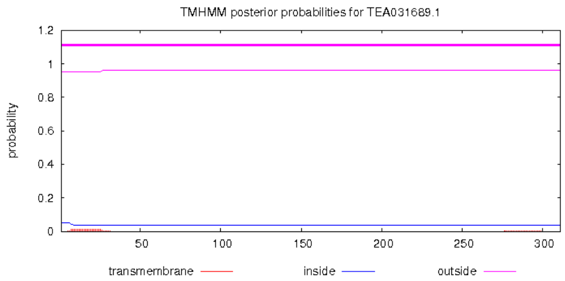

Supplementary Figure S1: Transmembrane helices of MAPKKKs of MEKK subfamily in *C. sinensis*. TMHMM Server, v.2.0 (http://www.cbs.dtu.dk/services/TMHMM/), was used to generate the transmembrane helices.

```
# TEA001765.1 Length: 842
# TEA001765.1 Number of predicted TMHs:  0
# TEA001765.1 Exp number of AAs in TMHs: 1.26294
# TEA001765.1 Exp number, first 60 AAs:  0
# TEA001765.1 Total prob of N-in:        0.00418
TEA001765.1     TMHMM2.0        outside      1   842
```

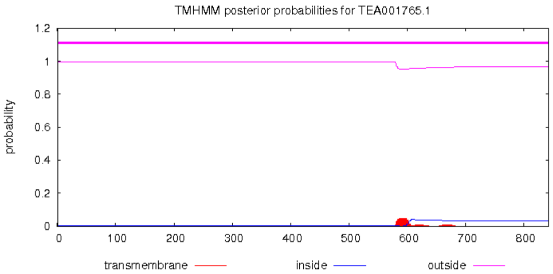


```
# TEA002020.1 Length: 896
# TEA002020.1 Number of predicted TMHs:  0
# TEA002020.1 Exp number of AAs in TMHs: 0.01584
# TEA002020.1 Exp number, first 60 AAs:  0
# TEA002020.1 Total prob of N-in:        0.00015
TEA002020.1     TMHMM2.0        outside      1   896
```

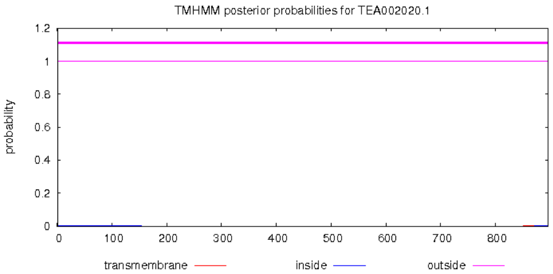


```
# TEA000256.1 Length: 1086
# TEA000256.1 Number of predicted TMHs:  0
# TEA000256.1 Exp number of AAs in TMHs: 0.00826
# TEA000256.1 Exp number, first 60 AAs:  0
# TEA000256.1 Total prob of N-in:        0.00004
TEA000256.1     TMHMM2.0        outside      1  1086
```

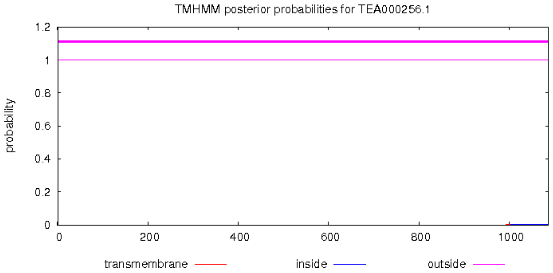


```
# TEA029086.1 Length: 919
# TEA029086.1 Number of predicted TMHs:  0
# TEA029086.1 Exp number of AAs in TMHs: 0.27701
# TEA029086.1 Exp number, first 60 AAs:  0
# TEA029086.1 Total prob of N-in:        0.00050
TEA029086.1     TMHMM2.0        outside      1   919
```

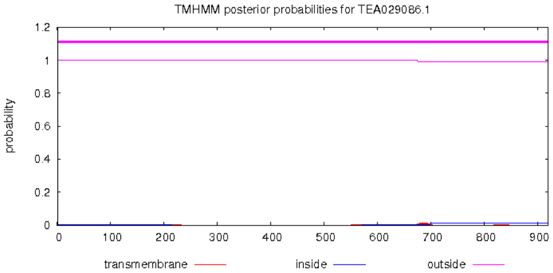


```
# TEA022129.1 Length: 940
# TEA022129.1 Number of predicted TMHs:  0
# TEA022129.1 Exp number of AAs in TMHs: 37.32592
# TEA022129.1 Exp number, first 60 AAs:  0
# TEA022129.1 Total prob of N-in:        0.17108
TEA022129.1     TMHMM2.0        outside      1   940
```

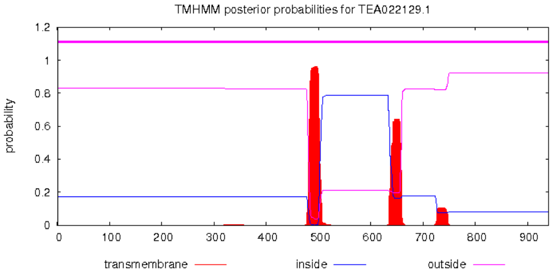


```
# TEA019143.1 Length: 724
# TEA019143.1 Number of predicted TMHs:  0
# TEA019143.1 Exp number of AAs in TMHs: 0.01226
# TEA019143.1 Exp number, first 60 AAs:  0
# TEA019143.1 Total prob of N-in:        0.00005
TEA019143.1     TMHMM2.0        outside      1   724
```

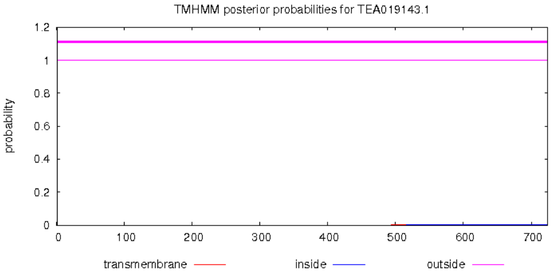


```
# TEA028452.1 Length: 846
# TEA028452.1 Number of predicted TMHs:  0
# TEA028452.1 Exp number of AAs in TMHs: 0.05099
# TEA028452.1 Exp number, first 60 AAs:  0
# TEA028452.1 Total prob of N-in:        0.00001
TEA028452.1     TMHMM2.0        outside      1   846
```

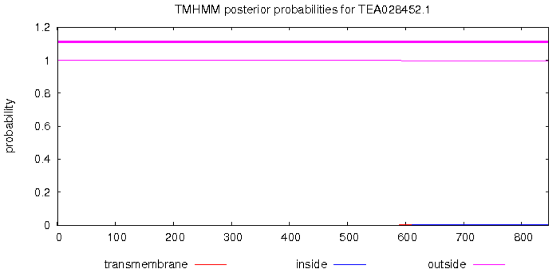


```
# TEA016969.1 Length: 1107
# TEA016969.1 Number of predicted TMHs:  0
# TEA016969.1 Exp number of AAs in TMHs: 6.33735
# TEA016969.1 Exp number, first 60 AAs:  0
# TEA016969.1 Total prob of N-in:        0.27806
TEA016969.1     TMHMM2.0        outside      1  1107
```

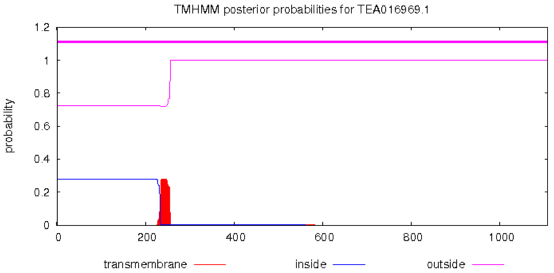

```
# TEA013270.1 Length: 755
# TEA013270.1 Number of predicted TMHs:  0
# TEA013270.1 Exp number of AAs in TMHs: 0.10844
# TEA013270.1 Exp number, first 60 AAs:  0
# TEA013270.1 Total prob of N-in:        0.00501
TEA013270.1     TMHMM2.0        outside      1   755
```

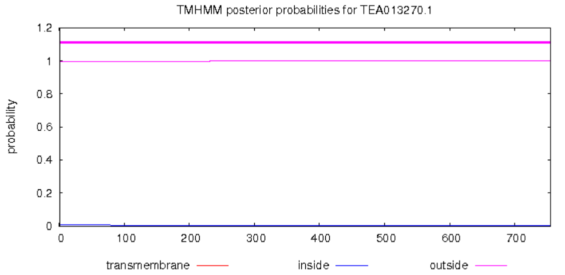


```
# TEA026716.1 Length: 368
# TEA026716.1 Number of predicted TMHs:  0
# TEA026716.1 Exp number of AAs in TMHs: 0.00933
# TEA026716.1 Exp number, first 60 AAs:  0.00015
# TEA026716.1 Total prob of N-in:        0.02057
TEA026716.1     TMHMM2.0        outside      1   368
```

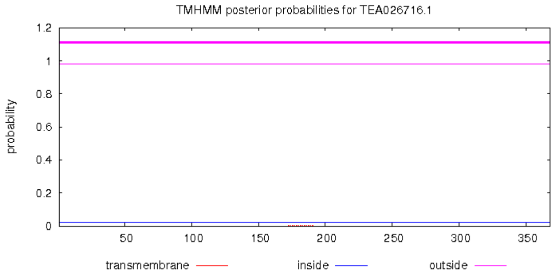


```
# TEA028758.1 Length: 1213
# TEA028758.1 Number of predicted TMHs:  0
# TEA028758.1 Exp number of AAs in TMHs: 0.0185
# TEA028758.1 Exp number, first 60 AAs:  0.00018
# TEA028758.1 Total prob of N-in:        0.00001
TEA028758.1     TMHMM2.0        outside      1  1213
```

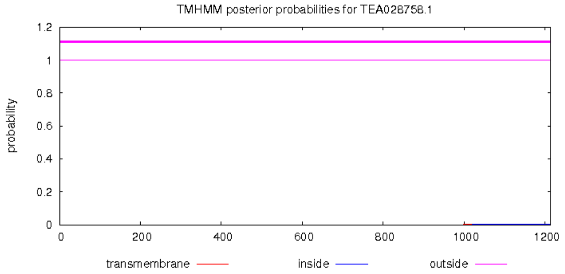


```
# TEA010804.1 Length: 305
# TEA010804.1 Number of predicted TMHs:  0
# TEA010804.1 Exp number of AAs in TMHs: 0.00132
# TEA010804.1 Exp number, first 60 AAs:  0
# TEA010804.1 Total prob of N-in:        0.01241
TEA010804.1     TMHMM2.0        outside      1   305
```

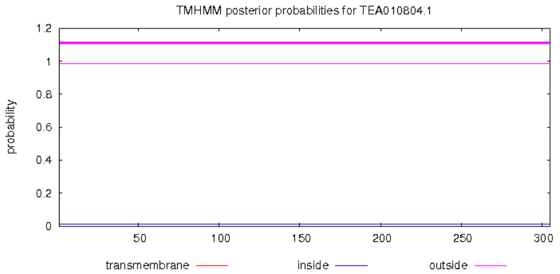


```
# TEA009451.1 Length: 1333
# TEA009451.1 Number of predicted TMHs:  0
# TEA009451.1 Exp number of AAs in TMHs: 0.02716
# TEA009451.1 Exp number, first 60 AAs:  0.02022
# TEA009451.1 Total prob of N-in:        0.00132
TEA009451.1     TMHMM2.0        outside      1  1333
```

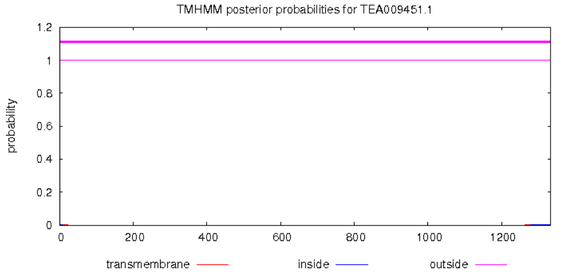


```
# TEA021421.1 Length: 1331
# TEA021421.1 Number of predicted TMHs:  0
# TEA021421.1 Exp number of AAs in TMHs: 0.01119
# TEA021421.1 Exp number, first 60 AAs:  0
# TEA021421.1 Total prob of N-in:        0.00001
TEA021421.1     TMHMM2.0        outside      1  1331
```

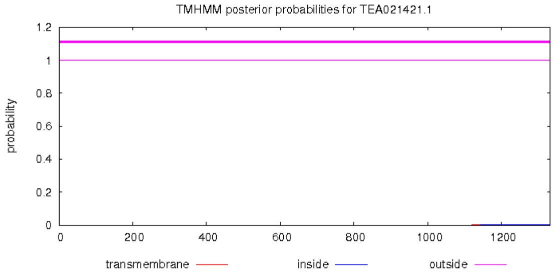


```
# TEA017670.1 Length: 561
# TEA017670.1 Number of predicted TMHs:  0
# TEA017670.1 Exp number of AAs in TMHs: 0.03517
# TEA017670.1 Exp number, first 60 AAs:  0
# TEA017670.1 Total prob of N-in:        0.00270
TEA017670.1     TMHMM2.0        outside      1   561
```

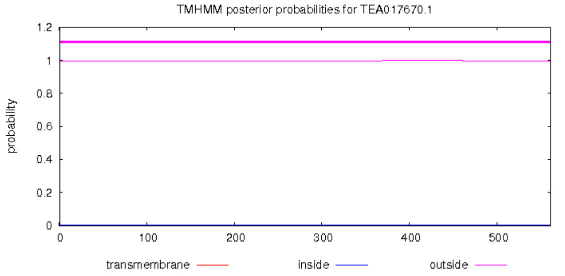


```
# TEA019184.1 Length: 601
# TEA019184.1 Number of predicted TMHs:  0
# TEA019184.1 Exp number of AAs in TMHs: 0.01866
# TEA019184.1 Exp number, first 60 AAs:  0
# TEA019184.1 Total prob of N-in:        0.00147
TEA019184.1     TMHMM2.0        outside      1   601
```

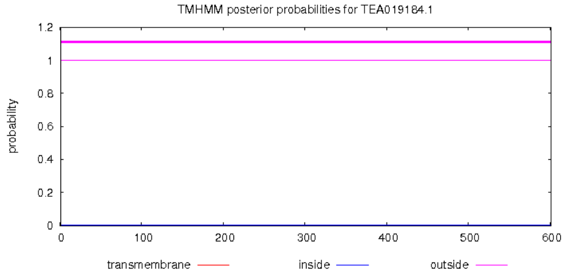

```
# TEA000933.1 Length: 407
# TEA000933.1 Number of predicted TMHs:  0
# TEA000933.1 Exp number of AAs in TMHs: 0.46154
# TEA000933.1 Exp number, first 60 AAs:  0
# TEA000933.1 Total prob of N-in:        0.00794
TEA000933.1     TMHMM2.0        outside      1   407
```

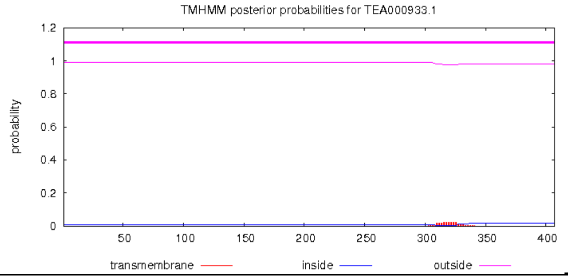


```
# TEA031230.1 Length: 489
# TEA031230.1 Number of predicted TMHs:  0
# TEA031230.1 Exp number of AAs in TMHs: 0.13166
# TEA031230.1 Exp number, first 60 AAs:  0.00013
# TEA031230.1 Total prob of N-in:        0.00470
TEA031230.1     TMHMM2.0        outside      1   489
```

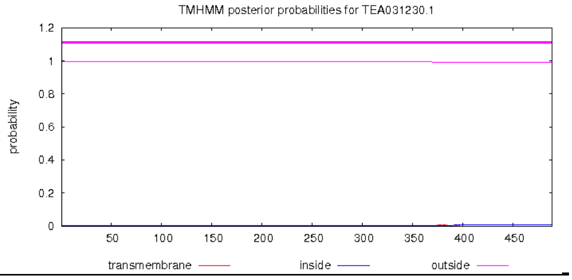


```
# TEA022171.1 Length: 404
# TEA022171.1 Number of predicted TMHs:  0
# TEA022171.1 Exp number of AAs in TMHs: 0.01418
# TEA022171.1 Exp number, first 60 AAs:  0
# TEA022171.1 Total prob of N-in:        0.01930
TEA022171.1     TMHMM2.0        outside      1   404
```

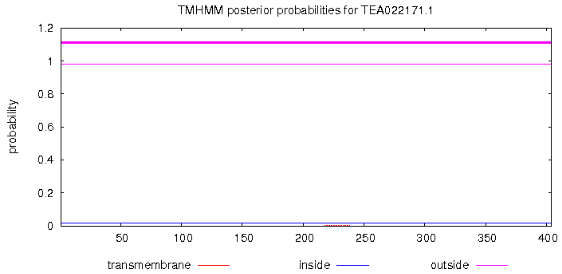


```
# TEA011280.1 Length: 368
# TEA011280.1 Number of predicted TMHs:  0
# TEA011280.1 Exp number of AAs in TMHs: 0.0076
# TEA011280.1 Exp number, first 60 AAs:  0
# TEA011280.1 Total prob of N-in:        0.00291
TEA011280.1     TMHMM2.0        outside      1   368
```

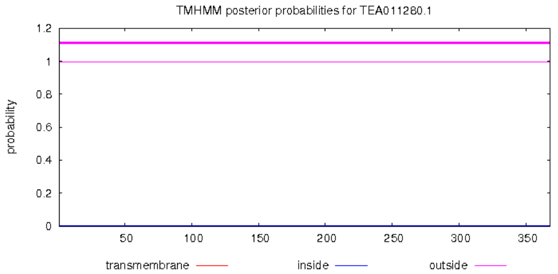


```
# TEA031223.1 Length: 434
# TEA031223.1 Number of predicted TMHs:  0
# TEA031223.1 Exp number of AAs in TMHs: 0.27035
# TEA031223.1 Exp number, first 60 AAs:  0
# TEA031223.1 Total prob of N-in:        0.01318
TEA031223.1     TMHMM2.0        outside      1   434
```

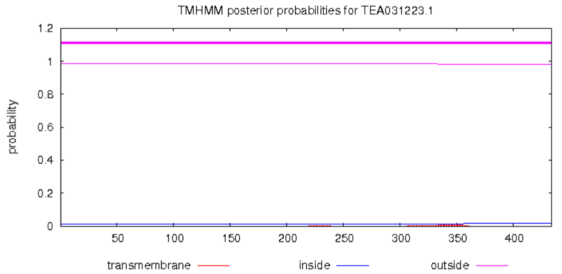


```
# TEA007232.1 Length: 368
# TEA007232.1 Number of predicted TMHs:  0
# TEA007232.1 Exp number of AAs in TMHs: 0.04776
# TEA007232.1 Exp number, first 60 AAs:  0
# TEA007232.1 Total prob of N-in:        0.01435
TEA007232.1     TMHMM2.0        outside      1   368
```

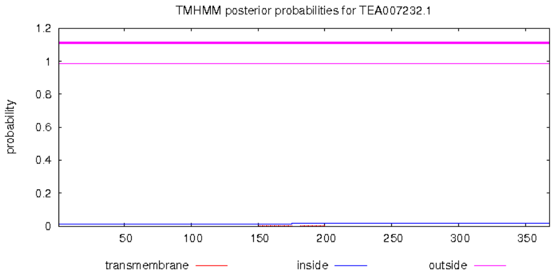


```
# TEA016553.1 Length: 432
# TEA016553.1 Number of predicted TMHs:  0
# TEA016553.1 Exp number of AAs in TMHs: 0.01056
# TEA016553.1 Exp number, first 60 AAs:  0
# TEA016553.1 Total prob of N-in:        0.00632
TEA016553.1     TMHMM2.0        outside      1   432
```

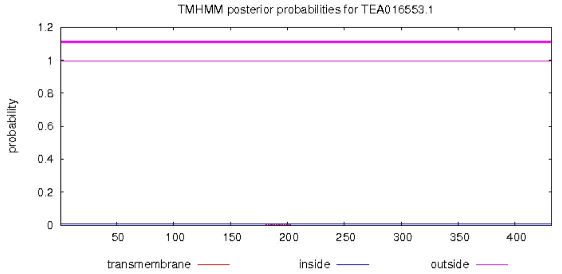


```
# TEA033032.1 Length: 415
# TEA033032.1 Number of predicted TMHs:  0
# TEA033032.1 Exp number of AAs in TMHs: 0.16877
# TEA033032.1 Exp number, first 60 AAs:  8e-05
# TEA033032.1 Total prob of N-in:        0.00767
TEA033032.1     TMHMM2.0        outside      1   415
```

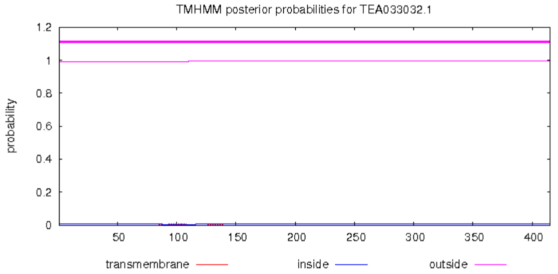

```
# TEA001764.1 Length: 351
# TEA001764.1 Number of predicted TMHs:  0
# TEA001764.1 Exp number of AAs in TMHs: 0.22163
# TEA001764.1 Exp number, first 60 AAs:  0.00043
# TEA001764.1 Total prob of N-in:        0.01088
TEA001764.1     TMHMM2.0        outside      1   351
```

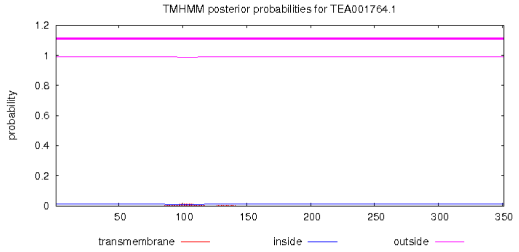


```
# TEA026000.1 Length: 1296
# TEA026000.1 Number of predicted TMHs:  0
# TEA026000.1 Exp number of AAs in TMHs: 0.3705
# TEA026000.1 Exp number, first 60 AAs:  0
# TEA026000.1 Total prob of N-in:        0.00000
TEA026000.1     TMHMM2.0        outside      1  1296
```

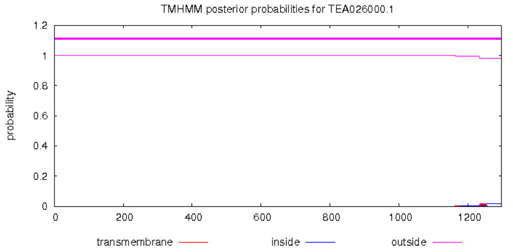


```
# TEA033556.1 Length: 541
# TEA033556.1 Number of predicted TMHs:  0
# TEA033556.1 Exp number of AAs in TMHs: 4.33922
# TEA033556.1 Exp number, first 60 AAs:  0.00021
# TEA033556.1 Total prob of N-in:        0.06460
TEA033556.1     TMHMM2.0        outside      1   541
```

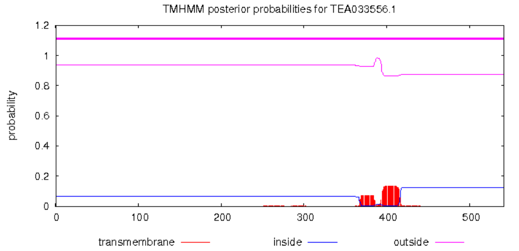


```
# TEA013875.1 Length: 341
# TEA013875.1 Number of predicted TMHs:  0
# TEA013875.1 Exp number of AAs in TMHs: 1.96914
# TEA013875.1 Exp number, first 60 AAs:  0.00196
# TEA013875.1 Total prob of N-in:        0.13897
TEA013875.1     TMHMM2.0        outside      1   341
```

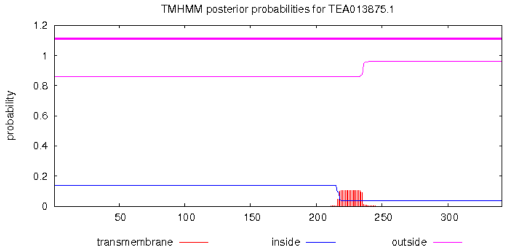


```
# TEA002722.1 Length: 1436
# TEA002722.1 Number of predicted TMHs:  0
# TEA002722.1 Exp number of AAs in TMHs: 0.01114
# TEA002722.1 Exp number, first 60 AAs:  6e-05
# TEA002722.1 Total prob of N-in:        0.00001
TEA002722.1     TMHMM2.0        outside      1  1436
```

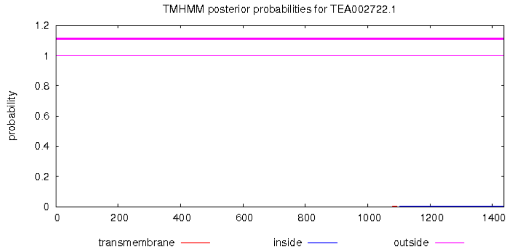


```
# TEA030052.1 Length: 1357
# TEA030052.1 Number of predicted TMHs:  0
# TEA030052.1 Exp number of AAs in TMHs: 0.02427
# TEA030052.1 Exp number, first 60 AAs:  0.00401
# TEA030052.1 Total prob of N-in:        0.00064
TEA030052.1     TMHMM2.0        outside      1  1357
```

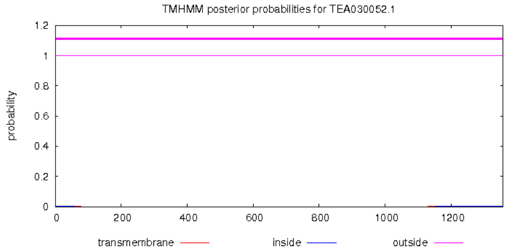


```
# TEA008343.1 Length: 334
# TEA008343.1 Number of predicted TMHs:  0
# TEA008343.1 Exp number of AAs in TMHs: 0.097979999
# TEA008343.1 Exp number, first 60 AAs:  0
# TEA008343.1 Total prob of N-in:        0.02590
TEA008343.1     TMHMM2.0        outside      1   334
```

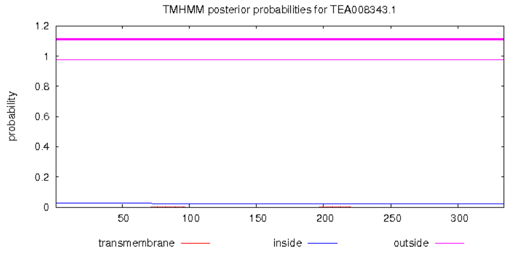

Supplementary Figure S2: Transmembrane helices of MAPKKKs of Raf subfamily in *C. sinensis*. TMHMM Server, v.2.0 (http://www.cbs.dtu.dk/services/TMHMM/), ~~was used to generate the transmembrane helices.~~

```
# TEA010125.1 Length: 675
# TEA010125.1 Number of predicted TMHs:  0
# TEA010125.1 Exp number of AAs in TMHs: 0.00052
# TEA010125.1 Exp number, first 60 AAs:  0
# TEA010125.1 Total prob of N-in:        0.00014
TEA010125.1     TMHMM2.0        outside      1   675
```

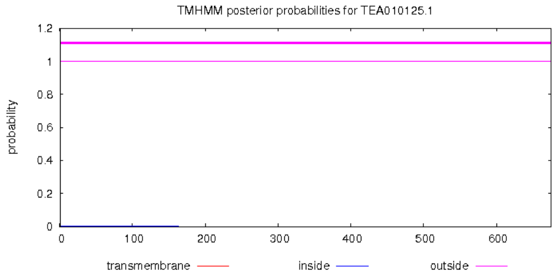


```
# TEA022762.1 Length: 732
# TEA022762.1 Number of predicted TMHs:  0
# TEA022762.1 Exp number of AAs in TMHs: 0.01389
# TEA022762.1 Exp number, first 60 AAs:  0.01054
# TEA022762.1 Total prob of N-in:        0.00094
TEA022762.1     TMHMM2.0        outside      1   732
```

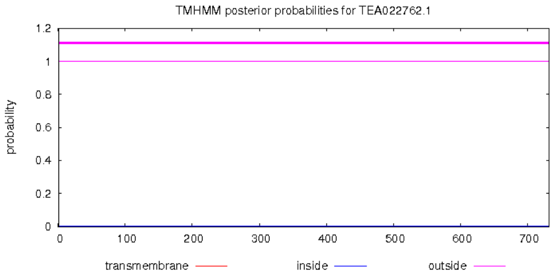


```
# TEA024720.1 Length: 655
# TEA024720.1 Number of predicted TMHs:  0
# TEA024720.1 Exp number of AAs in TMHs: 0.20568
# TEA024720.1 Exp number, first 60 AAs:  0.00353
# TEA024720.1 Total prob of N-in:        0.01086
TEA024720.1     TMHMM2.0        outside      1   655
```

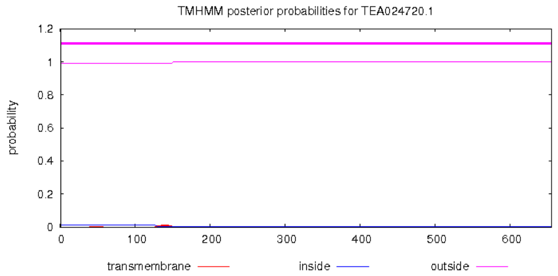


```
# TEA002087.1 Length: 719
# TEA002087.1 Number of predicted TMHs:  0
# TEA002087.1 Exp number of AAs in TMHs: 0.00067
# TEA002087.1 Exp number, first 60 AAs:  0
# TEA002087.1 Total prob of N-in:        0.00011
TEA002087.1     TMHMM2.0        outside      1   719
```

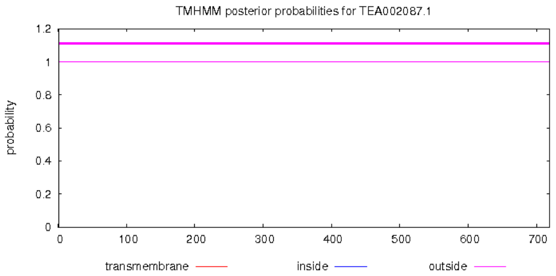


```
# TEA013346.1 Length: 831
# TEA013346.1 Number of predicted TMHs:  0
# TEA013346.1 Exp number of AAs in TMHs: 0.00018
# TEA013346.1 Exp number, first 60 AAs:  0
# TEA013346.1 Total prob of N-in:        0.00002
TEA013346.1     TMHMM2.0        outside      1   831
```

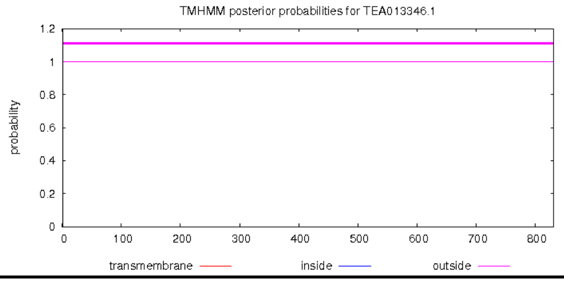


```
# TEA013344.1 Length: 481
# TEA013344.1 Number of predicted TMHs:  0
# TEA013344.1 Exp number of AAs in TMHs: 0.00164
# TEA013344.1 Exp number, first 60 AAs:  0
# TEA013344.1 Total prob of N-in:        0.00069
TEA013344.1     TMHMM2.0        outside      1   481
```

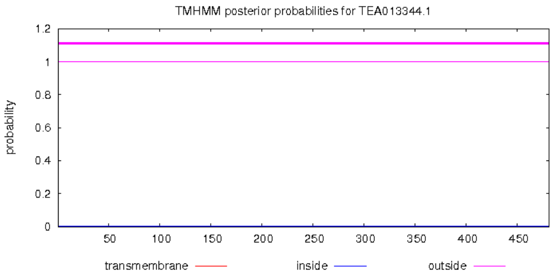


```
# TEA031068.1 Length: 762
# TEA031068.1 Number of predicted TMHs:  0
# TEA031068.1 Exp number of AAs in TMHs: 3.06785
# TEA031068.1 Exp number, first 60 AAs:  1.30494
# TEA031068.1 Total prob of N-in:        0.06089
TEA031068.1     TMHMM2.0        outside      1   762
```

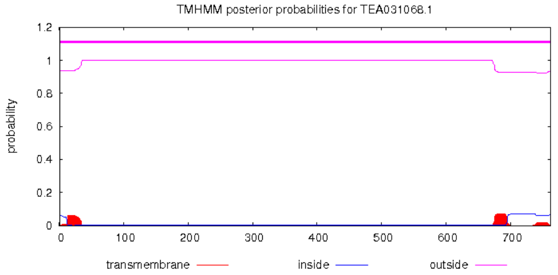


```
# TEA020698.1 Length: 664
# TEA020698.1 Number of predicted TMHs:  0
# TEA020698.1 Exp number of AAs in TMHs: 1.53983
# TEA020698.1 Exp number, first 60 AAs:  0.00118
# TEA020698.1 Total prob of N-in:        0.00060
TEA020698.1     TMHMM2.0        outside      1   664
```

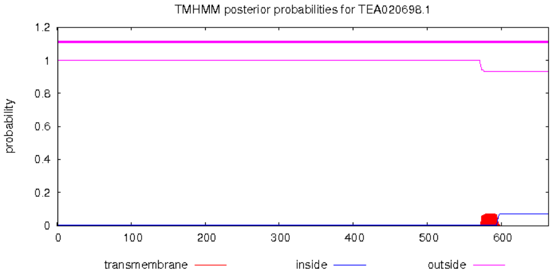

```
# TEA027328.1 Length: 748
# TEA027328.1 Number of predicted TMHs:  1
# TEA027328.1 Exp number of AAs in TMHs: 30.33445
# TEA027328.1 Exp number, first 60 AAs:  29.76268
# TEA027328.1 Total prob of N-in:        0.47196
# TEA027328.1 POSSIBLE N-term signal sequence
TEA027328.1     TMHMM2.0        inside       1     39
TEA027328.1     TMHMM2.0        TMhelix     40     58
TEA027328.1     TMHMM2.0        outside     59    748
```

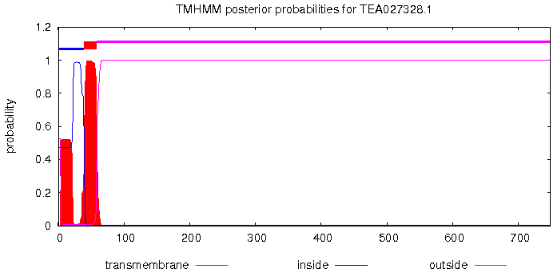


```
# TEA020112.1 Length: 300
# TEA020112.1 Number of predicted TMHs:  0
# TEA020112.1 Exp number of AAs in TMHs: 0.00508
# TEA020112.1 Exp number, first 60 AAs:  0
# TEA020112.1 Total prob of N-in:        0.01462
TEA020112.1     TMHMM2.0        outside      1    300
```

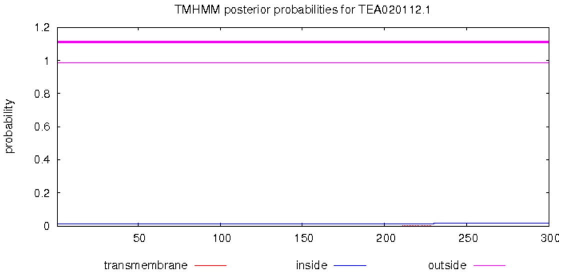


```
# TEA033250.1 Length: 622
# TEA033250.1 Number of predicted TMHs:  0
# TEA033250.1 Exp number of AAs in TMHs: 0.00119
# TEA033250.1 Exp number, first 60 AAs:  0
# TEA033250.1 Total prob of N-in:        0.00024
TEA033250.1     TMHMM2.0        outside      1    622
```

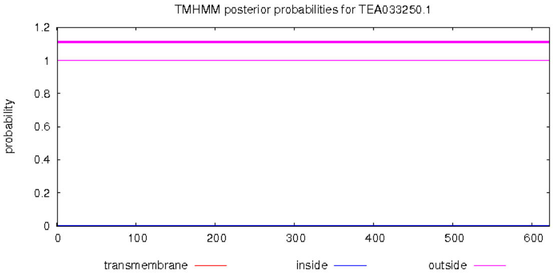

Supplementary Figure S3: Transmembrane helices of MAPKKKs of ZIK subfamily in *C. sinensis*. TMHMM Server, v.2.0 (http://www.cbs.dtu.dk/services/TMHMM/), ~~was used to generate the transmembrane helices.~~

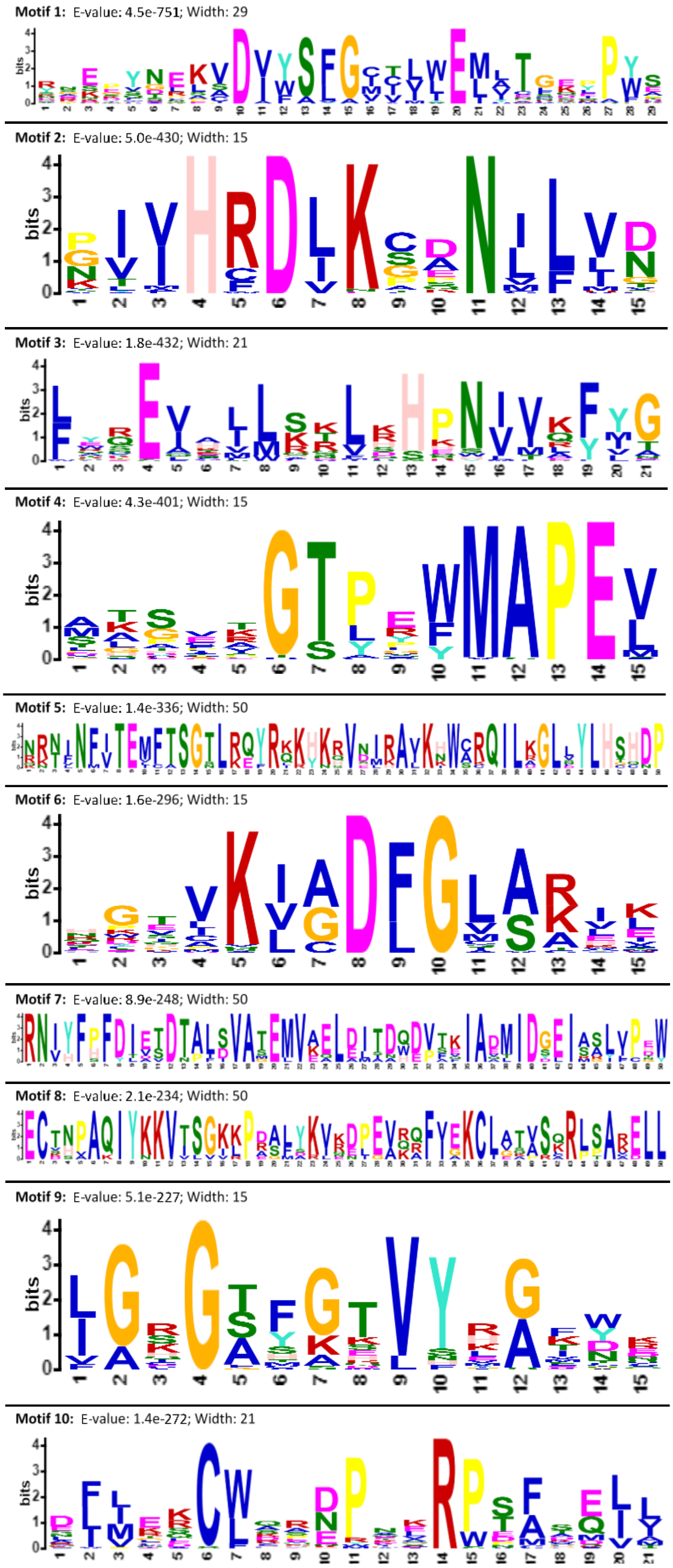

Supplementary Figure S4: Motif logos of the 10 identified motifs in 59 MAPKKKs ~~in~~ *C. sinensis*. The motif logos were generated by MEME suite.

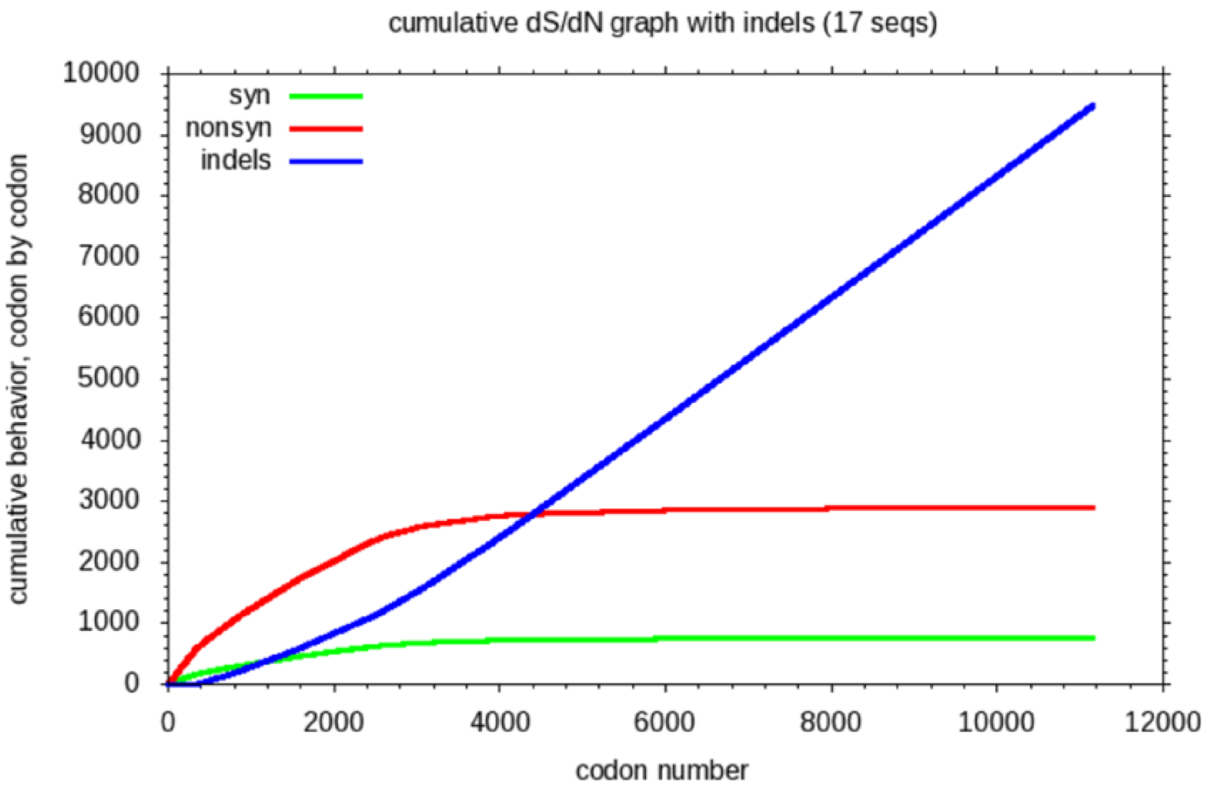

Supplementary Figure S5: The ds/dn cumulative graph of MAPKKKs of MEKK subfamily in *C. sinensis*. SNAP server (https://www.hiv.lanl.gov/content/sequence/SNAP/SNAP.html) has been used to generate the graph.

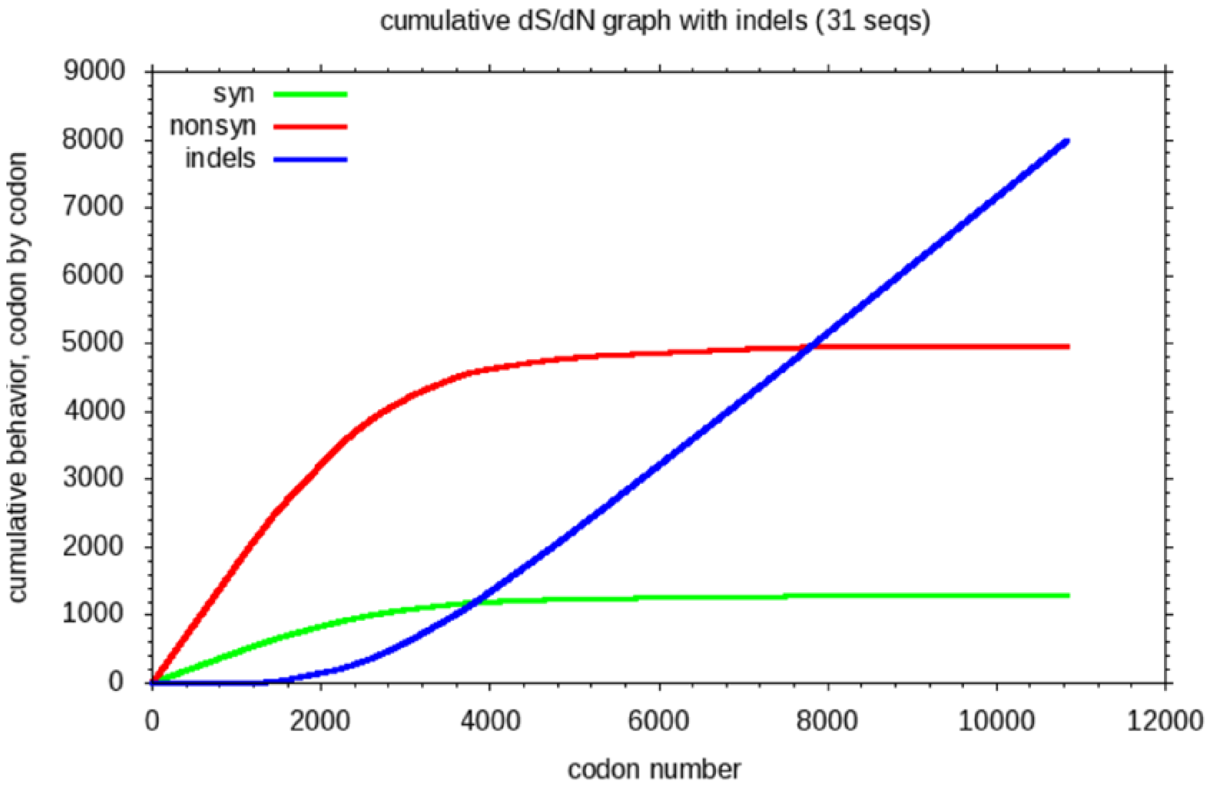

Supplementary Figure S6: The ds/dn cumulative graph of MAPKKKs of Raf subfamily in *C. sinensis*. SNAP server (https://www.hiv.lanl.gov/content/sequence/SNAP/SNAP.html) has been used to generate the graph.

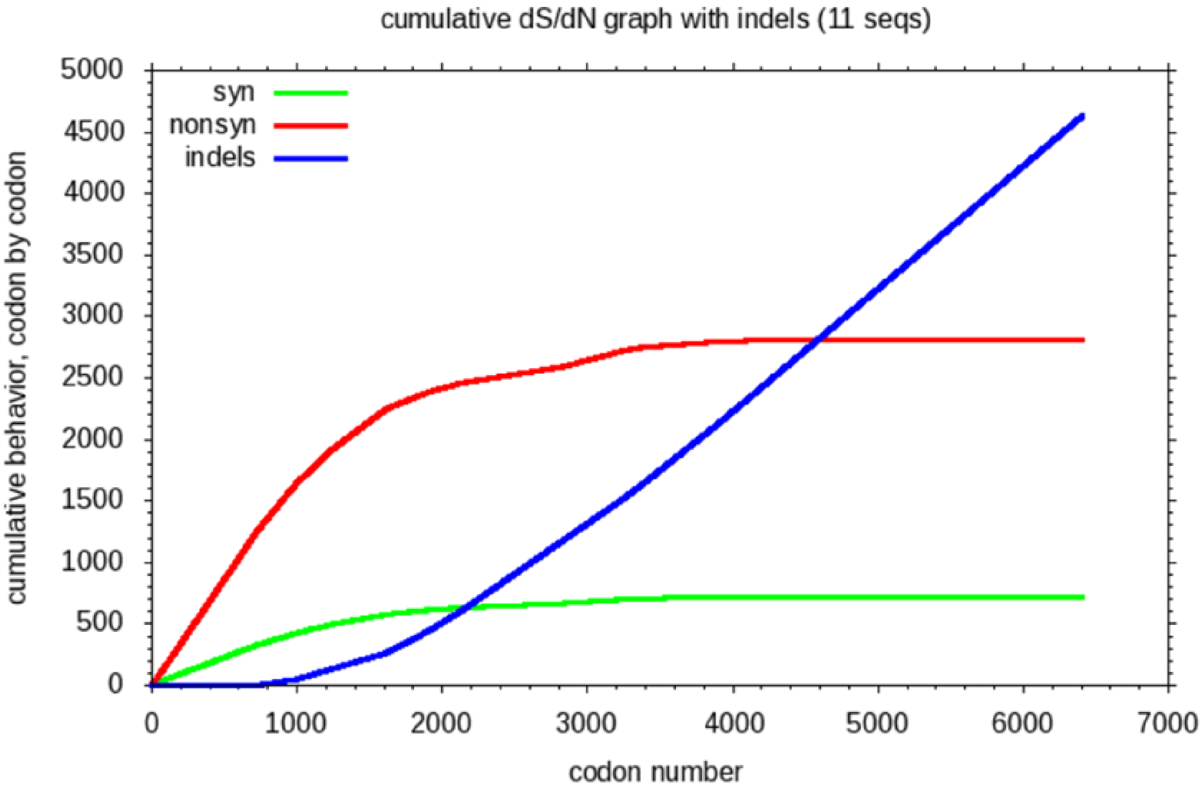

Supplementary Figure S7: The ds/dn cumulative graph of MAPKKKs of ZIK subfamily in *C. sinensis*. SNAP server (https://www.hiv.lanl.gov/content/sequence/SNAP/SNAP.html) has been used to generate the graph.

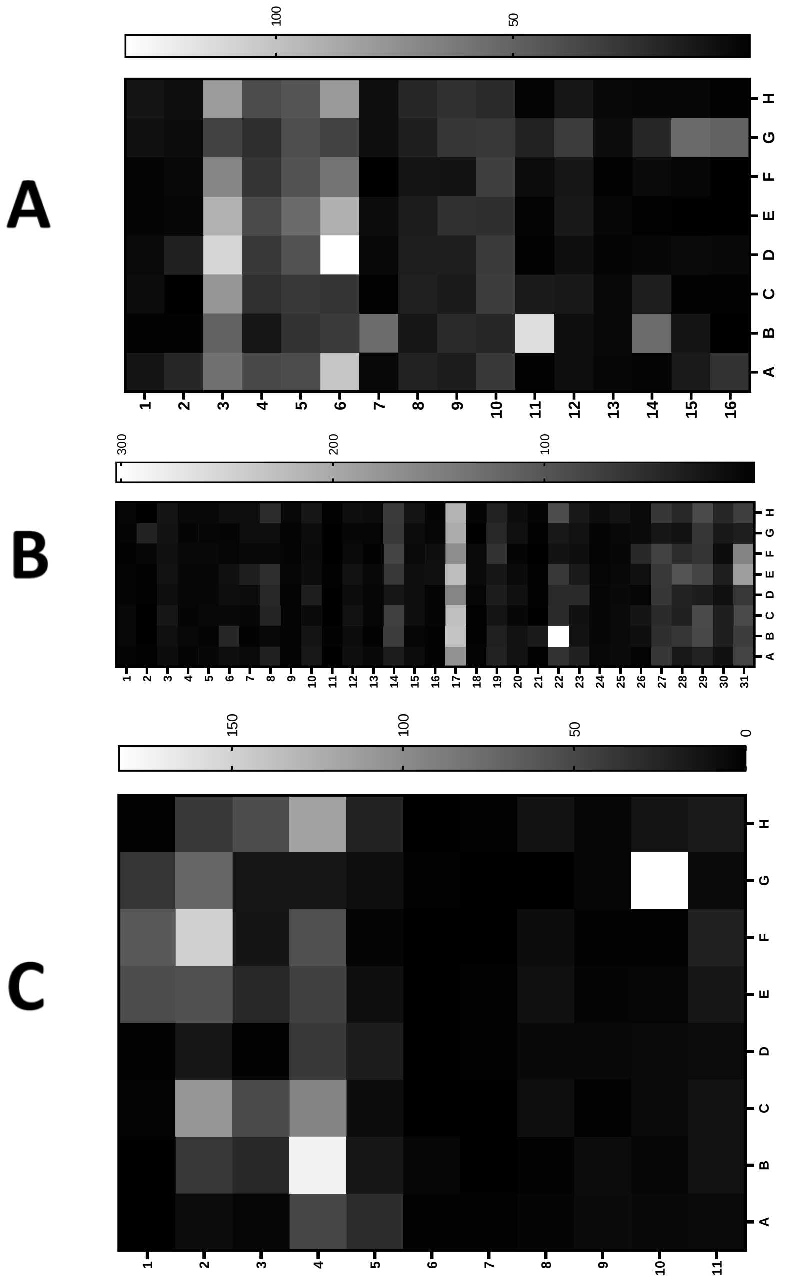

Supplementary Figure S8: Heat maps for tissue-specific expression patterns of A) MEKK; B) Raf and; C) ZIK genes in *C. sinensis*. The relative expression of these genes was analysed in different ~~developmental stages~~, by using GraphPad Prism 8 software. The level of expression was in transcript per million (TPM). The numbers below represent the tea MAPKKK genes and the alphabets on the right represent the 8 different tissues.

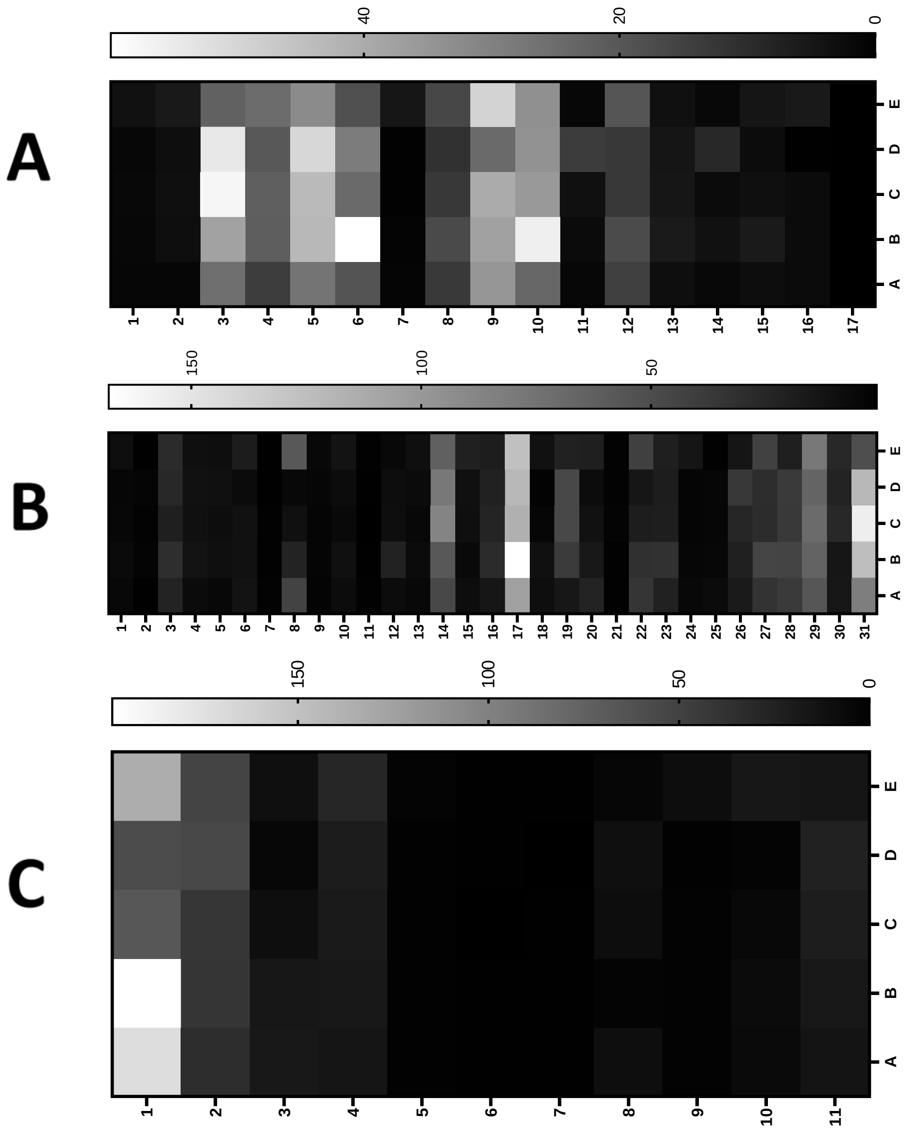

Supplementary Figure S9: Heat maps for cold stress expression patterns of A) MEKK; B) Raf and; C) ZIK genes in ***C. sinensis*****.** The relative expression of these genes was analysed in different ~~developmental~~ stages, by using GraphPad Prism 8 software. The level of expression was in transcript per million (TPM). The numbers below represent the tea MAPKKK genes and the alphabets on the right represent the parameters of study.

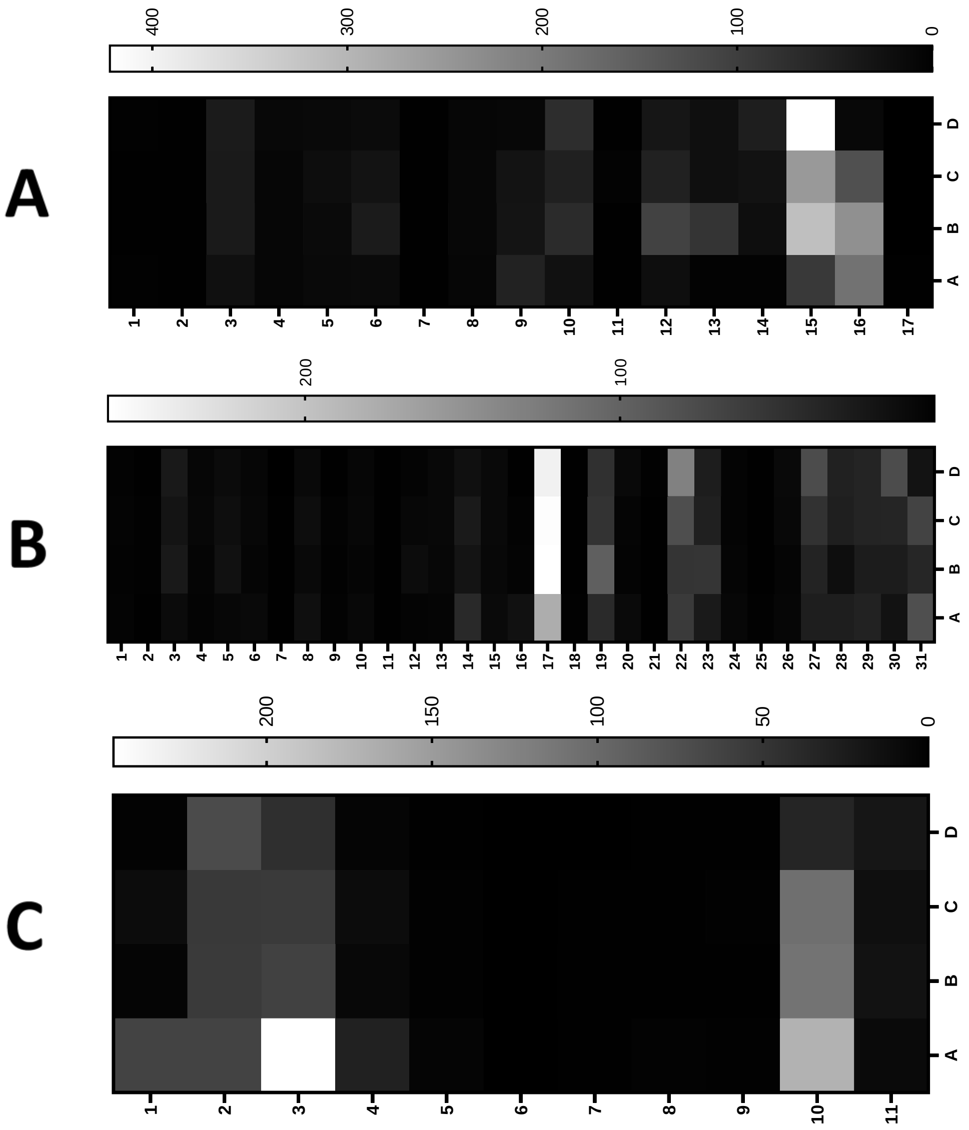

Supplementary Figure S10: Heat maps for drought stress expression patterns of A) MEKK; B) Raf and; C) ZIK genes in *C. sinensis*. The relative expression of these genes was analysed in different ~~developmental~~ stages, by using GraphPad Prism 8 software. The level of expression was in transcript per million (TPM). The numbers below represent the tea MAPKKK genes and the alphabets on the right represent the parameters of study.

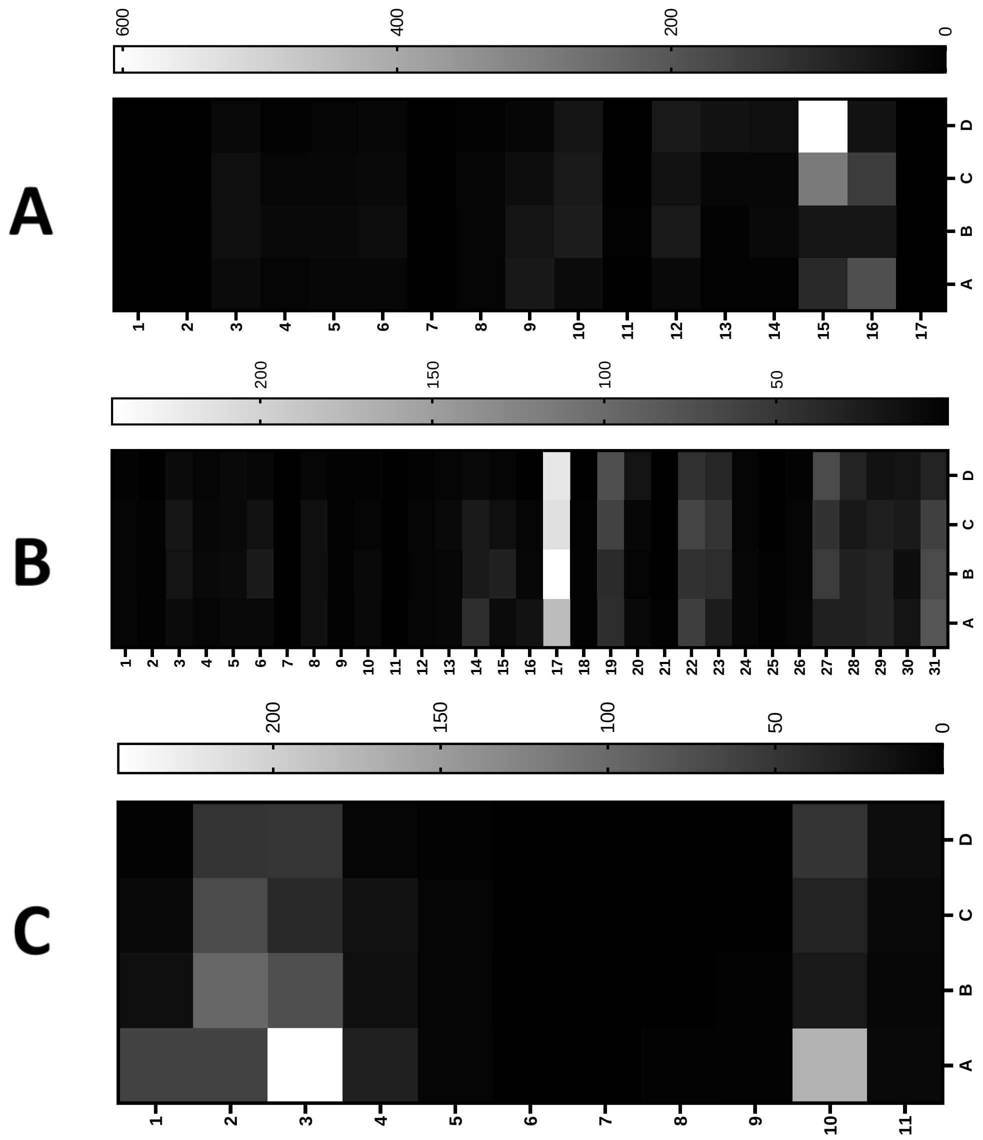

Supplementary Figure S11: Heat maps for salt stress expression patterns of (A) MEKK (B) Raf and (C) ZIK genes in *C. sinensis*. The relative expression of these genes was analysed in different ~~developmental~~ stages, by using GraphPad Prism 8 software. The level of expression was in transcript per million (TPM). The numbers below represent the tea MAPKKK genes and the alphabets on the right represent the parameters of study.

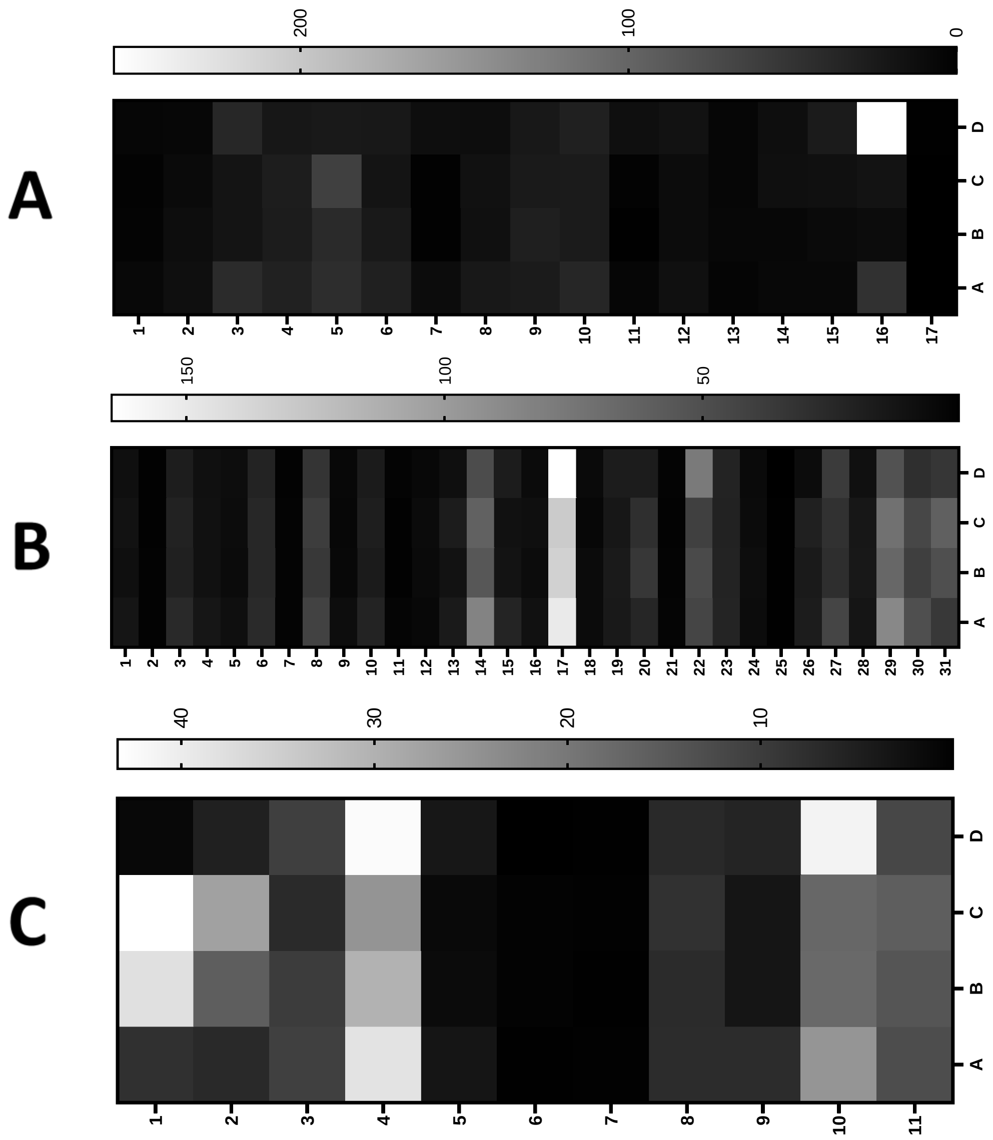

Supplementary Figure S12: Heat maps for MeJA treatment expression patterns of (A) MEKK (B) Raf and (C) ZIK genes in *C. sinensis*. The relative expression of these genes was analysed in different ~~developmental~~ stages, by using GraphPad Prism 8 software. The level of expression was in transcript per million (TPM). The numbers below represent the tea MAPKKK genes and the alphabets on the right represent the parameters of study.
