# Supplementary material for: In-silico genome wide analysis of Mitogen activated protein kinase kinase kinase gene family in C. sinensis
Source: PLoS One. 2021 Nov 4;16(11):e0258657. doi: 10.1371/journal.pone.0258657 (PMC8568164; doi:10.1371/journal.pone.0258657)
Supplement: Attachment — Submitted filename: Comments to Authors.pdf [file pone.0258657.s010.pdf]

The authors have substantially improved the revised manuscript and incorporated all the necessary changes required. I am satisfied with the authors response on the queries asked. However, before it is accepted for publication, I would like to know a bit about the process of orthologous gene identification authors have employed for my clarification.

- Did the authors use all the *Arabidopsis* MEKK, Raf and ZIK genes accession one by one and looked for the orthologs in *C.sinenis* TPIA database Orthologous Groups search option?
- Does TPIA database Orthologous Groups search option includes all the *C.sinensis* gene accessions that are orthologous to other species?

For future similar kind of work, I would advise authors to incorporate synteny analysis between the organism of interest and a model or reference organism. It is not required for this paper now. Authors just needs to address the concern I have raised above regarding orthologous gene identification. Further, authors need to make few minor changes in the manuscript before acceptance.

(1) I have strikeout and underlined few words in some sentences and also provided comments on those within the revised manuscript pdf file (revised manuscript with track changes part of the pdf file). Please find the attached file. Authors are advised to incorporate the required changes.

(2) The authors need to correct the legends of Fig 9, Fig 10, Fig 11, Fig 12.

The relative expression of the genes were analysed in the leaves of *C.sinensis* in different stages and not in different tissues for the Fig 9, Fig 10, Fig 11, Fig 12. Authors wrote "The relative expression of these genes were analysed in different tissues by using GraphPad Prism 8 software".

Authors need to incorporated the corrected legend for Fig 9, Fig 10, Fig 11, Fig 12. The corrected legend for Fig 9, Fig 10, Fig 11, Fig 12 are given below:

Fig 9. Gene expression patterns of (A) MEKK (B) Raf and (C) ZIK genes, under cold stress conditions in *C. sinensis*. The relative expression of these genes were analysed in different stages by using GraphPad Prism 8 software. The level of expression was in transcript per million (TPM).

Fig 10. Gene expression patterns of (A) MEKK (B) Raf and (C) ZIK genes, under drought stress conditions in *C. sinensis*. The relative expression of these genes were analysed in different stages by using GraphPad Prism 8 software. The level of expression was in transcript per million (TPM).

Fig 11. Gene expression patterns of (A) MEKK (B) Raf and (C) ZIK genes, under salt stress conditions in *C. sinensis*. The relative expression of these genes were analysed in different stages by using GraphPad Prism 8 software. The level of expression was in transcript per million (TPM).

Fig 12. Gene expression patterns of (A) MEKK (B) Raf and (C) ZIK genes, under Methyl jasmonate (MeJA) treatment in *C. sinensis*. The relative expression of these genes were analysed in different stages by using GraphPad Prism 8 software. The level of expression was in transcript per million (TPM).
