# Supplementary material for: In-silico genome wide analysis of Mitogen activated protein kinase kinase kinase gene family in C. sinensis
Source: PLoS One. 2021 Nov 4;16(11):e0258657. doi: 10.1371/journal.pone.0258657 (PMC8568164; doi:10.1371/journal.pone.0258657)
Supplement: Attachment — Submitted filename: PONE-D-21-05595_R1_reviewer.pdf [file pone.0258657.s011.pdf]

PLOS ONE

In-silico identification, expressional profile and regulatory network analysis of Mitogen Activated Protein Kinase Kinase Kinase gene family in C. sinensis

--Manuscript Draft--

**Manuscript Number:**	PONE-D-21-05595R1
**Article Type:**	Research Article
**Full Title:**	In-silico identification, expressional profile and regulatory network analysis of Mitogen Activated Protein Kinase Kinase Kinase gene family in C. sinensis
**Short Title:**	MAPKKK gene family in C. sinensis
**Corresponding Author:**	Neelam Mishra<br>Saint Joseph's College: Saint Joseph's University<br>bengaluru, Karnataka INDIA
**Keywords:**	Keywords: Mitogen Activated Protein Kinase; Arabidopsis thaliana; phylogenetic relationship; Functional interaction; abiotic stress
**Abstract:**	Mitogen activated protein kinase kinase kinase (MAPKKK) form the upstream component of MAPK cascade. It is well characterized in several plants such as Arabidopsis and rice however the knowledge about MAPKKKs in tea plant is largely unknown. In the present study, MAPKKK genes of tea were obtained through a genome wide search using Arabidopsis thaliana as the reference genome. Among 59 candidate MAPKKK genes in tea, 17 genes were MEKK-like, 31 genes were Raf-like and 11 genes were ZIK- like. Additionally, phylogenetic relationships were established along with structural analysis, which includes gene structure, its location as well as conserved motifs, cis-acting regulatory elements and functional domain signatures that were systematically examined, and further, predictions were validated by the results. Also, on the basis of orthologous genes in Arabidopsis, functional interaction was carried out in C. sinensis. The expressional profiles indicated major involvement of MAPKKK genes from tea in response to various abiotic stress factors. Taken together, this study provides the targets for additional inclusive identification, functional study, and provides comprehensive knowledge for a better understanding of the MAPKKK cascade regulatory network in C. sinensis.
**Order of Authors:**	Neelam Mishra
	Guoxin Shen
	Anurag srivastava
	shreya Subrahmanya
	Abhirup Paul
**Response to Reviewers:**	Dear Editor,<br><br>Two references as mentioned by you are now added in the revised manuscript.<br><br>Reviewer 1<br>Minor Comments<br>1.Few grammatical and typographical errors need to be corrected.<br>>Response: The grammatical and typographical errors have been corrected and are highlighted as yellow in the revised manuscript.<br><br>2.All the abbreviated forms are required to be written in their full form at the first instance of their occurrence.<br>>Response: All the abbreviated forms are written in their full form at the first instance of their occurrence in the revised manuscript.<br><br>3.Reframing of few sentences is required.<br>>Response: The sentences have been reframed in the revised manuscript under the yellow highlights as suggested by the reviewer.

Powered by Editorial Manager® and ProduXion Manager® from Aries Systems Corporation

4.Few questions related to homologous/orthologous gene pairs and few others questions needed to be answered.
>Response: We agree with the reviewer that few questions related to homologous/orthologous gene pairs and few other questions needs to be addressed in the manuscript. We have provided a detailed response of this question (Manuscript comments A and B) and have incorporated the corresponding changes in the revised manuscript (page 12).
Manuscript comments
A.What does authors mean here by saying Raf tree did not feature any orthologous gene pair? Does authors mean to say that Raf phylogenetic tree did not contain any tea orthologous gene from arabidopsis or any other species? If they are not homologous or orthologous genes how they are clustered in the same clade of the Raf phylogenetic tree? Please clarify a bit.
B.What does authors mean here by saying ZIK tree did not feature any orthologous gene pair? Does authors mean to say that ZIK phylogenetic tree did not contain any tea orthologous gene from arabidopsis or any other species? If they are not homologous or orthologous genes how they are clustered in the same clade of the ZIK phylogenetic tree? Please clarify a bit.

>Response: We thank the reviewer for the comment. The phylogenetic analysis of the Raf and ZIK genes of tea showed that the respective genes have clustered together in the same clade, along with the other Raf and ZIK genes identified from different plant species. This is suggestive of the fact that the genes are either homologous or orthologous to each other. However, the Raf and ZIK genes did not feature any orthologous gene with respect to Arabidopsis. This was validated only when the Raf and ZIK gene accession IDs were scanned in the TPIA database to search for the presence of orthologous genes in the later part of the study (Functional Interaction Network).
The reason for clustering of the Raf and ZIK genes under the same clade in their respective phylogenetic tree despite not being orthologous is that; orthologs are defined as genes that share a common ancestor by speciation which led to the genes clustering together. So, the identification of orthologous genes via in-silico approach is purely based on the assumption that these orthologous genes start to diverge after a speciation event and may diverge within the same clade or an adjacent clade, sharing the common ancestor (Wu et al., 2006). It is thus possible for such orthologous groups to exist in a phylogenetic tree. But, when these Raf and ZIK genes were further searched in the TPIA database, it showed the absence of any orthologous genes.
The same thing has been clarified and the corresponding changes have been made in the revised manuscript under the heading “Phylogenetic analysis of tea MAPKKKs” in the “Results” section with a line beginning with “The results are suggestive of the fact……….” (page 12).
Reference: Wu, Feinan et al. “Combining bioinformatics and phylogenetics to identify large sets of single-copy orthologous genes (COSII) for comparative, evolutionary and systematic studies: a test case in the euasterid plant clade.” Genetics vol. 174,3 (2006): 1407-20. doi:10.1534/genetics.106.062455

C.Authors have mentioned the function of tea orthologues in Arabidopsis that formed a part of the interaction network. However, they did not cite any reference on the basis of which they have included these statements. If they have taken from STRING server or database, they need to mention the same or if they have taken from literature then cite the reference.

>Response: The function of tea orthologues in Arabidopsis that formed a part of the interaction network was taken from TAIR database and the reference for the same has been provided in the revised manuscript under the heading “GO ontology analysis and functional interaction network of tea MAPKKKs” in the “Results” section (page 19).

D.Authors have mentioned that they have extracted expression data pertaining to MAPKKKs genes from TPIA database under different abiotic stress condition. They need to mention here about the specific tissue or organ of tea plant to which these expression data correspond. Authors are advised to look for this information in the TPIA database or any of the published research papers on this and incorporate the information here.

Powered by Editorial Manager® and ProduXion Manager® from Aries Systems Corporation

>Response: We thank the reviewer for the comment. The expression data of the leaves of the tea plant present in TPIA database was used to study the effect of different abiotic stress condition and the same has also been mentioned in the revised manuscript in the “Results” section under the heading “Abiotic stress induced differential expression levels of tea MAPKKKs” with a line beginning with “The expression data of the leaves of the tea plant……….” (page 21).

5.For some statements references need to be cited.
>Response: The references for the statements as mentioned by the reviewer have been cited in the revised manuscript.

6.Some typographical error regarding the total number of plant tissue samples whose expression have been analysed need to be corrected.
>Response: We thank reviewer for pointing this. We have corrected the results in the revised manuscript. There are eight genes in total and results have been provided appropriately (page 20-21).

7.Some figures caption/legend description needs to be corrected.
>Response: The figure legends as pointed out by the reviewer have been corrected in the revised manuscripts as well as in the supplemental files.

8.Specific tissue names whose expression was analysed under different abiotic stress treatment & MeJA treatment needs to be mentioned.
>Response: The expression analysis under different abiotic stress treatment and MeJA treatment was done for the leaves of the tea plant and the same has been mentioned in the revised manuscript.

9.Throughout the discussion in many instances some sentences almost seemed to be repetitive or similar to what was already mentioned in introduction and result section. Authors are advised to cross check this and modify or reframe the sentences wherever needed.
>Response: The repetitive statements in the discussion section have been modified in the revised manuscript.

10.Scientific names need to be italicized in the Supplementary Tables captions. For Example- C. sinensis.
>Response: The scientific names in the Supplementary Tables captions have been italicized in the revised manuscript.

11.In the supplementary figures: the heatmap labels, the numbers at the bottom and alphabets on the right side, each needs to be described specifically what they correspond to.
>Response: The numbers at the bottom corresponds to the tea genes while the alphabets on the right side represent 8 different tissues. The name of the tea genes and the name of the 8 different tissues have been provided in “S9 Fig.” of Supplemental information 2.

Similarly, for abiotic stress the numbers at the bottom corresponds to the tea genes while the alphabets on the right side represent different experimental stages. The name of the tea genes and different stages has been provided in “S10-S13 Figs.” of Supplemental information 2.

Major revision
1.Additional Work: The authors need to retrieve the genomic DNA sequences upstream of the transcriptional start site for each tea MAPKKK gene from TPIA database for identifying putative promoter cis-acting elements. For identifying cis elements, the authors may use PlantCARE database (http://bioinformatics.psb.ugent.be/webtools/plantcare/html/).
>Response: We thank the reviewer for this comment. The genomic DNA sequences upstream of the transcriptional start site for each tea MAPKKK gene has been retrieved from TPIA database in order to identify putative promoter cis-acting elements in the methodology and the results of these experiment has been provided in the revised manuscripts under the heading “Analysis of cis-regulatory elements” and “Retrieval of promoter sequences and analysis of cis-regulatory elements” (page 6 and page 15) respectively.

Powered by Editorial Manager® and ProduXion Manager® from Aries Systems Corporation

Reviewer 2
Major Comments
1.Few MAPKKK genes identified In-silico genome wide analysis from C. sinensis genome in this study should be validated by wet lab.
>Response: The main aim of this study was to identify the MAPKKK genes in tea using an in-silico approach. Expression analysis data presented in this study have already been experimentally verified by other researchers and is available in the TPIA database. In addition, all the identified genes were subjected to GO ontology analysis in order to predict the potential functions of all the 59 tea MAPKKKs. The corresponding text for the same has been added in the revised manuscript under the heading “GO ontology analysis and functional interaction network of tea MAPKKKs” with a line beginning with “The GO ontology analysis was performed in-order to predict the potential functions” (page 18).

2.The identified MAPKKK genes in different subfamilies should not just be based on their phylogenetic relationships.
>Response: The classification of the identified MAPKKK genes in 3 subfamilies were conducted based on their specific domain signatures (“Domain analysis of tea MAPKKKs”, page 12). The genes were further investigated under phylogenetic analysis, conserved protein motifs, intron-exon architecture and analysis of cis regulatory elements. After performing all of these analyses, the MAPKKK genes were classified into the three different subfamilies namely MEKK, ZIK and Raf sub-family. The corresponding text for the same has been added in the revised manuscript under the “Conclusion” section with a line beginning with “The classification of the identified MAPKKK genes……..” (page 30).

3.The network of functionally interacting genes should also include the genes that have experimentally validated support.
>Response: The genes included in the functional interaction network present in this study have been taken from the TAIR database, which are already experimentally verified. Further, the functions of the tea genes present in the network were validated by the TPIA database and GO ontology. The references for the same has been added and the text has been modified accordingly in the revised manuscript under the heading “GO ontology analysis and functional interaction network of tea MAPKKKs” (page 18-20).

4.Expression profile analysis by qRT-PCR is required to reveal the involvement of the tea MAPKKK genes in various tissues during development and under various abiotic stress stimuli and plant hormonal treatment.
>Response: TPIA database has the expression profile data of all the C. sinensis genes, which have been already experimentally verified. The expression profile data of the identified MAPKKK genes presented in this study have been retrieved from the TPIA database.
References:
a)For cold stress:
Wang X, Zhao Q, Ma C, et al. Global transcriptome profiles of Camellia sinensis during cold acclimation. BMC Genomics. 2013;14:415.
b)For salt and drought stress:
Zhang Q, Cai M, Yu X, et al. Transcriptome dynamics of Camellia sinensis in response to continuous salinity and drought stress. Tree Genet Genomes. 2017;13:78
c)For MeJA treatment:
Shi, J., Ma, C., Qi, D., Lv, H., Yang, T., Peng, Q., Chen, Z. et al. (2015) Transcriptional responses and flavor volatiles biosynthesis in methyl jasmonate-treated tea leaves. BMC Plant Biol. 15, 233
d)For tissue specific:
Wei, C.L., Yang, H., Wang, S.B., Zhao, J., Liu, C., Gao, L.P., Xia, E.H. et al. (2018) Draft genome sequence of Camellia sinensis var. sinensis provides insights into the evolution of the tea genome and tea quality. Proc. Natl Acad. Sci. 115, E4151–E4158.

5.The study should decipher a model of signalling mechanism mediated by MAPKKK genes cascade in tea.

Powered by Editorial Manager® and ProduXion Manager® from Aries Systems Corporation

	>Response: A putative signalling mechanism mediated by MAPKKK genes cascade in tea has been included in the “Discussion” section of the revised manuscript.
**Additional Information:**	
**Question**	**Response**
**Financial Disclosure**<br><br>Enter a financial disclosure statement that describes the sources of funding for the work included in this submission. Review the submission guidelines for detailed requirements. View published research articles from *PLOS ONE* for specific examples.<br><br>This statement is required for submission and **will appear in the published article** if the submission is accepted. Please make sure it is accurate.<br><br>**Unfunded studies**<br>Enter: *The author(s) received no specific funding for this work.*<br><br>**Funded studies**<br>Enter a statement with the following details:<br>• Initials of the authors who received each award<br>• Grant numbers awarded to each author<br>• The full name of each funder<br>• URL of each funder website<br>• Did the sponsors or funders play any role in the study design, data collection and analysis, decision to publish, or preparation of the manuscript?<br>• **NO** - Include this sentence at the end of your statement: *The funders had no role in study design, data collection and analysis, decision to publish, or preparation of the manuscript.*<br>• **YES** - Specify the role(s) played.<br><br>* typeset	This project was supported by the Key Technologies R & D Program for Crop Breeding of Zhejiang Province (2016C02054-19,2017C02010), the Natural Science Foundation of China (31670303), and the Joint Laboratory of Olive Oil Quality and Nutrition among China, Australia and Spain. The authors are thankful to DBT-eLibrary Consortium (DeLCON) for providing access to e-resources.
**Competing Interests**<br><br>Use the instructions below to enter a competing interest statement for this submission. On behalf of all authors, disclose any competing interests that could be perceived to bias this work—acknowledging all financial support and any other relevant financial or non-	The authors have declared that no competing interests exist.

financial competing interests.<br><br>This statement is required for submission and **will appear in the published article** if the submission is accepted. Please make sure it is accurate and that any funding sources listed in your Funding Information later in the submission form are also declared in your Financial Disclosure statement.<br><br>View published research articles from *PLOS ONE* for specific examples.<br><br>**NO authors have competing interests**<br><br>Enter: *The authors have declared that no competing interests exist.*<br><br>**Authors with competing interests**<br><br>Enter competing interest details beginning with this statement:<br><br>*I have read the journal's policy and the authors of this manuscript have the following competing interests: [insert competing interests here]*<br><br>* typeset	
**Ethics Statement**<br><br>Enter an ethics statement for this submission. This statement is required if the study involved:<br><br>• Human participants<br>• Human specimens or tissue<br>• Vertebrate animals or cephalopods<br>• Vertebrate embryos or tissues<br>• Field research<br><br>Write "N/A" if the submission does not require an ethics statement.<br><br>General guidance is provided below. Consult the submission guidelines for	N/A

detailed instructions. **Make sure that all information entered here is included in the Methods section of the manuscript.**<br><br>**Format for specific study types**<br><br>**Human Subject Research (involving human participants and/or tissue)**<br>• Give the name of the institutional review board or ethics committee that approved the study<br>• Include the approval number and/or a statement indicating approval of this research<br>• Indicate the form of consent obtained (written/oral) or the reason that consent was not obtained (e.g. the data were analyzed anonymously)<br><br>**Animal Research (involving vertebrate animals, embryos or tissues)**<br>• Provide the name of the Institutional Animal Care and Use Committee (IACUC) or other relevant ethics board that reviewed the study protocol, and indicate whether they approved this research or granted a formal waiver of ethical approval<br>• Include an approval number if one was obtained<br>• If the study involved *non-human primates*, add *additional details* about animal welfare and steps taken to ameliorate suffering<br>• If anesthesia, euthanasia, or any kind of animal sacrifice is part of the study, include briefly which substances and/or methods were applied<br><br>**Field Research**<br><br>Include the following details if this study involves the collection of plant, animal, or other materials from a natural setting:<br>• Field permit number<br>• Name of the institution or relevant body that granted permission	
**Data Availability**<br><br>Authors are required to make all data underlying the findings described fully available, without restriction, and from the time of publication. PLOS allows rare	Yes - all data are fully available without restriction

Powered by Editorial Manager® and ProduXion Manager® from Aries Systems Corporation

exceptions to address legal and ethical concerns. See the PLOS Data Policy and FAQ for detailed information.<br><br>A Data Availability Statement describing where the data can be found is required at submission. Your answers to this question constitute the Data Availability Statement and **will be published in the article**, if accepted.<br><br>**Important:** Stating 'data available on request from the author' is not sufficient. If your data are only available upon request, select 'No' for the first question and explain your exceptional situation in the text box.<br><br>Do the authors confirm that all data underlying the findings described in their manuscript are fully available without restriction?	
**Describe where the data may be found in full sentences. If you are copying our sample text, replace any instances of XXX with the appropriate details.**<br><br>• If the data are **held or will be held in a public repository**, include URLs, accession numbers or DOIs. If this information will only be available after acceptance, indicate this by ticking the box below. For example: *All XXX files are available from the XXX database (accession number(s) XXX, XXX.*).<br>• If the data are all contained **within the manuscript and/or Supporting Information files**, enter the following: *All relevant data are within the manuscript and its Supporting Information files.*<br>• If neither of these applies but you are able to provide **details of access elsewhere**, with or without limitations, please do so. For example:<br><br>*Data cannot be shared publicly because of [XXX]. Data are available from the XXX Institutional Data Access / Ethics Committee (contact via XXX) for researchers who meet the criteria for*	All relevant data are within the manuscript and its Supporting Information files.

access to confidential data.<br><br>*The data underlying the results presented in the study are available from (include the name of the third party and contact information or URL).*<br>• This text is appropriate if the data are owned by a third party and authors do not have permission to share the data.<br><br>* typeset	
Additional data availability information:	

Powered by Editorial Manager® and ProduXion Manager® from Aries Systems Corporation

Manuscript Click here to access/download;Manuscript;Manuscript.docx

In-silico identification, expressional profile and regulatory network analysis of Mitogen Activated Protein Kinase Kinase Kinase gene family in *C. sinensis*

Abhirup Paul[1†], Anurag P. Srivastava[2†], Shreya Subrahmanya[3], Guoxin Shen[4†*], Neelam Mishra[3*]

[1]Department of Biochemistry
REVA University
Bangalore, Karnataka,
India

[2]Department of life Sciences
Garden City University
Bangalore, Karnataka,
India

[3]Department of Botany
St. Joseph's College autonomous
Bangalore, Karnataka,
India

[4]Sericultural Research Institute,
Zhejiang Academy of Agricultural Sciences
Hangzhou 310021, China

[†]These authors contributed equally to this work.

[*]Corresponding authors:

Guoxin Shen, Ph.D., Professor, Tel: +86-571-86404298; Fax: +86-571-86404298

Email address: guoxin.shen@ttu.edu

Neelam Mishra, Ph.D., Assistant professor

Email address: neelamiitkgp@gmail.com; neelammishra@sjc.ac.in

Orcid id:

1. Abhirup Paul: 0000-0003-2143-7511
2. Neelam Mishra: 0000-0001-6191-5392

Abstract

Mitogen activated protein kinase kinase kinase (MAPKKK) form the upstream component of MAPK cascade. It is well characterized in several plants such as Arabidopsis and rice however the knowledge about MAPKKKs in tea plant is largely unknown. In the present study, MAPKKK genes of tea were obtained through a genome wide search using *Arabidopsis thaliana* as the reference genome. Among 59 candidate MAPKKK genes in tea, 17 genes were MEKK-like, 31 genes were Raf-like and 11 genes were ZIK- like. Additionally, phylogenetic relationships were established along with structural analysis, which includes gene structure, its location as well as conserved motifs, cis-acting regulatory elements and functional domain signatures that were systematically examined, and further, predictions were validated by the results. Also, on the basis of orthologous genes in Arabidopsis, functional interaction was carried out in *C. sinensis.* The expressional profiles indicated major involvement of MAPKKK genes from tea in response to various abiotic stress factors. Taken together, this study provides the targets for additional inclusive identification, functional study, and provides comprehensive knowledge for a better understanding of the MAPKKK cascade regulatory network in *C. sinensis*.

Keywords: Mitogen Activated Protein Kinase; *Arabidopsis thaliana*; Phylogenetic relationship; Functional interaction; Abiotic stress

Introduction

Mitogen-activated protein kinase (MAPK) cascades are universal signal transduction modules existing in eukaryotes, including yeasts, animals and plants. MAPKKKs (Mitogen activated protein kinase kinase kinase), which form the upstream component of three tier kinase module are usually activated by G-proteins (Guanine nucleotide binding protein) but sometimes activation is also done via an upstream MAP4K [1]. MAPKKKs are the first component of this phosphorelay cascade, which phosphorylates two serine/threonine residues in a conserved S/T-X_{3-5}-S/T (Serine/Threonine-X_{3-5}-Serine/Threonine) motif of the MKK (Mitogen activated protein kinase kinase) activation loop. MKKs that are dual-specificity kinases, activate the downstream MAPK through TDY or TEY phosphorylation motif in the activation loop (T-loop) [2, 3]. The activated MAPK ultimately phosphorylates various downstream substrates, including transcription factors and other signalling components that regulate the expression of downstream genes [4]. MAPKKKs form the largest group among MAPK cascade, with 80 members in Arabidopsis, 75 members in rice, 74 members in maize and 89 members in tomato [5, 6]. This largest group is further subdivided into three smaller groups on the basis of sequence similarities 1) MEKK subfamily 2) Raf subfamily 3) ZIK subfamily [6, 7]. Compared to MAPKs and MAPKKs, the MAPKKKs have more members and greater variety in primary structures and domain composition [8]. Phylogenetic analysis of the MAPKKK genes in various species reveals the diversity in plants. Among the MAPKKKs, the Raf subfamily is the largest group and comprises of 46 members in maize, 43 in rice, 27 in grapevines, and 48 in Arabidopsis. It is followed by the MEKK subfamily, which is the second largest family and comprises of 22 members in maize, 22 in rice, 9 in grapevine, and 21 in Arabidopsis. The ZIK subfamily is the smallest among the three subfamilies and comprises of 6 members in maize, 10 in rice, 9 in grapevines, and 11 in Arabidopsis [5, 6, 9]. The MEKK subfamily comprises of a conserved kinase domain

G(T/S)Px(W/Y/F)MAPEV [5]. The ZIK subfamily contains TPEFMAPE(L/V)Y while the Raf subfamily has GTxx(W/Y)MAPE as their conserved domain signatures [5]. All the MAPKKK proteins have a kinase domain, and most of them have a serine/threonine protein kinase active site [10]. Structural domain analysis of MAPKKKs in Arabidopsis, rice and cucumber showed that most of the Raf proteins have a C-terminal kinase domain and a long N-terminal regulatory domain. In contrast, members of the ZIK group have the N-terminal kinase domain, while members of the MEKK group have a less conserved kinase domain that lies in either N or C-terminals or present in the central part of the protein [6, 9, 11]. MAPKKKs play a significant role in distinct biological and physiological processes, and they have potential that could be utilized for the development of stress-tolerant transgenic plants [12]. Two of the best studied Arabidopsis MAPKKKs are EDR1 (Enhanced disease resistance) and CTR1 (Constitutive triple response) which are known to participate in defense responses and ethylene signalling respectively [2, 13, 14].

Camellia sinensis more commonly known as tea is the second most consumed beverage in the world besides water. Tea plant is an important commercial crop potentially rich in variety of bioactive ingredients. Many genome wide studies of different gene families have been carried out in tea however, the MAPKKK genes and its role in stress response in tea plant have not been studied in detail. In the present study, the MAPKKK family of genes was thoroughly defined on the basis of *in-silico* genome-wide search in tea using *Arabidopsis thaliana* as the reference genome. Gene locations on scaffolds, their structures, the cis-regulatory elements and their evolutionary aspect were systematically studied. Further, we analysed the interaction networks of proteins based on orthologous genes in Arabidopsis. This study provides an insight on structural and functional aspect of Mitogen Activated Protein Kinase Kinase Kinase gene family in *C. sinensis* and also highlights the MAPK signalling cascade-mediated pathway of *C. sinensis.*

Materials and methods

Identification of MAPKKK gene family in Tea

The predicted peptide sequences of tea were downloaded from the Tea Plant Information Archive (TPIA) database (http://tpia.teaplant.org/) [15]. To identify tea MAPKKK genes, a total of 415 previously known MAPKKK genes were retrieved from *Arabidopsis thaliana* (80), *Oryza sativa* (75), *Solanum lycopersicum* (71), *Solanum tuberosum* (81), *Capsicum annum* (60) and *Coffee canephora* (48) using TAIR database (https://www.arabidopsis.org/) [16], Rice Genome Annotation Project database (http://rice.plantbiology.msu.edu/) [17] and Sol Genomics Network database (https://solgenomics.net/) [18], respectively. The retrieved Arabidopsis and rice MAPKKK sequences were used as query sequences to search against the tea plant proteome database using the BLASTp algorithm with an e value set to 1e-5 and identity percentage of 50% as threshold. The identified sequences were checked to remove any chances of redundancy. Further, the obtained genes were aligned by CLUSTALW (https://www.ebi.ac.uk/Tools/msa/clustalo/) [19] and uploaded to SMART (http://smart.embl-heidelberg.de/) [20] and Pfam web tool (https://pfam.xfam.org/) to confirm the existence of kinase domains. The physicochemical properties of the identified tea MAPKKK genes were predicted using ProtParam tool incorporated in ExPASy database (https://expasy.org/) [21]. Subcellular localization of the peptides were predicted using the BaCelLo (Balanced subcellular localization predictor) (http://gpcr.biocomp.unibo.it/bacello/index.htm) [22] and TMHMM server v2.0 (http://www.cbs.dtu.dk/services/TMHMM/) [23] was employed to predict the presence of trans-membrane helices in tea MAPKKK peptide sequences.

Estimation of K_a/K_s ratios

K_a and K_s ratios were calculated using the SNAP v.2.1.1 online tool (https://www.hiv.lanl.gov/content/sequence/SNAP/SNAP.html) [24] to assess the synonymous and non-synonymous groups. The dN/dS values represent the selective pressure of duplicate genes and the dS values represent the time of divergence of duplication events.

Multiple sequence alignment and Phylogeny analysis

The tea MAPKKK protein sequences were subjected to multiple sequence alignment, using CLUSTALW (https://www.ebi.ac.uk/Tools/msa/clustalo/) [19] to check for conserved MAPKKK specific domains for each subfamily. Phylogenetic analyses were done separately for MEKK, Raf and ZIK sub-families, using the identified tea sequences, coupled with Arabidopsis, rice, tomato, potato, capsicum and coffee peptide sequences. The phylogenetic trees were constructed by the Neighbor-Joining algorithm of MEGA 7.0.14 [25] keeping all the parameters at default values. The consistencies of the obtained trees were assessed by the bootstrap method and replicate was set to 1000.

Intron exon structures and conserved motifs

The intron exon distribution pattern for tea MEKK, Raf and ZIK peptide sequences were analysed and visualised using the Gene Structure Display Server v2.0 (http://gsds.cbi.pku.edu.cn/) [26]. The full-length peptide sequences were uploaded to MEME suite (http://meme-suite.org/) [27] in-order to identify the conserved motifs.

Analysis of cis-regulatory elements

The promoter sequences of 2000 bp, which lies upstream of the translational start site of each of the tea MAPKKK genes were retrieved from the TPIA database. The PlantCARE database (http://bioinformatics.psb.ugent.be/webtools/plantcare/html/) [28, 29] was used for identifying and analysing the cis-acting regulatory elements in the promoter regions of the tea MAPKKK genes.

Mapping of tea MAPKKK genes onto scaffolds and gene duplication

TPIA database has incomplete genome assembly information. As a result, the tea MAPKKK genes were mapped onto their respective scaffolds using MapGene2chromosome web v2 (MG2C) software tool (http://mg2c.iask.in/mg2c_v2.0/) [30]. The genes were mapped according to their scaffold positional information available in TPIA database, which includes scaffold IDs for each gene, scaffold dimensions and the starting and ending position of each gene on the scaffolds.

GO ontology annotation and functional interaction network

GO Ontology (GO) analysis was also performed for all the tea MAPKKKs using QuickGO (QuickGO (ebi.ac.uk)). Furthermore, the network of functionally interacting orthologous genes between tea and Arabidopsis was identified and constructed using STRING online tool ((https://string-db.org/) [31] with default parameters.

Expression profiles of tea MAPKKK genes

The tissue specific expression profiles, which include expression levels in apical bud, flower, fruit, young leaf, mature leaf, old leaf, root, and stem were retrieved from TPIA database. Furthermore, gene expression data under different abiotic stress (cold, drought, salt) treatment as well as under methyl jasmonate (MeJA) treatment were retrieved from TPIA database. GraphPad Prism 8 (https://www.graphpad.com/scientific-software/prism/) was used to generate respective graphs for the gene expression data of MEKK, Raf and ZIK sub-families.

Results

Identification of MAPKKK gene family in *C. sinensis*

In order to identify the MAPKKK gene family in tea (*C. sinensis*), 415 known MAPKKK peptide sequences from *Arabidopsis thaliana* (80), *Oryza sativa* (75), *Solanum lycopersicum* (71), *Solanum tuberosum* (81), *Capsicum annum* (60) and *Coffee canephora* (48) were retrieved from their respective databases. To identify and categorize the MAPKKK genes in tea, BLASTp searches were conducted against the tea protein database, using the retrieved peptide sequences from Arabidopsis and rice as query sequences. For all BLASTp searches, e value and identity percentage were set to 1e-5 and 50% as threshold, respectively (S1-S3 Table). The identified tea peptides were again screened with a Hidden Markov Model (HMM) search to confirm the presence of serine/threonine-protein kinase-like domain (PF00069). The results yielded a total of 59 potential tea MAPKKK genes, which included 17 MEKK-like, 31 Raf-like and 11 ZIK-like genes and were incorporated into the final dataset.

The physicochemical properties of the identified tea MAPKKK protein sequences were evaluated using ExPASy ProtParam tool (Table 1-3). The length and molecular weight of the 17 MEKK proteins ranged from 311 to 1191 amino acid residues and 34828.88 to 130956.46 kDa, respectively (Table 1). For the Raf proteins, it ranged from 305 to 1436 amino acid residues and 35012.57 to 159263.21 kDa (Table 2), and for the ZIK proteins, it ranged from 300 to 831 amino acid residues and 34181.96 to 94422.51 kDa (Table 3). The theoretical pI values ranged from 4.58 to 9.50 for MEKK, 4.88 to 9.61 for Raf and 5.14 to 6.33 for ZIK proteins, indicating that most of the MEKK and Raf proteins have a basic nature while the

ZIK proteins are acidic in nature. The grand average of hydropathy (GRAVY index) in all the extracted MEKK, Raf and ZIK proteins were negative, ranging from -0.605 to -0.060, -0.661 to -0.182 and -0.582 to -0.350 respectively. This indicates that all the identified 59 tea MAPKKKs are hydrophilic in nature. 52 of the 59 putative tea MAPKKKs had instability index values above 40, while 6 Raf genes (TEA000933.1, TEA022171.1, TEA011280.1, TEA031223.1, TEA007232.1 and TEA013875.1) and 1 ZIK gene (TEA020112.1) had instability index values less than 40 (Table 1-3). This signifies the unstable nature of most of the identified tea MAPKKKs. Subcellular localization predicted 48 genes being localized in the nucleus, 9 genes in chloroplast and 2 genes in cytoplasm (Table 1-3). The presence of trans-membrane helices in the putative peptide sequences was also done and one of the ZIK gene (TEA027328.1) had one trans-membrane helix (S1-S3 Figs).

Table 1. Sequence characteristics and physicochemical properties of MAPKKKs belonging to MEKK subfamily in *C. sinensis.* Locus position, gene length, protein length, molecular weight and pI value, no. of negative and positive residues, GRAVY index, instability index, aliphatic index and subcellular localizations were analysed.

Gene ID	Locus position	Gene length (bp)	Protein length (aa)	Mol. Wt. (kDa)	pI value	No. of negative residues	No. of positive residues	GRAVY index	Instability index	Aliphatic index	Subcellular localization
TEA028357.1	Scaffold856:196999-204246-	7247	628	68667.76	5.60	77	67	-0.380	58.36	76.85	Nucleus
TEA025870.1	Scaffold790:521648-539960+	18312	776	85271.15	6.76	94	92	-0.379	45.58	81.08	Nucleus
TEA016319.1	Scaffold3144:371539-383072-	11533	627	68238.67	9.50	53	71	-0.535	50.61	68.23	Nucleus
TEA008165.1	Scaffold3102:729210-737275+	8065	1032	112285.36	9.04	84	102	-0.423	53.62	72.95	Nucleus
TEA027265.1	Scaffold1289:966535-975893+	9358	939	101539.85	9.35	80	104	-0.605	63.34	65.88	Nucleus
TEA006319.1	Scaffold2905:735285-744378+	9093	683	75479.57	9.32	62	78	-0.505	67.84	72.55	Chloroplast
TEA006473.1	Scaffold1374:1527992-1535696-	7704	710	78857.59	9.09	65	79	-0.516	69.53	71.59	Nucleus
TEA014429.1	Scaffold41:2381991-2415462+	33471	1191	130956.46	6.13	145	128	-0.350	45.47	89.93	Chloroplast
TEA031711.1	Scaffold5399:986467-998883-	12416	562	62129.83	6.31	72	69	-0.484	48.47	75.62	Nucleus
TEA001470.1	Scaffold558:920549-933450+	12901	789	87423.22	8.34	90	95	-0.313	49.68	84.13	Nucleus
TEA017119.1	Scaffold5354:234291-239017-	4726	506	56190.19	4.66	80	49	-0.481	47.12	69.53	Nucleus
TEA005306.1	Scaffold2184:2097399-2125258+	27859	1097	121164.56	5.40	162	127	-0.540	49.78	72.63	Nucleus
TEA009902.1	Scaffold438:521469-522821-	1352	450	49874.37	4.58	65	34	-0.060	44.64	91.60	Chloroplast
TEA029598.1	Scaffold944:301732-304329+	2597	423	46235.51	4.94	62	43	-0.433	51.54	74.18	Nucleus
TEA005122.1	Scaffold1857:297670-298674-	1004	334	36588.08	6.01	40	34	-0.381	46.55	78.23	Chloroplast
TEA028214.1	Scaffold613:628014-629048+	1034	344	38088.50	6.33	44	41	-0.322	45.18	79.36	Nucleus
TEA031689.1	Scaffold1549:309791-310726-	935	311	34828.88	6.04	44	40	-0.340	48.20	90.64	Nucleus

Table 2. Sequence characteristics and physicochemical properties of MAPKKKs belonging to Raf subfamily in *C. sinensis*. Locus position, gene length, protein length, molecular weight and pI value, no. of negative and positive residues, GRAVY index, instability index, aliphatic index and subcellular localizations were analysed.

Gene ID	Locus position	Gene length (bp)	Protein length (aa)	Mol. Wt. (kDa)	pI value	No. of negative residues	No. of positive residues	GRAVY index	Instability index	Aliphatic index	Subcellular localization
TEA001765.1	Scaffold1670:382409-407933-	25524	842	93193.15	5.86	107	92	-0.248	46.33	89.69	Nucleus
TEA002020.1	Scaffold3595:726640-735244+	8604	896	99135.31	6.37	111	103	-0.382	42.96	81.80	Nucleus
TEA000256.1	Scaffold3876:193108-215389+	22281	1086	119081.90	6.63	118	112	-0.441	44.80	78.36	Nucleus
TEA029086.1	Scaffold106:745738-778269+	32531	919	101696.51	5.17	119	85	-0.182	41.82	91.44	Chloroplast
TEA022129.1	Scaffold3036:784237-806418+	22181	940	104852.31	6.01	114	102	-0.217	48.52	89.81	Nucleus
TEA019143.1	Scaffold1695:623368-630213+	6845	724	79987.28	7.68	86	87	-0.609	41.33	70.98	Nucleus
TEA028452.1	Scaffold433:2415340-2426547+	11207	846	93141.05	6.10	107	93	-0.523	46.31	70.89	Nucleus
TEA016969.1	Scaffold4925:453439-477111+	23672	1107	124661.25	8.46	145	153	-0.506	46.58	77.06	Nucleus
TEA013270.1	Scaffold344:585774-608400+	22626	755	85320.07	5.83	104	84	-0.374	53.97	80.97	Nucleus
TEA026716.1	Scaffold1930:511463-522712-	11249	368	41783.40	5.63	52	44	-0.487	46.26	73.89	Nucleus
TEA028758.1	Scaffold9739:380569-387825-	7256	1213	135047.30	5.63	159	123	-0.661	51.62	66.13	Nucleus
TEA010804.1	Scaffold35:1009695-1024064+	14369	305	35012.57	6.50	44	41	-0.644	44.62	74.16	Nucleus
TEA009451.1	Scaffold1786:773656-783180-	9524	1333	148469.64	4.88	193	127	-0.547	45.20	72.24	Nucleus
TEA021421.1	Scaffold1504:1005366-1017261-	11895	1331	147137.87	5.50	181	135	-0.618	44.79	71.96	Nucleus
TEA017670.1	Scaffold1965:485241-501001+	15760	561	62970.57	5.67	78	62	-0.385	49.24	89.63	Nucleus
TEA019184.1	Scaffold4191:163416-174412+	10996	601	67890.36	5.90	76	65	-0.328	49.47	89.02	Nucleus
TEA000933.1	Scaffold397:63694-76812+	13118	407	45660.50	7.66	45	46	-0.291	38.27	81.89	Nucleus
TEA031230.1	Scaffold2248:916505-925818+	9313	489	54655.73	9.20	56	67	-0.311	43.48	87.32	Chloroplast
TEA022171.1	Scaffold382:2039496-2046332+	6836	404	44640.23	8.60	48	54	-0.379	26.52	78.89	Nucleus
TEA011280.1	Scaffold3804:571784-578194+	6410	368	41126.17	7.52	48	49	-0.312	25.58	82.91	Nucleus
TEA031223.1	Scaffold2248:107085-111327-	4242	434	48234.42	6.42	57	55	-0.280	26.84	84.22	Nucleus
TEA007232.1	Scaffold3038:2387807-2395630-	7823	368	41118.03	7.02	48	48	-0.424	35.65	77.34	Nucleus
TEA016553.1	Scaffold1761:1968012-1984037-	16025	432	49062.23	8.45	65	69	-0.513	43.90	83.06	Nucleus
TEA033032.1	Scaffold858:331961-346154-	14193	415	46688.59	6.05	59	52	-0.355	43.89	86.53	Nucleus
TEA001764.1	Scaffold619:1545624-1550286+	4662	351	39474.64	6.47	42	39	-0.191	43.90	86.72	Cytoplasm
TEA026000.1	Scaffold3457:1062923-1073461-	10538	1296	144765.39	5.40	177	122	-0.507	42.22	73.72	Nucleus
TEA033556.1	Scaffold192:400250-403690-	3440	541	61612.16	9.27	57	72	-0.371	46.58	86.19	Chloroplast
TEA013875.1	Scaffold5449:126808-131150+	4342	341	39047.30	6.76	44	42	-0.256	36.12	91.52	Cytoplasm
TEA002722.1	Scaffold1369:145416-156759+	11343	1436	159263.21	5.41	203	153	-0.566	45.20	72.12	Nucleus
TEA030052.1	Scaffold319:1148438-1156900+	8462	1357	148194.52	5.00	166	110	-0.444	50.17	73.96	Nucleus
TEA008343.1	Scaffold142:344598-352450+	7852	334	37992.05	9.61	35	46	-0.257	46.78	86.17	Nucleus

Table 3. Sequence characteristics and physicochemical properties of MAPKKKs belonging to ZIK subfamily in *C. sinensis.* Locus position, gene length, protein length, molecular weight and pI value, no. of negative and positive residues, GRAVY index, instability index, aliphatic index and subcellular localizations were analysed.

Gene ID	Locus position	Gene length (bp)	Protein length (aa)	Mol. Wt. (kDa)	pI value	No. of negative residues	No. of positive residues	GRAVY index	Instability index	Aliphatic index	Subcellular localization
TEA010125.1	Scaffold52:664232-675917-	11685	675	76706.84	5.67	91	68	-0.546	47.51	72.79	Nucleus
TEA022762.1	Scaffold9600:223976-236388+	12412	732	83655.02	5.87	103	83	-0.419	50.69	89.07	Nucleus
TEA024720.1	Scaffold1050:31289-41391+	10102	655	74571.17	6.33	89	81	-0.518	51.00	77.68	Nucleus
TEA002087.1	Scaffold754:205321-211058-	5737	719	81783.51	5.54	108	80	-0.582	42.80	75.41	Nucleus
TEA013346.1	Scaffold5883:262251-272009+	9758	831	94422.51	5.53	124	93	-0.504	43.60	79.06	Nucleus
TEA013344.1	Scaffold5883:191507-194485+	2978	481	55933.38	5.65	72	58	-0.472	40.70	80.04	Nucleus
TEA031068.1	Scaffold1571:837990-857219+	19229	762	86343.00	5.92	98	76	-0.375	40.49	85.07	Chloroplast
TEA020698.1	Scaffold2762:535605-540535-	4930	664	75716.56	5.63	95	74	0.439	46.00	81.91	Nucleus
TEA027328.1	Scaffold688:688353-693133+	4780	748	84782.46	5.48	100	81	-0.350	42.68	80.16	Chloroplast
TEA020112.1	Scaffold1093:624579-626760+	2181	300	34181.96	5.60	47	40	-0.428	33.33	85.47	Nucleus
TEA033250.1	Scaffold4160:2129637-2136050+	6415	622	69665.68	5.14	99	74	-0.449	43.80	84.00	Nucleus

Phylogenetic analysis of tea MAPKKKs

A phylogenetic analysis of the putative tea MAPKKK genes was carried out to evaluate the evolutionary relationships. MEGA 7.0.14 was used to generate the phylogenetic trees, using the Neighbor-Joining (NJ) algorithm, at default parameters and 1000 bootstrap replicates. Three different phylogenetic trees were constructed for MEKK, Raf and ZIK proteins, comprising of the identified tea sequences and already known 415 MAPKKK sequences from Arabidopsis, rice, tomato, potato, capsicum and coffee. For MEKK, the NJ tree was generated using 17 sequences from tea, 21 sequences from Arabidopsis, 22 sequences from rice, 17 sequences from tomato, 22 sequences from potato, 17 sequences from capsicum and 12 sequences from coffee (Fig 1A). The NJ tree was divided into 4 distinct clades, with an uniform distribution of genes in Clade A. Clade B consisted of only 6 capsicum genes while

clade D had only 2 genes of potato. Clade C however, had a share of tomato and potato gene clusters. For Raf, the NJ tree was generated using 31 sequences from tea, 48 sequences from Arabidopsis, 43 sequences from rice, 44 sequences from tomato, 43 sequences from potato, 37 sequences from capsicum and 28 sequences from coffee (Fig 1B). Unlike the MEKK tree, the Raf tree was divided into 11 different clades, with an uniform clustering of genes in all the clades. The NJ tree for ZIK was generated using 11 sequences from tea, 11 sequences from Arabidopsis, 10 sequences from rice, 10 sequences from tomato, 16 sequences from potato, 6 sequences from capsicum and 8 sequences from coffee (Fig 1C). The ZIK tree was divided into 7 clades and had a uniform clustering of genes in all the clades with only clade E consisting of 2 genes each of Arabidopsis and rice. The results are suggestive of the fact that the genes are either homologous or orthologous to each other. However, the Raf and ZIK genes did not feature any orthologous gene with respect to Arabidopsis. This was validated only when the Raf and ZIK gene accession IDs were scanned in the TPIA database to search for the presence of orthologous genes in the later part of the study (Functional Interaction Network).

Fig 1. Phylogenetic tree of (A) MEKK-like (B) Raf-like and (C) ZIK-like genes from *Arabidopsis thaliana* (black), *C. sinensis* (red), *Oryza sativa* (blue), *Solanum lycopersicum* (grey), *Solanum tuberosum* (green), *Capsicum annum* (brown), *Coffee canephora* (teal). The full-length MEKK, Raf and ZIK protein sequences were aligned using Clustal W, and the phylogenetic trees were constructed using MEGA 7.0.14 by the Neighbor-Joining (NJ) method with default parameters and 1000 bootstrap replicates.

Domain analysis of tea MAPKKKs

Among the 3 subgroups of plant MAPKKKs, the MEKK subfamily is fairly well known and characterized. Most MEKKs are known to be a part of the recognized MAP Kinase cascades, which activates the downstream MKKs. MEKK1 and MEKK2 from Arabidopsis, have been proven to play a significant role in plant innate immunity [32, 33, 34]. Similar to other plant

MAPKKKs, 16 out of 17 members of MEKK subfamily in tea displayed a characteristic conserved signature G(T/S)Px(W/Y/F)MAPEV, except TEA014429.1 (Fig 2A). Two of the most widely studied Arabidopsis Raf subfamily MAPKKKs, namely CTR1 and EDR1 are known to actively participate in ethylene mediated signalling and defense response mechanisms. All 31 members of the Raf subfamily in tea featured a conserved GTxx(W/Y) MAPE signature in its kinase domain with no exceptions (Fig 2B). The ZIK-like MAPKKKs are also known by the name WNK or with no lysine (K). They are not proven to be involved with the phosphorylation of the MKKs in plants but have specific functions. Arabidopsis ZIK1 is known to phosphorylate APRR3 *in-vitro*, which is a putative component of the circadian clock in plants and is believed to be involved in signal transduction pathway, regulating its biological activity [35]. Another ZIK cascade, involving ZIK2, ZIK5 and ZIK8 in Arabidopsis is known to regulate the flowering time by modulating the photoperiod [36]. The ZIK subfamily featured a characteristic GTPEFMAPE(L/V/M)(Y/F/L) conserved signature across all its 11 members in tea (Fig 2C) [5, 6]. The presence of these distinctive conserved signatures across the tea MAPKKKs further confirms identity and the subfamily they belong. The largest subfamily was found to be the Raf subfamily with 31 members, while the smallest was found to be the ZIK subfamily with only 11 members. These results are consistent with published reports on other plant MAPKKKs [5, 6].

Fig 2. Alignment of MAPKKKs of (A) MEKK subfamily (B) Raf subfamily and (C) ZIK subfamily in *C. sinensis*. ClustalX program was used for aligning the obtained sequences. The highlighted part (G(T/S)Px(F/Y/W)MAPEV) shows the conserved signature for the MEKK proteins. The highlighted section (GTxx(W/Y)MAPE) shows the conserved signature for the Raf proteins and the highlighted part (GTxx(W/Y)MAPE) shows the conserved signature for the ZIK proteins.

Motif composition of tea MAPKKKs

To understand the evolution and comprehend sequential characteristics of the MAPKKK proteins in tea, a conserved motif search was carried out using the MEME suite (Fig 3). Ten conserved motifs were identified in each of the three subfamilies. Almost all the tea MAPKKK proteins featured the protein kinase domain with motif 1, motif 2 and motif 3. Motif 4 was conserved across all the proteins with only one exception of TEA031230.1. Motif 5, motif 7 and motif 8 were only obtained for the ZIK subfamily with one exception of a MEKK-like TEA014429.1, which featured motif 8. Motif 6 and motif 9 were harboured by almost all the protein sequences. However, motif 10 was only specific to the MEKK and Raf subfamilies. Motif annotation revealed that motif 2 harboured a protein kinase ATP-binding site. Motif 6 contained a tyrosine kinase phosphorylation site. Motif 9 featured a serine/threonine protein kinase activation site (S4 Fig). The results suggested that proteins belonging to a same group harboured similar conserved motifs, further indicating that the classification of the tea MAPKKK subfamilies was backed by motif analyses.

Fig 3. The motif analysis of 59 identified MAPKKKs in *C. sinensis*. The motif figures were generated by MEME suite. A total of 10 motifs were identified and are marked individually.

Gene structure analysis of tea MAPKKKs

The intron-exon distribution pattern for tea MAPKKKs were analysed and visualised using the Gene Structure Display Server v2.0. Study of gene structure revealed differences in number of introns and exons, which contributes to variation in gene length. Introns or non-coding sequences are found abundantly within a genome and are regarded as an indicator of genome complexity [37, 38]. Analysis of the intron patterns could help to comprehend and provide insights into the evolution, function and regulation of the genes [37, 39, 40, 41, 42]. The analysis of the intron-exon architecture in tea revealed significant variation in the

number of introns and exons among the three subfamilies of MAPKKKs (Fig 4). However, genes belonging to the same clades had similar intron-exon distribution. The MEKK subfamily had 10 out of 17 genes (59% of the MEKK genes) possessing 6 to 16 exons (Fig 4A). TEA025870.1 had 19 exons and 18 introns in its gene. Two genes possessed 2 exons and 1 intron and the remaining 4 genes had no introns. Only 9 out of 17 genes featured UTR (Untranslated Regions) segments and 5 out of these 9 genes featured both 5' and 3' UTRs. 3 genes contained only the 5' UTR segments and 1 gene only had the 3' UTR segment. The genes belonging to the Raf subfamily had exons ranging from 6 to 18 and was featured by 27 out of 31 genes (87% of the Raf genes) (Fig 4B). TEA016969.1 featured a staggering 28 exons and was the highest among all the Raf genes. Three genes namely TEA000933.1, TEA013875.1 and TEA033556.1 had 2, 3 and 4 exons respectively which were the lowest number of exons found amongst all the Raf genes. 29 out of 31 genes possessed UTR segments. However, only 17 of the 29 genes had both 5' and 3' UTRs. 7 genes featured only the 5' UTR segment and remaining 5 genes only had the 3' UTR. Unlike the MEKK and Raf subfamilies, ZIK subfamily displayed a certain level of conservancy with respect to the number of exons and introns. 10 out of 11 ZIK genes (91% of the ZIK genes) had exons ranging from 7 to 10 (Fig 4C). TEA020112.1 however featured only 2 exons. 9 out of 11 genes possessed UTR segments and 5 of them had both 5' and 3' UTRs. 4 genes featured only the 5' UTR segment. However, no ZIK subfamily gene in tea featured only the 3' UTR segment like the MEKK and Raf subfamilies.

Fig 4. The intron/exon architecture of (A) MEKK (B) Raf and (C) ZIK genes in *C. sinensis.* Gene structure maps were drawn using the Gene Structure Display Server 2.0. Black boxes represent exons, blue boxes represent the UTRs and black lines represent introns. The gene length can be estimated by using the scale (in kb) given at the bottom.

Retrieval of promoter sequences and analysis of cis-regulatory elements

Cis-acting regulatory elements are often used for determining the function of genes, regulation of gene transcription and gene expression [43, 44]. In order to explore the transcriptional regulation and putative functions of the tea MAPKKK genes, promoter sequences of 2000 bp upstream of the initiation codon “ATG” was retrieved from the TPIA database. These sequences were then analysed using the PlantCARE database for the identification of the cis-acting regulatory elements (CAREs). It was found that the cis-acting elements were randomly scattered in the promoter regions of the tea MAPKKKs. The study revealed an aggregate of 56 CAREs in all the tea MEKK, Raf and ZIK genes (S4 Table). These elements were also arranged and grouped based on their specific biological functions (Fig 5A). The sequence length of the cis-acting elements ranged from 5 to 14 bp (Fig 5B) with most of the CAREs having sequence lengths of 6 and 9 bp. The analysis of the 56 CAREs revealed the involvement of 13 elements in plant growth and regulation, 26 in light responsiveness, 6 were stress response elements and the remaining 11 were involved in phytohormone responses. The light responsive elements comprised of the largest section of the identified CAREs in all of the 59 tea MAPKKKs with 26 regulatory elements. Among these, Box-4 and G-Box accounted for the major part in 51 and 45 tea MAPKKKs. Some of the other light response elements included AE box, GATA motif, GT1-motif, MRE and TCT motif. The CAREs related to the phytohormone responses mainly involved abscisic acid responsive element (ABRE), and MeJA responsive elements (CGTCA-motif and TGACG-motif) in 42 and 38 tea MAPKKKs accordingly. Other phytohormone responsive elements comprised of gibberellin responsive elements (TATC-box, P-box and GARE-motif), auxin responsive elements (TGA-element, AuxRR-core, TGA-box and AuxRE) and salicylic acid responsive element (TCA-element) in 35, 33 and 26 tea MAPKKKs respectively. Among the cis-elements which are associated with plant growth and development, 21 tea MAPKKKs possessed meristem expression elements (CAT-box and NON box) while 18 genes had zein

metabolism regulatory element (O2-site). Other plant growth related CAREs included regulatory elements (A-box and Box-II like sequences), endosperm expression element (GCN4_motif), circadian control, cell cycle regulation (MSA-like), Box-III and few seed specific (RY-element), root specific (motif I) and palisade mesophyll differentiation (HD-Zip 1) element that were discovered on the promoter regions of 19, 12, 8, 3, 7, 2, 1 and 1 tea MAPKKKs respectively. In addition, numerous stress response CAREs were also identified in the promoter regions. These included ARE (anaerobic induction element), LTR (low temperature responsiveness), TC-rich repeats (defense and stress responsiveness), MBS (drought inducibility), GC-motif (anoxic specific inducibility) and AT-rich sequences (maximal elicitor mediated activation) in 52, 19, 25, 18, 8 and 6 tea MAPKKKs respectively. These results indicate the involvement of MAPKKK genes in various responses like phytohormone treatments, low temperature, physiological stresses and plant growth and regulation.

Fig 5. Analysis of cis-acting elements identified from the MEKK, Raf and ZIK genes of *C. Sinensis*. All cis-acting elements have been identified using PlantCARE database. (A) Pie-chart showing the frequency of different cis-acting elements based on their specific biological activities. (B) Histogram showing the frequency of different sequence lengths of the cis-acting elements.

Genomic distribution map and evolutionary pressure on tea MAPKKKs

The tea MAPKKKs were mapped onto the genomic scaffolds to understand their distribution pattern. Due to the lack of chromosome-level assembly data in the TPIA database, the genes were mapped onto their respective scaffolds instead of the chromosomes. All 59 tea MAPKKKs were extensively distributed across 58 different genomic scaffolds. 17 MEKK genes were distributed across 17 different scaffolds (Fig 6A). Similarly, 31 Raf genes were distributed across 31 genomic scaffolds (Fig 6B). 11 ZIK genes were mapped onto 10 genomic scaffolds (Fig 6C). Two ZIK genes namely, TEA013344.1 and TEA013346.1 were

mapped on the same genomic scaffold 5883 and thus featured a duplication event. Additionally, both these genes possessed similar intron-exon architecture. This result is conclusive evidence that duplication events were of significant importance and played a crucial role in the expansion of the MAPKKK genes in *C. sinensis* genome. Further, the ratio of non-synonymous substitution rates (K_a) and synonymous substitution rates (K_s) was evaluated to illuminate the mechanism of gene divergence and evolutionary pressure on the tea MAPKKKs. The ratio determines the selective pressure acting on the respective proteins. If the K_a/K_s ratio is <1, it determines negative or purifying selection. If the K_a/K_s ratio is =1, it indicates neutral selection and if the K_a/K_s ratio is >1, it signifies positive selection [45]. For the MEKK subfamily, pair wise comparisons revealed that 72 gene pairs had K_a/K_s ratios above 1, indicating that they are under positive selection, 24 gene pairs had values less than 1, indicating a negative selection and remaining 40 were not a number (Nan) (S5 Table). Similarly, K_a/K_s ratios of the Raf subfamily revealed 341 gene pairs in positive selection, 96 in negative selection and 28 pairs as Nan (S6 Table). K_a/K_s ratios of ZIK subfamily uncovered 30 pairs in positive selection, 21 in negative selection and the remaining 4 as Nan (S7 Table). The K_a/K_s cumulative graphs of tea MAPKKKs were also generated (S5-S7 Figs). The results suggest strong positive selection pressures would have occurred, enabling different factors to regulate the MAPKKKs in *C. sinensis*.

Fig 6. The scaffold distribution of (A) MEKK subfamily (B) Raf subfamily and (C) ZIK subfamily genes in *C. sinensis*. MapGene2chromosome web v2 (MG2C) software tool (http://mg2c.iask.in/mg2c_v2.1/) was used to map genes onto their respective scaffolds. The scaffolds are drawn to scale and the scaffold numbers are indicated on the top.

GO ontology analysis and functional interaction network of tea MAPKKKs

The GO ontology analysis was performed in order to predict the potential functions of all the 59 tea MAPKKKs (S8 Fig). All the MEKK, Raf and ZIK proteins were assigned into three

major groups and 50 subgroups. The 3 major groups were biological process, cellular component and molecular function. In the first group, the proteins were distributed into 29 subgroups with ‘protein phosphorylation’ (GO:0006468, 26 sequences, 44.07%) with the largest representation. In the cellular component group, the MAPKKK proteins were distributed into 8 subgroups. Among these 8, ‘cytosol’ (GO:0005829, 4 sequences, 6.78%) had the highest representation followed by ‘cytoplasm’ (GO:0005737, 3 sequences, 5.08%) and ‘intracellullar’ (GO:0005622, 3 sequences, 5.08%). The molecular function group featured 16 subgroups with ‘ATP binding’ (GO:0005524, 28 sequences, 27.12%) and ‘protein kinase activity’ (GO:0004672, 25 sequences, 42.37%) having highest representation. They were followed by ‘protein serine/threonine kinase activity’ (GO:0004674, 16 sequences, 27.12%) and ‘kinase activity’ (GO:0016301, 13 sequences, 22.03%).

For better understanding of the interactions of MAPKKKs in *C. sinensis*, an interaction network was constructed based on the orthologous genes in Arabidopsis, using the STRING server (Fig 7). The functional interaction network of the genes has been built using that of Arabidopsis since tea database is not included in the STRING online server. TEA005306.1 in tea was found to be orthologous to AT5G55100 in Arabidopsis. This orthologous gene was identified using the TPIA database and AT5G55100 was used to build the interaction network. Additionally, tea proteins, homologous to the Arabidopsis proteins participating in the network were incorporated in the network (Fig 7). These homologous proteins were designated as STRING proteins and were selected on the basis of high bit scores. Similarity search programs like BLAST are widely used and produce accurate statistical estimates, ensuring that protein sequences with significant similarity also have similar structures [46]. Proteins that have high sequence and structural similarity generally tend to possess similar functions [47]. Based on TAIR database, AT5G55100 is involved in RNA processing and is expressed during 15 growth stages in 24 different organs and tissue of plant. It shows

interactions with AT4G33690 which is involved in biological process of protein binding [16]. AT2G29210 is involved with RNA splicing, mRNA processing and is expressed during 13 different growth stages in 23 different organs and tissues [16]. ATO (AT5G06160) encodes for a protein similar to pre-mRNA splicing factor SF3a60 and is involved in gametic cell fate determination [16]. Loss of function results in the ectopic expression of egg cell makers, thereby suggesting a role in restriction of gametic cell fate. TEA031585.1, which is homologous to ATO gene, is a part of the spliceosomal complex and is involved in mRNA splicing based on GO ontology. TK1 (AT2G36960) is a TSL-kinase interacting protein and is involved in protein binding [16]. It is expressed in 14 developmental stages in 25 different plant organs and tissue. GPT (Glucose-6-Phosphate translocator) (AT2G41490) is an integral component of membrane and has a UDP-N-acetylglucosamine-dolichyl-phosphate N-acetylglucosamine phosphotransferase activity [16]. It is expressed during 15 developmental stages in 23 different organ and tissue in the plant. AT3G57220 is located in the endoplasmic reticulum and has a UDP-N-acetylglucosamine-dolichyl-phosphate N-acetylglucosamine phosphotransferase activity. It is also linked with polysaccharide biosynthesis and is expressed during 10 growth stages in 16 different plant tissue and organ [16]. According to GO ontology, TEA009318.1 is also involved in phosphotransferase activity in tea and is homologous to both AT2G41490 and AT3G57220 in Arabidopsis.

Fig 7. Functional interaction network of tea MAPKKK proteins. The interaction network was build according to the ortholog in Arabidopsis. TEA005306.1 in tea is orthologous to AT5G55100 in Arabidopsis. The orthologous protein (red) and homologous proteins (black) are shown within brackets.

Tissue specific gene expression of tea MAPKKKs

The tissue specific expression pattern of the tea MAPKKK genes in various plant tissues were retrieved from the TPIA database where levels of expression were expressed using

transcripts per million (TPM). The TPIA database houses tissue specific expression data for 8 different plant tissues which includes apical bud, flower, fruit, young leaf, mature leaf, old leaf, root and stem (S8 Table). Among the 59 tea MAPKKK genes, expression data for 58 genes were retrieved with an exception of 1 MEKK gene, TEA031689.1. All 58 genes displayed varied levels of expression, with few of the transcripts barely readable (Fig 8). For the MEKK genes, the maximum level of expression in apical bud was shown by TEA006319.1. This gene also marked the highest level of expression in young leaf. TEA017119.1 showed highest level of expression in flower. TEA016319.1 displayed highest expression levels in fruit, mature leaf, old leaf and stem. TEA005122.1 was expressed maximum in root. TEA028357.1 and TEA009902.1 had negligible levels of expression in all of the 8 plant tissues (Fig 8A). For the Raf genes, TEA000933.1 showed highest levels of expression in apical bud, fruit, young leaf, mature leaf, old leaf, root and stem. TEA007232.1 was expressed maximum in flower. However, TEA001765.1, TEA013270.1, TEA028758.1 and TEA031230.1 had negligible levels of expression (Fig 8B). Finally, for the ZIK genes, TEA002087.1 displayed highest levels of expression in apical bud, flower, young leaf and stem. TEA022762.1 had highest levels of expression in fruit, mature leaf and old leaf. TEA020112.1 showed maximum expression in root. However, TEA013344.1, TEA031068.1, TEA020698.1 and TEA027328.1 showed minor levels of expression (Fig 8C). Heat maps for all the 58 genes, representing the tissue specific expression levels were also being generated (S9 Fig).

Fig 8. Tissue-specific expression patterns of (A) MEKK (B) Raf and (C) ZIK genes in *C. sinensis*. The relative expression of these genes were analysed in different tissues by using GraphPad Prism 8 software. The level of expression was in transcript per million (TPM). 58 out of the 59 identified genes had expression data in TPIA database with an exception of 1 MEKK gene (TEA031689.1).

Abiotic stress induced differential expression levels of tea MAPKKKs

The expression data of the leaves of the tea plant present in TPIA database (S9-S12 Table) was used in order to study the effect of cold, drought, salt stress along with methyl jasmonate (MeJA) treatment followed by the generation of expression graphs for the same (Fig 9-12). The cold acclimated (CA) data (unpublished), present in the TPIA database consists of 5 stages of expression. These are: 1. 25~20 °C (CK), 2. Fully acclimated at 10 °C for 6 h (CA1-6h) 3. 10~4 °C for 7 days (CA 1-7d), 4. Cold response at 4~0 °C for 7 days (CA 2-7d) and 5. Recovering under 25~20 °C for 7 days (DA-7d), where CK is the control [48]. Expression of MEKK genes revealed that 15 out of 17 genes were upregulated under CA 1-6h. TEA006473.1 was downregulated while TEA031689.1 displayed no expression levels. Expression levels under the CA 1-7d condition showed that 12 genes were upregulated, 4 genes were downregulated and remaining 1 gene showed no data. Under the CA 2-7d condition, expression levels revealed that 10 genes were upregulated, 6 genes were downregulated and remaining 1 gene displayed no expression data. Lastly, under the DA-7d condition, data revealed that 13 genes showed upregulation, 3 genes showed downregulation and 1 gene had no data (Fig 9A). The Raf and ZIK genes were also analysed based on the same 5 conditions. For the Raf genes, under CA 1-6h condition, 22 genes out of 31 were upregulated and 9 genes were downregulated. Under CA 1-7d condition, 16 genes were upregulated and 15 genes were downregulated. Expression levels under CA 2-7d revealed that 17 genes showed upregulation and remaining 14 genes showed downregulation. Under DA-7d condition, 21 genes were upregulated and 10 genes were downregulated (Fig 9B). Expression data of the ZIK genes revealed that under CA 1-6h, 7 out of 11 genes were upregulated and 4 genes were downregulated. CA 1-7d condition revealed that 5 genes were upregulated, 5 genes were downregulated and remaining 1 gene displayed no expression. Under CA 2-7d condition, 4 genes were upregulated, 6 genes were downregulated and 1 gene had no expression. Finally, under DA-7d, 8 genes showed upregulation and remaining 3

showed downregulation (Fig 9C). Heat maps for the retrieved expression data were also generated (S10 Fig).

Further, expression levels of all tea MAPKKKs were checked under drought stress conditions. Expression levels under drought stress are available in the TPIA database with respect to 25% polyethylene glycol (PEG) treatment and it includes 4 different stages: 1. 0h; 2. 24h; 3. 48h; and 4. 72h [49], where 0h was taken as the control. The expression levels of MEKK genes revealed that under PEG-N-24h condition, 12 genes were upregulated, 4 were downregulated and 1 gene did not show any expression. Under PEG-N-48h, 12 genes were upregulated, 4 were downregulated and 1 gene showed no expression. PEG-N-72h revealed 11 genes showing upregulation, 5 genes showing downregulation and 1 gene with no expression (Fig 10A). Expression of Raf genes showed that under the PEG-N-24h condition, 11 genes were upregulated, 20 genes were downregulated. Under PEG-N-48h, 16 genes showed upregulation while the remaining 15 genes were downregulated. PEG-N-72h revealed that 15 genes were upregulated and 16 genes were downregulated (Fig 10B). Finally, the expression data of ZIK genes revealed 10 out of 11 genes had different expression levels under the given conditions while 1 gene (TEA013344.1) had no data. Under the PEG-N-24h condition, expression data showed that only 1 gene was upregulated while the rest of the genes were downregulated. PEG-N-48h condition too revealed the same result with only 1 gene being upregulated. However, PEG-N-72h showed that 2 genes were upregulated and the rest of the genes were downregulated (Fig 10C). Heat maps for the afore-mentioned data were also generated (S11 Fig).

The expression levels of the tea MAPKKKs under salt stress condition were studied. Similar to the drought stress parameters, the salt stress data in TPIA database is recorded based on treatment with 200 mM NaCl under 4 stages: 1. 0h; 2. 24h; 3. 48h; and 4. 72h where 0h was taken as the control. Analysis of the MEKK genes revealed that under NaCl-N-24h, 9 genes

were upregulated and 8 genes were downregulated. For NaCl-N-48h condition, 9 genes showed upregulation and remaining 8 genes were downregulated. Expression levels under NaCl-N-72h revealed 5 genes being upregulated and the rest being downregulated (Fig 11A). For the Raf genes, expression data suggested that under NaCl-N-24h condition, 15 genes were upregulated and 16 genes were downregulated. Under the NaCl-N-48h condition, 16 genes showed upregulation and 15 genes were downregulated. Expression levels under NaCl-N-72h showed that 8 genes were upregulated and remaining 23 were downregulated (Fig 11B). For ZIK genes, 10 out of 11 genes had expression levels while 1 gene (TEA013344.1) had no effect under the given conditions. Expression levels under NaCl-N-24h condition, only 2 genes showed upregulation and the rest of the genes were downregulated. For NaCl-N-48h condition, only 1 gene was upregulated while the remaining 9 were downregulated. NaCl-N-72h condition too revealed a similar result with 2 genes being upregulated and remaining 8 being downregulated (Fig 11C). Heat maps were generated for the above-mentioned data as well (S12 Fig).

Finally, the expression levels of the tea MAPKKKs under MeJA treatment were studied and analysed. The hormonal treatment data is recorded based on the results of exposing the plant parts to aqueous solution of MeJA, under 4 stages: 1. 0h: 2. 12h: 3. 24h and 4. 48h where, 0h was used as the control. For the MEKK genes, under the 12h_MeJA condition, 3 genes showed upregulation, 13 genes were downregulated and remaining 1 gene had no expression at all. Under the 24h_MeJA condition, 4 genes were upregulated, 12 were downregulated and 1 gene was not expressed at all. Under 48h_MeJA condition, 8 genes were upregulated and 9 genes were downregulated (Fig 12A). Similarly, for the Raf genes, treatment under 12h_MeJA condition revealed that 10 out of 31 genes were upregulated and remaining 21 genes were downregulated. Under 24h_MeJA condition, 8 genes showed upregulation while 23 genes were downregulated. 48h_MeJA revealed that only 4 genes were upregulated and

rest of the genes were downregulated (Fig 12B). ZIK genes under the 12h_MeJA condition revealed that 4 genes were upregulated and 7 genes were downregulated. 24h_MeJA condition showed 5 genes being upregulated and remaining 6 being downregulated. 48h_MeJA condition suggested that 3 genes were upregulated and remaining 8 being downregulated (Fig 12C). Heat maps for these data were also generated (S13 Fig). A similar approach was taken for highlighting biotic stress responsive and defensive role of chitinase genes in tea [50].

Fig 9. Gene expression patterns of (A) MEKK (B) Raf and (C) ZIK genes, under cold stress conditions in *C. sinensis*. The relative expression of these genes were analysed in different tissues by using GraphPad Prism 8 software. The level of expression was in transcript per million (TPM).

Fig 10. Gene expression patterns of (A) MEKK (B) Raf and (C) ZIK genes, under drought stress conditions in *C. sinensis*. The relative expression of these genes were analysed in different tissues by using GraphPad Prism 8 software. The level of expression was in transcript per million (TPM).

Fig 11. Gene expression patterns of (A) MEKK (B) Raf and (C) ZIK genes, under salt stress conditions in *C. sinensis*. The relative expression of these genes were analysed in different tissues by using GraphPad Prism 8 software. The level of expression was in transcript per million (TPM).

Fig 12. Gene expression patterns of (A) MEKK (B) Raf and (C) ZIK genes, under Methyl jasmonate (MeJA) treatment in *C. sinensis*. The relative expression of these genes were analysed in different tissues by using GraphPad Prism 8 software. The level of expression was in transcript per million (TPM).

Discussion

The MAPKKK-MAPKK-MAPK signalling cascade plays an important role in plant development as well as in response to various environmental stresses [5, 34, 51]. Investigation of the MAPKKK genes, which form a significant component of this core regulatory network would certainly aid to a better understanding of the signalling genes. Although much progress has been made in identifying the functions of MAPKKK genes in

many organisms, these genes are yet to be analysed in *C. sinensis*. The objective of this study was to provide a comprehensive synopsis of the phylogenetic relationship, intron-exon architecture, motifs, functional domains, cis regulatory elements, genomic distribution and expression patterns of the MAPKKK genes in tea. Herein, a grand total of 59 MAPKKK proteins were screened and identified from tea plant genome. Previous studies in Arabidopsis [33], cucumber [10] and rice [6] have showed that the genes of MAPKKK family are classified into 3 subfamilies namely MEKK, Raf and ZIK [33, 10, 6]. Phylogenetic analysis (Fig 1) in tea showed similar results which indicate that genes in tea are also classified into these subfamilies. All the identified tea MAPKKKs had their respective subfamily specific domains. Motif analyses revealed that all MAPKKK proteins had protein kinase domains and proteins belonging to the same subfamily shared similar motifs (Fig 3). This result is consistent to previous studies conducted on other plants like cucumber [10], Arabidopsis [33] and banana [52]. The study of intron-exon architecture in tea MAPKKK genes revealed a significant variation in the number of introns and exons (Fig 4). The average number of exons in MEKK genes ranged between 6 to16. Highest number of exons found among the MEKK genes was 18. Raf genes had an average of 6 to 18 exons, with the highest being a staggering 28 found in TEA016969.1 and ZIK genes had exons between 7 to 10. Raf subfamily thereby featured more number of introns than MEKK and ZIK subfamilies. Reports suggest that the rate at which introns are lost is faster compared to the rate at which introns are gained after segmental duplication [53]. This is a conclusive evidence to infer that Raf subfamily might contain the original set of genes, from which genes of other subfamilies have been derived. The analysis also proposed that genes belonging to the same subgroup featured similar intron-exon organization. The MAPKKK genes also displayed a significant variation with respect to the UTR segments. Most genes possessed both 5’ and 3’ UTRs while few had only the 5’ UTR or 3’ UTR segment. These variations of the gene structures suggest that the tea

genome has been variable during its evolutionary history. Similar occurrence was also observed in plants like cassava [54], grapevine [55] and maize [39]. The interactions between the transcription factors and the promoter binding sites have crucial roles in regulation of gene expression at the transcriptional level [43]. The promoter sequence analysis for all the tea MAPKKKs revealed the diverse variety of cis-acting regulatory elements and their respective biological functions (Fig 5). Further in the study, all the identified genes were mapped onto their respective scaffolds (Fig 6). Duplication events observed among the ZIK genes shows the evidence that these genes play a crucial role in *C. sinensis*. The ratio of non-synonymous substitution rates (K_a) and synonymous substitution rates (K_s) was evaluated which indicated strong positive selection pressures to have occurred, enabling different factors to regulate the MAPKKKs in *C. sinensis* genome.

Earlier, comprehensive study in other plants has shown that MAPK cascade genes are extensively involved in controlling a number of biological processes which include cell growth, proliferation and response to various biotic and abiotic stresses such as salt stress, cold stress and drought stress [56, 57, 58, 59]. Tea plant is a woody perennial tree and has a life span of more than 100 years. [60]. However, traditional breeding techniques for tea are slow and limited primarily to selection which leads to narrowing down of its genetic base. Plants develop numerous signalling pathways to convert external stimuli into intracellular reactions in order to defend themselves against various environmental stress factors [61, 62]. MAPKKKs function at the highest level of the MAPK signalling cascade, helping with development and stress tolerance in plants.

A receptor mediated activation of MAPKKK proteins receive upstream signals to activate MAPKK proteins by phosphorylating the serine/threonine residues of the conserved motif (Ser/Thr-X3−5-Ser/Thr) [63, 64], which further phosphorylate specific MAPKs [65]. The activated MAPKK proteins further activates downstream MAPK proteins in the T-X-Y motif

[63, 64]. The phosphorylated MAPK proteins then activate multiple downstream target proteins, including transcription factors, protein kinases, and cytoskeletal components [63, 64].

MAPKKKs have been extensively studied in Arabidopsis and have been characterised. Previous literatures have conveyed that MEKK1-MKK1-MPK4 cascade is activated following a wounding stress response [66]. The MEKK1-MKK2-MPK4/MPK6 cascade is stimulated in salt and cold stress conditions [67]. Biochemical and genetic research suggests that MEKK1 is critically significant in response to cold stress and salt stress in Arabidopsis [68]. MAPK proteins classified in the same clades have been reported to perform similar roles in different organisms [69, 70]. Expression data presented in this study revealed TEA005122.1 had the highest level of expression under salt stress and this gene belongs to clade A along with AtMEKK1. A similar clustering event with AtMEKK1 was observed for TEA006319.1 which displayed the highest levels of expression under cold stress. Hence, TEA005122.1 and TEA006319.1 might get activated in response to cold and salt stress and initiate MEKK1 signalling cascade in tea. MKK3 encodes for a Mitogen Activated Protein Kinase Kinase, that stimulates MPK8, and is a target of MPKKK20, regulating ROS accumulation. MKK3-MAPKKK17-MAPKKK18 form an element of the ABA signalling pathway. MAPKKK17 and MAPKKK18 belong to Ser/Thr protein kinase family and help in the ABA-dependent activation of the MKK3-MPK7 pathway [71]. Previous study has shown that abcissic acid mediates drought stress response [72]. In our study, TEA005122.1 belonging to clade A is homologous to AtMEKK18 and shows the highest level of expression under drought stress which may be suggestive of the fact that TEA005122.1 might be responsible for drought stress responsive pathway in tea.

Analysis of gene expression in different plant parts under various environmental stress stimuli is key to understand the functions of the genes. Among the MEKK genes,

TEA016319.1 was expressed consistently in all the 8 plant tissues (Fig 8A). While for the Raf and ZIK genes, TEA000933.1 and TEA002087.1 were the consistently expressed genes (Fig 8B and Fig 8C). Reactive oxygen species (ROS) are oxygen derivatives, which are highly reactive by-products of the aerobic metabolism [73]. Plants consist of a complicated network of ROS scavenging antioxidant enzymes that helps to regulate the ROS levels under normal physiological conditions [73, 74]. Although a change from normal physiological conditions to adverse conditions shifts the equilibrium, resulting in increased ROS production. This might lead to serious oxidative damage and cell death because ROS are highly toxic to the cellular machinery [74]. Studies have suggested that the MAPK signalling cascade comprising of the MAPKKK-MAPKK-MAPK module is stimulated when excess ROS levels are detected under different stress conditions such as salt stress, cold stress and drought stress [73, 74]. It has been revealed that MPKKK1 activates two of its highly homologous MAPKKs (MKK1 and MKK2), which operate upstream of both MPK4 and MPK6 [67, 74]. Expression data for treatment under Methyl jasmonate (MeJA) revealed that TEA028214.1 among the MEKK genes, TEA000933.1 among the Raf genes and TEA002087.1 among the ZIK genes were expressed the most under the 72_MeJA condition (Fig 12). Collectively, these findings suggest the involvement of a number of MAPKKK genes, being upregulated and expressed under the stress conditions. In general, this study provides a detailed and comprehensive analysis of the MAPKKK genes in tea. Further extensive studies needs to be conducted on MAPKKK genes of tea that could provide a better understanding on the various functions of these set of genes in developmental processes and expression under various abiotic stress stimuli.

Conclusion

Mitogen activated protein kinases (MAPK) signalling cascade plays significant roles in different biological processes. The signalling components are linked to the upstream and

downstream regulators by phosphorylation. There has been substantial development in identifying the different MAPKKK genes and understand their physiological roles in various plants. However, these genes had not been yet explored and studied in tea plant. *In-silico* genome wide analysis had identified 59 MAPKKK genes from *C. sinensis* genome. The classification of the identified MAPKKK genes in 3 subfamilies were conducted based on their specific domain signatures. The genes were further investigated under phylogenetic analysis, conserved protein motifs, intron-exon architecture and analysis of cis regulatory elements. The 59 genes were mapped onto their respective genomic scaffolds and a network of functionally interacting genes was constructed. Further, expression profile analyses were conducted to reveal the involvement of the tea MAPKKK genes in various tissues during development and understand the expression pattern of these genes under various abiotic stress stimuli and plant hormonal treatment. These data will provide detailed information about the tea MAPKKK genes for further characterization of the MAPK signalling cascade and lay a concrete foothold for further exploration and research on *C. sinensis*.

Acknowledgements

This project was supported by the Key Technologies R & D Program for Crop Breeding of Zhejiang Province (2016C02054-19,2017C02010), the Natural Science Foundation of China (31670303), and the Joint Laboratory of Olive Oil Quality and Nutrition among China, Australia and Spain. The authors are thankful to DBT-eLibrary Consortium (DeLCON) for providing access to e-resources.

Author contributions: A.P., A.P.S. and S.S., designed and performed experiments, A.P.S., N.M., and G.S., devised the experiments, helped in data analysis and writing the manuscript.

Author details: Abhirup Paul: Department of Biochemistry, REVA University, Bangalore, Karnataka, India (Email: abhirupm16@gmail.com); Anurag P. Srivastava: Department of Life Sciences, Garden City University, Bangalore, Karnataka, India (Email: anurag.srivastava@gardencity.university, anuiitkgp@gmail.com); Shreya Subrahmanya: Department of Botany, St. Joseph's college autonomous, Bengaluru, Karnataka, India (Email: shreyasub916@gmail.com); Guoxin Shen: Sericultural Research Institute, Zhejiang Academy of Agricultural Sciences, Hangzhou 310021, China (Email: guoxin.shen@ttu.edu); Neelam Mishra: Department of Botany, St. Joseph's college autonomous, Bengaluru, Karnataka, India (Email: neelamiitkgp@gmail.com, neelammishra@sjc.ac.in).

Funding: Not Applicable

Data availability: All data generated or analysed during this study are included in this article and are provided in the Electronic Supplemental Materials (Supplemental_information 1 and Supplemental_information 2)

Compliance with ethical standards

Competing Financial Interests: There are no competing financial interests

Ethics approval: Not applicable

References

1. Champion, A., Picaud, A. & Henry, Y. Reassessing the MAP3K and MAP4K relationships. *Trends Plant Sci.* (2004). **9**, 123–129
2. Rodriguez, M. C., Petersen, M. & Mundy, J. Mitogen-activated protein kinase signaling in plants. *Annu. Rev. Plant Biol.* (2010). **61**, 621–649
3. Doczi, R., Okresz, L., Romero, A. E., Paccanaro, A. & Bogre, L. Exploring the evolutionary path of plant MAPK networks. *Trends Plant Sci.* (2012). **17**, 518–525
4. Colcombet, J. & Hirt, H. Arabidopsis MAPKs: A complex signalling network involved in multiple biological processes. *Biochem. J.* (2008). **413**, 217–226
5. Jonak, C., Okresz, L., Bogre, L. & Hirt, H. Complexity, cross talk and integration of plant MAPKinase signalling. *Current opinion in plant biology*. (2002). **5**, 415–424
6. Rao, K.P., Richa, T., Kumar, K., Raghuram, B. & Sinha, A. K. In silico analysis reveals 75 members of mitogen-activated protein kinase kinase kinase gene family in rice. *DNA Research.* (2010). **17,** 139–153

7. Sinha, A. K, Jaggi, M., Raghuram, B. & Tuteja, N. Mitogen-activated protein kinase signaling in plants under abiotic stress. *Plant signaling & behavior.* (2011). **6**, 196–203
8. Hamel, L. P., Sheen, J. & Seguin, A. Ancient signals: comparative genomics of green plant CDPKs. *Trends in plant science.* (2014). **19,** 79–89
9. Wu, J., Wang, J., Pan, C., Guan, X., Wang, Y., Liu, S., He, Y., Chen, J., Chen, L. & Lu, G. Genome-wide identification of MAPKK and MAPKKK gene families in tomato and transcriptional profiling analysis during development and stress response. *PloS one.* (2014). **9**,e103032. https://doi.org/10.1371/journal.pone.0103032.
10. Wang, J., Pan, C., Wang, Y., Ye, L., Wu, J., Chen, L., Zou, T. & Lu, G. Genome-wide identification of MAPK, MAPKK, and MAPKKK gene families and transcriptional profiling analysis during development and stress response in cucumber. *BMC Genomics.* (2015). **16,** 386
11. Popescu, S. C., Popescu, G. V., Bachan, S., Zhang, Z., Gerstein, M., Snyder, M. & Dinesh Kumar, S. P. MAPK target networks in Arabidopsis thaliana revealed using functional protein microarrays. *Genes Dev.* (2009). **23,** 80-92
12. Sun M, Xu Y, Huang J, et al. Global Identification, Classification, and Expression Analysis of MAPKKK genes: Functional Characterization of MdRaf5 Reveals Evolution and Drought-Responsive Profile in Apple. Sci Rep. 2017. **7**(1), 13511.
13. Huang, X., Luo, T., Fu, X., Fan, Q. & Liu J. Cloning and molecular characterization of a dehydration/drought tolerance in transgenic tobacco. *J. Exp. Bot.* (2011). **62**, 5191–5206.
14. Frye, C. A., Tang, D. & Innes, R. W. Negative regulation of defense responses in plants by a conserved MAPKK kinase. *Proc. Natl. Acad. Sci. U.S.A.* (2001). **98,** 373–378
15. Xia, E. H., Li, F. D., Tong, W., Li, P. H., Wu, Q., Zhao, H. J., Ge, R. H., Li, R. P., Li, Y. Y., Zhang, Z. Z., Wei, C. L. & Wan, X. C. Tea Plant Information Archive (TPIA): A comprehensive genomics and bioinformatics platform for tea plant. *Plant Biotechnology Journal.* (2019). **17,** 1938–1953
16. Berardini, T. Z., Reiser, L., Li, D., Mezheritsky, Y., Muller, R., Strait, E. & Huala, E. The Arabidopsis Information Resource: Making and mining the "gold standard" annotated reference plant genome. *Genesis*. (2015). **53,** 474–485
17. Kawahara, Y., de la Bastide, M., Hamilton, J. P., Kanamori, H., McCombie, W. R., Ouyang, S., Schwartz, D. C., Tanaka, T., Wu, J., Zhou, S., Childs, K. L., Davidson, R. M., Lin, H., Quesada-Ocampo, L., Vaillancourt, B., Sakai, H., Lee, S. S., Kim, J., Numa, H., Itoh, T., Buell, C. R. & Matsumoto, T. Improvement of the Oryza sativa Nipponbare reference genome using next generation sequence and optical map data. *Rice.* (2013). **6,** 4
18. Fernandez-Pozo, N., Menda, N., Edwards, J. D., Saha, S., Tecle, I. Y., Strickler, S. R., Bombarely, A., Fisher-York, T., Pujar, A., Foerster, H., Yan, A. & Mueller, L. A. The Sol Genomics Network (SGN)--from genotype to phenotype to breeding. *Nucleic Acids Res.* (2015). **43,** D1036-D1041 doi:10.1093/nar/gku1195
19. Madeira, F., Park, Y. M., Lee, J., Buso, N., Gur, T., Madhusoodanan, N., Basutkar, P., Tivey, A. R. N., Potter, S. C., Finn, R. D. & Lopez, R. The EMBL-EBI search and sequence analysis tools APIs. *Nucleic Acids Res.* (2019). **47,** W636-W641 (2019). doi: 10.1093/nar/gkz268.
20. Letunic, I., Doerks, T. & Bork, P. SMART: recent updates, new developments and status in 2015. *Nucleic Acids Res.* (2015). **43,** (Database issue):D257-D260 doi:10.1093/nar/gku949.
21. Gasteiger, E., Hoogland, C., Gattiker, A., Duvaud, S., Wilkins, M. R., Appel, R. D. & Bairoch, A. Protein Identification and Analysis Tools on the ExPASy Server; (In) John M. Walker (ed): The Proteomics Protocols Handbook, Humana Press (2005). pp. 571-607.

22. Pierleoni, A., Martelli, P. L., Fariselli, P. & Casadio, R. BaCelLo: a balanced subcellular localization predictor, *Bioinformatics.* (2006). **22,** 408-416. https://doi.org/10.1093/bioinformatics/btl222.
23. Sonnhammer, E. L. L., von Heijne, G. & Krogh, A. A hidden Markov model for predicting transmembrane helices in protein sequences. In Proc. of Sixth Int. Conf. on Intelligent Systems for Molecular Biology, (1998). p 175-182 Ed J. Glasgow, T. Littlejohn, F. Major, R. Lathrop, D. Sankoff, and C. Sensen. Menlo Park, CA: AAAI Press,
24. Korber, B. HIV Signature and Sequence Variation Analysis. Computational Analysis of HIV Molecular Sequences, Chapter 4, pages 55-72. (2000). Allen G. Rodrigo and Gerald H. Learn, eds. Dordrecht, Netherlands: Kluwer Academic Publishers.
25. Kumar, S., Stecher, G. & Tamura, K. MEGA7: Molecular Evolutionary Genetics Analysis Version 7.0 for Bigger Datasets. *Mol Biol Evol.* (2016). **33,** 1870-1874. doi:10.1093/molbev/msw054.
26. Hu, B., Jin, J., Guo, A. Y., Zhang, H., Luo, H. & Gao, G. GSDS 2.0: an upgraded gene feature visualization server. *Bioinformatics.* (2015). 31, 1296-1297
27. Bailey, T. L., Boden, M., Buske, F.A., Frith, M., Grant, C. E, Clementi, L., Ren, J., Li, W. W. & Noble, W. S. MEME SUITE: tools for motif discovery and searching. *Nucleic Acids Res.* (2009). **37,** W202-208. doi: 10.1093/nar/gkp335.
28. Rombauts S, Déhais P, Van Montagu M, Rouzé P. PlantCARE, a plant cis-acting regulatory element database. *Nucleic Acids Res.* (1999). **27**(1):295-6. doi: 10.1093/nar/27.1.295. PMID: 9847207; PMCID: PMC148162. 2.
29. Lescot M, Déhais P, Thijs G, Marchal K, Moreau Y, Van de Peer Y, Rouzé P, Rombauts S. PlantCARE, a database of plant cis-acting regulatory elements and a portal to tools for in silico analysis of promoter sequences. *Nucleic Acids Res*. (2002). **30,** 325-7. doi: 10.1093/nar/30.1.325. PMID: 11752327; PMCID: PMC99092.
30. Jiangtao, C., Yingzhen, K., Qian, W., Yuhe, S., Daping, G, Jing, L.V. & Guanshan, L. Mapgene2chrom, a tool to draw gene physical map based on perl and svg languages. *Hereditas.* (2015). **37,** 91-97.
31. Szklarczyk, D., Gable, A. L., Lyon, D., Junge, A., Wyder, S., Cepas, J. H., Simonovic, M., Doncheva, N. T., Morris, J. H. , Bork, P., Jensen, L. J. & von Mering, C. STRING v11: protein-protein association networks with increased coverage, supporting functional discovery in genome wide experimental datasets. *Nucleic Acids Res.* (2019). **47**, (D1):D607-D613.
32. Gao, M., Liu, J., Bi, D., Zhang, Z., Cheng, F., Chen, S. & Zhang, Y. MEKK1, MKK1/MKK2 and MPK4 function together in a mitogen-activated protein kinase cascade to regulate innate immunity in plants. *Cell Res.* (2008). **18,** 1190–1198 (2008).
33. Kong, Q., Qu, N., Gao, M., Zhang, Z., Ding, X., Yang, F., Li, Y., Dong, O. X, Chen, S., Li, X. & Zhang, Y. The MEKK1-MKK1/ MKK2-MPK4 kinase cascade negatively regulates immunity mediated by a mitogen-activated protein kinase kinase kinase in Arabidopsis. *Plant Cell.* (2012). **24,** 2225–2236
34. Pitzschke, A., Djamei, A., Bitton, F. & Hirt, H. A major role of the MEKK1- MKK1/2-MPK4 pathway in ROS signalling. *Mol Plant.* (2009). **2,** 120–137
35. Murakami-Kojima, M., Nakamichi, N., Yamashino, T. & Mizuno, T. The APRR3 component of the clock-associated APRR1/TOC1 quintet is phosphorylated by a novel protein kinase belonging to the WNK family, the gene for which is also transcribed rhythmically in Arabidopsis thaliana. *Plant Cell Physiol.* (2002). **43,** 675–683
36. Wang, Y., Liu, K., Liao, H., Zhuang, C., Ma, H. & Yan, X. The plant WNK gene family and regulation of flowering time in Arabidopsis. *Plant Biol.* (2008). **10,** 548– 562

37. Goyal, R. K., Tulpan, D., Chomistek, N., González-Peña Fundora, D., West, C., Ellis, B. E., Frick, M., Laroche, A. & Foroud, N. A. Analysis of MAPK and MAPKK gene families in wheat and related Triticeae species. *BMC Genomics*. (2018). **19,** 178
38. Taft, R. J., Pheasant, M. & Mattick, J. S. The relationship between non-proteincoding DNA and eukaryotic complexity. *BioEssays.* (2007). **29,** 288–299
39. Liu, Z., Shi, L., Liu, Y., Tang, Q., Shen, L., Yang, S., Cai, J., Yu, H., Wang, R., Wen, J., Lin, Y., Hu, J., Liu, C., Zhang,Y., Mou, S. & He, S. Genome wide identification and transcriptional expression analysis of mitogen-activated protein kinase and mitogen-activated protein kinase kinase genes in *Capsicum annuum*. *Front. Plant Sci.* (2015). **6**, 780 doi: 10.3389/fpls.2015.00780.
40. Zhang, G., Li, C., Li, Q., Li, B., Larkin, D. M., Lee, C., Storz, J. F., Antunes, A., Greenwold, M. J., Meredith, R.W. et al. Comparative genomics reveals insights into avian genome evolution and adaptation. *Science.* (2014). **346,** 1311–20
41. Fedorova, L. & Fedorov, A. Introns in gene evolution. In: Long M, editor. Origin and evolution of new gene functions. Dordrecht: Springer Netherlands, (2003). pp. 123–131
42. Deutsch, M. & Long, M. Intron-exon structures of eukaryotic model organisms. *Nucleic Acids Res.* (1999). **27,** 3219-3228
43. Wu, A., Hao, P., Wei, H., Sun, H., Cheng, S., Chen, P., Ma, Q., Gu, L., Zhang, M., Wang, H. and Yu, S. Genome-Wide Identification and Characterization of Glycosyltransferase Family 47 in Cotton. *Front. Genet.* (2019).10:824. doi: 10.3389/fgene.2019.00824.
44. Liu, Z., An, C., Zhao, Y., Xiao, Y., Bao, L., Gong, C., & Gao, Y. Genome-Wide Identification and Characterization of the CsFHY3/FAR1 Gene Family and Expression Analysis under Biotic and Abiotic Stresses in Tea Plants (Camellia sinensis). *Plants* (2021). **10,** 570. https://doi.org/ 10.3390/plants1003057
45. Liu, W., Li, W., He, Q., Daud, M. K., Chen, J. & Zhu, S. Genome-wide survey and expression analysis of Calcium-Dependent Protein Kinase in *Gossypium raimondii*. *PLoS One.* (2014). **9,** e98189
46. Pearson, W. R. An introduction to sequence similarity ("homology") searching. *Current protocols in bioinformatics.* (2013). doi:10.1002/0471250953.bi0301s42
47. Gan, H. H., Perlow, R. A., Roy, S., Ko, J., Wu, M., Huang, J., Yan, S., Nicoletta, A., Vafai, J., Sun, D., Wang, L., Noah, J. E., Pasquali, S. & Schlick, T. Analysis of protein sequence/structure similarity relationship. *Biophysical Journal.* (2002). **83,** 2781-2791
48. Wang, X. C., Zhao, Q. Y., Ma, C. L., Zhang, Z. H., Cao, H. L, Kong, Y. M., Yue, C., Hao, X. Y., Chen, L., Ma, J. Q., Jin, J. Q., Li, X. & Yang, Y. J. Global transcriptome profiles of Camellia sinensis during cold acclimation. *BMC Genomics.* (2013). **14,** 415
49. Zhang, Q., Cai, M., Yu, X., Wang, L., Guo, C., Ming, R. & Zhang, J. Transcriptome dynamics of Camellia sinensis in response to continuous salinity and drought stress. *Tree Genetics & Genomes.* (2017). **13,** 78
50. Bordoloi, K.S., Krishnatreya, D.B., Baruah, P.M. *et al.* Genome-wide identification and expression profiling of chitinase genes in tea (*Camellia sinensis* (L.) O. Kuntze) under biotic stress conditions. *Physiol Mol Biol Plants* (2021). **27,** 369–385.
51. Moustafa, K., AbuQamar, S., Jarrar, M., Al-Rajab, A. J. & Trémouillaux-Guiller, J. MAPK cascades and major abiotic stresses. *Plant Cell Rep.* (2014). **33,** 1217–1225
52. Wang, L., Hu, W., Tie, W., Ding, Z., Ding, X., Liu, Y., Yan, Y., Wu, C., Peng, M., Xu, B. & Jin, Z. The MAPKKK and MAPKK gene families in banana: identification, phylogeny and expression during development, ripening and abiotic stress. *Sci Rep.* (2017). **7,** 1159

53. Nuruzzaman, M., Manimekalai, R, Sharoni, A. M., Satoh, K., Kondoh, H., Ooka, H. & Kikuchi, S. Genome-wide analysis of NAC transcription factor family in rice. *Genes.* (2010). **465,** 30-44
54. Ye, J., Yang, H., Shi, H., Wei, Y., Tie, W., Ding, Z., Yan, Y., Luo, Y., Xia, Z., Wang, W., Peng, M., Li, K., Zhang, H. & Hu, W. The MAPKKK gene family in cassava: Genome-wide identification and expression analysis against drought stress. *Sci Rep.* (2017).**7,** 14939. https://doi.org/10.1038/s41598-017-13988-8.
55. Wang, G., Lovato, A., Polverari, A., Wang, M., Liang, Y. H., Ma, Y. C. & Cheng, Z. M. Genome-wide identifcation and analysis of mitogen activated protein kinase kinase kinase gene family in grapevine (Vitis vinifera). *BMC Plant Biol.* (2014). **14,** 219
56. Virk, N., Li, D., Tian, L., Huang, L., Hong, Y., Li, X., Zhang, Y., Liu, B., Zhang, H. & Song, F. Arabidopsis Raf-like mitogen-activated protein kinase kinase kinase gene Raf43 is required for tolerance to multiple abiotic stresses. *PLoS One.* (2015). **10,** e0133975.
57. Jia, H., Hao, L., Guo, X., Liu, S., Yan, Y. & Guo, X. A Raf-like MAPKKK gene, GhRaf19, negatively regulates tolerance to drought and salt and positively regulates resistance to cold stress by modulating reactive oxygen species in cotton. *Plant Sci.* (2016). **252,** 267–281
58. Shitamichi, N., Matsuoka, D., Sasayama, D., Furuya, T. & Nanmori, T. Over-expression of MAP3Kδ4, an ABA-inducible Raf-like MAP3K that confers salt tolerance in Arabidopsis. Plant *Biotechnol.* (2013). **30,** 111–118
59. Shou, H., Bordallo, P. & Wang, K. Expression of the Nicotiana protein kinase (NPK1) enhanced drought tolerance in transgenic maize. *J. Exp. Bot.* (2004). **55,** 1013–1019
60. Mukhopadhyay, M., Mondal, T.K. & Chand, P.K. Biotechnological advances in tea (*Camellia sinensis* [L.] O. Kuntze): a review. *Plant Cell Rep* (2016). **35,** 255–287.
61. Tuteja, N. Abscisic acid and abiotic stress signaling. *Plant Signal Behav.* (2007). **2,** 135–138
62. Ning, J., Li, X., Hicks, L. M. & Xiong, L. A Raf-like MAPKKK gene DSM1 mediates drought resistance through reactive oxygen species scavenging in rice. *Plant Physiol.* (2010). **152,** 876–890.
63. Mishra, N.S., Tuteja, R., and Tuteja, N. Signaling through MAP kinase networks in plants. *Arch. Biochem. Biophys*. (2006). **452**, 55–68. doi: 10.1016/j.abb.2006. 05.001.
64. Zhang, A., Jiang, M., Zhang, J., Tan, M., and Hu, X. Mitogen-activated protein kinase is involved in abscisic acid-induced antioxidant defense and acts downstream of reactive oxygen species production in leaves of maize plants. *Plant Physiol*. (2006). **141**, 475–487. doi: 10.1104/pp.105.075416
65. Chang, L. & Karin, M. Mammalian MAP kinase signaling cascades. *Nature.* (2001). **410**, 37-40 doi: 10.1038/35065000.
66. Hadiarto, T., Nanmori, T., Matsuoka, D., Iwasaki, T., Sato, K., Fukami, Y., Azuma, T. & Yasuda, T. Activation of Arabidopsis MAPK kinase kinase (AtMEKK1) and induction of AtMEKK1–AtMEK1 pathway by wounding. *Planta.* (2006). **223,** 708–713
67. Teige, M., Scheikl, E., Eulgem, T., Dóczi, R., Ichimura, K., Shinozaki, K., Dangl, J. L. & Hirt, H. The MKK2 pathway mediates cold and salt stress signaling in Arabidopsis. *Mol Cell.* (2004). **15,** 141–152
68. Nicole, M.C., Hamel, L.P., Morency, M.J., et al. MAP-ping genomic organization and organ-specific expression profiles of poplar MAP kinases and MAP kinase kinases. *BMC Genomics*. (2006). **7**, 223
69. Asai, T., Tena, G., Plotnikova, J., Willmann, M. R., Chiu, W. L., GomezGomez, L., Boller, T., Ausubel, F. M. & Sheen J. MAP kinase signalling cascade in Arabidopsis innate immunity. *Nature.* (2002). **415,** 977–983

70. Liu, Z., Zhang, L., Xue, C., Fang, H., Zhao, J., Liu, M. Genome-wide identification and analysis of MAPK and MAPKK gene family in Chinese jujube (Ziziphus jujuba mill.). *BMC Genomics.* (2017). **18,** 855
71. Chatterjee, A., Paul, A., Unnati, G.M. et al. MAPK cascade gene family in Camellia sinensis: In-silico identification, expression profiles and regulatory network analysis. *BMC Genomics* (2020). **21**, 613
72. Takahashi, Fuminori, et al. "Drought stress responses and resistance in plants: From cellular responses to long-distance intercellular communication." *Frontiers in Plant Science* (2020). **11**, 1407
73. Liu, Y. & He, C. A review of redox signaling and the control of MAP kinase pathway in plants. *Redox Biology.* (2016). **11,** 192–204
74. Pitzschke, A. & Hirt, H. Mitogen-Activated Protein Kinases and Reactive Oxygen Species Signaling in Plants. *Plant Physiology.* (2006). **141,** 351–356.

Supporting Information:

1. **S1 Table. BLAST positives table for MEKK genes of *C. sinensis***
2. **S2 Table. BLAST positives table for Raf genes of *C. sinensis***
3. **S3 Table. BLAST positives table for ZIK genes of C. sinensis**
4. **S4 Table. Function specific list of cis-acting elements identified from 2 kbp upstream region of all the identified MEKK, Raf and ZIK genes of *C. sinensis***
5. **S5 Table. Ka/Ks ratios of MAPKKKs of MEKK subfamily in *C. sinensis***
6. **S6 Table. Ka/Ks ratios of MAPKKKs of Raf subfamily in *C. sinensis***
7. **S7 Table. Ka/Ks ratios of MAPKKKs of ZIK subfamily in *C. sinensis***
8. **S8 Table. Tissue specific expression data of tea MAPKKKs**
9. **S9 Table. Expression data of tea MAPKKKs under cold stress**
10. **S10 Table. Expression data of tea MAPKKKs under drought stress**
11. **S11 Table. Expression data of tea MAPKKKs under salt stress**
12. **S12 Table. Expression data of tea MAPKKKs under MeJA treatment**
13. **S1 Fig. Transmembrane helices of MAPKKKs of MEKK subfamily in *C. sinensis*.** TMHMM Server, v.2.0 (http://www.cbs.dtu.dk/services/TMHMM/), was used to predict the presence of transmembrane helices.
14. **S2 Fig. Transmembrane helices of MAPKKKs of Raf subfamily in *C. sinensis*.** TMHMM Server, v.2.0 (http://www.cbs.dtu.dk/services/TMHMM/), was used to predict the presence of transmembrane helices.
15. **S3 Fig. Transmembrane helices of MAPKKKs of ZIK subfamily in *C. sinensis***. TMHMM Server, v.2.0 (http://www.cbs.dtu.dk/services/TMHMM/), was used to predict the presence of transmembrane helices.
16. **S4 Fig. Motif logos of the 10 identified motifs in 59 MAPKKKs of *C. sinensis***. The motif logos were generated by MEME suite.
17. **S5 Fig. The ds/dn cumulative graph of MAPKKKs of MEKK subfamily in *C. sinensis***. SNAP server (https://www.hiv.lanl.gov/content/sequence/SNAP/SNAP.html) has been used to generate the graph.

18. **S6 Fig. The ds/dn cumulative graph of MAPKKKs of Raf subfamily in *C. sinensis*.** SNAP server (https://www.hiv.lanl.gov/content/sequence/SNAP/SNAP.html) has been used to generate the graph.
19. **S7 Fig. The ds/dn cumulative graph of MAPKKKs of ZIK subfamily in *C. sinensis***. SNAP server (https://www.hiv.lanl.gov/content/sequence/SNAP/SNAP.html) has been used to generate the graph.
20. **S8 Fig. GO analysis of all the 59 MAPKKKs in *C. sinensis*.** The results have been grouped into three main categories: Biological Process, Cellular Component and Molecular function. The y-axis represents the frequency of genes while the x-axis represents the potential functions.
21. **S9 Fig. Heat maps for tissue-specific expression patterns of A) MEKK; B) Raf and; C) ZIK genes in *C. sinensis*.** The relative expression of these genes were analysed in different tissues, by using GraphPad Prism 8 software. The level of expression was in transcript per million (TPM). The 8 different tissues are represented on the right and the tea genes are marked below.
22. **S10 Fig. Heat maps for cold stress expression patterns of A) MEKK; B) Raf and; C) ZIK genes in *C. sinensis*.** The relative expression of these genes were analysed in different stages, by using GraphPad Prism 8 software. The level of expression was in transcript per million (TPM). The different stages are represented on the right and the tea genes are marked below.
23. **S11 Fig. Heat maps for drought stress expression patterns of A) MEKK; B) Raf and; C) ZIK genes in *C. sinensis***. The relative expression of these genes were analysed in different stages, by using GraphPad Prism 8 software. The level of expression was in transcript per million (TPM). The different stages are represented on the right and the tea genes are marked below.
24. **S12 Fig. Heat maps for salt stress expression patterns of (A) MEKK (B) Raf and (C) ZIK genes in *C. sinensis*.** The relative expression of these genes were analysed in different stages, by using GraphPad Prism 8 software. The level of expression was in transcript per million (TPM). The different stages are represented on the right and the tea genes are marked below.
25. **S13 Fig. Heat maps for MeJA treatment expression patterns of (A) MEKK (B) Raf and (C) ZIK genes in *C. sinensis***. The relative expression of these genes were analysed in different stages, by using GraphPad Prism 8 software. The level of expression was in transcript per million (TPM). The different stages are represented on the right and the tea genes are marked below.

Figure

Click here to access/download;Figure;Fig 1.docx

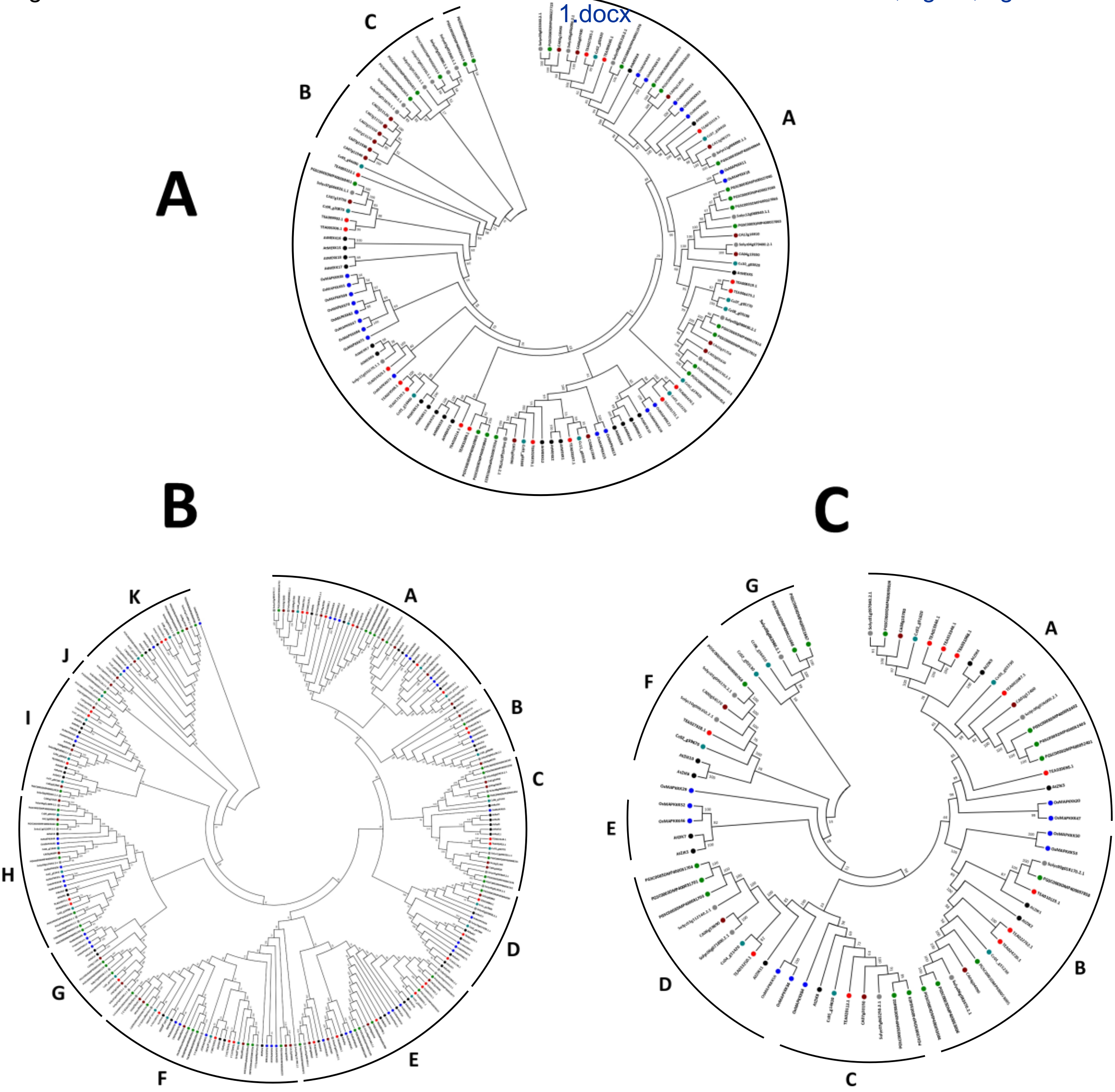

Figure

Click here to access/download;Figure;Fig 2.docx

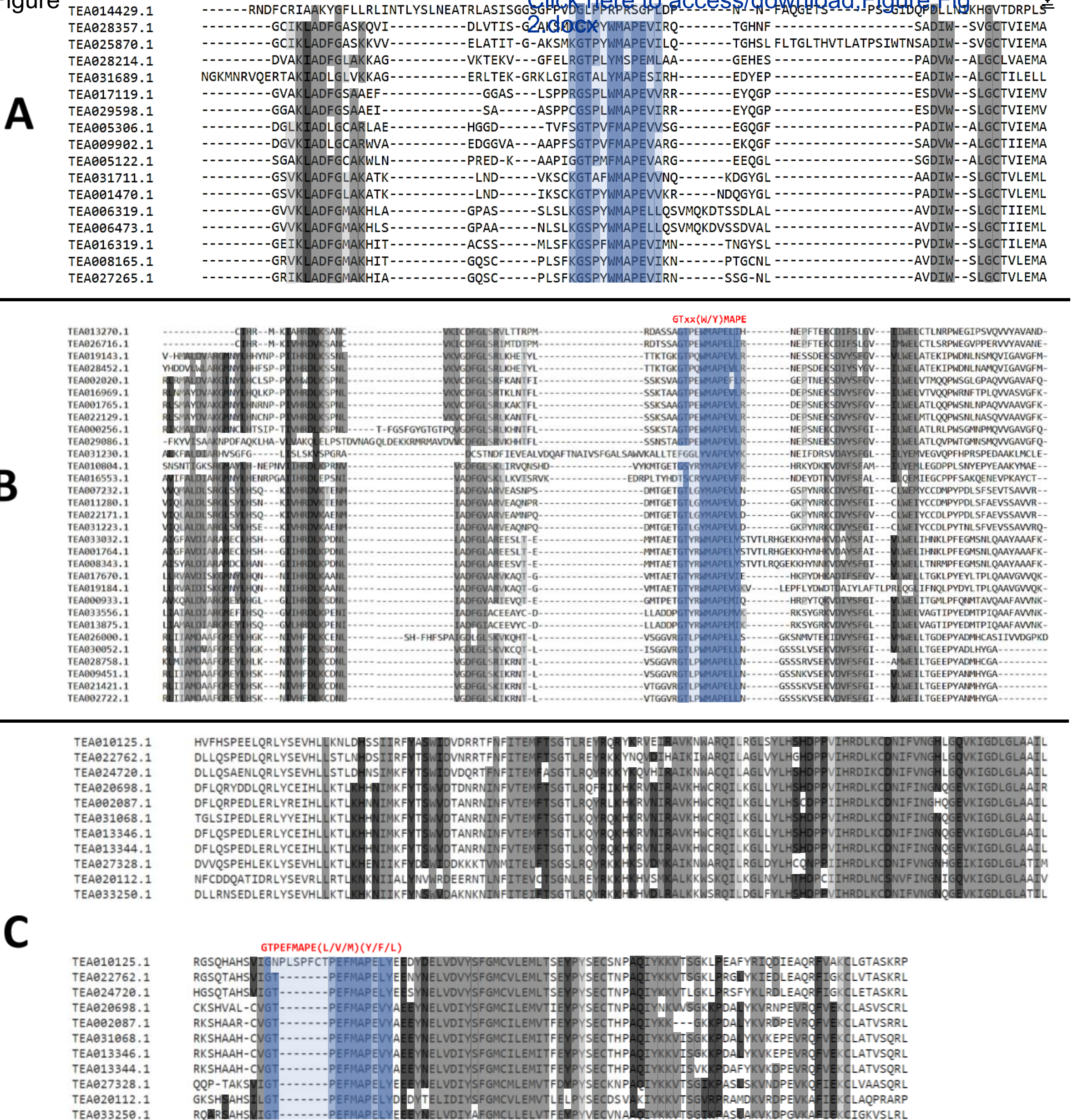

Figure

Click here to access/download;Figure;Fig 3.docx

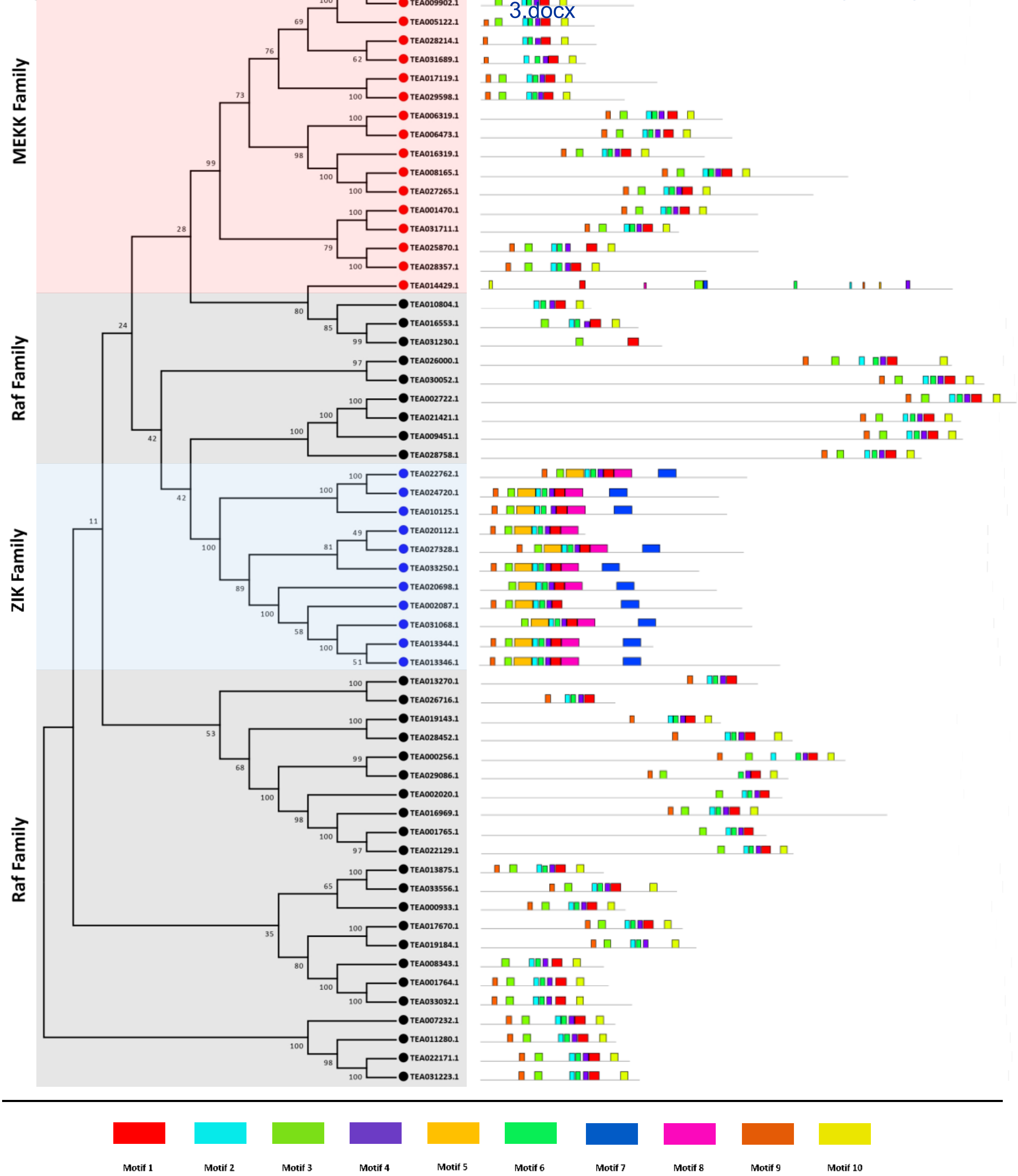

Figure
Click here to access/download;Figure;Fig 4.docx

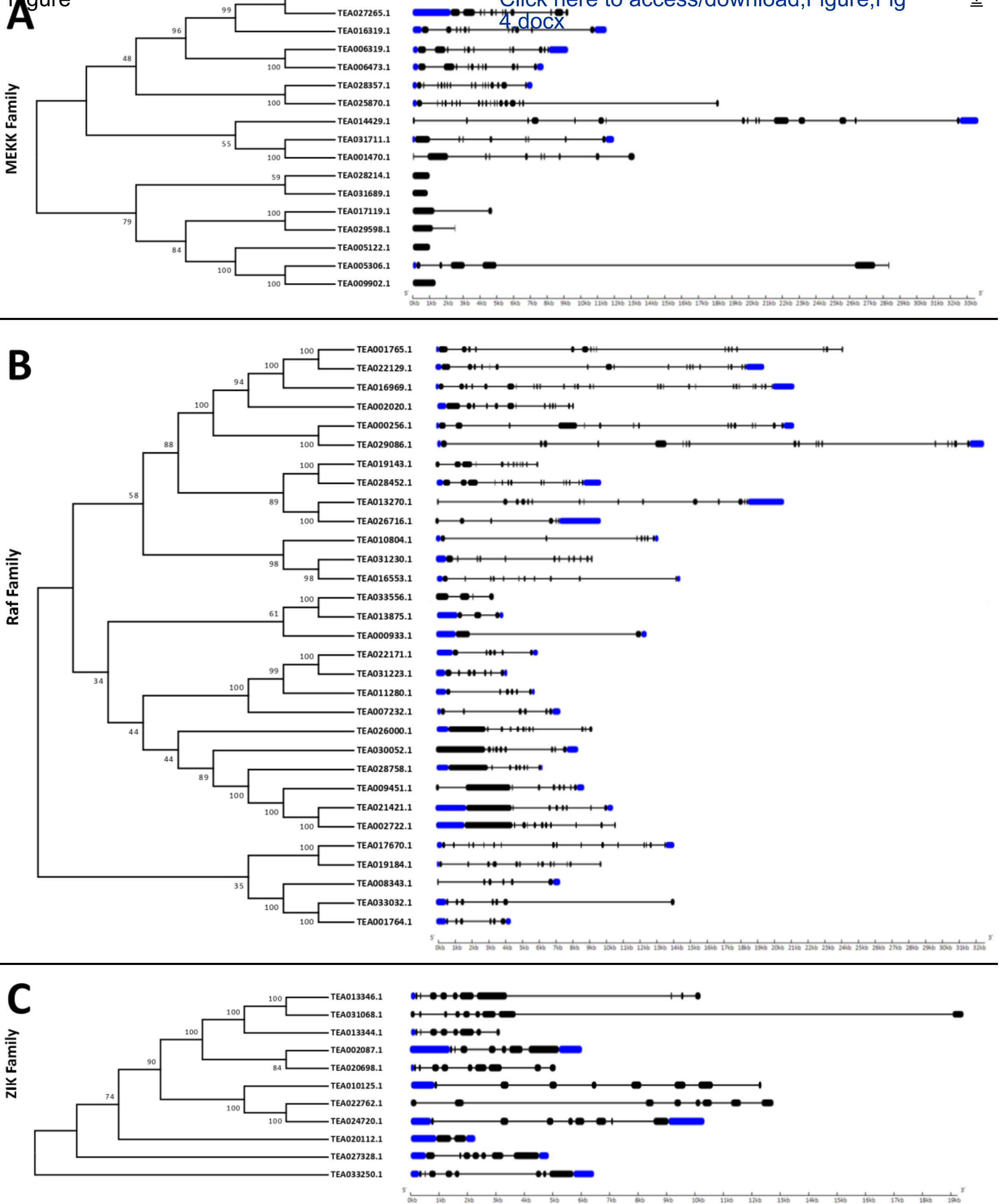

Figure

Click here to access/download;Figure;Fig 5.docx

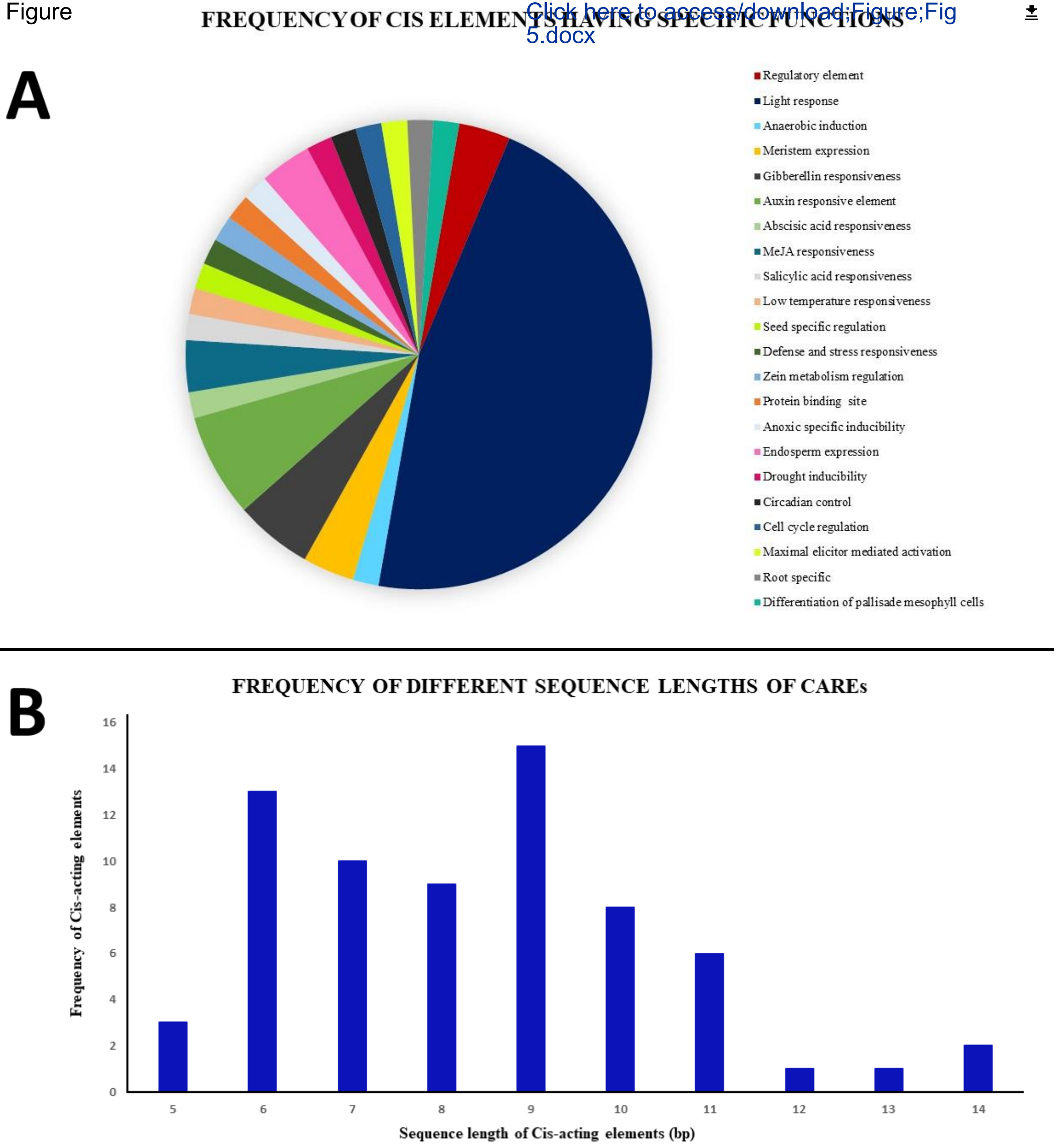

Figure

Click here to access/download;Figure;Fig 6.docx

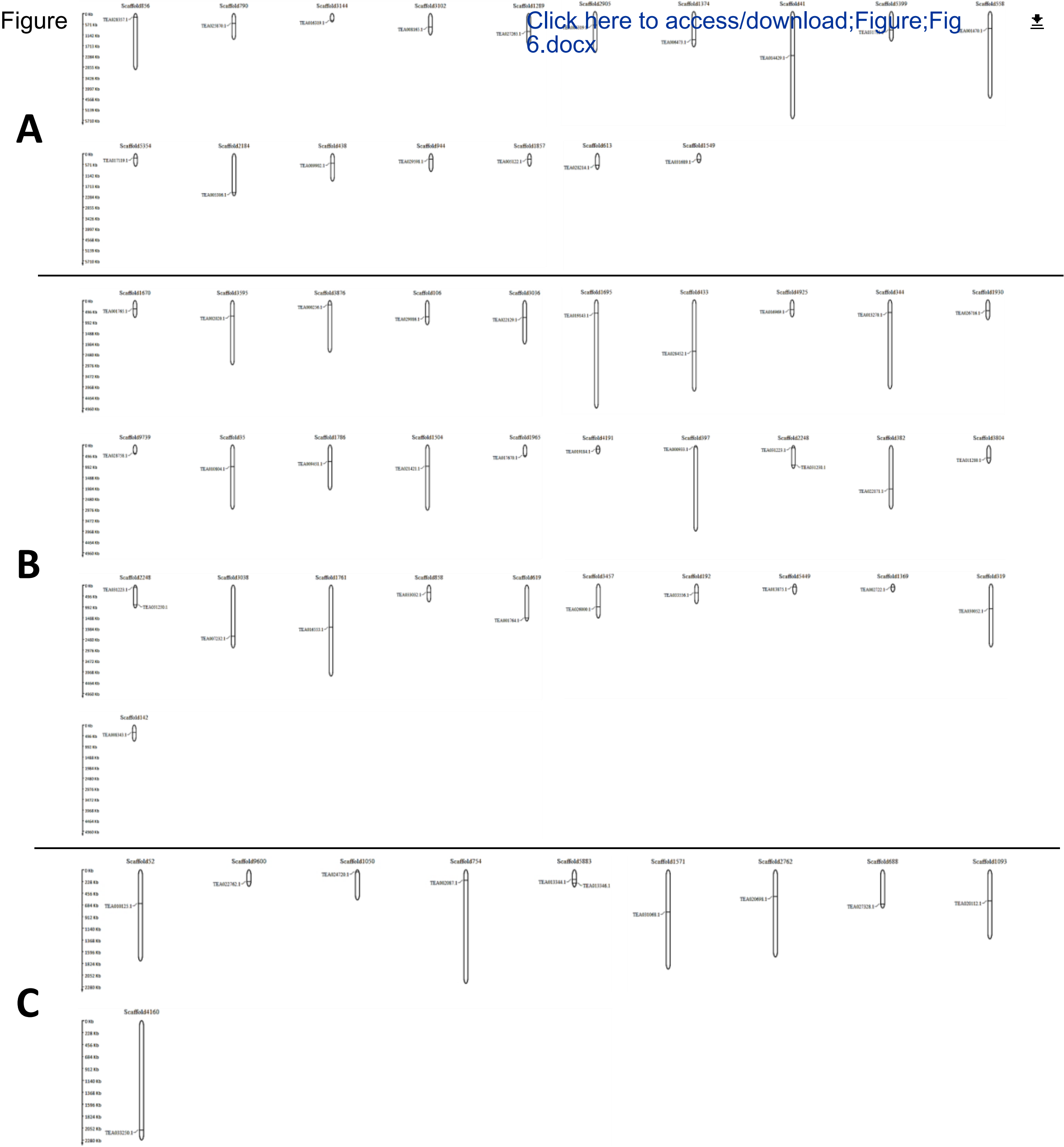

Figure

Click here to access/download;Figure;Fig 7.docx

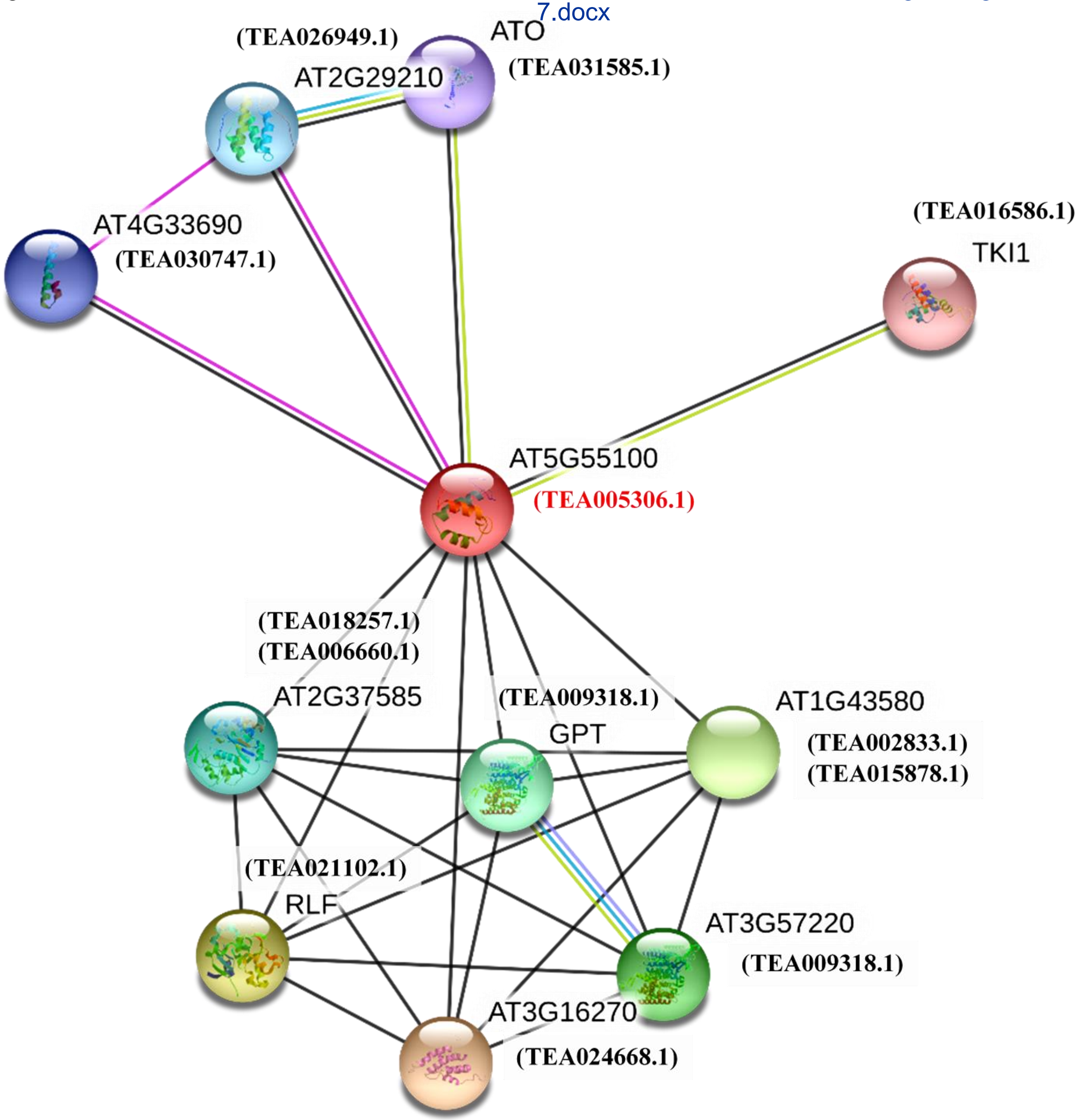

Figure
Click here to access/download;Figure;Fig 8.docx

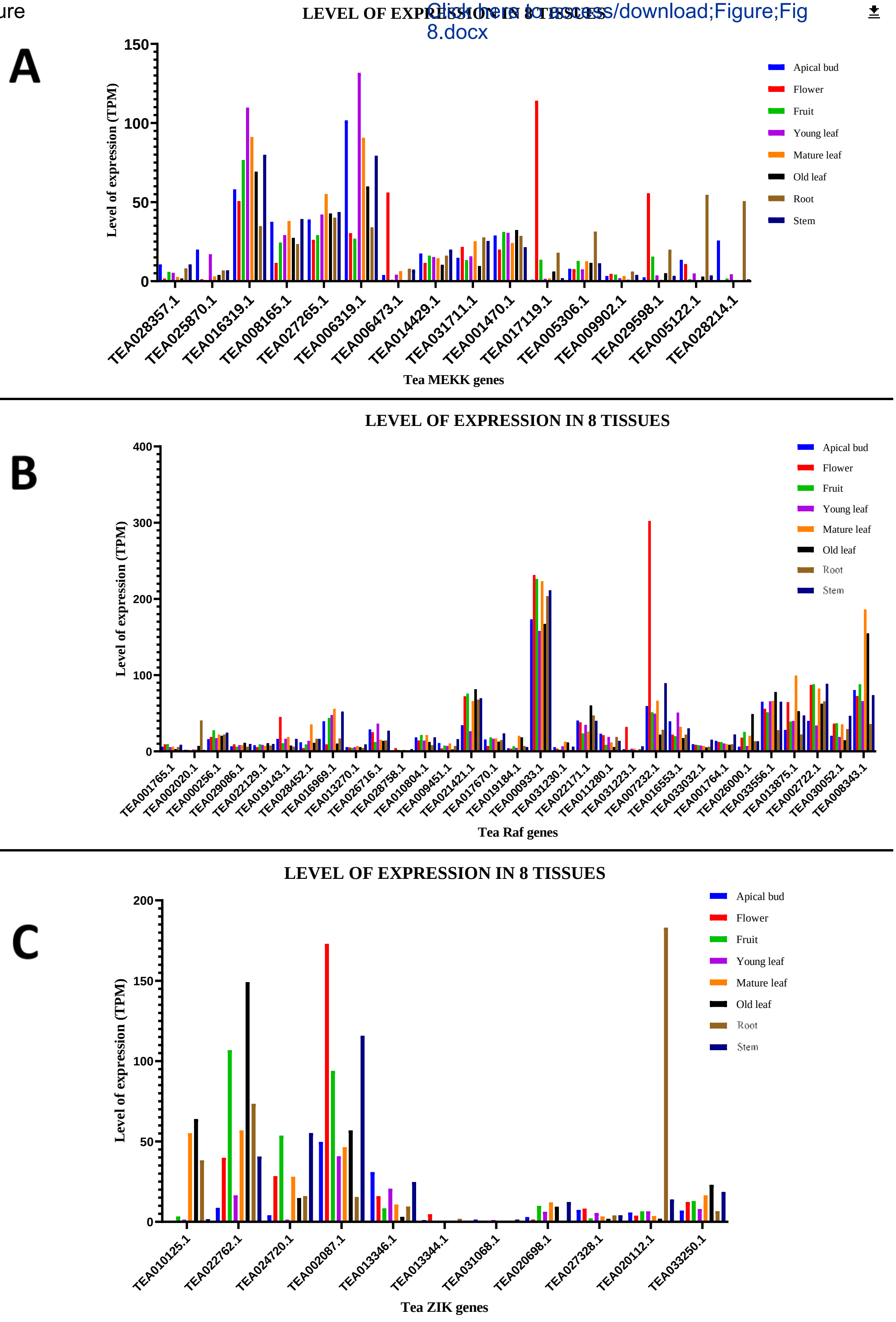

Figure

Click here to access/download;Figure;Fig 9.docx

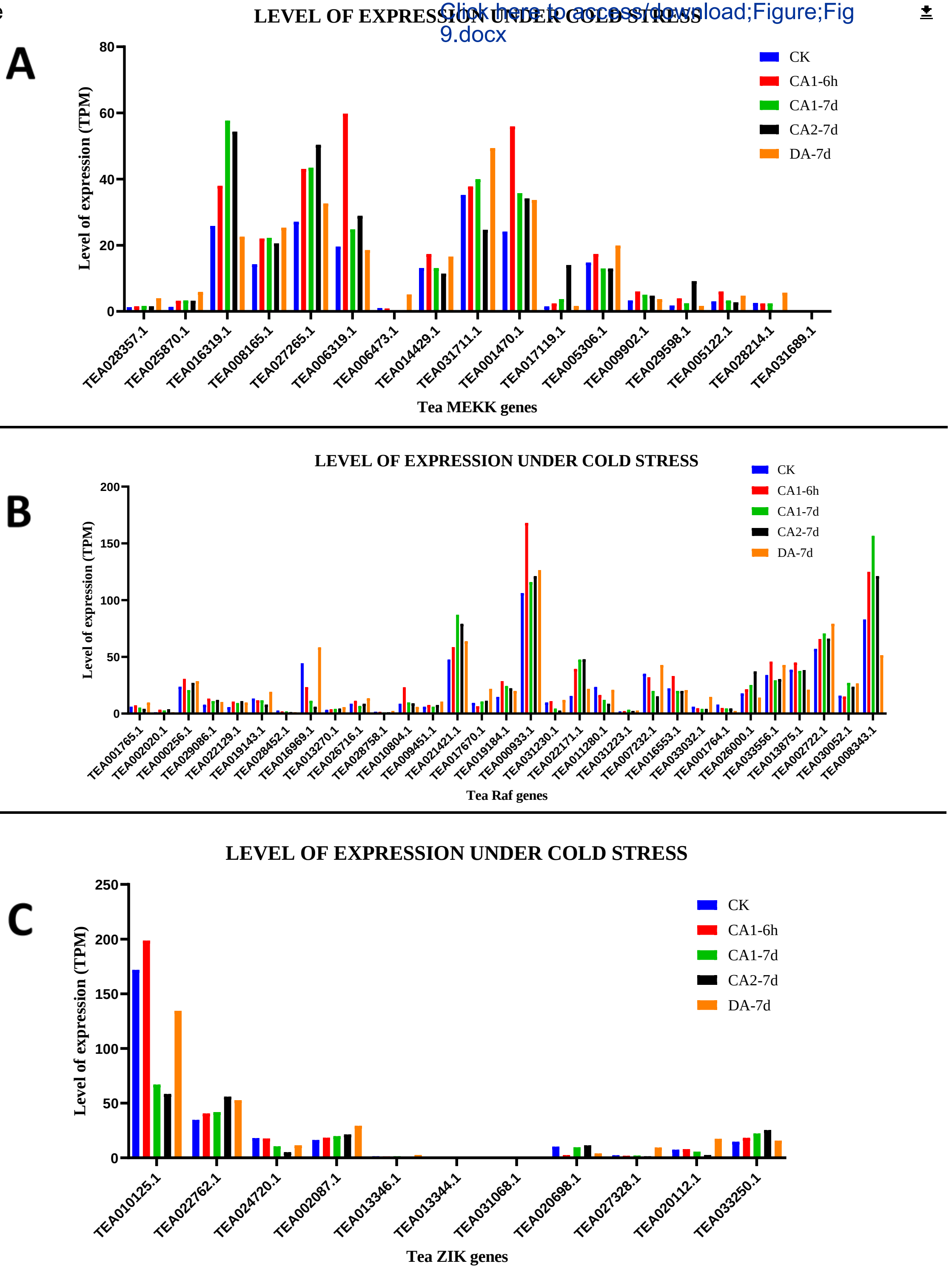

Figure

Click here to access/download;Figure;Fig 10.docx

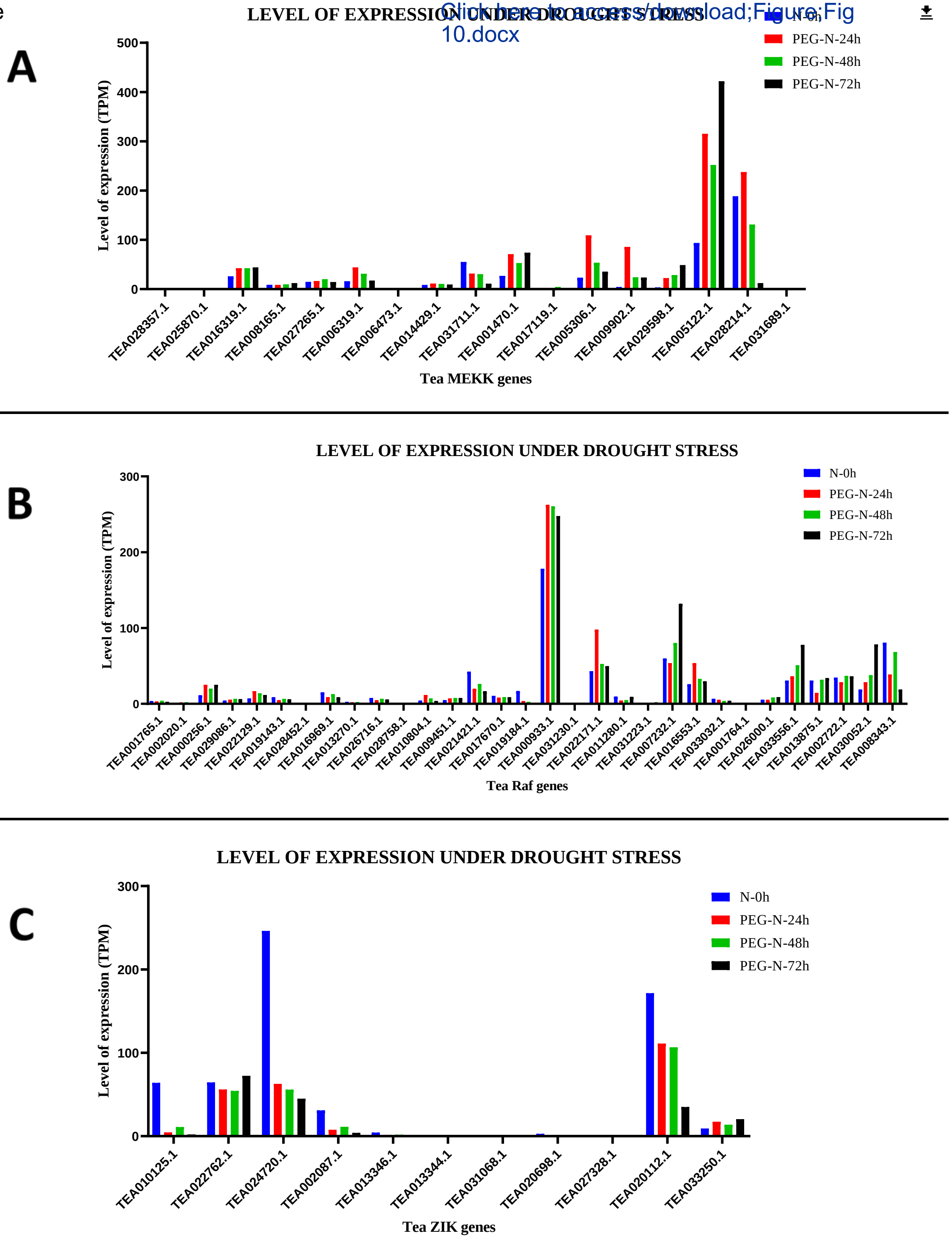

Figure

Click here to access/download;Figure;Fig 11.docx

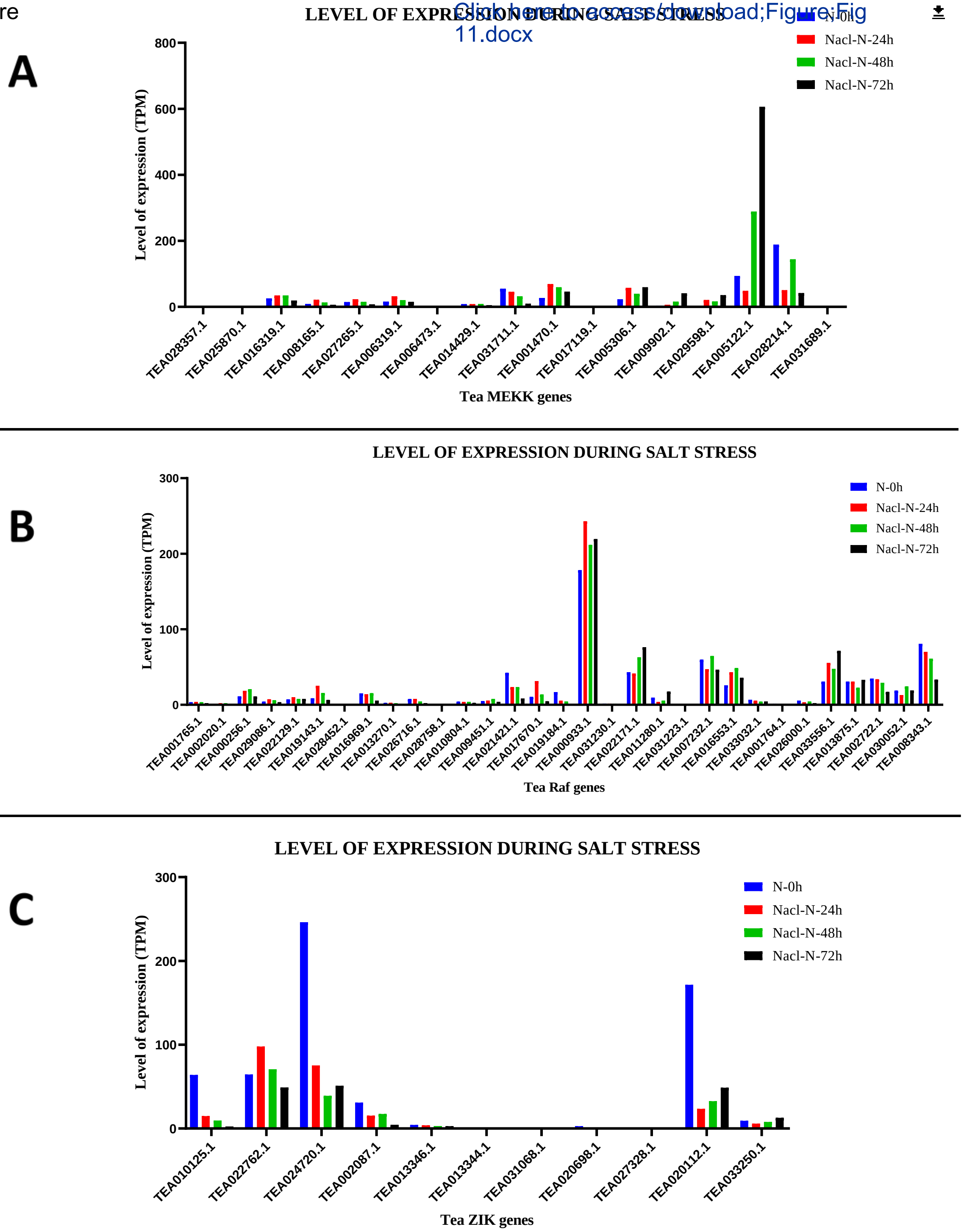

Figure

Click here to access/download;Figure;Fig 12.docx

A

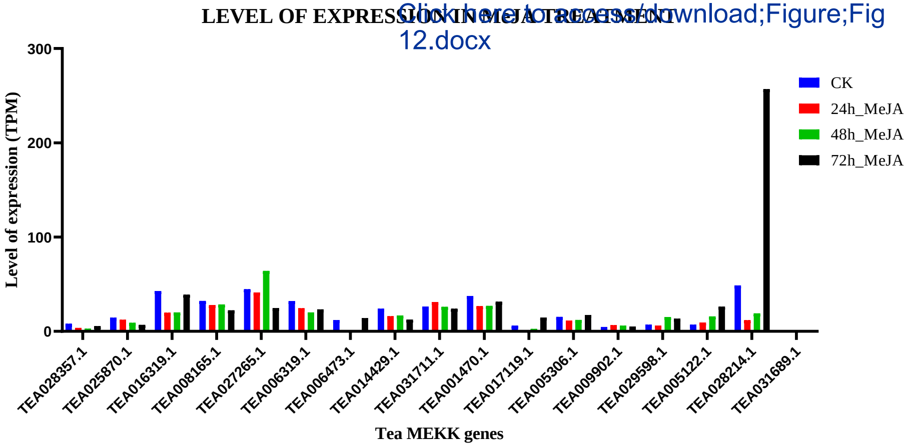

B

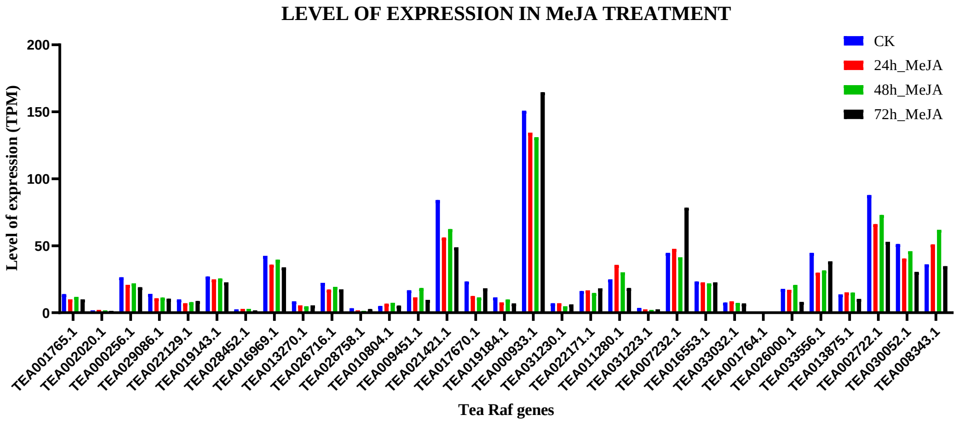

C

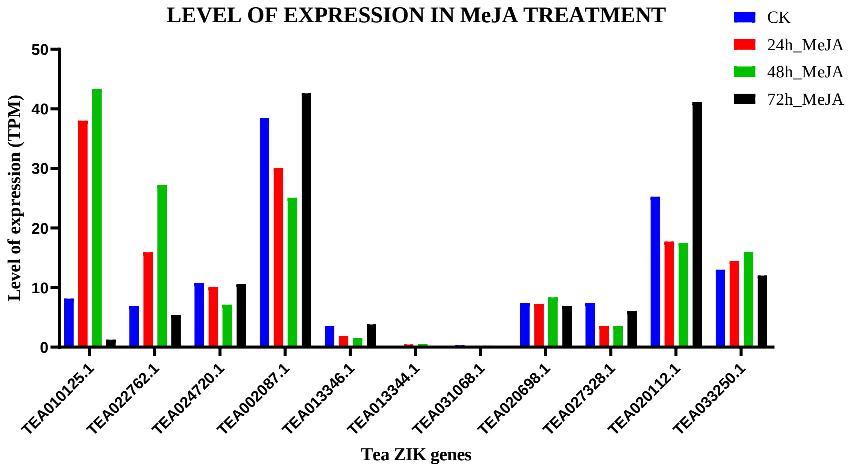

Click here to access/download
Supporting Information
Supporting information_1.pdf

Click here to access/download
Supporting Information
Supporting information_2.pdf

In-silico identification, expressional profile and regulatory network analysis of Mitogen Activated Protein Kinase Kinase Kinase gene family in *C. sinensis*

Abhirup Paul[1†], Anurag P. Srivastava[2†], Shreya Subrahmanya[3], Guoxin Shen[4†*], Neelam Mishra[3*]

[1]Department of Biochemistry

REVA University

Bangalore, Karnataka,

India

[2]Department of life Sciences

Garden City University

Bangalore, Karnataka,

India

[3]Department of Botany

St. Joseph's College autonomous

Bangalore, Karnataka,

India

[4]Sericultural Research Institute,

Zhejiang Academy of Agricultural Sciences

Hangzhou 310021, China

[†]These authors contributed equally to this work.

[*]Corresponding authors:

Guoxin Shen, Ph.D., Professor, Tel: +86-571-86404298; Fax: +86-571-86404298

Email address: guoxin.shen@ttu.edu

Neelam Mishra, Ph.D., Assistant professor

Email address: neelamiitkgp@gmail.com; neelammishra@sjc.ac.in

Orcid id:

1. Abhirup Paul: 0000-0003-2143-7511
2. Neelam Mishra: 0000-0001-6191-5392

Abstract

Mitogen activated protein kinase kinase kinase (MAPKKK) form the upstream component of MAPK cascade. It is well characterized in several plants such as Arabidopsis and rice however the knowledge about MAPKKKs in tea plant is largely unknown. In the present study, MAPKKK genes of tea were obtained through a genome wide search using *Arabidopsis thaliana* as the reference genome. Among 59 candidate MAPKKK genes in tea, 17 genes were MEKK-like, 31 genes were Raf-like and 11 genes were ZIK- like. Additionally, phylogenetic relationships were established along with structural analysis, which includes gene structure, its location as well as conserved motifs, cis-acting regulatory elements and functional domain signatures that were systematically examined, ~~and further, predictions were validated by the results.~~ Also, on the basis of orthologous genes in Arabidopsis, functional interaction was carried out in *C. sinensis.* The expressional profiles indicated major involvement of MAPKKK genes from tea in response to various abiotic stress factors. Taken together, this study provides the targets for additional inclusive identification, functional study, and provides comprehensive knowledge for a better understanding of the MAPKKK cascade regulatory network in *C. sinensis*.

Keywords: Mitogen Activated Protein Kinase; *Arabidopsis thaliana*; Phylogenetic relationship; Functional interaction; Abiotic stress

Introduction

Mitogen-activated protein kinase (MAPK) cascades are universal signal transduction modules existing in eukaryotes, including yeasts, animals and plants. MAPKKKs (Mitogen activated protein kinase kinase kinase), which form the upstream component of three tier kinase module are usually activated by G-proteins (Guanine nucleotide binding protein) but sometimes activation is also done via an upstream MAP4K [1]. MAPKKKs are the first component of this phosphorelay cascade, which phosphorylates two serine/threonine residues in a conserved S/T-X_{3-5}-S/T (Serine/Threonine-X_{3-5}-Serine/Threonine) motif of the MKK (Mitogen activated protein kinase kinase) activation loop. MKKs that are dual-specificity kinases, activate the downstream MAPK through TDY or TEY phosphorylation motif in the activation loop (T-loop) [2, 3]. The activated MAPK ultimately phosphorylates various downstream substrates, including transcription factors and other signalling components that regulate the expression of downstream genes [4]. MAPKKKs form the largest group among MAPK cascade, with 80 members in Arabidopsis, 75 members in rice, 74 members in maize and 89 members in tomato [5, 6]. This largest group is further subdivided into three smaller groups on the basis of sequence similarities 1) MEKK subfamily 2) Raf subfamily 3) ZIK subfamily [6, 7]. Compared to MAPKs and MAPKKs, the MAPKKKs have more members and greater variety in primary structures and domain composition [8]. Phylogenetic analysis of the MAPKKK genes in various species reveals the diversity in plants. Among the MAPKKKs, the Raf subfamily is the largest group and comprises of 46 members in maize, 43 in rice, 27 in grapevines, and 48 in Arabidopsis. It is followed by the MEKK subfamily, which is the second largest family and comprises of 22 members in maize, 22 in rice, 9 in grapevine, and 21 in Arabidopsis. The ZIK subfamily is the smallest among the three subfamilies and comprises of 6 members in maize, 10 in rice, 9 in grapevines, and 11 in Arabidopsis [5, 6, 9]. The MEKK subfamily comprises of a conserved kinase domain

G(T/S)Px(W/Y/F)MAPEV [5]. The ZIK subfamily contains TPEFMAPE(L/V)Y while the Raf subfamily has GTxx(W/Y)MAPE as their conserved domain signatures [5]. All the MAPKKK proteins have a kinase domain, and most of them have a serine/threonine protein kinase active site [10]. Structural domain analysis of MAPKKKs in Arabidopsis, rice and cucumber showed that most of the Raf proteins have a C-terminal kinase domain and a long N-terminal regulatory domain. In contrast, members of the ZIK group have the N-terminal kinase domain, while members of the MEKK group have a less conserved kinase domain that lies in either N or C-terminals or present in the central part of the protein [6, 9, 11]. MAPKKKs play a significant role in distinct biological and physiological processes, and they have potential that could be utilized for the development of stress-tolerant transgenic plants [12]. Two of the best studied Arabidopsis MAPKKKs are EDR1 (Enhanced disease resistance) and CTR1 (Constitutive triple response) which are known to participate in defense responses and ethylene signalling respectively [2, 13, 14].

Camellia sinensis more commonly known as tea is the second most consumed beverage in the world besides water. Tea plant is an important commercial crop potentially rich in variety of bioactive ingredients. Many genome wide studies of different gene families have been carried out in tea however, the MAPKKK genes and its role in stress response in tea plant have not been studied in detail. In the present study, the MAPKKK family of genes was thoroughly defined on the basis of *in-silico* genome-wide search in tea using *Arabidopsis thaliana* as the reference genome. Gene locations on scaffolds, their structures, the cis-regulatory elements and their evolutionary aspect were systematically studied. Further, we analysed the interaction networks of proteins based on orthologous genes in Arabidopsis. This study provides an insight on structural and functional aspect of Mitogen Activated Protein Kinase Kinase Kinase gene family in *C. sinensis* and also highlights the MAPK signalling cascade-mediated pathway of *C. sinensis*.

Materials and methods

Identification of MAPKKK gene family in Tea

The predicted peptide sequences of tea were downloaded from the Tea Plant Information Archive (TPIA) database (http://tpia.teaplant.org/) [15]. To identify tea MAPKKK genes, a total of 415 previously known MAPKKK genes were retrieved from *Arabidopsis thaliana* (80), *Oryza sativa* (75), *Solanum lycopersicum* (71), *Solanum tuberosum* (81), *Capsicum annum* (60) and *Coffee canephora* (48) using TAIR database (https://www.arabidopsis.org/) [16], Rice Genome Annotation Project database (http://rice.plantbiology.msu.edu/) [17] and Sol Genomics Network database (https://solgenomics.net/) [18], respectively. The retrieved Arabidopsis and rice MAPKKK sequences were used as query sequences to search against the tea plant proteome database using the BLASTp algorithm with an e value set to 1e-5 and identity percentage of 50% as threshold. The identified sequences were checked to remove any chances of redundancy. Further, the obtained genes were aligned by CLUSTALW (https://www.ebi.ac.uk/Tools/msa/clustalo/) [19] and uploaded to SMART (http://smart.embl-heidelberg.de/) [20] and Pfam web tool (https://pfam.xfam.org/) to confirm the existence of kinase domains. The physicochemical properties of the identified tea MAPKKK genes were predicted using ProtParam tool incorporated in ExPASy database (https://expasy.org/) [21]. Subcellular localization of the peptides were predicted using the BaCelLo (Balanced subcellular localization predictor) (http://gpcr.biocomp.unibo.it/bacello/index.htm) [22] and TMHMM server v2.0 (http://www.cbs.dtu.dk/services/TMHMM/) [23] was employed to predict the presence of trans-membrane helices in tea MAPKKK peptide sequences.

Estimation of K_a/K_s ratios

K_a and K_s ratios were calculated using the SNAP v.2.1.1 online tool (https://www.hiv.lanl.gov/content/sequence/SNAP/SNAP.html) [24] to assess the synonymous and non-synonymous groups. The dN/dS values represent the selective pressure of duplicate genes and the dS values represent the time of divergence of duplication events.

Multiple sequence alignment and Phylogeny analysis

The tea MAPKKK protein sequences were subjected to multiple sequence alignment, using CLUSTALW (https://www.ebi.ac.uk/Tools/msa/clustalo/) [19] to check for conserved MAPKKK specific domains for each subfamily. Phylogenetic analyses were done separately for MEKK, Raf and ZIK sub-families, using the identified tea sequences, coupled with Arabidopsis, rice, tomato, potato, capsicum and coffee peptide sequences. The phylogenetic trees were constructed by the Neighbor-Joining algorithm of MEGA 7.0.14 [25] keeping all the parameters at default values. The consistencies of the obtained trees were assessed by the bootstrap method and replicate was set to 1000.

Intron exon structures and conserved motifs

The intron exon distribution pattern for tea MEKK, Raf and ZIK peptide sequences were analysed and visualised using the Gene Structure Display Server v2.0 (http://gsds.cbi.pku.edu.cn/) [26]. The full-length peptide sequences were uploaded to MEME suite (http://meme-suite.org/) [27] in-order to identify the conserved motifs.

Analysis of cis-regulatory elements

The promoter sequences of 2000 bp, which lies upstream of the translational start site of each of the tea MAPKKK genes were retrieved from the TPIA database. The PlantCARE database (http://bioinformatics.psb.ugent.be/webtools/plantcare/html/) [28, 29] was used for identifying and analysing the cis-acting regulatory elements in the promoter regions of the tea MAPKKK genes.

Mapping of tea MAPKKK genes onto scaffolds and gene duplication

TPIA database has incomplete genome assembly information. As a result, the tea MAPKKK genes were mapped onto their respective scaffolds using MapGene2chromosome web v2 (MG2C) software tool (http://mg2c.iask.in/mg2c_v2.0/) [30]. The genes were mapped according to their scaffold positional information available in TPIA database, which includes scaffold IDs for each gene, scaffold dimensions and the starting and ending position of each gene on the scaffolds.

GO ontology annotation and functional interaction network

GO Ontology (GO) analysis was also performed for all the tea MAPKKKs using QuickGO (QuickGO (ebi.ac.uk)). Furthermore, the network of functionally interacting orthologous genes between tea and Arabidopsis was identified and constructed using STRING online tool ((https://string-db.org/) [31] with default parameters.

Expression profiles of tea MAPKKK genes

The tissue specific expression profiles, which include expression levels in apical bud, flower, fruit, young leaf, mature leaf, old leaf, root, and stem were retrieved from TPIA database. Furthermore, gene expression data under different abiotic stress (cold, drought, salt) treatment as well as under methyl jasmonate (MeJA) treatment were retrieved from TPIA database. GraphPad Prism 8 (https://www.graphpad.com/scientific-software/prism/) was used to generate respective graphs for the gene expression data of MEKK, Raf and ZIK sub-families.

Results

Identification of MAPKKK gene family in *C. sinensis*

In order to identify the MAPKKK gene family in tea (*C. sinensis*), 415 known MAPKKK peptide sequences from *Arabidopsis thaliana* (80), *Oryza sativa* (75), *Solanum lycopersicum* (71), *Solanum tuberosum* (81), *Capsicum annum* (60) and *Coffee canephora* (48) were retrieved from their respective databases. To identify and categorize the MAPKKK genes in tea, BLASTp searches were conducted against the tea protein database, using the retrieved peptide sequences from Arabidopsis and rice as query sequences. For all BLASTp searches, e value and identity percentage were set to 1e-5 and 50% as threshold, respectively (S1-S3 Table). The identified tea peptides were again screened with a Hidden Markov Model (HMM) search to confirm the presence of serine/threonine-protein kinase-like domain (PF00069). The results yielded a total of 59 potential tea MAPKKK genes, which included 17 MEKK-like, 31 Raf-like and 11 ZIK-like genes and were incorporated into the final dataset.

The physicochemical properties of the identified tea MAPKKK protein sequences were evaluated using ExPASy ProtParam tool (Table 1-3). The length and molecular weight of the 17 MEKK proteins ranged from 311 to 1191 amino acid residues and 34828.88 to 130956.46 kDa, respectively (Table 1). For the Raf proteins, it ranged from 305 to 1436 amino acid residues and 35012.57 to 159263.21 kDa (Table 2), and for the ZIK proteins, it ranged from 300 to 831 amino acid residues and 34181.96 to 94422.51 kDa (Table 3). The theoretical pI values ranged from 4.58 to 9.50 for MEKK, 4.88 to 9.61 for Raf and 5.14 to 6.33 for ZIK proteins, indicating that most of the MEKK and Raf proteins have a basic nature while the

ZIK proteins are acidic in nature. The grand average of hydropathy (GRAVY index) in all the extracted MEKK, Raf and ZIK proteins were negative, ranging from -0.605 to -0.060, -0.661 to -0.182 and -0.582 to -0.350 respectively. This indicates that all the identified 59 tea MAPKKKs are hydrophilic in nature. 52 of the 59 putative tea MAPKKKs had instability index values above 40, while 6 Raf genes (TEA000933.1, TEA022171.1, TEA011280.1, TEA031223.1, TEA007232.1 and TEA013875.1) and 1 ZIK gene (TEA020112.1) had instability index values less than 40 (Table 1-3). This signifies the unstable nature of most of the identified tea MAPKKKs. Subcellular localization predicted 48 genes being localized in the nucleus, 9 genes in chloroplast and 2 genes in cytoplasm (Table 1-3). The presence of trans-membrane helices in the putative peptide sequences was also ~~done~~ and one of the ZIK gene (TEA027328.1) had one trans-membrane helix (S1-S3 Figs).

Table 1. Sequence characteristics and physicochemical properties of MAPKKKs belonging to MEKK subfamily in *C. sinensis.* Locus position, gene length, protein length, molecular weight and pI value, no. of negative and positive residues, GRAVY index, instability index, aliphatic index and subcellular localizations were analysed.

Gene ID	Locus position	Gene length (bp)	Protein length (aa)	Mol. Wt. (kDa)	pI value	No. of negative residues	No. of positive residues	GRAVY index	Instability index	Aliphatic index	Subcellular localization
TEA028357.1	Scaffold856:196999-204246-	7247	628	68667.76	5.60	77	67	-0.380	58.36	76.85	Nucleus
TEA025870.1	Scaffold790:521648-539960+	18312	776	85271.15	6.76	94	92	-0.379	45.58	81.08	Nucleus
TEA016319.1	Scaffold3144:371539-383072-	11533	627	68238.67	9.50	53	71	-0.535	50.61	68.23	Nucleus
TEA008165.1	Scaffold3102:729210-737275+	8065	1032	112285.36	9.04	84	102	-0.423	53.62	72.95	Nucleus
TEA027265.1	Scaffold1289:966535-975893+	9358	939	101539.85	9.35	80	104	-0.605	63.34	65.88	Nucleus
TEA006319.1	Scaffold2905:735285-744378+	9093	683	75479.57	9.32	62	78	-0.505	67.84	72.55	Chloroplast
TEA006473.1	Scaffold1374:1527992-1535696-	7704	710	78857.59	9.09	65	79	-0.516	69.53	71.59	Nucleus
TEA014429.1	Scaffold41:2381991-2415462+	33471	1191	130956.46	6.13	145	128	-0.350	45.47	89.93	Chloroplast
TEA031711.1	Scaffold5399:986467-998883-	12416	562	62129.83	6.31	72	69	-0.484	48.47	75.62	Nucleus
TEA001470.1	Scaffold558:920549-933450+	12901	789	87423.22	8.34	90	95	-0.313	49.68	84.13	Nucleus
TEA017119.1	Scaffold5354:234291-239017-	4726	506	56190.19	4.66	80	49	-0.481	47.12	69.53	Nucleus
TEA005306.1	Scaffold2184:2097399-2125258+	27859	1097	121164.56	5.40	162	127	-0.540	49.78	72.63	Nucleus
TEA009902.1	Scaffold438:521469-522821-	1352	450	49874.37	4.58	65	34	-0.060	44.64	91.60	Chloroplast
TEA029598.1	Scaffold944:301732-304329+	2597	423	46235.51	4.94	62	43	-0.433	51.54	74.18	Nucleus
TEA005122.1	Scaffold1857:297670-298674-	1004	334	36588.08	6.01	40	34	-0.381	46.55	78.23	Chloroplast
TEA028214.1	Scaffold613:628014-629048+	1034	344	38088.50	6.33	44	41	-0.322	45.18	79.36	Nucleus
TEA031689.1	Scaffold1549:309791-310726-	935	311	34828.88	6.04	44	40	-0.340	48.20	90.64	Nucleus

Table 2. Sequence characteristics and physicochemical properties of MAPKKKs belonging to Raf subfamily in *C. sinensis.* Locus position, gene length, protein length, molecular weight and pI value, no. of negative and positive residues, GRAVY index, instability index, aliphatic index and subcellular localizations were analysed.

Gene ID	Locus position	Gene length (bp)	Protein length (aa)	Mol. Wt. (kDa)	pI value	No. of negative residues	No. of positive residues	GRAVY index	Instability index	Aliphatic index	Subcellular localization
TEA001765.1	Scaffold1670:382409-407933-	25524	842	93193.15	5.86	107	92	-0.248	46.33	89.69	Nucleus
TEA002020.1	Scaffold3595:726640-735244+	8604	896	99135.31	6.37	111	103	-0.382	42.96	81.80	Nucleus
TEA000256.1	Scaffold3876:193108-215389+	22281	1086	119081.90	6.63	118	112	-0.441	44.80	78.36	Nucleus
TEA029086.1	Scaffold106:745738-778269+	32531	919	101696.51	5.17	119	85	-0.182	41.82	91.44	Chloroplast
TEA022129.1	Scaffold3036:784237-806418+	22181	940	104852.31	6.01	114	102	-0.217	48.52	89.81	Nucleus
TEA019143.1	Scaffold1695:623368-630213+	6845	724	79987.28	7.68	86	87	-0.609	41.33	70.98	Nucleus
TEA028452.1	Scaffold433:2415340-2426547+	11207	846	93141.05	6.10	107	93	-0.523	46.31	70.89	Nucleus
TEA016969.1	Scaffold4925:453439-477111+	23672	1107	124661.25	8.46	145	153	-0.506	46.58	77.06	Nucleus
TEA013270.1	Scaffold344:585774-608400+	22626	755	85320.07	5.83	104	84	-0.374	53.97	80.97	Nucleus
TEA026716.1	Scaffold1930:511463-522712-	11249	368	41783.40	5.63	52	44	-0.487	46.26	73.89	Nucleus
TEA028758.1	Scaffold9739:380569-387825-	7256	1213	135047.30	5.63	159	123	-0.661	51.62	66.13	Nucleus
TEA010804.1	Scaffold35:1009695-1024064+	14369	305	35012.57	6.50	44	41	-0.644	44.62	74.16	Nucleus
TEA009451.1	Scaffold1786:773656-783180-	9524	1333	148469.64	4.88	193	127	-0.547	45.20	72.24	Nucleus
TEA021421.1	Scaffold1504:1005366-1017261-	11895	1331	147137.87	5.50	181	135	-0.618	44.79	71.96	Nucleus
TEA017670.1	Scaffold1965:485241-501001+	15760	561	62970.57	5.67	78	62	-0.385	49.24	89.63	Nucleus
TEA019184.1	Scaffold4191:163416-174412+	10996	601	67890.36	5.90	76	65	-0.328	49.47	89.02	Nucleus
TEA000933.1	Scaffold397:63694-76812+	13118	407	45660.50	7.66	45	46	-0.291	38.27	81.89	Nucleus
TEA031230.1	Scaffold2248:916505-925818+	9313	489	54655.73	9.20	56	67	-0.311	43.48	87.32	Chloroplast
TEA022171.1	Scaffold382:2039496-2046332+	6836	404	44640.23	8.60	48	54	-0.379	26.52	78.89	Nucleus
TEA011280.1	Scaffold3804:571784-578194+	6410	368	41126.17	7.52	48	49	-0.312	25.58	82.91	Nucleus
TEA031223.1	Scaffold2248:107085-111327-	4242	434	48234.42	6.42	57	55	-0.280	26.84	84.22	Nucleus
TEA007232.1	Scaffold3038:2387807-2395630-	7823	368	41118.03	7.02	48	48	-0.424	35.65	77.34	Nucleus
TEA016553.1	Scaffold1761:1968012-1984037-	16025	432	49062.23	8.45	65	69	-0.513	43.90	83.06	Nucleus
TEA033032.1	Scaffold858:331961-346154-	14193	415	46688.59	6.05	59	52	-0.355	43.89	86.53	Nucleus
TEA001764.1	Scaffold619:1545624-1550286+	4662	351	39474.64	6.47	42	39	-0.191	43.90	86.72	Cytoplasm
TEA026000.1	Scaffold3457:1062923-1073461-	10538	1296	144765.39	5.40	177	122	-0.507	42.22	73.72	Nucleus
TEA033556.1	Scaffold192:400250-403690-	3440	541	61612.16	9.27	57	72	-0.371	46.58	86.19	Chloroplast
TEA013875.1	Scaffold5449:126808-131150+	4342	341	39047.30	6.76	44	42	-0.256	36.12	91.52	Cytoplasm
TEA002722.1	Scaffold1369:145416-156759+	11343	1436	159263.21	5.41	203	153	-0.566	45.20	72.12	Nucleus
TEA030052.1	Scaffold319:1148438-1156900+	8462	1357	148194.52	5.00	166	110	-0.444	50.17	73.96	Nucleus
TEA008343.1	Scaffold142:344598-352450+	7852	334	37992.05	9.61	35	46	-0.257	46.78	86.17	Nucleus

Table 3. Sequence characteristics and physicochemical properties of MAPKKKs belonging to ZIK subfamily in *C. sinensis.* Locus position, gene length, protein length, molecular weight and pI value, no. of negative and positive residues, GRAVY index, instability index, aliphatic index and subcellular localizations were analysed.

Gene ID	Locus position	Gene length (bp)	Protein length (aa)	Mol. Wt. (kDa)	pI value	No. of negative residues	No. of positive residues	GRAVY index	Instability index	Aliphatic index	Subcellular localization
TEA010125.1	Scaffold52:664232-675917-	11685	675	76706.84	5.67	91	68	-0.546	47.51	72.79	Nucleus
TEA022762.1	Scaffold9600:223976-236388+	12412	732	83655.02	5.87	103	83	-0.419	50.69	89.07	Nucleus
TEA024720.1	Scaffold1050:31289-41391+	10102	655	74571.17	6.33	89	81	-0.518	51.00	77.68	Nucleus
TEA002087.1	Scaffold754:205321-211058-	5737	719	81783.51	5.54	108	80	-0.582	42.80	75.41	Nucleus
TEA013346.1	Scaffold5883:262251-272009+	9758	831	94422.51	5.53	124	93	-0.504	43.60	79.06	Nucleus
TEA013344.1	Scaffold5883:191507-194485+	2978	481	55933.38	5.65	72	58	-0.472	40.70	80.04	Nucleus
TEA031068.1	Scaffold1571:837990-857219+	19229	762	86343.00	5.92	98	76	-0.375	40.49	85.07	Chloroplast
TEA020698.1	Scaffold2762:535605-540535-	4930	664	75716.56	5.63	95	74	0.439	46.00	81.91	Nucleus
TEA027328.1	Scaffold688:688353-693133+	4780	748	84782.46	5.48	100	81	-0.350	42.68	80.16	Chloroplast
TEA020112.1	Scaffold1093:624579-626760+	2181	300	34181.96	5.60	47	40	-0.428	33.33	85.47	Nucleus
TEA033250.1	Scaffold4160:2129637-2136050+	6415	622	69665.68	5.14	99	74	-0.449	43.80	84.00	Nucleus

Phylogenetic analysis of tea MAPKKKs

A phylogenetic analysis of the putative tea MAPKKK genes was carried out to evaluate the evolutionary relationships. MEGA 7.0.14 was used to generate the phylogenetic trees, using the Neighbor-Joining (NJ) algorithm, at default parameters and 1000 bootstrap replicates. Three different phylogenetic trees were constructed for MEKK, Raf and ZIK proteins, comprising of the identified tea sequences and already known 415 MAPKKK sequences from Arabidopsis, rice, tomato, potato, capsicum and coffee. For MEKK, the NJ tree was generated using 17 sequences from tea, 21 sequences from Arabidopsis, 22 sequences from rice, 17 sequences from tomato, 22 sequences from potato, 17 sequences from capsicum and 12 sequences from coffee (Fig 1A). The NJ tree was divided into 4 distinct clades, with an uniform distribution of genes in Clade A. Clade B consisted of only 6 capsicum genes while

clade D had only 2 genes of potato. Clade C however, had a share of tomato and potato gene clusters. For Raf, the NJ tree was generated using 31 sequences from tea, 48 sequences from Arabidopsis, 43 sequences from rice, 44 sequences from tomato, 43 sequences from potato, 37 sequences from capsicum and 28 sequences from coffee (Fig 1B). Unlike the MEKK tree, the Raf tree was divided into 11 different clades, with an uniform clustering of genes in all the clades. The NJ tree for ZIK was generated using 11 sequences from tea, 11 sequences from Arabidopsis, 10 sequences from rice, 10 sequences from tomato, 16 sequences from potato, 6 sequences from capsicum and 8 sequences from coffee (Fig 1C). The ZIK tree was divided into 7 clades and had a uniform clustering of genes in all the clades with only clade E consisting of 2 genes each of Arabidopsis and rice. The results are suggestive of the fact that the genes are either homologous or orthologous to each other. However, the Raf and ZIK genes did not feature any orthologous gene with respect to Arabidopsis. This was validated only when the Raf and ZIK gene accession IDs were scanned in the TPIA database to search for the presence of orthologous genes in the later part of the study (Functional Interaction Network).

Fig 1. Phylogenetic tree of (A) MEKK-like (B) Raf-like and (C) ZIK-like genes from *Arabidopsis thaliana* (black), *C. sinensis* (red), *Oryza sativa* (blue), *Solanum lycopersicum* (grey), *Solanum tuberosum* (green), *Capsicum annum* (brown), *Coffee canephora* (teal). The full-length MEKK, Raf and ZIK protein sequences were aligned using Clustal W, and the phylogenetic trees were constructed using MEGA 7.0.14 by the Neighbor-Joining (NJ) method with default parameters and 1000 bootstrap replicates.

Domain analysis of tea MAPKKKs

Among the 3 subgroups of plant MAPKKKs, the MEKK subfamily is fairly well known and characterized. Most MEKKs are known to be a part of the recognized MAP Kinase cascades, which activates the downstream MKKs. MEKK1 and MEKK2 from Arabidopsis, have been proven to play a significant role in plant innate immunity [32, 33, 34]. Similar to other plant

MAPKKKs, 16 out of 17 members of MEKK subfamily in tea displayed a characteristic conserved signature G(T/S)Px(W/Y/F)MAPEV, except TEA014429.1 (Fig 2A). Two of the most widely studied Arabidopsis Raf subfamily MAPKKKs, namely CTR1 and EDR1 are known to actively participate in ethylene mediated signalling and defense response mechanisms. All 31 members of the Raf subfamily in tea featured a conserved GTxx(W/Y) MAPE signature in its kinase domain with no exceptions (Fig 2B). The ZIK-like MAPKKKs are also known by the name WNK or with no lysine (K). They are not proven to be involved with the phosphorylation of the MKKs in plants but have specific functions. Arabidopsis ZIK1 is known to phosphorylate APRR3 *in-vitro*, which is a putative component of the circadian clock in plants and is believed to be involved in signal transduction pathway, regulating its biological activity [35]. Another ZIK cascade, involving ZIK2, ZIK5 and ZIK8 in Arabidopsis is known to regulate the flowering time by modulating the photoperiod [36]. The ZIK subfamily featured a characteristic GTPEFMAPE(L/V/M)(Y/F/L) conserved signature across all its 11 members in tea (Fig 2C) [5, 6]. The presence of these distinctive conserved signatures across the tea MAPKKKs further confirms identity and the subfamily they belong. The largest subfamily was found to be the Raf subfamily with 31 members, while the smallest was found to be the ZIK subfamily with only 11 members. These results are consistent with published reports on other plant MAPKKKs [5, 6].

Fig 2. Alignment of MAPKKKs of (A) MEKK subfamily (B) Raf subfamily and (C) ZIK subfamily in *C. sinensis*. ClustalX program was used for aligning the obtained sequences. The highlighted part (G(T/S)Px(F/Y/W)MAPEV) shows the conserved signature for the MEKK proteins. The highlighted section (GTxx(W/Y)MAPE) shows the conserved signature for the Raf proteins and the highlighted part (GTxx(W/Y)MAPE) shows the conserved signature for the ZIK proteins.

Motif composition of tea MAPKKKs

To understand the evolution and comprehend sequential characteristics of the MAPKKK proteins in tea, a conserved motif search was carried out using the MEME suite (Fig 3). Ten conserved motifs were identified in each of the three subfamilies. Almost all the tea MAPKKK proteins featured the protein kinase domain with motif 1, motif 2 and motif 3. Motif 4 was conserved across all the proteins with only one exception of TEA031230.1. Motif 5, motif 7 and motif 8 were only obtained for the ZIK subfamily with one exception of a MEKK-like TEA014429.1, which featured motif 8. Motif 6 and motif 9 were harboured by almost all the protein sequences. However, motif 10 was only specific to the MEKK and Raf subfamilies. Motif annotation revealed that motif 2 harboured a protein kinase ATP-binding site. Motif 6 contained a tyrosine kinase phosphorylation site. Motif 9 featured a serine/threonine protein kinase activation site (S4 Fig). The results suggested that proteins belonging to a same group harboured similar conserved motifs, further indicating that the classification of the tea MAPKKK subfamilies was backed by motif analyses.

Fig 3. The motif analysis of 59 identified MAPKKKs in *C. sinensis.* The motif figures were generated by MEME suite. A total of 10 motifs were identified and are marked individually.

Gene structure analysis of tea MAPKKKs

The intron-exon distribution pattern for tea MAPKKKs were analysed and visualised using the Gene Structure Display Server v2.0. Study of gene structure revealed differences in number of introns and exons, which contributes to variation in gene length. Introns or non-coding sequences are found abundantly within a genome and are regarded as an indicator of genome complexity [37, 38]. Analysis of the intron patterns could help to comprehend and provide insights into the evolution, function and regulation of the genes [37, 39, 40, 41, 42]. The analysis of the intron-exon architecture in tea revealed significant variation in the

number of introns and exons among the three subfamilies of MAPKKKs (Fig 4). However, genes belonging to the same clades had similar intron-exon distribution. The MEKK subfamily had 10 out of 17 genes (59% of the MEKK genes) possessing 6 to 16 exons (Fig 4A). TEA025870.1 had 19 exons and 18 introns in its gene. Two genes possessed 2 exons and 1 intron and the remaining 4 genes had no introns. Only 9 out of 17 genes featured UTR (Untranslated Regions) segments and 5 out of these 9 genes featured both 5' and 3' UTRs. 3 genes contained only the 5' UTR segments and 1 gene only had the 3' UTR segment. The genes belonging to the Raf subfamily had exons ranging from 6 to 18 and was featured by 27 out of 31 genes (87% of the Raf genes) (Fig 4B). TEA016969.1 featured a staggering 28 exons and was the highest among all the Raf genes. Three genes namely TEA000933.1, TEA013875.1 and TEA033556.1 had 2, 3 and 4 exons respectively which were the lowest number of exons found amongst all the Raf genes. 29 out of 31 genes possessed UTR segments. However, only 17 of the 29 genes had both 5' and 3' UTRs. 7 genes featured only the 5' UTR segment and remaining 5 genes only had the 3' UTR. Unlike the MEKK and Raf subfamilies, ZIK subfamily displayed a certain level of conservancy with respect to the number of exons and introns. 10 out of 11 ZIK genes (91% of the ZIK genes) had exons ranging from 7 to 10 (Fig 4C). TEA020112.1 however featured only 2 exons. 9 out of 11 genes possessed UTR segments and 5 of them had both 5' and 3' UTRs. 4 genes featured only the 5' UTR segment. However, no ZIK subfamily gene in tea featured only the 3' UTR segment like the MEKK and Raf subfamilies.

Fig 4. The intron/exon architecture of (A) MEKK (B) Raf and (C) ZIK genes in *C. sinensis*. Gene structure maps were drawn using the Gene Structure Display Server 2.0. Black boxes represent exons, blue boxes represent the UTRs and black lines represent introns. The gene length can be estimated by using the scale (in kb) given at the bottom.

Retrieval of promoter sequences and analysis of cis-regulatory elements

Cis-acting regulatory elements are often used for determining the function of genes, regulation of gene transcription and gene expression [43, 44]. In order to explore the transcriptional regulation and putative functions of the tea MAPKKK genes, promoter sequences of 2000 bp upstream of the initiation codon “ATG” was retrieved from the TPIA database. These sequences were then analysed using the PlantCARE database for the identification of the cis-acting regulatory elements (CAREs). It was found that the cis-acting elements were randomly scattered in the promoter regions of the tea MAPKKKs. The study revealed an aggregate of 56 CAREs in all the tea MEKK, Raf and ZIK genes (S4 Table). These elements were also arranged and grouped based on their specific biological functions (Fig 5A). The sequence length of the cis-acting elements ranged from 5 to 14 bp (Fig 5B) with most of the CAREs having sequence lengths of 6 and 9 bp. The analysis of the 56 CAREs revealed the involvement of 13 elements in plant growth and regulation, 26 in light responsiveness, 6 were stress response elements and the remaining 11 were involved in phytohormone responses. The light responsive elements comprised of the largest section of the identified CAREs in all of the 59 tea MAPKKKs with 26 regulatory elements. Among these, Box-4 and G-Box accounted for the major part in 51 and 45 tea MAPKKKs. Some of the other light response elements included AE box, GATA motif, GT1-motif, MRE and TCT motif. The CAREs related to the phytohormone responses mainly involved abscisic acid responsive element (ABRE), and MeJA responsive elements (CGTCA-motif and TGACG-motif) in 42 and 38 tea MAPKKKs accordingly. Other phytohormone responsive elements comprised of gibberellin responsive elements (TATC-box, P-box and GARE-motif), auxin responsive elements (TGA-element, AuxRR-core, TGA-box and AuxRE) and salicylic acid responsive element (TCA-element) in 35, 33 and 26 tea MAPKKKs respectively. Among the cis-elements which are associated with plant growth and development, 21 tea MAPKKKs possessed meristem expression elements (CAT-box and NON box) while 18 genes had zein

metabolism regulatory element (O2-site). Other plant growth related CAREs included regulatory elements (A-box and Box-II like sequences), endosperm expression element (GCN4_motif), circadian control, cell cycle regulation (MSA-like), Box-III and few seed specific (RY-element), root specific (motif I) and palisade mesophyll differentiation (HD-Zip 1) element that were discovered on the promoter regions of 19, 12, 8, 3, 7, 2, 1 and 1 tea MAPKKKs respectively. In addition, numerous stress response CAREs were also identified in the promoter regions. These included ARE (anaerobic induction element), LTR (low temperature responsiveness), TC-rich repeats (defense and stress responsiveness), MBS (drought inducibility), GC-motif (anoxic specific inducibility) and AT-rich sequences (maximal elicitor mediated activation) in 52, 19, 25, 18, 8 and 6 tea MAPKKKs respectively. These results indicate the involvement of MAPKKK genes in various responses like phytohormone treatments, low temperature, physiological stresses and plant growth and regulation.

Fig 5. Analysis of cis-acting elements identified from the MEKK, Raf and ZIK genes of *C. Sinensis*. All cis-acting elements have been identified using PlantCARE database. (A) Pie-chart showing the frequency of different cis-acting elements based on their specific biological activities. (B) Histogram showing the frequency of different sequence lengths of the cis-acting elements.

Genomic distribution map and evolutionary pressure on tea MAPKKKs

The tea MAPKKKs were mapped onto the genomic scaffolds to understand their distribution pattern. Due to the lack of chromosome-level assembly data in the TPIA database, the genes were mapped onto their respective scaffolds instead of the chromosomes. All 59 tea MAPKKKs were extensively distributed across 58 different genomic scaffolds. 17 MEKK genes were distributed across 17 different scaffolds (Fig 6A). Similarly, 31 Raf genes were distributed across 31 genomic scaffolds (Fig 6B). 11 ZIK genes were mapped onto 10 genomic scaffolds (Fig 6C). Two ZIK genes namely, TEA013344.1 and TEA013346.1 were

mapped on the same genomic scaffold 5883 and thus featured a duplication event. Additionally, both these genes possessed similar intron-exon architecture. This result is conclusive evidence that duplication events were of significant importance and played a crucial role in the expansion of the MAPKKK genes in *C. sinensis* genome. Further, the ratio of non-synonymous substitution rates (K_a) and synonymous substitution rates (K_s) was evaluated to illuminate the mechanism of gene divergence and evolutionary pressure on the tea MAPKKKs. The ratio determines the selective pressure acting on the respective proteins. If the K_a/K_s ratio is <1, it determines negative or purifying selection. If the K_a/K_s ratio is =1, it indicates neutral selection and if the K_a/K_s ratio is >1, it signifies positive selection [45]. For the MEKK subfamily, pair wise comparisons revealed that 72 gene pairs had K_a/K_s ratios above 1, indicating that they are under positive selection, 24 gene pairs had values less than 1, indicating a negative selection and remaining 40 were not a number (Nan) (S5 Table). Similarly, K_a/K_s ratios of the Raf subfamily revealed 341 gene pairs in positive selection, 96 in negative selection and 28 pairs as Nan (S6 Table). K_a/K_s ratios of ZIK subfamily uncovered 30 pairs in positive selection, 21 in negative selection and the remaining 4 as Nan (S7 Table). The K_a/K_s cumulative graphs of tea MAPKKKs were also generated (S5-S7 Figs). The results suggest strong positive selection pressures would have occurred, enabling different factors to regulate the MAPKKKs in *C. sinensis*.

Fig 6. The scaffold distribution of (A) MEKK subfamily (B) Raf subfamily and (C) ZIK subfamily genes in *C. sinensis*. MapGene2chromosome web v2 (MG2C) software tool (http://mg2c.iask.in/mg2c_v2.1/) was used to map genes onto their respective scaffolds. The scaffolds are drawn to scale and the scaffold numbers are indicated on the top.

GO ontology analysis and functional interaction network of tea MAPKKKs

The GO ontology analysis was performed in order to predict the potential functions of all the 59 tea MAPKKKs (S8 Fig). All the MEKK, Raf and ZIK proteins were assigned into three

major groups and 50 subgroups. The 3 major groups were biological process, cellular component and molecular function. In the first group, the proteins were distributed into 29 subgroups with ‘protein phosphorylation’ (GO:0006468, 26 sequences, 44.07%) with the largest representation. In the cellular component group, the MAPKKK proteins were distributed into 8 subgroups. Among these 8, ‘cytosol’ (GO:0005829, 4 sequences, 6.78%) had the highest representation followed by ‘cytoplasm’ (GO:0005737, 3 sequences, 5.08%) and ‘intracellullar’ (GO:0005622, 3 sequences, 5.08%). The molecular function group featured 16 subgroups with ‘ATP binding’ (GO:0005524, 28 sequences, 27.12%) and ‘protein kinase activity’ (GO:0004672, 25 sequences, 42.37%) having highest representation. They were followed by ‘protein serine/threonine kinase activity’ (GO:0004674, 16 sequences, 27.12%) and ‘kinase activity’ (GO:0016301, 13 sequences, 22.03%).

For better understanding of the interactions of MAPKKKs in *C. sinensis*, an interaction network was constructed based on the orthologous genes in Arabidopsis, using the STRING server (Fig 7). The functional interaction network of the genes has been built using that of Arabidopsis since tea database is not included in the STRING online server. TEA005306.1 in tea was found to be orthologous to AT5G55100 in Arabidopsis. This orthologous gene was identified using the TPIA database and AT5G55100 was used to build the interaction network. Additionally, tea proteins, homologous to the Arabidopsis proteins participating in the network were incorporated in the network (Fig 7). These homologous proteins were designated as STRING proteins and were selected on the basis of high bit scores. Similarity search programs like BLAST are widely used and produce accurate statistical estimates, ensuring that protein sequences with significant similarity also have similar structures [46]. Proteins that have high sequence and structural similarity generally tend to possess similar functions [47]. Based on TAIR database, AT5G55100 is involved in RNA processing and is expressed during 15 growth stages in 24 different organs and tissue of plant. It shows

interactions with AT4G33690 which is involved in biological process of protein binding [16]. AT2G29210 is involved with RNA splicing, mRNA processing and is expressed during 13 different growth stages in 23 different organs and tissues [16]. ATO (AT5G06160) encodes for a protein similar to pre-mRNA splicing factor SF3a60 and is involved in gametic cell fate determination [16]. Loss of function results in the ectopic expression of egg cell makers, thereby suggesting a role in restriction of gametic cell fate. TEA031585.1, which is homologous to ATO gene, is a part of the spliceosomal complex and is involved in mRNA splicing based on GO ontology. TK1 (AT2G36960) is a TSL-kinase interacting protein and is involved in protein binding [16]. It is expressed in 14 developmental stages in 25 different plant organs and tissue. GPT (Glucose-6-Phosphate translocator) (AT2G41490) is an integral component of membrane and has a UDP-N-acetylglucosamine-dolichyl-phosphate N-acetylglucosamine phosphotransferase activity [16]. It is expressed during 15 developmental stages in 23 different organ and tissue in the plant. AT3G57220 is located in the endoplasmic reticulum and has a UDP-N-acetylglucosamine-dolichyl-phosphate N-acetylglucosamine phosphotransferase activity. It is also linked with polysaccharide biosynthesis and is expressed during 10 growth stages in 16 different plant tissue and organ [16]. According to GO ontology, TEA009318.1 is also involved in phosphotransferase activity in tea and is homologous to both AT2G41490 and AT3G57220 in Arabidopsis.

Fig 7. Functional interaction network of tea MAPKKK proteins. The interaction network was build according to the ortholog in Arabidopsis. TEA005306.1 in tea is orthologous to AT5G55100 in Arabidopsis. The orthologous protein (red) and homologous proteins (black) are shown within brackets.

Tissue specific gene expression of tea MAPKKKs

The tissue specific expression pattern of the tea MAPKKK genes in various plant tissues were retrieved from the TPIA database where levels of expression were expressed using

transcripts per million (TPM). The TPIA database houses tissue specific expression data for 8 different plant tissues which includes apical bud, flower, fruit, young leaf, mature leaf, old leaf, root and stem (S8 Table). Among the 59 tea MAPKKK genes, expression data for 58 genes were retrieved with an exception of 1 MEKK gene, TEA031689.1. All 58 genes displayed varied levels of expression, with few of the transcripts barely readable (Fig 8). For the MEKK genes, the maximum level of expression in apical bud was shown by TEA006319.1. This gene also marked the highest level of expression in young leaf. TEA017119.1 showed highest level of expression in flower. TEA016319.1 displayed highest expression levels in fruit, mature leaf, old leaf and stem. TEA005122.1 was expressed maximum in root. TEA028357.1 and TEA009902.1 had negligible levels of expression in all of the 8 plant tissues (Fig 8A). For the Raf genes, TEA000933.1 showed highest levels of expression in apical bud, fruit, young leaf, mature leaf, old leaf, root and stem. TEA007232.1 was expressed maximum in flower. However, TEA001765.1, TEA013270.1, TEA028758.1 and TEA031230.1 had negligible levels of expression (Fig 8B). Finally, for the ZIK genes, TEA002087.1 displayed highest levels of expression in apical bud, flower, young leaf and stem. TEA022762.1 had highest levels of expression in fruit, mature leaf and old leaf. TEA020112.1 showed maximum expression in root. However, TEA013344.1, TEA031068.1, TEA020698.1 and TEA027328.1 showed minor levels of expression (Fig 8C). Heat maps for all the 58 genes, representing the tissue specific expression levels were also being generated (S9 Fig).

Fig 8. Tissue-specific expression patterns of (A) MEKK (B) Raf and (C) ZIK genes in *C. sinensis*. The relative expression of these genes were analysed in different tissues by using GraphPad Prism 8 software. The level of expression was in transcript per million (TPM). 58 out of the 59 identified genes had expression data in TPIA database with an exception of 1 MEKK gene (TEA031689.1).

Abiotic stress induced differential expression levels of tea MAPKKKs

The expression data of the leaves of the tea plant present in TPIA database (S9-S12 Table) was used in order to study the effect of cold, drought, salt stress along with methyl jasmonate (MeJA) treatment followed by the generation of expression graphs for the same (Fig 9-12). The cold acclimated (CA) data (unpublished), present in the TPIA database consists of 5 stages of expression. These are: 1. 25~20 °C (CK), 2. Fully acclimated at 10 °C for 6 h (CA1-6h) 3. 10~4 °C for 7 days (CA 1-7d), 4. Cold response at 4~0 °C for 7 days (CA 2-7d) and 5. Recovering under 25~20 °C for 7 days (DA-7d), where CK is the control [48]. Expression of MEKK genes revealed that 15 out of 17 genes were upregulated under CA 1-6h. TEA006473.1 was downregulated while TEA031689.1 displayed no expression levels. Expression levels under the CA 1-7d condition showed that 12 genes were upregulated, 4 genes were downregulated and remaining 1 gene showed no data. Under the CA 2-7d condition, expression levels revealed that 10 genes were upregulated, 6 genes were downregulated and remaining 1 gene displayed no expression data. Lastly, under the DA-7d condition, data revealed that 13 genes showed upregulation, 3 genes showed downregulation and 1 gene had no data (Fig 9A). The Raf and ZIK genes were also analysed based on the same 5 conditions. For the Raf genes, under CA 1-6h condition, 22 genes out of 31 were upregulated and 9 genes were downregulated. Under CA 1-7d condition, 16 genes were upregulated and 15 genes were downregulated. Expression levels under CA 2-7d revealed that 17 genes showed upregulation and remaining 14 genes showed downregulation. Under DA-7d condition, 21 genes were upregulated and 10 genes were downregulated (Fig 9B). Expression data of the ZIK genes revealed that under CA 1-6h, 7 out of 11 genes were upregulated and 4 genes were downregulated. CA 1-7d condition revealed that 5 genes were upregulated, 5 genes were downregulated and remaining 1 gene displayed no expression. Under CA 2-7d condition, 4 genes were upregulated, 6 genes were downregulated and 1 gene had no expression. Finally, under DA-7d, 8 genes showed upregulation and remaining 3

showed downregulation (Fig 9C). Heat maps for the retrieved expression data were also generated (S10 Fig).

Further, expression levels of all tea MAPKKKs were checked under drought stress conditions. Expression levels under drought stress are available in the TPIA database with respect to 25% polyethylene glycol (PEG) treatment and it includes 4 different stages: 1. 0h; 2. 24h; 3. 48h; and 4. 72h [49], where 0h was taken as the control. The expression levels of MEKK genes revealed that under PEG-N-24h condition, 12 genes were upregulated, 4 were downregulated and 1 gene did not show any expression. Under PEG-N-48h, 12 genes were upregulated, 4 were downregulated and 1 gene showed no expression. PEG-N-72h revealed 11 genes showing upregulation, 5 genes showing downregulation and 1 gene with no expression (Fig 10A). Expression of Raf genes showed that under the PEG-N-24h condition, 11 genes were upregulated, 20 genes were downregulated. Under PEG-N-48h, 16 genes showed upregulation while the remaining 15 genes were downregulated. PEG-N-72h revealed that 15 genes were upregulated and 16 genes were downregulated (Fig 10B). Finally, the expression data of ZIK genes revealed 10 out of 11 genes had different expression levels under the given conditions while 1 gene (TEA013344.1) had no data. Under the PEG-N-24h condition, expression data showed that only 1 gene was upregulated while the rest of the genes were downregulated. PEG-N-48h condition too revealed the same result with only 1 gene being upregulated. However, PEG-N-72h showed that 2 genes were upregulated and the rest of the genes were downregulated (Fig 10C). Heat maps for the afore-mentioned data were also generated (S11 Fig).

The expression levels of the tea MAPKKKs under salt stress condition were studied. Similar to the drought stress parameters, the salt stress data in TPIA database is recorded based on treatment with 200 mM NaCl under 4 stages: 1. 0h; 2. 24h; 3. 48h; and 4. 72h where 0h was taken as the control. Analysis of the MEKK genes revealed that under NaCl-N-24h, 9 genes

were upregulated and 8 genes were downregulated. For NaCl-N-48h condition, 9 genes showed upregulation and remaining 8 genes were downregulated. Expression levels under NaCl-N-72h revealed 5 genes being upregulated and the rest being downregulated (Fig 11A). For the Raf genes, expression data suggested that under NaCl-N-24h condition, 15 genes were upregulated and 16 genes were downregulated. Under the NaCl-N-48h condition, 16 genes showed upregulation and 15 genes were downregulated. Expression levels under NaCl-N-72h showed that 8 genes were upregulated and remaining 23 were downregulated (Fig 11B). For ZIK genes, 10 out of 11 genes had expression levels while 1 gene (TEA013344.1) had no effect under the given conditions. Expression levels under NaCl-N-24h condition, only 2 genes showed upregulation and the rest of the genes were downregulated. For NaCl-N-48h condition, only 1 gene was upregulated while the remaining 9 were downregulated. NaCl-N-72h condition too revealed a similar result with 2 genes being upregulated and remaining 8 being downregulated (Fig 11C). Heat maps were generated for the above-mentioned data as well (S12 Fig).

Finally, the expression levels of the tea MAPKKKs under MeJA treatment were studied and analysed. The hormonal treatment data is recorded based on the results of exposing the plant parts to aqueous solution of MeJA, under 4 stages: 1. 0h: 2. 12h: 3. 24h and 4. 48h where, 0h was used as the control. For the MEKK genes, under the 12h_MeJA condition, 3 genes showed upregulation, 13 genes were downregulated and remaining 1 gene had no expression at all. Under the 24h_MeJA condition, 4 genes were upregulated, 12 were downregulated and 1 gene was not expressed at all. Under 48h_MeJA condition, 8 genes were upregulated and 9 genes were downregulated (Fig 12A). Similarly, for the Raf genes, treatment under 12h_MeJA condition revealed that 10 out of 31 genes were upregulated and remaining 21 genes were downregulated. Under 24h_MeJA condition, 8 genes showed upregulation while 23 genes were downregulated. 48h_MeJA revealed that only 4 genes were upregulated and

rest of the genes were downregulated (Fig 12B). ZIK genes under the 12h_MeJA condition revealed that 4 genes were upregulated and 7 genes were downregulated. 24h_MeJA condition showed 5 genes being upregulated and remaining 6 being downregulated. 48h_MeJA condition suggested that 3 genes were upregulated and remaining 8 being downregulated (Fig 12C). Heat maps for these data were also generated (S13 Fig). A similar approach was taken for highlighting biotic stress responsive and defensive role of chitinase genes in tea [50].

Fig 9. Gene expression patterns of (A) MEKK (B) Raf and (C) ZIK genes, under cold stress conditions in *C. sinensis*. The relative expression of these genes were analysed in different ~~tissues~~ by using GraphPad Prism 8 software. The level of expression was in transcript per million (TPM).

Fig 10. Gene expression patterns of (A) MEKK (B) Raf and (C) ZIK genes, under drought stress conditions in *C. sinensis*. The relative expression of these genes were analysed in different ~~tissues~~ by using GraphPad Prism 8 software. The level of expression was in transcript per million (TPM).

Fig 11. Gene expression patterns of (A) MEKK (B) Raf and (C) ZIK genes, under salt stress conditions in *C. sinensis*. The relative expression of these genes were analysed in different ~~tissues~~ by using GraphPad Prism 8 software. The level of expression was in transcript per million (TPM).

Fig 12. Gene expression patterns of (A) MEKK (B) Raf and (C) ZIK genes, under Methyl jasmonate (MeJA) treatment in *C. sinensis*. The relative expression of these genes were analysed in different ~~tissues~~ by using GraphPad Prism 8 software. The level of expression was in transcript per million (TPM).

Discussion

The MAPKKK-MAPKK-MAPK signalling cascade plays an important role in plant development as well as in response to various environmental stresses [5, 34, 51]. Investigation of the MAPKKK genes, which form a significant component of this core regulatory network would certainly aid to a better understanding of the signalling genes. Although much progress has been made in identifying the functions of MAPKKK genes in

many organisms, these genes are yet to be analysed in *C. sinensis*. The objective of this study was to provide a comprehensive synopsis of the phylogenetic relationship, intron-exon architecture, motifs, functional domains, cis regulatory elements, genomic distribution and expression patterns of the MAPKKK genes in tea. Herein, a grand total of 59 MAPKKK proteins were screened and identified from tea plant genome. Previous studies in Arabidopsis [33], cucumber [10] and rice [6] have showed that the genes of MAPKKK family are classified into 3 subfamilies namely MEKK, Raf and ZIK [33, 10, 6]. Phylogenetic analysis (Fig 1) in tea showed similar results which indicate that genes in tea are also classified into these subfamilies. All the identified tea MAPKKKs had their respective subfamily specific domains. Motif analyses revealed that all MAPKKK proteins had protein kinase domains and proteins belonging to the same subfamily shared similar motifs (Fig 3). This result is consistent to previous studies conducted on other plants like cucumber [10], Arabidopsis [33] and banana [52]. The study of intron-exon architecture in tea MAPKKK genes revealed a significant variation in the number of introns and exons (Fig 4). The average number of exons in MEKK genes ranged between 6 to16. Highest number of exons found among the MEKK genes was 18. Raf genes had an average of 6 to 18 exons, with the highest being a staggering 28 found in TEA016969.1 and ZIK genes had exons between 7 to 10. Raf subfamily thereby featured more number of introns than MEKK and ZIK subfamilies. Reports suggest that the rate at which introns are lost is faster compared to the rate at which introns are gained after segmental duplication [53]. This is a conclusive evidence to infer that Raf subfamily might contain the original set of genes, from which genes of other subfamilies have been derived. The analysis also proposed that genes belonging to the same subgroup featured similar intron-exon organization. The MAPKKK genes also displayed a significant variation with respect to the UTR segments. Most genes possessed both 5’ and 3’ UTRs while few had only the 5’ UTR or 3’ UTR segment. These variations of the gene structures suggest that the tea

genome has been variable during its evolutionary history. Similar occurrence was also observed in plants like cassava [54], grapevine [55] and maize [39]. The interactions between the transcription factors and the promoter binding sites have crucial roles in regulation of gene expression at the transcriptional level [43]. The promoter sequence analysis for all the tea MAPKKKs revealed the diverse variety of cis-acting regulatory elements and their respective biological functions (Fig 5). Further in the study, all the identified genes were mapped onto their respective scaffolds (Fig 6). Duplication events observed among the ZIK genes shows the evidence that these genes play a crucial role in *C. sinensis*. The ratio of non-synonymous substitution rates (K_a) and synonymous substitution rates (K_s) was evaluated which indicated strong positive selection pressures to have occurred, enabling different factors to regulate the MAPKKKs in *C. sinensis* genome.

Earlier, comprehensive study in other plants has shown that MAPK cascade genes are extensively involved in controlling a number of biological processes which include cell growth, proliferation and response to various biotic and abiotic stresses such as salt stress, cold stress and drought stress [56, 57, 58, 59]. Tea plant is a woody perennial tree and has a life span of more than 100 years. [60]. However, traditional breeding techniques for tea are slow and ~~limited primarily~~ to selection which leads to narrowing down of its genetic base. Plants develop numerous signalling pathways to convert external stimuli into intracellular reactions in order to defend themselves against various environmental stress factors [61, 62]. MAPKKKs function at the highest level of the MAPK signalling cascade, helping with development and stress tolerance in plants.

A receptor mediated activation of MAPKKK proteins receive upstream signals to activate MAPKK proteins by phosphorylating the serine/threonine residues of the conserved motif (Ser/Thr-X3−5-Ser/Thr) [63, 64], which further phosphorylate specific MAPKs [65]. The activated MAPKK proteins further activates downstream MAPK proteins in the T-X-Y motif

[63, 64]. The phosphorylated MAPK proteins then activate multiple downstream target proteins, including transcription factors, protein kinases, and cytoskeletal components [63, 64].

MAPKKKs have been extensively studied in Arabidopsis and have been characterised. Previous literatures have conveyed that MEKK1-MKK1-MPK4 cascade is activated following a wounding stress response [66]. The MEKK1-MKK2-MPK4/MPK6 cascade is stimulated in salt and cold stress conditions [67]. Biochemical and genetic research suggests that MEKK1 is critically significant in response to cold stress and salt stress in Arabidopsis [68]. MAPK proteins classified in the same clades have been reported to perform similar roles in different organisms [69, 70]. Expression data presented in this study revealed TEA005122.1 had the highest level of expression under salt stress and this gene belongs to clade A along with AtMEKK1. A similar clustering event with AtMEKK1 was observed for TEA006319.1 which displayed the highest levels of expression under cold stress. Hence, TEA005122.1 and TEA006319.1 might get activated in response to cold and salt stress and initiate MEKK1 signalling cascade in tea. MKK3 encodes for a Mitogen Activated Protein Kinase Kinase, that stimulates MPK8, and is a target of MPKKK20, regulating ROS accumulation. MKK3-MAPKKK17-MAPKKK18 form an element of the ABA signalling pathway. MAPKKK17 and MAPKKK18 belong to Ser/Thr protein kinase family and help in the ABA-dependent activation of the MKK3-MPK7 pathway [71]. Previous study has shown that abcissic acid mediates drought stress response [72]. In our study, TEA005122.1 belonging to clade A is homologous to AtMEKK18 and shows the highest level of expression under drought stress which may be suggestive of the fact that TEA005122.1 might be responsible for drought stress responsive pathway in tea.

Analysis of gene expression in different plant parts under various environmental stress stimuli is key to understand the functions of the genes. Among the MEKK genes,

TEA016319.1 was expressed consistently in all the 8 plant tissues (Fig 8A). While for the Raf and ZIK genes, TEA000933.1 and TEA002087.1 were the consistently expressed genes (Fig 8B and Fig 8C). Reactive oxygen species (ROS) are oxygen derivatives, which are highly reactive by-products of the aerobic metabolism [73]. Plants consist of a complicated network of ROS scavenging antioxidant enzymes that helps to regulate the ROS levels under normal physiological conditions [73, 74]. Although a change from normal physiological conditions to adverse conditions shifts the equilibrium, resulting in increased ROS production. This might lead to serious oxidative damage and cell death because ROS are highly toxic to the cellular machinery [74]. Studies have suggested that the MAPK signalling cascade comprising of the MAPKKK-MAPKK-MAPK module is stimulated when excess ROS levels are detected under different stress conditions such as salt stress, cold stress and drought stress [73, 74]. It has been revealed that MPKKK1 activates two of its highly homologous MAPKKs (MKK1 and MKK2), which operate upstream of both MPK4 and MPK6 [67, 74]. Expression data for treatment under Methyl jasmonate (MeJA) revealed that TEA028214.1 among the MEKK genes, TEA000933.1 among the Raf genes and TEA002087.1 among the ZIK genes were expressed the most under the 72_MeJA condition (Fig 12). Collectively, these findings suggest the involvement of a number of MAPKKK genes, being upregulated and expressed under the stress conditions. In general, this study provides a detailed and comprehensive analysis of the MAPKKK genes in tea. Further extensive studies needs to be conducted on MAPKKK genes of tea that could provide a better understanding on the various functions of these set of genes in developmental processes and expression under various abiotic stress stimuli.

Conclusion

Mitogen activated protein kinases (MAPK) signalling cascade plays significant roles in different biological processes. The signalling components are linked to the upstream and

downstream regulators by phosphorylation. There has been substantial development in identifying the different MAPKKK genes and understand their physiological roles in various plants. However, these genes had not been yet explored and studied in tea plant. *In-silico* genome wide analysis had identified 59 MAPKKK genes from *C. sinensis* genome. The classification of the identified MAPKKK genes in 3 subfamilies were conducted based on their specific domain signatures. The genes were further investigated under phylogenetic analysis, conserved protein motifs, intron-exon architecture and analysis of cis regulatory elements. The 59 genes were mapped onto their respective genomic scaffolds and a network of functionally interacting genes was constructed. Further, expression profile analyses were conducted to reveal the involvement of the tea MAPKKK genes in various tissues during development and understand the expression pattern of these genes under various abiotic stress stimuli and plant hormonal treatment. These data will provide detailed information about the tea MAPKKK genes for further characterization of the MAPK signalling cascade and lay a concrete foothold for further exploration and research on *C. sinensis*.

Acknowledgements

This project was supported by the Key Technologies R & D Program for Crop Breeding of Zhejiang Province (2016C02054-19,2017C02010), the Natural Science Foundation of China (31670303), and the Joint Laboratory of Olive Oil Quality and Nutrition among China, Australia and Spain. The authors are thankful to DBT-eLibrary Consortium (DeLCON) for providing access to e-resources.

Author contributions: A.P., A.P.S. and S.S., designed and performed experiments, A.P.S., N.M., and G.S., devised the experiments, helped in data analysis and writing the manuscript.

Author details: Abhirup Paul: Department of Biochemistry, REVA University, Bangalore, Karnataka, India (Email: abhirupm16@gmail.com); Anurag P. Srivastava: Department of Life Sciences, Garden City University, Bangalore, Karnataka, India (Email: anurag.srivastava@gardencity.university, anuiitkgp@gmail.com); Shreya Subrahmanya: Department of Botany, St. Joseph's college autonomous, Bengaluru, Karnataka, India (Email: shreyasub916@gmail.com); Guoxin Shen: Sericultural Research Institute, Zhejiang Academy of Agricultural Sciences, Hangzhou 310021, China (Email: guoxin.shen@ttu.edu); Neelam Mishra: Department of Botany, St. Joseph's college autonomous, Bengaluru, Karnataka, India (Email: neelamiitkgp@gmail.com, neelammishra@sjc.ac.in).

Funding: Not Applicable

Data availability: All data generated or analysed during this study are included in this article and are provided in the Electronic Supplemental Materials (Supplemental_information 1 and Supplemental_information 2)

Compliance with ethical standards

Competing Financial Interests: There are no competing financial interests

Ethics approval: Not applicable

References

1. Champion, A., Picaud, A. & Henry, Y. Reassessing the MAP3K and MAP4K relationships. *Trends Plant Sci.* (2004). **9**, 123–129
2. Rodriguez, M. C., Petersen, M. & Mundy, J. Mitogen-activated protein kinase signaling in plants. *Annu. Rev. Plant Biol.* (2010). **61**, 621–649
3. Doczi, R., Okresz, L., Romero, A. E., Paccanaro, A. & Bogre, L. Exploring the evolutionary path of plant MAPK networks. *Trends Plant Sci.* (2012). **17**, 518–525
4. Colcombet, J. & Hirt, H. Arabidopsis MAPKs: A complex signalling network involved in multiple biological processes. *Biochem. J.* (2008). **413**, 217–226
5. Jonak, C., Okresz, L., Bogre, L. & Hirt, H. Complexity, cross talk and integration of plant MAPKinase signalling. *Current opinion in plant biology*. (2002). **5**, 415–424
6. Rao, K.P., Richa, T., Kumar, K., Raghuram, B. & Sinha, A. K. In silico analysis reveals 75 members of mitogen-activated protein kinase kinase kinase gene family in rice. *DNA Research.* (2010). **17,** 139–153

7. Sinha, A. K, Jaggi, M., Raghuram, B. & Tuteja, N. Mitogen-activated protein kinase signaling in plants under abiotic stress. *Plant signaling & behavior.* (2011). **6**, 196–203
8. Hamel, L. P., Sheen, J. & Seguin, A. Ancient signals: comparative genomics of green plant CDPKs. *Trends in plant science.* (2014). **19,** 79–89
9. Wu, J., Wang, J., Pan, C., Guan, X., Wang, Y., Liu, S., He, Y., Chen, J., Chen, L. & Lu, G. Genome-wide identification of MAPKK and MAPKKK gene families in tomato and transcriptional profiling analysis during development and stress response. *PloS one.* (2014). **9**,e103032. https://doi.org/10.1371/journal.pone.0103032.
10. Wang, J., Pan, C., Wang, Y., Ye, L., Wu, J., Chen, L., Zou, T. & Lu, G. Genome-wide identification of MAPK, MAPKK, and MAPKKK gene families and transcriptional profiling analysis during development and stress response in cucumber. *BMC Genomics.* (2015). **16,** 386
11. Popescu, S. C., Popescu, G. V., Bachan, S., Zhang, Z., Gerstein, M., Snyder, M. & Dinesh Kumar, S. P. MAPK target networks in Arabidopsis thaliana revealed using functional protein microarrays. *Genes Dev.* (2009). **23,** 80-92
12. Sun M, Xu Y, Huang J, et al. Global Identification, Classification, and Expression Analysis of MAPKKK genes: Functional Characterization of MdRaf5 Reveals Evolution and Drought-Responsive Profile in Apple. Sci Rep. 2017. **7**(1), 13511.
13. Huang, X., Luo, T., Fu, X., Fan, Q. & Liu J. Cloning and molecular characterization of a dehydration/drought tolerance in transgenic tobacco. *J. Exp. Bot.* (2011). **62**, 5191–5206.
14. Frye, C. A., Tang, D. & Innes, R. W. Negative regulation of defense responses in plants by a conserved MAPKK kinase. *Proc. Natl. Acad. Sci. U.S.A.* (2001). **98,** 373–378
15. Xia, E. H., Li, F. D., Tong, W., Li, P. H., Wu, Q., Zhao, H. J., Ge, R. H., Li, R. P., Li, Y. Y., Zhang, Z. Z., Wei, C. L. & Wan, X. C. Tea Plant Information Archive (TPIA): A comprehensive genomics and bioinformatics platform for tea plant. *Plant Biotechnology Journal.* (2019). **17,** 1938–1953
16. Berardini, T. Z., Reiser, L., Li, D., Mezheritsky, Y., Muller, R., Strait, E. & Huala, E. The Arabidopsis Information Resource: Making and mining the "gold standard" annotated reference plant genome. *Genesis*. (2015). **53,** 474–485
17. Kawahara, Y., de la Bastide, M., Hamilton, J. P., Kanamori, H., McCombie, W. R., Ouyang, S., Schwartz, D. C., Tanaka, T., Wu, J., Zhou, S., Childs, K. L., Davidson, R. M., Lin, H., Quesada-Ocampo, L., Vaillancourt, B., Sakai, H., Lee, S. S., Kim, J., Numa, H., Itoh, T., Buell, C. R. & Matsumoto, T. Improvement of the Oryza sativa Nipponbare reference genome using next generation sequence and optical map data. *Rice.* (2013). **6,** 4
18. Fernandez-Pozo, N., Menda, N., Edwards, J. D., Saha, S., Tecle, I. Y., Strickler, S. R., Bombarely, A., Fisher-York, T., Pujar, A., Foerster, H., Yan, A. & Mueller, L. A. The Sol Genomics Network (SGN)--from genotype to phenotype to breeding. *Nucleic Acids Res.* (2015). **43,** D1036-D1041 doi:10.1093/nar/gku1195
19. Madeira, F., Park, Y. M., Lee, J., Buso, N., Gur, T., Madhusoodanan, N., Basutkar, P., Tivey, A. R. N., Potter, S. C., Finn, R. D. & Lopez, R. The EMBL-EBI search and sequence analysis tools APIs. *Nucleic Acids Res.* (2019). **47,** W636-W641 (2019). doi: 10.1093/nar/gkz268.
20. Letunic, I., Doerks, T. & Bork, P. SMART: recent updates, new developments and status in 2015. *Nucleic Acids Res.* (2015). **43,** (Database issue):D257-D260 doi:10.1093/nar/gku949.
21. Gasteiger, E., Hoogland, C., Gattiker, A., Duvaud, S., Wilkins, M. R., Appel, R. D. & Bairoch, A. Protein Identification and Analysis Tools on the ExPASy Server; (In) John M. Walker (ed): The Proteomics Protocols Handbook, Humana Press (2005). pp. 571-607.

22. Pierleoni, A., Martelli, P. L., Fariselli, P. & Casadio, R. BaCelLo: a balanced subcellular localization predictor, *Bioinformatics.* (2006). **22,** 408-416. https://doi.org/10.1093/bioinformatics/btl222.
23. Sonnhammer, E. L. L., von Heijne, G. & Krogh, A. A hidden Markov model for predicting transmembrane helices in protein sequences. In Proc. of Sixth Int. Conf. on Intelligent Systems for Molecular Biology, (1998). p 175-182 Ed J. Glasgow, T. Littlejohn, F. Major, R. Lathrop, D. Sankoff, and C. Sensen. Menlo Park, CA: AAAI Press,
24. Korber, B. HIV Signature and Sequence Variation Analysis. Computational Analysis of HIV Molecular Sequences, Chapter 4, pages 55-72. (2000). Allen G. Rodrigo and Gerald H. Learn, eds. Dordrecht, Netherlands: Kluwer Academic Publishers.
25. Kumar, S., Stecher, G. & Tamura, K. MEGA7: Molecular Evolutionary Genetics Analysis Version 7.0 for Bigger Datasets. *Mol Biol Evol.* (2016). **33,** 1870-1874. doi:10.1093/molbev/msw054.
26. Hu, B., Jin, J., Guo, A. Y., Zhang, H., Luo, H. & Gao, G. GSDS 2.0: an upgraded gene feature visualization server. *Bioinformatics.* (2015). 31, 1296-1297
27. Bailey, T. L., Boden, M., Buske, F.A., Frith, M., Grant, C. E, Clementi, L., Ren, J., Li, W. W. & Noble, W. S. MEME SUITE: tools for motif discovery and searching. *Nucleic Acids Res.* (2009). **37,** W202-208. doi: 10.1093/nar/gkp335.
28. Rombauts S, Déhais P, Van Montagu M, Rouzé P. PlantCARE, a plant cis-acting regulatory element database. *Nucleic Acids Res.* (1999). **27**(1):295-6. doi: 10.1093/nar/27.1.295. PMID: 9847207; PMCID: PMC148162. 2.
29. Lescot M, Déhais P, Thijs G, Marchal K, Moreau Y, Van de Peer Y, Rouzé P, Rombauts S. PlantCARE, a database of plant cis-acting regulatory elements and a portal to tools for in silico analysis of promoter sequences. *Nucleic Acids Res*. (2002). **30,** 325-7. doi: 10.1093/nar/30.1.325. PMID: 11752327; PMCID: PMC99092.
30. Jiangtao, C., Yingzhen, K., Qian, W., Yuhe, S., Daping, G, Jing, L.V. & Guanshan, L. Mapgene2chrom, a tool to draw gene physical map based on perl and svg languages. *Hereditas.* (2015). **37,** 91-97.
31. Szklarczyk, D., Gable, A. L., Lyon, D., Junge, A., Wyder, S., Cepas, J. H., Simonovic, M., Doncheva, N. T., Morris, J. H. , Bork, P., Jensen, L. J. & von Mering, C. STRING v11: protein-protein association networks with increased coverage, supporting functional discovery in genome wide experimental datasets. *Nucleic Acids Res.* (2019). **47**, (D1):D607-D613.
32. Gao, M., Liu, J., Bi, D., Zhang, Z., Cheng, F., Chen, S. & Zhang, Y. MEKK1, MKK1/MKK2 and MPK4 function together in a mitogen-activated protein kinase cascade to regulate innate immunity in plants. *Cell Res.* (2008). **18,** 1190–1198 (2008).
33. Kong, Q., Qu, N., Gao, M., Zhang, Z., Ding, X., Yang, F., Li, Y., Dong, O. X, Chen, S., Li, X. & Zhang, Y. The MEKK1-MKK1/ MKK2-MPK4 kinase cascade negatively regulates immunity mediated by a mitogen-activated protein kinase kinase kinase in Arabidopsis. *Plant Cell.* (2012). **24,** 2225–2236
34. Pitzschke, A., Djamei, A., Bitton, F. & Hirt, H. A major role of the MEKK1- MKK1/2-MPK4 pathway in ROS signalling. *Mol Plant.* (2009). **2,** 120–137
35. Murakami-Kojima, M., Nakamichi, N., Yamashino, T. & Mizuno, T. The APRR3 component of the clock-associated APRR1/TOC1 quintet is phosphorylated by a novel protein kinase belonging to the WNK family, the gene for which is also transcribed rhythmically in Arabidopsis thaliana. *Plant Cell Physiol.* (2002). **43,** 675–683
36. Wang, Y., Liu, K., Liao, H., Zhuang, C., Ma, H. & Yan, X. The plant WNK gene family and regulation of flowering time in Arabidopsis. *Plant Biol.* (2008). **10,** 548– 562

37. Goyal, R. K., Tulpan, D., Chomistek, N., González-Peña Fundora, D., West, C., Ellis, B. E., Frick, M., Laroche, A. & Foroud, N. A. Analysis of MAPK and MAPKK gene families in wheat and related Triticeae species. *BMC Genomics*. (2018). **19,** 178
38. Taft, R. J., Pheasant, M. & Mattick, J. S. The relationship between non-proteincoding DNA and eukaryotic complexity. *BioEssays.* (2007). **29,** 288–299
39. Liu, Z., Shi, L., Liu, Y., Tang, Q., Shen, L., Yang, S., Cai, J., Yu, H., Wang, R., Wen, J., Lin, Y., Hu, J., Liu, C., Zhang,Y., Mou, S. & He, S. Genome wide identification and transcriptional expression analysis of mitogen-activated protein kinase and mitogen-activated protein kinase kinase genes in *Capsicum annuum*. *Front. Plant Sci.* (2015). **6**, 780 doi: 10.3389/fpls.2015.00780.
40. Zhang, G., Li, C., Li, Q., Li, B., Larkin, D. M., Lee, C., Storz, J. F., Antunes, A., Greenwold, M. J., Meredith, R.W. et al. Comparative genomics reveals insights into avian genome evolution and adaptation. *Science.* (2014). **346,** 1311–20
41. Fedorova, L. & Fedorov, A. Introns in gene evolution. In: Long M, editor. Origin and evolution of new gene functions. Dordrecht: Springer Netherlands, (2003). pp. 123–131
42. Deutsch, M. & Long, M. Intron-exon structures of eukaryotic model organisms. *Nucleic Acids Res.* (1999). **27,** 3219-3228
43. Wu, A., Hao, P., Wei, H., Sun, H., Cheng, S., Chen, P., Ma, Q., Gu, L., Zhang, M., Wang, H. and Yu, S. Genome-Wide Identification and Characterization of Glycosyltransferase Family 47 in Cotton. *Front. Genet.* (2019).10:824. doi: 10.3389/fgene.2019.00824.
44. Liu, Z., An, C., Zhao, Y., Xiao, Y., Bao, L., Gong, C., & Gao, Y. Genome-Wide Identification and Characterization of the CsFHY3/FAR1 Gene Family and Expression Analysis under Biotic and Abiotic Stresses in Tea Plants (Camellia sinensis). *Plants* (2021). **10,** 570. https://doi.org/ 10.3390/plants1003057
45. Liu, W., Li, W., He, Q., Daud, M. K., Chen, J. & Zhu, S. Genome-wide survey and expression analysis of Calcium-Dependent Protein Kinase in *Gossypium raimondii*. *PLoS One.* (2014). **9,** e98189
46. Pearson, W. R. An introduction to sequence similarity ("homology") searching. *Current protocols in bioinformatics.* (2013). doi:10.1002/0471250953.bi0301s42
47. Gan, H. H., Perlow, R. A., Roy, S., Ko, J., Wu, M., Huang, J., Yan, S., Nicoletta, A., Vafai, J., Sun, D., Wang, L., Noah, J. E., Pasquali, S. & Schlick, T. Analysis of protein sequence/structure similarity relationship. *Biophysical Journal.* (2002). **83,** 2781-2791
48. Wang, X. C., Zhao, Q. Y., Ma, C. L., Zhang, Z. H., Cao, H. L, Kong, Y. M., Yue, C., Hao, X. Y., Chen, L., Ma, J. Q., Jin, J. Q., Li, X. & Yang, Y. J. Global transcriptome profiles of Camellia sinensis during cold acclimation. *BMC Genomics.* (2013). **14,** 415
49. Zhang, Q., Cai, M., Yu, X., Wang, L., Guo, C., Ming, R. & Zhang, J. Transcriptome dynamics of Camellia sinensis in response to continuous salinity and drought stress. *Tree Genetics & Genomes.* (2017). **13,** 78
50. Bordoloi, K.S., Krishnatreya, D.B., Baruah, P.M. *et al.* Genome-wide identification and expression profiling of chitinase genes in tea (*Camellia sinensis* (L.) O. Kuntze) under biotic stress conditions. *Physiol Mol Biol Plants* (2021). **27,** 369–385.
51. Moustafa, K., AbuQamar, S., Jarrar, M., Al-Rajab, A. J. & Trémouillaux-Guiller, J. MAPK cascades and major abiotic stresses. *Plant Cell Rep.* (2014). **33,** 1217–1225
52. Wang, L., Hu, W., Tie, W., Ding, Z., Ding, X., Liu, Y., Yan, Y., Wu, C., Peng, M., Xu, B. & Jin, Z. The MAPKKK and MAPKK gene families in banana: identification, phylogeny and expression during development, ripening and abiotic stress. *Sci Rep.* (2017). **7,** 1159

53. Nuruzzaman, M., Manimekalai, R, Sharoni, A. M., Satoh, K., Kondoh, H., Ooka, H. & Kikuchi, S. Genome-wide analysis of NAC transcription factor family in rice. *Genes.* (2010). **465,** 30-44
54. Ye, J., Yang, H., Shi, H., Wei, Y., Tie, W., Ding, Z., Yan, Y., Luo, Y., Xia, Z., Wang, W., Peng, M., Li, K., Zhang, H. & Hu, W. The MAPKKK gene family in cassava: Genome-wide identification and expression analysis against drought stress. *Sci Rep.* (2017).**7,** 14939. https://doi.org/10.1038/s41598-017-13988-8.
55. Wang, G., Lovato, A., Polverari, A., Wang, M., Liang, Y. H., Ma, Y. C. & Cheng, Z. M. Genome-wide identifcation and analysis of mitogen activated protein kinase kinase kinase gene family in grapevine (Vitis vinifera). *BMC Plant Biol.* (2014). **14,** 219
56. Virk, N., Li, D., Tian, L., Huang, L., Hong, Y., Li, X., Zhang, Y., Liu, B., Zhang, H. & Song, F. Arabidopsis Raf-like mitogen-activated protein kinase kinase kinase gene Raf43 is required for tolerance to multiple abiotic stresses. *PLoS One.* (2015). **10,** e0133975.
57. Jia, H., Hao, L., Guo, X., Liu, S., Yan, Y. & Guo, X. A Raf-like MAPKKK gene, GhRaf19, negatively regulates tolerance to drought and salt and positively regulates resistance to cold stress by modulating reactive oxygen species in cotton. *Plant Sci.* (2016). **252,** 267–281
58. Shitamichi, N., Matsuoka, D., Sasayama, D., Furuya, T. & Nanmori, T. Over-expression of MAP3Kδ4, an ABA-inducible Raf-like MAP3K that confers salt tolerance in Arabidopsis. Plant *Biotechnol.* (2013). **30,** 111–118
59. Shou, H., Bordallo, P. & Wang, K. Expression of the Nicotiana protein kinase (NPK1) enhanced drought tolerance in transgenic maize. *J. Exp. Bot.* (2004). **55,** 1013–1019
60. Mukhopadhyay, M., Mondal, T.K. & Chand, P.K. Biotechnological advances in tea (*Camellia sinensis* [L.] O. Kuntze): a review. *Plant Cell Rep* (2016). **35,** 255–287.
61. Tuteja, N. Abscisic acid and abiotic stress signaling. *Plant Signal Behav.* (2007). **2,** 135–138
62. Ning, J., Li, X., Hicks, L. M. & Xiong, L. A Raf-like MAPKKK gene DSM1 mediates drought resistance through reactive oxygen species scavenging in rice. *Plant Physiol.* (2010). **152,** 876–890.
63. Mishra, N.S., Tuteja, R., and Tuteja, N. Signaling through MAP kinase networks in plants. *Arch. Biochem. Biophys*. (2006). **452**, 55–68. doi: 10.1016/j.abb.2006. 05.001.
64. Zhang, A., Jiang, M., Zhang, J., Tan, M., and Hu, X. Mitogen-activated protein kinase is involved in abscisic acid-induced antioxidant defense and acts downstream of reactive oxygen species production in leaves of maize plants. *Plant Physiol*. (2006). **141**, 475–487. doi: 10.1104/pp.105.075416
65. Chang, L. & Karin, M. Mammalian MAP kinase signaling cascades. *Nature.* (2001). **410**, 37-40 doi: 10.1038/35065000.
66. Hadiarto, T., Nanmori, T., Matsuoka, D., Iwasaki, T., Sato, K., Fukami, Y., Azuma, T. & Yasuda, T. Activation of Arabidopsis MAPK kinase kinase (AtMEKK1) and induction of AtMEKK1–AtMEK1 pathway by wounding. *Planta.* (2006). **223,** 708–713
67. Teige, M., Scheikl, E., Eulgem, T., Dóczi, R., Ichimura, K., Shinozaki, K., Dangl, J. L. & Hirt, H. The MKK2 pathway mediates cold and salt stress signaling in Arabidopsis. *Mol Cell.* (2004). **15,** 141–152
68. Nicole, M.C., Hamel, L.P., Morency, M.J., et al. MAP-ping genomic organization and organ-specific expression profiles of poplar MAP kinases and MAP kinase kinases. *BMC Genomics*. (2006). **7**, 223
69. Asai, T., Tena, G., Plotnikova, J., Willmann, M. R., Chiu, W. L., GomezGomez, L., Boller, T., Ausubel, F. M. & Sheen J. MAP kinase signalling cascade in Arabidopsis innate immunity. *Nature.* (2002). **415,** 977–983

70. Liu, Z., Zhang, L., Xue, C., Fang, H., Zhao, J., Liu, M. Genome-wide identification and analysis of MAPK and MAPKK gene family in Chinese jujube (Ziziphus jujuba mill.). *BMC Genomics.* (2017). **18,** 855
71. Chatterjee, A., Paul, A., Unnati, G.M. et al. MAPK cascade gene family in Camellia sinensis: In-silico identification, expression profiles and regulatory network analysis. *BMC Genomics* (2020). **21**, 613
72. Takahashi, Fuminori, et al. "Drought stress responses and resistance in plants: From cellular responses to long-distance intercellular communication." *Frontiers in Plant Science* (2020). **11**, 1407
73. Liu, Y. & He, C. A review of redox signaling and the control of MAP kinase pathway in plants. *Redox Biology.* (2016). **11,** 192–204
74. Pitzschke, A. & Hirt, H. Mitogen-Activated Protein Kinases and Reactive Oxygen Species Signaling in Plants. *Plant Physiology.* (2006). **141,** 351–356.

Supporting Information:

1. **S1 Table. BLAST positives table for MEKK genes of *C. sinensis***
2. **S2 Table. BLAST positives table for Raf genes of *C. sinensis***
3. **S3 Table. BLAST positives table for ZIK genes of C. sinensis**
4. **S4 Table. Function specific list of cis-acting elements identified from 2 kbp upstream region of all the identified MEKK, Raf and ZIK genes of *C. sinensis***
5. **S5 Table. Ka/Ks ratios of MAPKKKs of MEKK subfamily in *C. sinensis***
6. **S6 Table. Ka/Ks ratios of MAPKKKs of Raf subfamily in *C. sinensis***
7. **S7 Table. Ka/Ks ratios of MAPKKKs of ZIK subfamily in *C. sinensis***
8. **S8 Table. Tissue specific expression data of tea MAPKKKs**
9. **S9 Table. Expression data of tea MAPKKKs under cold stress**
10. **S10 Table. Expression data of tea MAPKKKs under drought stress**
11. **S11 Table. Expression data of tea MAPKKKs under salt stress**
12. **S12 Table. Expression data of tea MAPKKKs under MeJA treatment**
13. **S1 Fig. Transmembrane helices of MAPKKKs of MEKK subfamily in *C. sinensis*.** TMHMM Server, v.2.0 (http://www.cbs.dtu.dk/services/TMHMM/), was used to predict the presence of transmembrane helices.
14. **S2 Fig. Transmembrane helices of MAPKKKs of Raf subfamily in *C. sinensis*.** TMHMM Server, v.2.0 (http://www.cbs.dtu.dk/services/TMHMM/), was used to predict the presence of transmembrane helices.
15. **S3 Fig. Transmembrane helices of MAPKKKs of ZIK subfamily in *C. sinensis***. TMHMM Server, v.2.0 (http://www.cbs.dtu.dk/services/TMHMM/), was used to predict the presence of transmembrane helices.
16. **S4 Fig. Motif logos of the 10 identified motifs in 59 MAPKKKs of *C. sinensis***. The motif logos were generated by MEME suite.
17. **S5 Fig. The ds/dn cumulative graph of MAPKKKs of MEKK subfamily in *C. sinensis***. SNAP server (https://www.hiv.lanl.gov/content/sequence/SNAP/SNAP.html) has been used to generate the graph.

18. **S6 Fig. The ds/dn cumulative graph of MAPKKKs of Raf subfamily in *C. sinensis*.** SNAP server (https://www.hiv.lanl.gov/content/sequence/SNAP/SNAP.html) has been used to generate the graph.
19. **S7 Fig. The ds/dn cumulative graph of MAPKKKs of ZIK subfamily in *C. sinensis***. SNAP server (https://www.hiv.lanl.gov/content/sequence/SNAP/SNAP.html) has been used to generate the graph.
20. **S8 Fig. GO analysis of all the 59 MAPKKKs in *C. sinensis*.** The results have been grouped into three main categories: Biological Process, Cellular Component and Molecular function. The y-axis represents the frequency of genes while the x-axis represents the potential functions.
21. **S9 Fig. Heat maps for tissue-specific expression patterns of A) MEKK; B) Raf and; C) ZIK genes in *C. sinensis*.** The relative expression of these genes were analysed in different tissues, by using GraphPad Prism 8 software. The level of expression was in transcript per million (TPM). The 8 different tissues are represented on the right and the tea genes are marked below.
22. **S10 Fig. Heat maps for cold stress expression patterns of A) MEKK; B) Raf and; C) ZIK genes in *C. sinensis*.** The relative expression of these genes were analysed in different stages, by using GraphPad Prism 8 software. The level of expression was in transcript per million (TPM). The different stages are represented on the right and the tea genes are marked below.
23. **S11 Fig. Heat maps for drought stress expression patterns of A) MEKK; B) Raf and; C) ZIK genes in *C. sinensis***. The relative expression of these genes were analysed in different stages, by using GraphPad Prism 8 software. The level of expression was in transcript per million (TPM). The different stages are represented on the right and the tea genes are marked below.
24. **S12 Fig. Heat maps for salt stress expression patterns of (A) MEKK (B) Raf and (C) ZIK genes in *C. sinensis*.** The relative expression of these genes were analysed in different stages, by using GraphPad Prism 8 software. The level of expression was in transcript per million (TPM). The different stages are represented on the right and the tea genes are marked below.
25. **S13 Fig. Heat maps for MeJA treatment expression patterns of (A) MEKK (B) Raf and (C) ZIK genes in *C. sinensis***. The relative expression of these genes were analysed in different stages, by using GraphPad Prism 8 software. The level of expression was in transcript per million (TPM). The different stages are represented on the right and the tea genes are marked below.
