# Supplementary material for: In-silico genome wide analysis of Mitogen activated protein kinase kinase kinase gene family in C. sinensis
Source: PLoS One. 2021 Nov 4;16(11):e0258657. doi: 10.1371/journal.pone.0258657 (PMC8568164; doi:10.1371/journal.pone.0258657)
Supplement: Attachment — Submitted filename: Comments to the Authors.doc [file pone.0258657.s003.doc]

**Comments to the Authors**

**Please find the attachments (pdf version of manuscript & supplementary files) for details.**

In this work, authors have carried out genome-wide *In-silico* identification, expressional profile and regulatory network analysis of Mitogen Activated Protein Kinase Kinase Kinase gene family in *C. sinensis* by retrieval of genome, proteome & gene expression data from TPIA database. There is no error in terms of the technical aspects of the work done through bioinformatics approach and the information generated is presented in an appropriate way. Though, the manuscript is well written, there are some minor corrections and clarifications needs to be made before it is accepted for publication that will help to improve the readability & presentation of the work. Further, authors need to do an additional work which is very common part of genome wide *In-silico* mining of gene families. The authors need to retrieve the genomic DNA sequences upstream of the transcriptional start site for each tea MAPKKK gene from TPIA database for identifying putative promoter cis-acting elements. For identifying cis elements, the authors may use PlantCARE database (http://bioinformatics.psb.ugent.be/webtools/plantcare/html/).

All the corrections needed to be done are highlighted and commented in the pdf version of the manuscript & supplementary files provided. The questions pertaining to the text in the manuscript which needs to be answered are also provided as comments in the manuscript pdf version. Please find the attached pdf version of manuscript & supplementary files for comments. Few points where correction & revision **(Minor revision)** is required are given below & commented in details in the pdf version of the manuscript and supplementary files.

1. Few grammatical and typographical errors needs to be corrected.
2. All the abbreviated forms are required to be written in their full form at the first instance of their occurrence.
3. Reframing of few sentences is required.
4. Few questions related to homologous/orthologous gene pairs and few others questions needed to be answered.
5. For some statements references need to be cited.
6. Some typographical error regarding the total number of plant tissue samples whose expression have been analysed need to be corrected.
7. Some figures caption/legend description needs to be corrected.
8. Specific tissue names whose expression was analysed under different abiotic stress treatment & MeJA treatment needs to be mentioned.
9. Throughout the discussion in many instances some sentences almost seemed to be repetitive or similar to what was already mentioned in introduction and result section. Authors are advised to cross check this and modify or reframe the sentences wherever needed.
10. Scientific names need to be italicized in the Supplementary Tables captions. For Example- *C.sinensis*.
11. In the supplementary figures: the heatmap labels, the numbers at the bottom and alphabets on the right side, each needs to be described specifically what they correspond to.

**Major Revision**

*Additional Work*: The authors need to retrieve the genomic DNA sequences upstream of the transcriptional start site for each tea MAPKKK gene from TPIA database for identifying putative promoter cis-acting elements. For identifying cis elements, the authors may use PlantCARE database (http://bioinformatics.psb.ugent.be/webtools/plantcare/html/).
