# Supplementary material for: In-silico genome wide analysis of Mitogen activated protein kinase kinase kinase gene family in C. sinensis
Source: PLoS One. 2021 Nov 4;16(11):e0258657. doi: 10.1371/journal.pone.0258657 (PMC8568164; doi:10.1371/journal.pone.0258657)
Supplement: Attachment — Submitted filename: Response to reviewers.docx [file pone.0258657.s012.docx]

To,

Dr. Ramegowda Venkategowda,

Academic Editor

PLOS ONE

Dear Dr. Ramegowda,

Please find enclosed our revised manuscript (PONE-D-21-05595) entitled “*In-silico* genome wide analysis of Mitogen Activated Protein Kinase Kinase Kinase gene family in *C. sinensis*” by Paul et al. that is being considered as a regular paper for publication in PLOS ONE.

We thank reviewers for their comments and suggestions. All the major and minor comments raised by reviewers have been addressed and the manuscript as well as the supplemental files has been revised accordingly. Text for the same has been added accordingly in the revised manuscript with detailed explanations. We have made all the required corrections in the revised manuscript as suggested by reviewer#1. In context to the second comment raised by reviewer#2, we have provided a GO enrichment analysis table for all the identified tea MAPKKKs in Supporting Information 1 file as “S8 Table” and the figure corresponding to this (S8 Fig.) has also been modified in the Supporting Information 2. We are submitting the marked version (yellow highlight) of the revised manuscript to indicate the changes made as well as an unmarked version in response to reviewer’s comments.

Hope you will find the revised manuscript suitable for publication in PLOS ONE.

Thanking you,

With regards,

Neelam Mishra

**# Reviewer 1**

The authors have substantially improved the revised manuscript and incorporated all the necessary changes required. I am satisfied with the authors response on the queries asked. However, before it is accepted for publication, I would like to know a bit about the process of orthologous gene identification authors have employed for my clarification.

1. ***Did the authors use all the Arabidopsis MEKK, Raf and ZIK genes accession one by one and looked for the orthologs in C. sinenis TPIA database Orthologous Groups search option?***

**>Response:** Yes, all the identified tea MAPKKKs (17 MEKK, 31 Raf and 11 ZIK genes) were searched one by one in the TPIA database Orthologous Groups search option ([Orthologous Groups (shengxin.ren)](http://tpdbtmp.shengxin.ren:81/OrthologousGroups.html)) to find the presence any orthologous genes between tea and Arabidopsis. The same thing has also been mentioned in the revised manuscript under the “**GO ontology analysis and functional interaction network of tea MAPKKKs**” in the “**RESULTS**” section with a line beginning with “All the identified tea MAPKKKs……….” (Page 19).

2. ***Does TPIA database Orthologous Groups search option includes all the C. sinensis gene accessions that are orthologous to other species?***

**>Response:** Yes, the TPIA database orthologous group search option includes the list of all the *C. sinensis* genes which are orthologous to 11 representative plant species, namely Kiwi (*Actinidia chinensis*), Amborella (*Amborella trichopoda*), Arabidopsis (*Arabidopsis thaliana*), Coffee (*Coffee Arabica*), African oil palm (*Elaeis guineensis*), Medicago (*Medicago truncatula*), Poplar (*Populus trichocarpa*), Peach (*Prunus persica*), Cocoa (*Theobroma cacao*), Assam tea (*Camellia teatre*) and Grape (*Vitis vinifera*). The list for the same can also be found in TPIA database ([Orthologous Groups (shengxin.ren)](http://tpdbtmp.shengxin.ren:81/OrthologousGroups.html)). This has been clarified in the revised manuscript under the section under the “**GO ontology annotation and functional interaction network**” in the “**MATERIALS AND METHODS**” section (Page 7).

**Minor Comments**

1. ***I have strikeout and underlined few words in some sentences and also provided comments on those within the revised manuscript pdf file (revised manuscript with track changes part of the pdf file). Please find the attached file. Authors are advised to incorporate the required changes.***

**>Response:** All the required changes as suggested by the reviewer have been incorporated in the revised manuscript.

2. ***The authors need to correct the legends of Fig 9, Fig 10, Fig 11, Fig 12.***

**>Response:** We thank reviewer for the comment. The figure legends of the aforementioned figures (Fig 9, Fig 10, Fig 11, Fig 12) have been corrected in the revised manuscript.

**# Reviewer 2**

**Major Comments**

1. ***The title is suggestive of a rigorous regulatory network analysis of MAPKKK in tea. However, this does not seem to be the case here. In its current form, this network analysis pipeline makes no sense because no biological knowledge about tea kinases is extracted and highlighted anywhere in the MS. Furthermore, apart from the title, several other parts of the MS are misleading. In methods, the network analysis section suggests that a network of all orthologs between tea and arabidopsis was built, but in results it seems only one gene was used as a guide.***
i. ***Explain why only one gene and why that one?***

**>Response:** In this study, the STRING server was used for constructing the functional interaction network in tea. Due to the absence of tea database in the STRING server, the Arabidopsis association model had to be employed to construct the functional interaction network (Wang et al., 2018, Chatterjee et al., 2020). In addition, studies have also shown that proteins sharing higher degree of sequence and structural similarities often tend to have similar functions (Gan et al., 2002; Wang et al., 2018). Among the 59 MAPKKKs in tea, only TEA005306.1 was found to be orthologous to AT5G55100.1. As no other genes were found to be orthologous to any other Arabidopsis MAPKKKS, AT5G55100.1 was used to build the interaction network. The interaction network thus generated helps us to predict how TEA005306.1 functionally interacts with other tea genes (Lui et al., 2021, Wang et al., 2018, Chatterjee et al., 2020)

References:

*1. Wang, YX., Liu, ZW., Wu, ZJ. et al. Genome-wide identification and expression analysis of GRAS family transcription factors in tea plant (Camellia sinensis). Sci Rep* ***8,*** *3949 (2018).*

*2. Chatterjee, A., Paul, A., Unnati, G.M. et al. MAPK cascade gene family in Camellia sinensis: In-silico identification, expression profiles and regulatory network analysis. BMC Genomics* ***21,*** *613 (2020).*

*3. Gan HH, Perlow RA, Roy S, Ko J, Wu M, Huang J, et al. Analysis of protein 844 sequence/structure similarity relationships. Biophys J. 2002;83(5):2781–91.*

*4. Liu, Z.; An, C.; Zhao, Y.; Xiao, Y.; Bao, L.; Gong, C.; Gao, Y. Genome-Wide Identification and Characterization of the CsFHY3/FAR1 Gene Family and Expression Analysis under Biotic and Abiotic Stresses in Tea Plants (Camellia sinensis). Plants 2021, 10, 570.*

ii. ***I am curious to know how many of the original 59 genes (also mentioned in the abstract) are part of the network shown?. The authors can also show the overlaps, if any, between the interacting partners of all tea kinases that match Arabidopsis kinases.***

**>Response:** The given interaction network shows 1 gene (TEA005306.1) out of all the 59 predicted MAPKKKs in tea, around which the network is constructed. The same has been mentioned in the revised manuscript under “**GO ontology analysis and functional interaction network of tea MAPKKKs**” in the “**RESULTS**” section.

The given interaction network does not include any overlapping partners between the tea kinases and Arabidopsis kinases.

iii. ***How does the network shown tell us about the predicted kinases?***

**>Response:** STRING is a database of known and predicted protein-protein interactions. The interactions include direct (physical) and indirect (functional) associations (https://string-db.org/). The main aim of the interaction network analysis was to see how the predicted kinases interact with the other genes in the tea genome (Wang et al., 2018; Chatterjee et al., 2020; Liu et al., 2021). Out of the predicted tea kinases, TEA005306.1 was found to be orthologous to one Arabidopsis kinase (AT5G55100.1), hence only this gene was used for generating the interaction network (Chatterjee et al., 2020). The interaction network thus given in the manuscript is to show how TEA005306.1 functionally interacts with other genes in the tea genome (Wang et al., 2018; Chatterjee et al., 2020; Liu et al., 2021). The text for the same has also been added in the revised manuscript under

“**GO ontology analysis and functional interaction network of tea MAPKKKs**” in the “**RESULTS**” section with a line beginning with “This was done to predict……” (Page 19).

References:

*1. Wang, YX., Liu, ZW., Wu, ZJ. et al. Genome-wide identification and expression analysis of GRAS family transcription factors in tea plant (Camellia sinensis). Sci Rep* ***8,*** *3949 (2018).*

*2. Chatterjee, A., Paul, A., Unnati, G.M. et al. MAPK cascade gene family in Camellia sinensis: In-silico identification, expression profiles and regulatory network analysis. BMC Genomics* ***21,*** *613 (2020).*

*3. Liu, Z.; An, C.; Zhao, Y.; Xiao, Y.; Bao, L.; Gong, C.; Gao, Y. Genome-Wide Identification and Characterization of the CsFHY3/FAR1 Gene Family and Expression Analysis under Biotic and Abiotic Stresses in Tea Plants (Camellia sinensis). Plants 2021, 10, 570.*

iv. ***In my opinion, a better network analysis strategy could be employed, or the title should be modified to reflect other important analysis performed (e.g. phylogenetic analysis).***

**>Response:** We thank the reviewer for his suggestion. We agree that the title of the manuscript needs to be modified to reflect other important analyses performed in this study and so the title has been changed to, “***In-silico* genome wide analysis of Mitogen Activated Protein Kinase Kinase Kinase gene family in *C. sinensis***” in the revised version as suggested by the reviewer.

2. ***The authors mention that they used GO analysis for predicting gene function. However, the authors did not perform any statistical tests to support the results. GO analysis is not really a prediction method, but in this case, it can be used to validate one’s expectation from the nature of the study (e.g. terms like ‘protein phosphorylation’). Please describe the following points in greater detail:***
i. ***Exactly how was the GO analysis performed? The link provided does not work. Did you use sequences (Blast2GO?), gene IDs, or ortholog gene IDs as input to topGO?***

**>Response:** We thank the reviewer for the comments. All the comments raised by the reviewer have been answered individually as given below.

***Exactly how was the GO analysis performed?***

**>Response:** The TPIA database provides the set of all the GO terms to which a particular tea gene is annotated (TPIA database > Tea gene annotation > Gene ontology classification). All the identified tea MAPKKKs were then searched individually to retrieve data of all the GO terms to which they have been annotated. The terms that appear for each searched gene, open up to AmiGO 2 server ([AmiGO 2: Welcome (geneontology.org)](http://amigo.geneontology.org/amigo)), displaying detailed information for the respective GO terms. This data was reconfirmed by searching the GO terms in QuickGO server (<https://www.ebi.ac.uk/QuickGO/>). The obtained GO terms were then divided into 3 main categories i.e, Biological process (BP), Cellular component (CC) and Molecular function (MF) and a graph for the same was generated. Texts for the same has been mentioned in the revised manuscript under the “**GO ontology annotation and functional interaction network**” in the “**MATERIALS AND METHODS**” section (Page 7).

***The link provided does not work***

**>Response:** We thank reviewer for the comment. The link has been updated in the revised manuscript (<https://www.ebi.ac.uk/QuickGO/>) under the “**GO ontology annotation and functional interaction network**” in the “**MATERIALS AND METHODS**” section (Page 7).

***Did you use sequences (Blast2GO?), gene IDs, or ortholog gene IDs as input to topGO?***

>**Response:** The tea MAPKKKs genes were not used as input to topGO. The GO analysis was done using the TPIA database and AmiGO 2 server as described in the response to the first comment.

ii. ***Is the overlap between tea genes with GO terms shown in Fig. S8 statistically significant? Same goes for CC and MF analysis.***

**>Response:** We thank reviewer for the comment. In order to find the statistical significance of the obtained GO terms, we have conducted a statistical analysis using TeaCoN database (Zhang et al., 2020). A GO statistics table has been added in Supporting Information File 1, titled **“S8 Table: The GO enrichment analysis of all the 59 MAPKKKs of *C. sinensis*”** and the GO enrichment figure has also been modified (**S8 Fig.**) in the revised Supporting Information files.

Reference:

*1. Rui Zhang, Yong Ma, Xiaoyi Hu, Ying Chen, Xiaolong He, Ping Wang, Qi Chen, Chi-Tang Ho, Xiaochun Wan, Youhua Zhang, and Shihua Zhang, TeaCoN: A database of gene co-expression network for tea plant (Camellia sinensis), BMC genomics, 2020.*

**Minor Comments**

1. ***Figure legends should not be repetitions of the methods. Please clearly explain in the legend what is shown in the plot, specially in the supplemental figures Fig. S1-3. You should not expect a general reader to already know the outputs of TMHMM Server. What do probabilities above 1 mean? What are the red peaks?***

**>Response:** We completely agree with the reviewer and we thank reviewer for the comment. The plots of figure S1-S3 show the posterior probabilities of inside/outside/TM helix. The plot is obtained by calculating the total probability that a residue sits in helix, inside, or outside summed over all possible paths through the model. The red peaks represent the possible transmembrane helices present. If the red peak crosses the transmembrane line (represented in pink colour), then it’s calculated as a transmembrane helix. The required modification has been made and the legends are revised for the Supplementary figures as suggested by the reviewer.

2. ***The tradition set by the original creators of GO is to refer to them as ‘ontologies’ and ‘terms’, rather than ‘groups’ and ‘subgroups’, respectively. Please rephrase to stick with the convention.***

**>Response:** We thank the reviewer for this comment. The required correction has been made in the revised manuscript under the **GO ontology analysis and functional interaction network of tea MAPKKKs**” in the “**RESULTS**” section (Page 18).

3. ***In the abstract, the sentence “..on the basis of orthologous genes in Arabidopsis, functional interaction was carried out in C. sinensis.” is suggestive of wet-lab experiments. Please rephrase to clearly reflect network analysis of one of the 59 genes the story revolves around.***

**>Response:** The suggested modification has been and marked in the revised manuscript in the “**ABSTRACT**” section with a line beginning with “Also, on the basis of orthologous…….” (Page 2).

4. ***Page 19: I believe that the lines between “Similarity search programs like BLAST…...tend to possess similar functions” are to provide a logical reasoning for your approach of using Arabidopsis network in STRING. If this is the case, please rephrase these lines to reflect the exact logic. It’s somewhat confusing in its current form.***

**>Response:** We thank the reviewer for suggesting the above modification. The required change has been made and marked in the revised manuscript in the “**GO ontology analysis and functional interaction network of tea MAPKKKs**” section with a line beginning with “All the identified tea MAPKKKs were searched in the TPIA……….” (Page 19).
