# Supplementary material for: In-silico genome wide analysis of Mitogen activated protein kinase kinase kinase gene family in C. sinensis
Source: PLoS One. 2021 Nov 4;16(11):e0258657. doi: 10.1371/journal.pone.0258657 (PMC8568164; doi:10.1371/journal.pone.0258657)
Supplement: Attachment — Submitted filename: reviewer comments.doc [file pone.0258657.s008.doc]

Dear Authors,

Congratulation for your work!

 The study is highly generalized with genome wide identification of MAPKKK in tea. This major finding is preliminary. So I would suggest a major revision for this work. The following points can enhance the importance of the study:

1)  Few MAPKKK genes identified In-silico genome wide analysis from *C. sinensis* genome in this study should be validated by wet lab.

2) The identified MAPKKK genes in different subfamilies should not just be based on their phylogenetic relationships.

3) The network of functionally interacting genes should also include the genes that have experimentally validated support.

4) Expression profile analysis by qRT-PCR is required to reveal the involvement of the tea MAPKKK genes in various tissues during development and under various abiotic stress stimuli and plant hormonal treatment.

5) The study should decipher a model of signalling mechanism mediated by MAPKKK genes cascade in tea.
